# Genetic and chemical diversity of *Uncaria tomentosa* (Willd. ex. Schult.) DC. in the Brazilian Amazon

**DOI:** 10.1371/journal.pone.0177103

**Published:** 2017-05-05

**Authors:** Isabela Cristina Gomes Honório, Bianca Waleria Bertoni, Mariana Pires de Campos Telles, Ramilla dos Santos Braga, Suzelei de Castro França, Juliana da Silva Coppede, Valéria Siero Conde Correa, José Alexandre Felizola Diniz Filho, Ana Maria Soares Pereira

**Affiliations:** 1 Universidade Estadual Paulista “Júlio de Mesquita Filho”, Campus Lageado, Botucatu, São Paulo, Brazil; 2 Unidade de Biotecnologia, Universidade de Ribeirão Preto, Ribeirânia, Ribeirão Preto, São Paulo, Brazil; 3 Escola de Ciências Agrárias e Biológicas, Pontifícia Universidade Católica de Goiás, Goiânia, Goiás, Brazil; 4 Laboratório de Genética & Biodiversidade, Universidade Federal de Goiás, Goiânia, Goiás, Brazil; 5 Reserva EcoCerrado Brasil, Araxá, Minas Gerais, Brazil; 6 Departamento de Ecologia, ICB, Universidade Federal de Goiás, Goiânia, Goiás, Brazil; Centro de Pesquisas Rene Rachou, BRAZIL

## Abstract

*Uncaria tomentosa* (Willd. ex Schult.) DC., a plant native to the Amazon region, is used widely in popular medicine and by the pharmaceutical industry because of its anti-inflammatory activity. However, the survival of this species is endangered by deforestation and indiscriminate collection, and a preservation plan is urgently required. The objectives of this study were to determine the genetic and chemical variability between and within eight populations of *U*. *tomentosa* from the Brazilian states of Acre, Pará and Amapá, and to investigate possible correlations between genetic and geographical distances, and between geographical distances or altitude and the accumulation of bioactive oxindole alkaloids. Three sequence-related amplified polymorphism (SRAP) markers were employed to fingerprint genomic DNA, and the amounts of mitraphylline and isomitraphylline in leaf samples were established by high-performance liquid chromatography. Although significant divergence existed between the tested populations (F_ST_ = 0.246), the largest genetic diversity and the highest percentage of polymorphism (95.68%) was found within the population from Mâncio Lima, Acre. Gene flow was considered rather limited (Nm = 1.57), and no correlations between genetic and geographical distances were detected, suggesting that population structure followed an island model. Accumulations of mitraphylline and isomitraphylline varied in the range 32.94 to 0.57 and 3.75 to 0.36 mg g^-1^ dry weight, respectively. The concentration of isomitraphylline was positively influenced by altitude, such that the population collected at the site with the highest elevation (Tarauacá, Acre) exhibited the greatest alkaloid content. SRAP markers were very efficient in fingerprinting genomic DNA from *U*. *tomentosa* populations and clearly showed that genetic variability within populations was greater than between populations. A conservation and management plan should prioritize the creation of germplasm banks to prevent the loss of existing genetic variability, particularly within alkaloid-rich populations such as those of Tarauacá.

## Introduction

*Uncaria tomentosa* (Willd. ex Schult.) DC. (Rubiaceae) is indigenous to the Amazon biome and is distributed in various South American countries, including Brazil where it is found chiefly in the states of Acre, Amapá, Amazonas and Pará [1]. The species exhibits significant anti-inflammatory activity, along with anticancer, antidiabetic, antimicrobial, antioxidant and immunostimulant properties, and may be effective in the treatment of Parkinson disease [2]. For these reasons, *U*. *tomentosa* is commercialized worldwide and is included in the National List of Essential Medicines (*Relação Nacional de Medicamentos Essenciais*, RENAME) disseminated by the Brazilian Ministry of Health to all municipalities through the National Health System (*Sistema Único de Saúde*, SUS) [3].

*U*. *tomentosa* is rich in alkaloids and contains various representatives of this class of natural products including tetracyclic indole (corynanthein, dihydrocorynanthein, hirsutein and hirsutin), tetracyclic oxindole (corynoxeine, isocorynoxeine, rhyncophylline, isorhyncophylline, rotundifolin and isorotundifolin), pentacyclic indole (acuamigin, augustine, augustoline, isoamalicine, tetrahydroalstonin), pentacyclic oxindole (mitraphylline, isomitraphylline, pteropodine, isopteropodine, speciophylline and uncarine F) and glycoindole (3α-dihydrocadambine and dolichantosin) alkaloids [4–6]. Mitraphylline is considered the chemical marker of the therapeutically important components accumulated by *U*. *tomentosa* [7].

The extensive deforestation that has occurred in the Amazon region, along with indiscriminate collection and lack of concern about the regeneration of *U*. *tomentosa*, has compromised the survival of the species in the wild [7]. To date, there have been no reports concerning the conservation of natural populations of *U*. *tomentosa*, making it difficult to devise strategies for the preservation of this important medicinal plant. In this context, studies on the genetic diversity of medicinal plants, and on the association between findings obtained by molecular techniques and chemical marker analyses, could contribute to the maintenance and management of resources that are in particular demand by the pharmaceutical industry [8,9].

Among the various molecular techniques available for comparing DNA profiles of individuals and populations, the sequence-related amplified polymorphism (SRAP) method is particularly simple to use and provides results that are highly reliable and reproducible. Moreover, SRAP markers may be employed to target genetic variation in open reading frame (ORF) sequences of plant genomes [10].

The objectives of this study were, therefore: (i) to determine the genetic and chemical variability between and within eight populations of *U*. *tomentosa* collected in the Amazon region of Brazil, (ii) to establish correlations between genetic and geographical distances, and (iii) to investigate possible correlations between geographic distances or altitude and the accumulation of mitraphylline/isomitraphylline. The results of the study showed that SRAP markers are very efficient for fingerprinting *U*. *tomentosa* genotypes, and also afforded important indicators regarding strategies for the conservation and management of this important medicinal plant.

## Materials and methods

### Plant material and sampling

Plants of *Uncaria tomentosa* were identified by Dr. Piero Giuseppe Delprete, of Herbier de Guyane, Institut de Recherche pour le Développement. A voucher specimen was deposited in the Herbarium of Medicinal Plants at UNAERP with voucher number HPMU 2904–3066.

**Fig 1:**
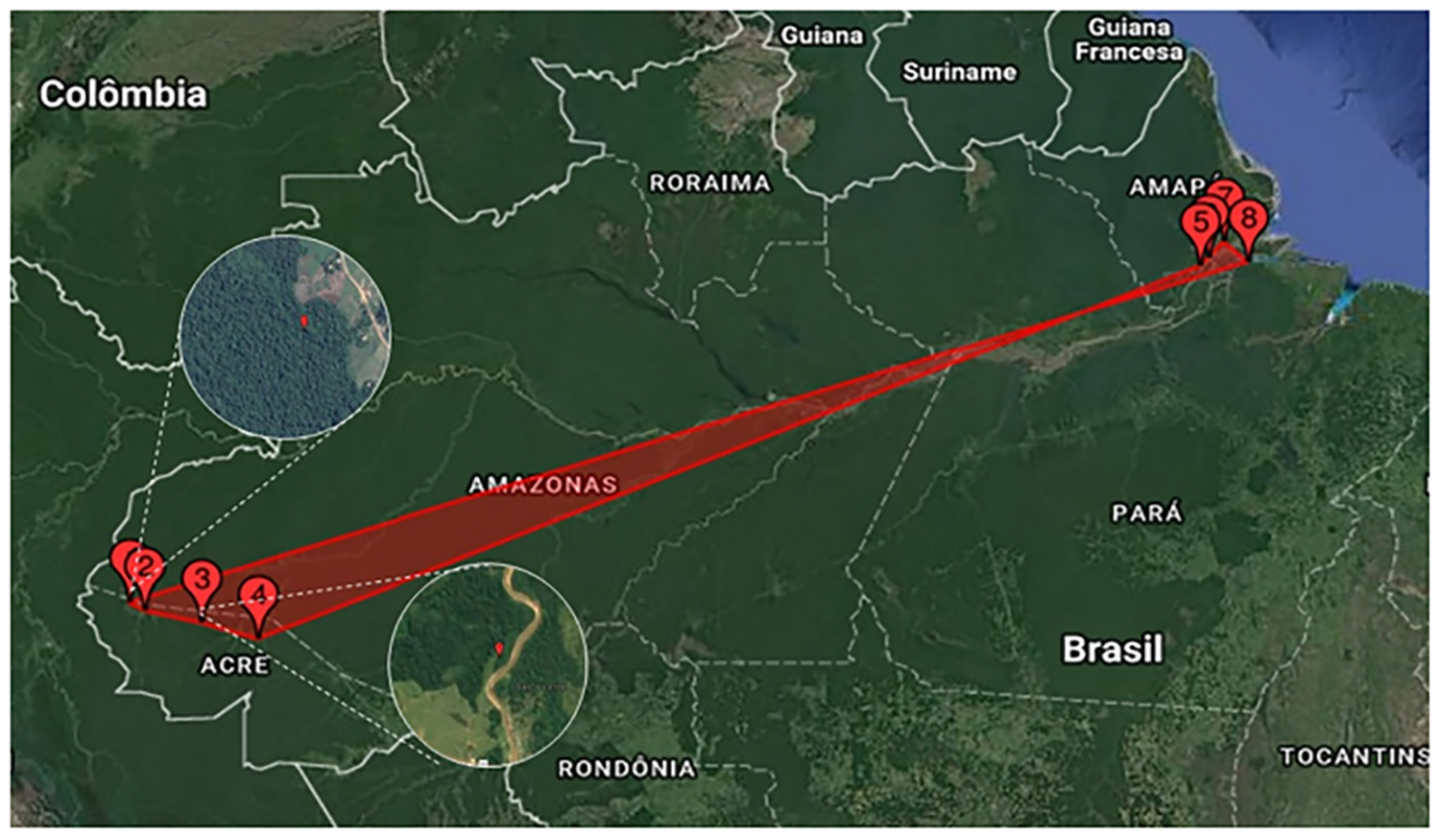
Sampling sites of *Uncaria tomentosa* populations located in the Amazon region of Brazil. The sites of collection of the eight populations collected found in the states of Acre (AC), Amapá (AP) and Pará (PA) are showed on the map (1–8), 1- Mâncio Lima, AC; 2- Cruzeiro do Sul, AC; 3- Tarauacá, AC 4-Feijó, AC; 5- Mazagão, AP; 6- Santana, AP; 7- Macapá, AP and 8-Afuá, PA. Populations with the largest (1) and smallest (3) genetic variability are evinced by white circles. (Map source: https://eros.usgs.gov/).

A total of 160 specimens of *U*. *tomentosa* were collected from eight populations (20 specimens per population) located at different sampling sites in the Amazon region of Brazil, and coded according to location, namely Cruzeiro do Sul (UTCS), Feijó (UTFJ), Mâncio Lima (UTML) and Tarauacá (UTTA) in the state of Acre, Macapá (UTMC), Mazagão (UTMZ) and Santana (UTAS) in the state of Amapá, and Afuá (UTAF) in the state of Pará. The exact locations of the sampling sites are shown in Fig 1, and the geographical coordinates and altitudes are presented in Table 1. The collection of *U*. *tomentosa* specimens investigated in this study was previously authorized by the Brazilian Council for the Administration and Management of Genetic Patrimony (CGEN) of the Brazilian Ministry of the Environment (MMA) via the National Council for Scientific and Technological Development (CNPq—CGEN/MMA Process number:010102/2015-9).

**Table 1:** Locations, geographical coordinates and altitudes of the populations of *Uncaria tomentosa* collected in the Amazon region of Brazil.

Population codes	Municipality/State	Latitude	Longitude	Altitude (m)
UTAF	Afuá, Pará	-00°07’39.8	-50°23’19.2”	28
UTCS	Cruzeiro do Sul, Acre	-07°40’12.2	-72°38’21.1”	172
UTFJ	Feijó, Acre	-08°17’36.0”	-70°22’26.1”	167
UTMC	Macapá, Amapá	+00°19’46.3”	-50°52’14.9”	09
UTML	Mâncio Lima, Acre	-07°28’48.8”	-72°56’42.2”	175
UTMZ	Mazagão, Amapá	-00°12’11.6”	-51°21’56.7”	15
UTSA	Santana, Amapá	-00°02’12.0”	-51°12’10.9”	-10
UTTA	Tarauacá, Acre	-07°56’42.3”	-71°29’00.4”	206

Young and mature leaves were sampled from 20 individuals in each population.

For SRAP analysis, young healthy leaves were sampled from each specimen, transferred to labeled conical-bottom test tubes containing silica gel and stored in the freezer at -20°C until required for DNA extraction. For HPLC analysis, young and mature leaves were collected from each specimen, transferred to labeled paper bags and subsequently dried at 45°C in a forced-air oven (Marconi, Piracicaba, SP, Brazil).

All experiments were performed in the Molecular Biology and Phytochemical Laboratories of the Biotechnology Department at the Universidade Federal de Ribeirão Preto (UNAERP) Ribeirão Preto, SP, Brazil.

### SRAP analysis

Genomic DNA was extracted from 100 mg samples of young leaves using the cetyltrimethylammonium bromide (CTAB) method [11] with some modifications. The integrity of extracted DNA was evaluated by electrophoresis on 1% agarose gels in 1 X Tris/Borate/EDTA (TBE) buffer, while quantitative evaluation was performed spectrophotometrically with the aid of a NanoPhotometer^®^ P360 instrument (Implen, Munich, Germany).

SRAP analysis was carried out following an established protocol with five combinations of published forward (me) and reverse (em) primers [10] from which three pairs were selected for further experimentation. Polymerase chain reaction (PCR) was performed with a reaction mixture containing 1 μL of 10 X reaction buffer, 0.8 μL of MgCl_2_ (25 mmol/L), 1 μL of dNTP mixture (2.5 mmol/L), 0.4 μL of forward primer (5 μM), 0.4 μL of reverse primer (5 μM), 0.2 μL of Taq DNA polymerase (5 U μL^-1^), 1 μL of DNA template (10 ng μL^-1^ for the UTAF sample and 5 ng μL^-1^ for the remainder) and deionized water to a final volume of 10 μL. The amplification procedure involved 5 cycles of denaturation at 94°C for 1 min, annealing at 35°C for 1 min and extension at 72°C for 1 min, followed by 35 cycles of denaturation at 94°C for 1 min, annealing at 50°C for 1 min and extension at 72°C for 1 min, with a final extension step of 7 min at 72°C. Amplicons were denatured at 95°C for 5 min and submitted to denaturing 6% polyacrylamide gel electrophoresis in 1 X TBE buffer for 2.5 h at a constant voltage of 80 and maximum temperature of 50°C. The gel was stained with silver nitrate and the bands revealed with sodium carbonate [12].

### Extraction and quantification of mitraphylline and isomitraphylline

The pentacyclic oxindole alkaloids mitraphylline and isomitraphylline were extracted following the procedure described by Bertol et al. [13] with some modifications. Dried leaves from individual specimens of *U*. *tomentosa* were reduced to a fine powder in a Marconi MA048 cutting mill fitted with a 40 mesh sieve. A sample (100 mg) of the powder was mixed with 1 mL of methanol (100% pure; LojaSynth, Diadema, SP, Brazil) in an amber flask and submitted to static maceration at room temperature (22 ± 1°C) for 24 h, following which the mixture was filtered and the filtrate reduced to dryness in a fume cupboard. All extractions were performed in triplicate (S1 File).

Prior to analysis by high pressure liquid chromatography (HPLC), extracts were submitted to a solid-phase extraction (SPE) clean-up procedure on Supelco-57054 C18 cartridges (Sigma, St. Louis, MO, United States) that had been previously eluted with 1 mL of methanol followed by 1 mL of an 80:20 (*v/v*) mixture of methanol (J.T. Baker HPLC grade; Avantor Performance Materials, Center Valley, PA, USA) and Milli-Q Ultrapure water (Merck Millipore, Darmstadt, Germany). Samples (15 mg) of the dried extracts were redissolved in 1 mL of the 80: 20 (*v/v*) methanol: water mixture and applied to SPE cartridges that were subsequently eluted with 3 mL of the same solvent mixture. Aliquots (20 μL) of eluents (5 mg mL^-1^) were analyzed on a Shimadzu (Kyoto, Japan) model LC-10ADvp instrument coupled to a Shimadzu SPD-M10Avp diode array detector and fitted with a Zorbax Eclipse XDB-C18 column (150 x 4.6 mm i.d., 5 μm; Agilent, Santa Clara, CA, USA) protected by a Zorbax Eclipse XDB-C18 pre-column (4.6 x 12.5 mm i.d., 5 μm). Gradient elution was performed at room temperature (22 ± 1°C) with 10 mmol/L aqueous ammonium acetate, pH adjusted to 6.9 with triethanolamine (solvent A; Neon, São Paulo, Brazil) and acetonitrile (solvent B; J.T. Baker HPLC grade) and 10 mmol/L aqueous ammonium acetate adjusted to pH 6.9 with triethanolamine (solvent A; Neon, São Paulo, Brazil). The mobile phase was supplied at a continuous flow rate of 0.8 mL min^-1^ according to the program: 35% B between 0.01 and 18.00 min, 50% B between 18.01 and 25.00 min, 35 to 100% B from 25.01 to 40 min, and 35% B between 40.01 and 45 min. The detection wavelength was set at 245 nm and the acquired data were processed using LabSolutions Multi LC-PDA software from Shimadzu.

The content of alkaloids was determined by a previously validated HPLC-PDA method [14] using mitraphylline (LGC STANDARDS CDX 00013955–005) and isomitraphylline (Chromadex ASB-00009417-005) as external standard LOD: 0.22 μg mL-1; LOQ: 0.75 μg mL^-1^ and LOD: 0.12 μg mL-1; LOQ: 0.24 μg mL^-1^, respectively. Validation of analytical data (linearity, precision, accuracy, detection limit and quantitation limit) was carried out following the Sanitary Vigilance National Agency guidelines [15].

Calibration curves for the alkaloids were constructed using concentrations in the range of 500; 250; 125; 62.5; 31.2; 15.6; 7.8; 3.9; 1.9 μg.mL^-1^ and each of these concentrations was injected in triplicate.

Ratio of the peak areas of the mitraphylline and isomitraphylline standards were calculated and plotted against the corresponding standard concentration using linear regression of the standard curves.

### Statistical analysis

Binary data were submitted to analysis of molecular variance (AMOVA), a statistical technique that allows the decomposition of total genetic variance into its between and within population components. Descriptive analysis of total variability was obtained by calculating the percentage of polymorphic loci, Nei’s genetic diversity index (H) and Shannon's diversity index (I). The unweighted pair group method with arithmetic mean (UPGMA) was used to group populations according to genetic divergence estimated from Nei’s genetic distances [16]. Geographical distances were calculated with the help of TrackMaker software version 13.8 (Geo Studio Tecnologia, Belo Horizonte, MG, Brazil). The Mantel test was performed with 10,000 permutations in order to evaluate the correlation between genetic and geographic distance [17]. All analyses were carried out using Popgene 32 [18] and Genes version 2009.7.0 [19] software.

The variability and genetic structure of populations were investigated through principal coordinates analysis (PCoA) using the software packages GenAlEx version 6.5 [20] and STRUCTURE version 2.2.4 [21, 22]. The most likely number of population groups was established using the Bayesian model-based clustering algorithm (in which individuals are assigned to *K* population genetic clusters based on their nuclear multilocus genotypes) and the admixed ancestry model. For each run, the initial burn-in was 200,000 iterations followed by a run-length of 500,000 iterations for *K* = 1 to 10 population genetic clusters [22] (S1 Dataset).

HPLC data relating to the accumulation of mitraphylline and isomitraphylline within and between populations were submitted to analysis of variance (ANOVA) and, when significant differences were detected, mean values were compared using Scott-Knott test at 5% probability (S2 Dataset). A dendrogram was constructed using the UPGMA clustering method to establish the organization of chemical variables among the evaluated populations. The matrix-based cophenetic correlations produced by the UPGMA dendrogram were calculated using the *vegan* and *ecodist* R packages [23]. The Euclidian distance matrix of chemical data (i.e. content of mitraphylline and isomitraphylline in leaves) was correlated with geographical distance, altitude and Nei’s genetic distance matrices. Pairwise relationships between populations were estimated using a simple Mantel test with 10,000 permutations performed with the aid of *vegan*, *fields* and *ecodist* R packages.

## Results and discussion

### Genetic diversity between and within *U*. *tomentosa* populations

Amplicons were obtained with all five combinations of SRAP primer pairs tested and the three pairs that produced the highest percentage of clear polymorphic fragments in all *U*. *tomentosa* populations were selected for further experimentation. These SRAP primer pairs generated a total of 185 amplified bands with percentage polymorphism values of 97.5% with me3/em3 and 100% with me1/em6 and me5/em3. The average number of polymorphic loci per primer pair was 61.7 with me3/em3 presenting the most polymorphic loci and me1/em6 the least (Table 2). These results confirm that SRAP markers are very efficient for fingerprinting *U*. *tomentosa* genotypes, and are in accord with findings reported for other species of economic relevance such as *Passiflora edulis* f. *flavicarpa* [24], *Euterpe edulis* Mart. [25] and *Phaseolus vulgaris* L. [26].

**Table 2:** Sequence-related amplified polymorphism (SRAP) analysis of *Uncaria tomentosa*.

Primer pairs (forward/reverse)	Nucleotide sequence	Number of polymorphic loci	Percentage polymorphism
me3/em3	Forward: 5’TGAGTCCAAACCGGAAT3’	80	97.5
	Reverse: 5’GACTGCGTACGAATTGAC3’		
me1/em6	Forward 5’TGAGTCCAAACCGGATA3’	50	100
	Reverse: 5’GACTGCGTACGAATTGCA3’		
me5/em3	Forward 5’TGAGTCCAAACCGGAAG3’	55	100
	Reverse: 5’GACTGCGTACGAATTGAC3’		

As shown in Table 3, specimens from the population of *U*. *tomentosa* in Mâncio Lima, Acre (UTML) exhibited the largest genetic variability (95.68%) and the highest values of Ne, H and I indices of genetic variation, while specimens from the population in Tarauacá, Acre (UTTA) presented the smallest genetic variability (62.16%) and, consequently, lower genetic variability. UTML specimens exhibited broad stems (15–20 cm) and were collected from a preserved forest area where the plants received little light. In contrast, UTTA specimens presented thin stems (3–5 cm) and were collected from the edge of the forest where the plants received direct sunlight. Moreover, the UTTA population was characterized by young individuals, most likely descendents of the original forest, and was located in an area of anthropic activity near to the village of São Vicente on the margins of the Gregório River.

**Table 3:** Genetic parameters of the populations of *Uncaria tomentosa* collected in the Amazon region of Brazil.

Population code	Percentage polymorphic loci	Observed number of alleles (N_a_)	Expected number of alleles (N_e_)	Nei's index (H)	Shannon's index (I)
UTAF	87.57	1.87	1.30	0.2035	0.3303
UTCS	72.97	1.72	1.26	0.1726	0.2786
UTFJ	94.59	1.94	1.26	0.1852	0.3144
UTMC	81.08	1.81	1.28	0.1845	0.2975
UTML	95.68	1.95	1.50	0.3151	0.4818
UTMZ	87.03	1.87	1.44	0.2648	0.4037
UTSA	83.24	1.83	1.46	0.2766	0.4176
UTTA	62.16	1.62	1.17	0.1204	0.2031
Total	98.92	1.98	1.45	0.2836	0.4425

Population codes are defined in Table 1.

The results obtained from AMOVA (Table 4) revealed that most of the genetic variability (75%) was within *U*. *tomentosa* populations rather than between populations (25%) as shown by the F_ST_ value of 0.246 (*P* < 0.001). This genetic structure suggests that conservation plans would be more efficient and successful if a larger number of individuals were collected within populations rather than in different populations. High intra-population diversity implies greater plasticity of responses to temporary stress situations [27]. Species with efficient and diverse mechanisms of pollen and seed dispersal generally have greater genetic variability within populations than between populations [28]. Thus, the efficiency of *U*. *tomentosa* in dispersing seeds and pollen through wind and insect pollinators [29] certainly contributed to the observed results.

**Table 4:** Genetic variability between and within populations of *Uncaria tomentosa* collected in the Amazon region of Brazil. Data estimated by analysis of molecular variance (AMOVA).

Source	Degrees of freedom	Mean square error	Variance components	Percentage variability	*P*	Fixation index F_ST_
**Between populations**	7	197.646	8.571	25	< 0.001	0.246
**Within populations**	152	26.226	26.226	75		
**Total**	159		34.797	100		

Allogamous species are expected to present high genetic variability within populations because intra-population divergence is inversely proportional to gene flow, in other words when gene flow is high genetic divergence is low [28]. The high within-population variability observed in *U*. *tomentosa* suggests that the species is allogamous, although the present study was not designed to consider this issue and little is known about the mechanisms of fertilization in this species.

According to Mantel tests, geographical and genetic distances among the eight *U*. *tomentosa* populations were not correlated (Table 5), thus the genetic structure of populations is not reflected in the geographical proximity of the individuals [22]. By associating this non-significant correlation with the somewhat low F_ST_ value, which is a measure of differentiation between populations, it is possible to suggest that populations of *U*. *tomentosa* are not spatially structured but follow an island model instead. In this case, it may be assumed that the inter-population genetic differentiation is not related to the spatial heterogeneity observed in the study populations [17].

**Table 5:** Geographical and genetic distances between populations of *Uncaria tomentosa* collected in the Amazon region of Brazil.

	UTTA	UTFJ	UTMZ	UTML	UTCS	UTSA	UTMC	UTAF
UTTA	*	129	2394	170	130	2414	2466	2498
UTFJ	0.006	*	2291	295	255	2313	2360	2393
UTMZ	0.054	0.044	*	2530	2502	23.27	80.77	108
UTM^4^	0.104	0.083	0.048	*	39.44	2551	2594	2634
UTCS	0.167	0.142	0.087	0.047	*	2525	2569	2595
UTSA	0.252	0.213	0.011	0.086	0.065	*	12.09	91.75
UTMC	0.028	0.032	0.075	0.114	0.196	0.266	*	75.07
UTAF	0.144	0.120	0.086	0.849	0.035	0.097	0.166	*

Geographic distances (km) are shown above the diagonal line (*), while the genetic distances are shown below the line. Population codes are defined in Table 1.

The estimated value gene flow was low (1.57), and this may constitute a problem for the survival of this species. The existence of high gene flow among populations is of particular importance since new alleles can then be introduced into the population. As a consequence, the genetic variability between local populations would tend to decrease while genetic variability within the populations would increase [30]. In this manner, gene flow acts as evolutionary factor responsible for the homogenization of populations, thereby limiting the effects of genetic drift and local selection [31]. Anthropization and fragmentation of natural areas generate barriers that negatively affect the exchange of alleles within a population and provoke a decrease in gene flow. Eventually, individuals that would normally cross at random can no longer exchange alleles resulting in their fixation. Thus, the population becomes more vulnerable to extinction because of the high degree of kinship among individuals [32].

The higher the F_ST_ value, the lower the dispersion capacity of the species and the greater the difficulty in exchanging alleles between populations, thus increasing the probability of the populations becoming endogamous, as highlighted by Frankham et al. [33]. These researchers argued that endogamy and loss of genetic diversity are inevitable within small populations, thereby reducing their reproduction rates and survival in the short term. In addition, endogamous populations have a reduced capacity to adapt to climate change, and this increases the risk of extinction [8, 33]. The low values of N_m_ and F_ST_ found in the *U*. *tomentosa* populations of the present study reflect the environments where the specimens were collected which, despite their wide diversity of flora, were within anthroposized and fragmented landscapes.

**Fig 2:**
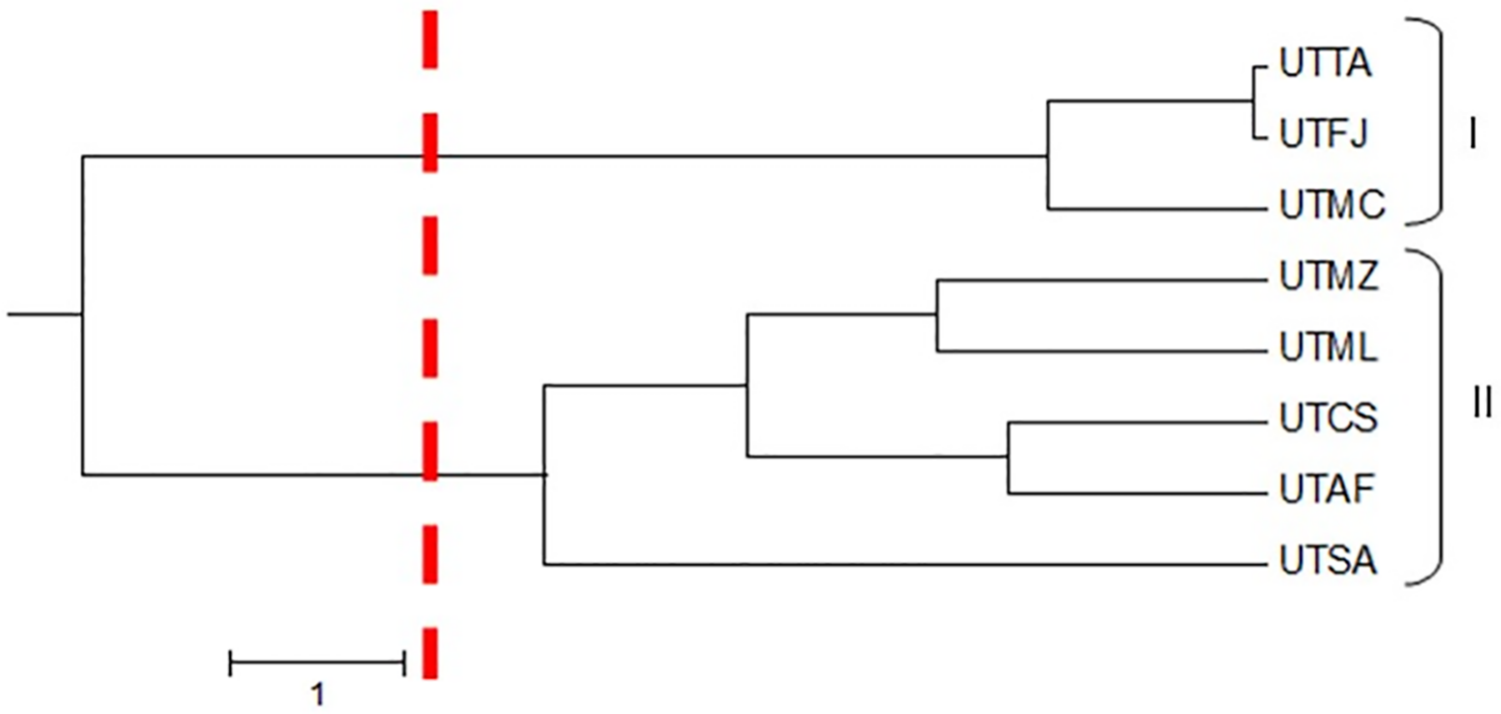
Dendrogram, constructed using the unweighted pair group method with arithmetic mean (UPGMA), showing eight populations of *Uncaria tomentosa* separated into two clusters. Group I contained UTTA (Tarauacá, Acre), UTFJ (Feijó, Acre) and UTMC (Macapá, Amapá), while group II comprised UTMZ (Mazagão, Amapá), UTML (Mâncio Lima, Acre), UTCS (Cruzeiro do Sul, Acre), UTSA (Santana, Amapá) and UTAF (Afuá, Pará).

**Fig 3:**
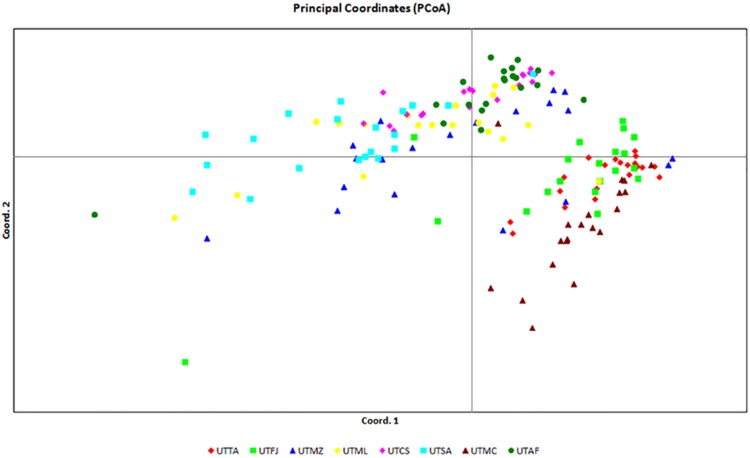
Graphical representation of principal coordinates analysis (PCoA) showing individuals (*n* = 20) belonging to eight populations of *Uncaria tomentosa* from the Amazon region of Brazil. Population sampling was carried out in the following locations: UTAF (Afuá, Pará), UTCS (Cruzeiro do Sul, Acre), UTFJ (Feijó, Acre), UTMC (Macapá, Amapá), UTML (Mâncio Lima, Acre), UTMZ (Mazagão, Amapá), UTSA (Santana, Amapá), UTTA (Tarauacá, Acre). PCoA was performed using SRAP markers.

**Fig 4:**
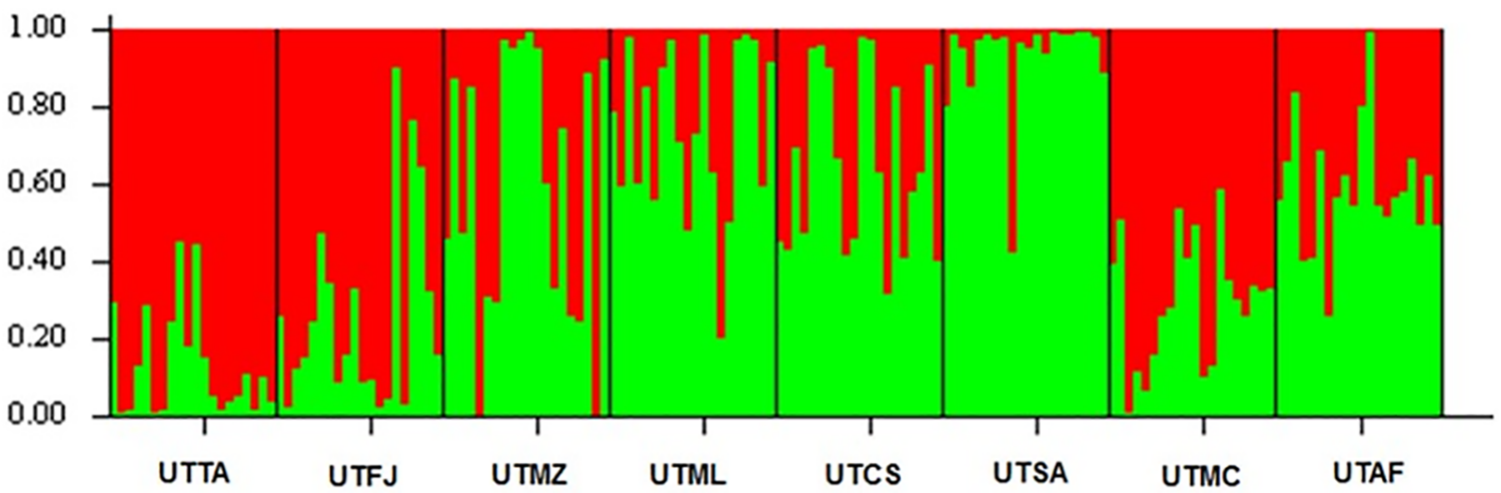
Bayesian analysis, performed using STRUCTURE software, of eight populations of *Uncaria tomentosa* from the Amazon region of Brazil showing the tendency to form two clusters. Group I (predominantly red) contained UTTA (Tarauacá, Acre), UTFJ (Feijó, Acre) and UTMC (Macapá, Amapá), while group II (predominantly green) comprised UTMZ (Mazagão, Amapá), UTML (Mâncio Lima, Acre), UTCS (Cruzeiro do Sul, Acre), UTSA (Santana, Amapá) and UTAF (Afuá, Pará).

The UPGMA dendrogram presented in Fig 2 showed that the eight populations tended to form two clusters (K = 2), one containing UTTA, UTFJ and UTMC (group I) and the other comprising UTMZ, UTML, UTCS, UTSA and UTAF (group II). This population structure was confirmed by PCoA (Fig 3) and Bayesian (Fig 4) analyses. UTTA and UTSA presented the smallest degree of admixture as shown by the coancestry values (Q = 0.88 and 0.93%, respectively). This finding indicates that there is low gene flow between the two populations, from which it may be inferred that UTTA and UTSA are most divergent one from the other and present distinct characteristics. The tendency of individuals from different populations to form clusters indicates the existence of considerable variability within the populations, as verified by the F_ST_ value. Genetic differentiation within a population, or even between distinct populations, may occur when geographical distances are relatively small and implies that genetic diversity is spatially structured [34]. However, in the case of *U*. *tomentosa*, the Mantel test showed that the correlation between genetic and geographical distance was not statistically significant (*rm* = 0.1607; *P* > 0.05). This means that the organization of genetic variability of *U*. *tomentosa* populations occurs randomly along the geographic distribution of the species and that gene flow between populations, if any, occurs over spans that are shorter than the smallest distance between the populations analyzed in this study.

Our study has shown that there is an urgent need for conservation projects involving *U*. *tomentosa*, with particular emphasis on the creation of *in vitro* and *in situ* germplasm banks to prevent the loss of existing genetic variability. The conservation of this species must be prioritized because, according to a study by Soares-Filho et al. [35], 40% of the total forests of the Amazon will have been deforested by 2050, including at least two thirds of the forest cover in the six main river basins. Moreover, it has recently been shown that 36 to 57% of all Amazonian tree species are likely to be classified as globally at risk since they meet the criteria for inclusion in the International Union for Conservation of Nature (IUCN) Red List [36]. If this is confirmed, the threat to the survival of *U*. *tomentosa* in its natural habitat will be even greater because the growth and development of this woody vine depends on the support and protection of trees.

### Variation of oxindole alkaloid content between and within *U*. *tomentosa* populations

**Fig 5:**
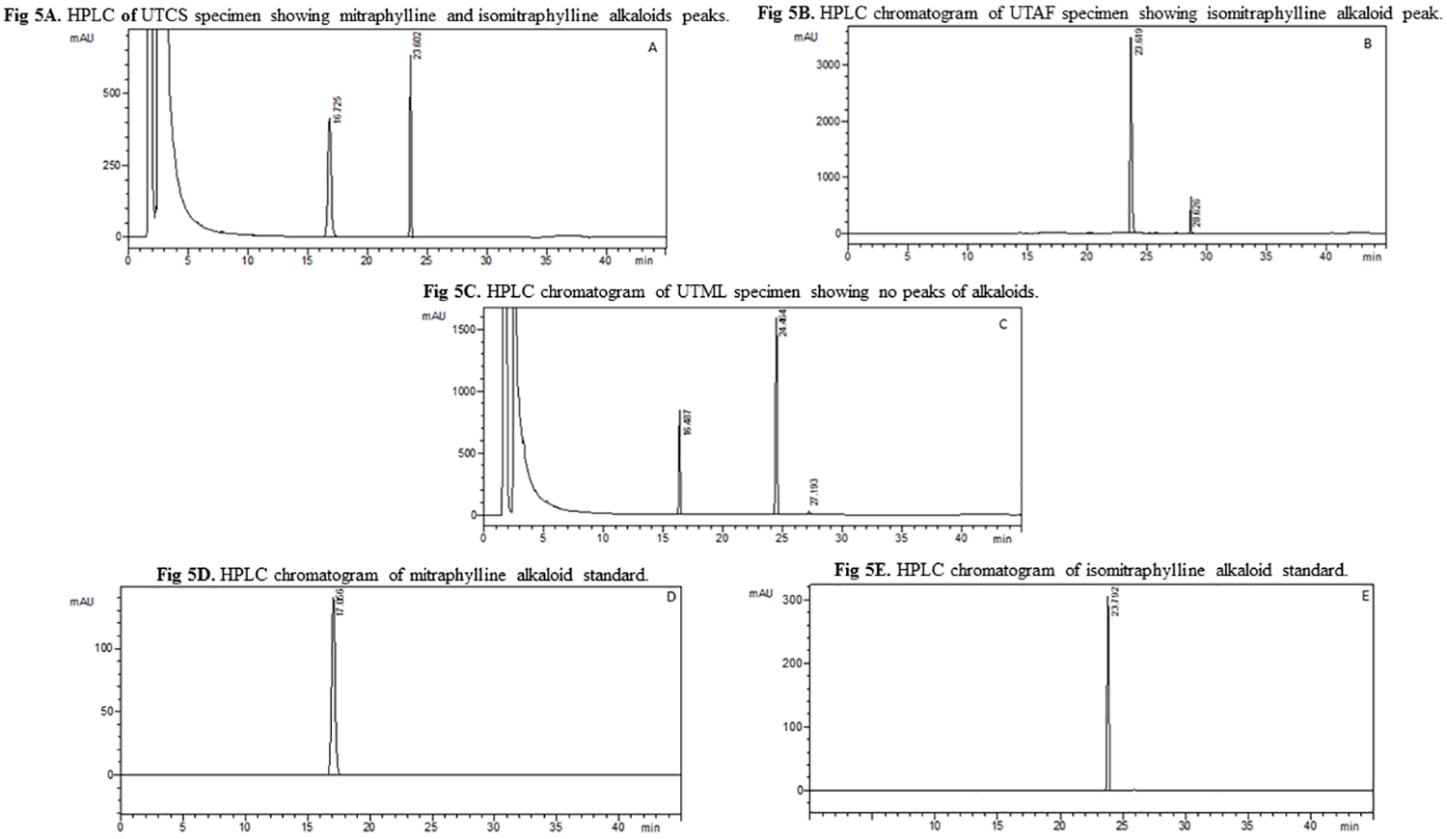
High performance liquid chromatographic (HPLC) analyses of leaf extracts of three specimens of *Uncaria tomentosa* populations from the Amazon region of Brazil. The chromatograms were obtained from specimens collected in: (**A**) Cruzeiro do Sul, AC, in which peaks labeled **a** and **b** correspond to mitraphylline and isomitraphylline, respectively; (**B**) Afuá, PA, in which the peak labeled **b** corresponds to isomitraphylline;; (**C**) Mâncio Lima, AC, in which peaks **a** and **b** are absent. Standards (D) mitraphylline and (E) isomitraphylline.

Concentrations of the pentacyclic oxindole alkaloids mitraphylline and isomitraphylline varied considerably between and within the study populations (Tables 6 and 7). Furthermore, some individuals accumulated both alkaloids (Fig 5A), while most individuals accumulated only isomitraphylline (Fig 5B) and a few accumulated neither (Fig 5C). The validation parameters are summarised in Table 8. A study involving the chemical analysis of leaves from 22 *U*. *tomentosa* plants collected at various sites in Peru suggested the existence of chemotypes since some specimens were found to accumulate pentacyclic oxindole alkaloids while others did not [37]. Quantitative and qualitative variations in the accumulation of secondary metabolites in plants are influenced by genetic and environmental factors, as well as by interactions thereof, that generate chemotypes and contribute to biological diversity [33–40].

**Table 6:** Mean concentrations of mitraphylline (Mit) and isomitraphylline (Iso) in populations of *Uncaria tomentosa* collected in the Amazon region of Brazil. In each row, mean values bearing dissimilar letters are significantly different according to Scott-Knott test at 5% probability. Data were submitted to x^½^ + 0.5 transformation. NA—not available. Population codes are defined in Table 1.

Population/alkaloid		Concentrations of pentacyclic oxindole alkaloids in individual specimens (mg g^-1^ dry weight)																			
		1	2	3	4	5	6	7	8	9	10	11	12	13	14	15	16	17	18	19	20
UTAF	**Mit**	3.32^c^	6.00^a^	2.49^d^	1.14^f^	2.73^d^	2.16^e^	0.00^g^	3.99^b^	0.00^g^	0.00^g^	0.00^g^	1.81^e^	0.81^f^	0.78^f^	0.00^g^	0.00^g^	0.00^g^	0.00^g^	0.00^g^	0.00^g^
	**Iso**	1.66^b^	1.23^c^	0.81^d^	0.78^d^	1.71^b^	1.23c	1.07c	1.70^b^	1.49^b^	0.61^d^	2.76^a^	1.43^b^	0.73^d^	0.61^d^	0.32^d^	0.96c	0.96^c^	1.02^c^	1.32^c^	1.85^b^
UTCS	**Mit**	4.09^f^	6.21^e^	6.18^e^	12.32^c^	5.79^e^	9.13^d^	5.23f	7.39^e^	5.93^e^	16.54^b^	6.11^e^	11.64^c^	11.21^c^	10.16^d^	4.27^f^	32.94^a^	5.01^f^	0.0^g^	0.0^g^	4.90^f^
	**Iso**	1.82^d^	1.55^e^	1.51^e^	4.22^d^	2.19^d^	1.89^d^	1.08^e^	1.92^d^	1.73^d^	3.90^b^	2.02^d^	2.92^c^	2.20^d^	2.11^d^	2.52^c^	7.37^a^	1.15^e^	0.46^e^	2.71^c^	1.33^e^
UTFJ	**Mit**	1.35^e^	0.91^e^	2.58^d^	0.64^e^	1.09^e^	4.05^c^	2.60^e^	1.51^d^	1.10^e^	7.20^a^	1.24^e^	2.25^d^	6.23^b^	5.45^b^	2.29^d^	NA	2.81^d^	6.68^a^	2.88^d^	3.94^c^
	**Iso**	1.51^b^	1.05^b^	1.75^b^	0.74^b^	0.83^b^	1.62^b^	2.23^a^	1.45^b^	1.26^b^	1.84^b^	0.94^b^	1.45^b^	2.61^a^	2.49^a^	1.30^b^	NA	1.27^b^	3.12^a^	1.22^b^	1.75^b^
UTMC	**Mit**	5.81^e^	7.09^c^	6.61^d^	7.38^c^	8.25^b^	7.96^b^	8.04^b^	10.35^a^	9.74^a^	5.62^e^	9.41^a^	8.04^b^	4.17^f^	4.37^f^	5.36^e^	6.96^c^	0.62^g^	0.57^g^	0.00^g^	0.00^g^
	**Iso**	1.73^a^	2.41^a^	2.18a	4.82^a^	3.01^a^	1.81^a^	2.16^a^	3.28^a^	1.71^a^	2.77^a^	2.98^a^	2.46^a^	2.60^a^	2.18^a^	1.60^a^	2.49^a^	0.25^a^	0.23^a^	0.00^a^	0.00^a^
UTML	**Mit**	9.01^d^	11.12^c^	12.22^b^	5.39^e^	18.03^a^	9.91^d^	5.28^e^	10.62^c^	9.21^d^	5.18^e^	0.00^f^	0.00^f^	0.00^f^	0.00^f^	0.00f	0.00f	0.00^f^	0.00^f^	0.00^f^	0.0^f^
	**Iso**	2.50^b^	2.79^b^	3.75^b^	1.64^b^	5.29^b^	2.36^b^	2.16^b^	3.48^b^	2.40^b^	1.96^b^	0.00^c^	0.00^c^	0.00^c^	8.02^a^	12.04^a^	0.00^c^	0.00^c^	0.00^c^	0.00^c^	0.01^c^
UTMZ	**Mit**	11.05^a^	5.68^c^	8.92a	7.20^b^	7.05^b^	10.26^a^	7.67^b^	6.76^b^	7.58^b^	6.55^b^	6.61^b^	4.86^c^	4.49^c^	7.49^b^	3.60^c^	3.96^c^	3.63^c^	2.28^c^	10.73^a^	4.82^c^
	**Iso**	1.85^a^	0.96^c^	1.99^a^	1.34^b^	1.26^c^	1.68^a^	1.21^c^	1.12^c^	1.15^c^	1.04^c^	1.06^c^	0.78^d^	0.76^d^	1.42^b^	0.52^d^	0.65^d^	0.60^d^	0.47^d^	1.91^a^	0.77^d^
UTSA	**Mit**	0.00^h^	0.00^h^	3.88^f^	6.88^d^	2.53^f^	7.61^c^	11.15^a^	0.0^h^	6.13^d^	5.23^e^	3.68^f^	4.58^e^	3.23^f^	7.92^c^	3.38^f^	4.68^e^	1.78^g^	7.75^c^	7.11^d^	9.54^b^
	**Iso**	2.58^a^	0.74^d^	1.08^c^	1.69^b^	1.05^c^	1.71^b^	2.44^a^	1.49^b^	1.30^c^	1.22^c^	1.06^c^	1.19^c^	0.95^d^	1.76^b^	0.76^d^	1.26^c^	0.65^d^	1.87^b^	1.63^b^	2.59^a^
UTTA	**Mit**	11.88^c^	0.00^f^	0.00^f^	0.00^f^	9.35^d^	15.61^b^	6.95^e^	14.97^b^	14.38^b^	6.91^e^	12.86^c^	1.38^f^	11.75^c^	9.81^d^	9.79^d^	9.63^d^	16.22^b^	9.16^d^	12.31^c^	19.45^a^
	**Iso**	3.15^a^	1.98^a^	3.73^a^	3.25^a^	2.03^a^	3.47^a^	1.85^a^	3.57^a^	3.15^a^	1.80^a^	3.16^a^	0.36^a^	10.0^a^	2.07^a^	2.28^a^	2.07^a^	3.67^a^	1.66^a^	2.69^a^	3.16^a^

**Table 7:** Mean concentrations of mitraphylline and isomitraphylline in populations of *Uncaria tomentosa* collected in the Amazon region of Brazil.

Population	Mitraphylline (mg g^-1^ dry weight)	Isomitraphylline (mg g^-1^ dry weight)
UTAF	1.31^b^	1.14^b^
UTCS	8.28^a^	2.31^a^
UTFJ	3.27^b^	1.61^b^
UTMC	5.65^b^	2.18^a^
UTML	4.73^b^	2.50^a^
UTMZ	6.47^b^	1.12^b^
UTSA	4.93^b^	1.43^b^
UTTA	11.17^a^	2.99^a^

In each column mean values bearing dissimilar letters are significantly different according to Scott-Knott test at 5% probability.

**Table 8:** Validation data for mitraphylline.

	Parameter	Results
Linearity (standard)	Function	y = 70.031x + 3×10^6^
	R	0.9999
	R^2^	0.9998
Precision	Intra-day	
	3.57 ± 0.02, lower concentration	RSD ≤ 0.60% (n = 3)
	32.14 ± 0.64, medium concentration	RSD ≤ 2.00% (n = 3)
	252.30 ± 4.21, higher concentration	RSD ≤ 1.67% (n = 3)
Accuracy	3.57 ± 0.02 (3.50 μg.mL^-1^ theoretical concentration)	101.86% (n = 3)
	32.14 ± 0.64 (31.25 μg.mL^-1^ theoretical concentration)	103.02% (n = 3)
	252.30 ± 4.21 (250.00 μg.mL^-1^ theoretical concentration)	100.92% (n = 3)
Detection limit	LOD	0.22 μg.mL^-1^
Quantitation limit	LOQ	0.75 μg.mL^-1^

In the present study, mitraphylline accumulation was detected in 81.2% of the *U*. *tomentosa* specimens analyzed, while isomitraphylline was detected in 94.3% individuals. Plants from the UTTA and UTCS populations generally presented higher levels of these alkaloids compared with individuals from other populations (Table 6). Indeed, remarkably high concentrations of mitraphylline and isomitraphylline (32.94 and 7.97 mg g^-1^ dry weight, respectively) were detected in one plant from the UTCS population. In contrast, half of the specimens from the UTAF population did not accumulate mitraphylline, and this explains the very low mean concentration of the alkaloid in this population compared with the others (Table 7).

There was no correlation between genetic and chemical similarities, considering that the individuals of genetic group I (i.e. UTTA, UTFJ and UTMC individuals) exhibited varying concentrations of mitraphylline and isomitraphylline. This finding demonstrates that the genes involved in the production of these alkaloids may be more expressed in one population than in the others. Thus, while the UTTA population occupied a prominent position in relation to the remainder regarding alkaloid content, it presented the lowest genetic variability.

**Fig 6:**
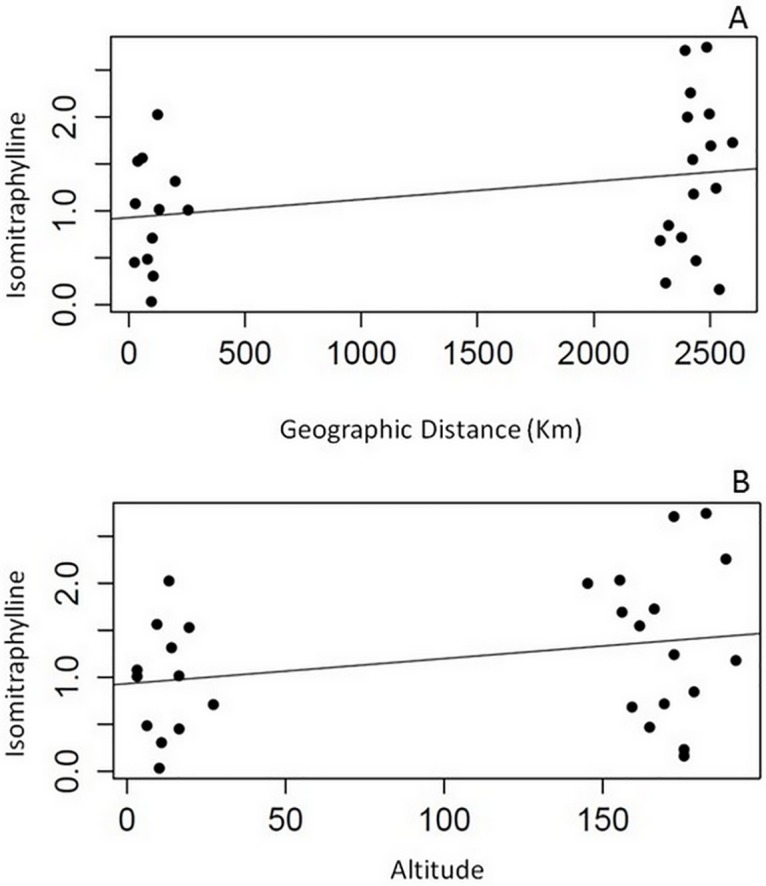
Correlations between geographical distance or altitude and the accumulation of isomitraphylline in *Uncaria tomentosa* populations from the Amazon region of Brazil. (**A**) Geographical distance *vs*. isomitraphylline accumulation (*rm* = 0.3005; *P* < 0.0344); and (**B**) Altitude *vs*. isomitraphylline accumulation (*rm* = 0.2828; *P* < 0.0471). Pairwise relationships between populations were estimated using a simple Mantel test with 10,000 permutations performed with the aid of *vegan*, *fields* and *ecodist* R packages.

Matrix correlation analyses revealed that there were no correlations between geographic distance or altitude and the accumulation of mitraphylline. On the other hand, there was a positive and statistically significant correlation (*rm* = 0.3005; *P* < 0.0344) between geographical distance and isomitraphylline accumulation. Although this correlation was not strong, it suggests that geographic distance may be an important factor in the accumulation of isomitraphylline in *U*. *tomentosa*, since populations in close proximity to each other accumulated similar amounts of this alkaloid (Fig 6A). A positive and statistically significant correlation (*rm* = 0.2828; *P* < 0.0471) was also observed between altitude and isomitraphylline accumulation, signifying that the content of this alkaloid increased slightly at higher altitude (Fig 6B). The effect of altitude on the production of various types of alkaloids has been demonstrated in several plants [41–43]. A study on *U*. *tomentosa* bark samples collected in Peru showed that at lower altitudes tetracyclic oxindole alkaloids accumulated, whereas at higher altitudes the pentacyclic oxindole alkaloids predominated [44]. The results presented herein provide further evidence of the positive effect of altitude on the accumulation of pentacyclic oxindole alkaloids. At higher altitudes, solar UV radiation is more intense [45], a factor that is associated directly with the biosynthesis of some types of alkaloids [46,47]. In *Catharanthus roseus*, for example, UV light has been shown to influence, through the activation of nuclear factors, the expression of the gene coding for tryptophan decarboxylase, a key enzyme in the biosynthetic pathway to the indole alkaloids [48].

## Conclusions

SRAP markers are very efficient for fingerprinting *U*. *tomentosa* genotypes. The results obtained by SRAP analysis revealed that genetic variability within *U*. *tomentosa* populations was greater than between populations and that the genetic structure of the populations followed an island model. We conclude that there is an urgent need for conservation projects involving *U*. *tomentosa*, with particular emphasis on the creation of germplasm banks. Moreover, considering that mitraphylline is the bioactive marker of *U*. *tomentosa* and that there is a high demand from the pharmaceutical industry for high quality specimens of this medicinal plant, the conservation and management of the species should prioritize mitraphylline-rich populations.

## Supporting information

S1 Dataset Numerical data used for statistics. (XLS)

S2 Dataset Variance analysis data. (PDF)

S1 File Gustavo Bertol Master Degree thesis. (PDF)

## References

[1] Pereira RCA, Lopes JVM. Aspectos botânicos, etnobotânicos, agronômicos e fitoquímicos de unha-de-gato. Fortaleza: Embrapa Agroindústria Tropical 2006 http://www.cnpat.embrapa.br/cd/jss/acervo/Dc_105.pdf

[2] Zhang Q, Zhao JJ, Xu J, Feng F, Qu W. Medicinal uses, phytochemistry and pharmacology of the genus *Uncaria*. J Ethnopharmacol. 2015;173: 48–80. 10.1016/j.jep.2015.06.011 26091967

[3] Ministério da Saúde do Brasil. Relação nacional de medicamentos essenciais–RENAME 2013. 8th ed. Brasília: Secretaria de Ciência, Tecnologia e Insumos Estratégicos. 2014. http://portalsaude.saude.gov.br/images/pdf/2014/julho/09/livro-rename-2013-atualizado.pdf

[4] Laus G, Brössner D, Keplinger K. Alkaloids of Peruvian *Uncaria tomentosa*. Phytochem. 1997;45: 855–860.

[5] Falkiewicz B, Lukasiak J. Vilcacora [*Uncaria tomentosa* (Willd.) DC. and *Uncaria guianensis* (Aublet) Gmell.]–A review of published scientific literature. Case Rep Clin Pract Rev. 2001;2: 305–316. Avaiable from: http://www.samento.com.ec/sciencelib/carticles/Uncaria%20A%20review.pdf

[6] Luna-Palencia GR, Huerta-Heredia AA, Cerda-García-Rojas CM, Ramos-Valdivia AC. Differential alkaloid profile in *Uncaria tomentosa* micropropagated plantlets and root cultures. Biotechnol Lett. 2013;35: 791–797. 10.1007/s10529-012-1128-8 23296316

[7] Honório ICG, Bertoni BW, Pereira AMS. *Uncaria tomentosa* and *Uncaria guianensis* an agronomic history to be written. Cienc Rural. 2016;46: 1401–1410.

[8] Silva MS, Pereira AMS, Morel LJF, França SC, Bertoni BW. Association of loganin contents with the genetic characterization of natural populations of *Palicourea rigida* Kunth determined by AFLP molecular markers. Biochem Syst Ecol. 2013;51: 189–194.

[9] Falk DA, Knapp EE, Guerrant EO. An introduction to restoration genetics. 1st ed Washington: Society of Ecological Restoration; 2001.

[10] Li G, Quiros CF. Sequence-related amplified polymorphism (SRAP), a new marker system based on a simple PCR reaction: its application to mapping and gene tagging in *Brassica*. Theor Appl Genet. 2001;103: 455–461.

[11] Doyle JJ, Doyle JL. Isolation of plant DNA from fresh tissue. Focus. 1990;12: 13–15.

[12] Creste S, Tulmann Neto A, Figueira A. Detection of single sequence repeat polymorphisms in denaturing polyacrylamide sequencing gels by silver staining. Plant Mol Biol Rep. 2001;19: 299–306.

[13] Bertol G, Franco L, Oliveira BH. HPLC analysis of oxindole alkaloids in *Uncaria tomentosa*: Sample preparation and analysis optimization by factorial design. Phytochem Anal. 2012;23: 143–151. 10.1002/pca.1335 21809407

[14] Peñaloza EMC, Kaiser S, Resende PE, Pittol V, Carvalho AR, Ortega GG. Chemical composition variability in the *Uncaria tomentosa* (cat’s claw) wild population. Quim Nova. 2015:38: 378–386.

[15] ANVISA. 2003. Resolução‐RE n. 899, de 29 de maio de 2003. Dispõe sobre o Guia para validação de métodos analíticos e bioanalíticos. Agência Nacional de Vigilância Sanitária (http://portal.anvisa.gov.br/documents/10181/2718376/RE_899_2003_COMP.pdf/ff6fdc6b-3ad1-4d0f-9af2-3625422e6f4b

[16] Nei M. Estimation of average heterozygosity and genetic distance from a small number of individuals. Genetics. 1978;89: 583–590. 17248844 10.1093/genetics/89.3.583 PMC1213855

[17] Diniz-Filho JAF, Soares TN, Lima JS, Dobrovolski R, Landeiro VL, Telles MPC, et al Mantel test in population genetics. Genet Mol Biol. 2013;36: 475–485. 10.1590/S1415-47572013000400002 24385847 PMC3873175

[18] Yeh FC, Boyle TJB. Population genetic analysis of co-dominant and dominant markers and quantitative traits. Belg J Bot. 1997;129: 157–163.

[19] Cruz CD. GENES: a software package for analysis in experimental statistics and quantitative genetics. Acta Sci Agron. 2013;35: 271–276.

[20] Peakall R, Smouse PE. GenAlEx 6.5: genetic analysis in Excel. Population genetic software for teaching and research—an update. Bioinformatics. 2012;28: 2537–2539. 10.1093/bioinformatics/bts460 22820204 PMC3463245

[21] Pritchard JK, Stephens M, Donnelly P. Inference of population structure using multilocus genotype data. Genetics 2000; 155: 945–959. 10835412 10.1093/genetics/155.2.945 PMC1461096

[22] Evanno G, Regnaut S, Goudet J. Detecting the number of clusters of individuals using the software STRUCTURE: a simulation study. Mol Ecol. 2005;14: 2611–2620. 10.1111/j.1365-294X.2005.02553.x 15969739

[23] R Development Core Team. R: A language and environment for statistical computing. Vienna: R Foundation for Statistical Computing 2015 http://www.R-project.org/.

[24] Ganga RMD, Ruggiero C, Lemos EGM, Grili GVG, Gonçalves MM, Chagas EA, et al Diversidade genética em maracujazeiro-amarelo utilizando marcadores moleculares fAFLP. Rev Bras Frutic. 2004;26: 494–498.

[25] Cardoso SRS, Eloy NB, Provan J, Cardoso MA, Ferreira PCG. Genetic differentiation of *Euterpe edulis* Mart. populations estimated by AFLP analysis. Mol Ecol. 2000;9: 1753–1760. 11091311 10.1046/j.1365-294x.2000.01056.x

[26] Ceylan A, Öcal N, Akbulut M. Genetic diversity among the Turkish common bean cultivars (*Phaseolus vulgaris* L.) as assessed by SRAP, POGP and cpSSR markers. Biochem Syst Ecol. 2014;54: 219–229.

[27] Marcon G. Estrutura genética de populações de Stylosanthes humilis H. b. K. (Leguminosae) de três regiões ecogeográficas do Estado de Pernambuco. Doctoral Thesis, Escola Superior de Agricultura “Luiz de Queiroz”, Universidade de São Paulo. 1988.

[28] Loveless MD, Hamrick JL. Ecological determinants of genetic structure in plant populations. Ann Rev Ecol Syst. 1984;15: 65–95.

[29] Pereira RCA, Pinto JEBP, Bertolucci SKV, Castro EM, Silva FG. Germinação, avaliação do ácido giberélico e posição do explante no alongamento in vitro de *Uncaria guianensis* (Aublet) Gmelin Rubiaceae (Unha-de-gato). Cien Agrotec. 2006;30: 637–642.

[30] Templeton AR. Genética de populações e teoria microevolutiva. 1st ed Ribeirão Preto: Sociedade Brasileira de Genética 2011.

[31] Seoane CES, Kageyama PY, Sebben AM. Efeitos da fragmentação florestal na estrutura genética de populações de *Esenbeckia leiocarpa* Engl. (Guarantã). Sci Forest. 2000;57: 123–139. Available from: http://www.ipef.br/publicacoes/scientia/nr57/cap09.pdf

[32] Frankham R, Ballou JD, Briscoe DA. Fundamentos de genética da conservação. 1st ed Ribeirão Preto: Sociedade Brasileira de Genética 2008.

[33] Ellstrand CA, Elam DR. Population genetics on sequences of small population size: implications for plant conservation. Annu Rev Ecol Syst. 1993;24: 217–242.

[34] Linhart YB, Mitton JB, Sturgeon KB, Davis ML. Genetic variation in space and time in a population of ponderosa pine. Heredity. 1981;46: 407–426.

[35] Soares-Filho BS, Nepstad DC, Curran LM, Cerqueira GC, Garcia RA, Ramos CA, et al Modelling conservation in the Amazon basin. Nature. 2006;440: 520–523. 10.1038/nature04389 16554817

[36] ter Steege H, Pitman NCA, Killeen TJ, Laurance WF, Peres CA, Guevara JE, et, al Estimating the global conservation status of more than 15,000 Amazonian tree species. Sci. Adv. 2015;1: e1500936 10.1126/sciadv.1500936 26702442 PMC4681336

[37] Peñaloza EMC, Kaiser S, Resende PE, Pittol V, Carvalho AR, Ortega GG. Chemical composition variability in the *Uncaria tomentosa* (cat’s claw) wild population. Quim Nova. 2015;38: 378–386.

[38] D’andrea A, Adami M, Salvatore G, Ricciardi A, Veglia J, Agrelo A, et al. Examen del aceite esencial de Lippia alba (Mill.) N. E. Br. la salvia morada de Corrientes. IX Reunión de Comunicaciones Científicas y Tecnológicas, Universidad Nacional del Nordeste (UNNE). 1995.

[39] Hu Y, Zhang Q, Xin H, Qin LP, Lu BR, Rahman K, et al Association between chemical and genetic variation of *Vitex rotundifolia* populations from different locations in China: its implication for quality control of medicinal plants. Biomed Chromatogr. 2007;21: 967–975. 10.1002/bmc.841 17474140

[40] Zhang F, Chen S, Chen F, Fang W, Deng Y, Chang Q, et al Genetic analysis and associated SRAP markers for flowering traits of chrysanthemum (*Chrysanthemum morifolium*). Euphytica. 2011;177: 15–24.

[41] Nejadhabibvash F, Rahmani F, Heidari R, Jamei R, Azimi F. Study of inheritance and environment on tropane alkaloids within *Hyoscyamus* species. Aust J Crop Sci. 2012;6: 1428–1434.

[42] Andola HC, Gaira KS, Rawal RS, Rawat MSM, Bhatt ID. Habitat-dependent variations in berberine content of *Berberis asiatica* Roxb. ex. DC. in Kumaon, Western Himalaya. Chem Biodivers. 2010;7: 415–420. 10.1002/cbdv.200900041 20151388

[43] Chandra P, Purohit AN. Berberine contents and alkaloid profile of *Berberis* species from different altitudes. Biochem Syst Ecol. 1980;8: 379–380.

[44] Torrejón GD, Martín JJG, Loayza DG, Alanoca R. Contenido de alcaloides en corteza de *Uncaria tomentosa* (Wild.) DC procedente de diferentes hábitats de la región Ucayali–Perú. Rev Soc Quím Peru. 2010;76: 271–278.

[45] Blumthaler M, Ambach W, Ellinger R. Increase in solar UV radiation with altitude. J Photochem Photobiol B Biol. 1997;39: 130–134.

[46] Lydon J, Casale JF, Kong H, Sullivan JH, Daughtry CST, Bailey B. The effects of ambient solar UV radiation on alkaloid production by *Erythroxylum novogranatense* var. *novogranatense*. Photochem Photobiol. 2009;85: 1156–1161. 10.1111/j.1751-1097.2009.00562.x 19453388

[47] Gregianini TS, da Silveira VC, Porto DD, Kerber VA, Henriques AT, Fett-Neto AG. The alkaloid brachycerine is induced by ultraviolet radiation and is a singlet oxygen quencher. Photochem Photobiol. 2003; 78: 470–474. 14653578 10.1562/0031-8655(2003)078<0470:tabiib>2.0.co;2

[48] Ouwerkerk PB, Trimborn TO, Hilliou F, Memelink J. Nuclear factors GT-1 and 3AF1 interact with multiple sequences within the promoter of the Tdc gene from Madagascar periwinkle: GT-1 is involved in UV light-induced expression. Mol Gen Genet. 1999;261: 610–622. 10394897 10.1007/s004380050003

