# Supplementary material for: Genetic and chemical diversity of Uncaria tomentosa (Willd. ex. Schult.) DC. in the Brazilian Amazon
Source: PLoS One. 2017 May 5;12(5):e0177103. doi: 10.1371/journal.pone.0177103 (PMC5419575; doi:10.1371/journal.pone.0177103)
Supplement: S2 Dataset — (PDF) [file pone.0177103.s002.pdf]

Isomitraphyline
Among population

Arquivo analisado:

C:\Users\Jordany\Desktop\isabela.dbf

Variável analisada: CONC

Opção de transformação: Variável sem transformação (Y)

TABELA DE ANÁLISE DE VARIÂNCIA

FV	GL	SQ	QM	Fc	Pr>Fc
AMOSTRA	7	9.869733	1.409962	7.033	0.0006
erro	16	3.207600	0.200475		
Total corrigido	23	13.077333			
CV (%) =	23.42				
Média geral:	1.9116667	Número de observações:	24		

Teste Scott-Knott (1974) para a FV AMOSTRA

NMS: 0,05

Média harmonica do número de repetições (r): 3
Erro padrão: 0,258505319094211

Tratamentos	Médias	Resultados do teste
Utmz	1.120000	a1
Utaf	1.140000	a1
Utsa	1.433333	a1
Utfj	1.613333	a1
Utmc	2.183333	a2
Utcs	2.306667	a2
Utml	2.503333	a2
Utta	2.993333	a2

Isomitraphyline
Within population

Arquivo analisado:

F:\Dados Tese\estatísticas\Estatistica UT\Fitoquímica\utaf iso.dbf

Variável analisada: CONC

Opção de transformação: Variável sem transformação (Y)

TABELA DE ANÁLISE DE VARIÂNCIA

FV	GL	SQ	QM	Fc	Pr>Fc
TRAT	19	17.668473	0.929920	8.077	0.0000
erro	40	4.605200	0.115130		
Total corrigido	59	22.273673			

CV (%) = 27.99
Média geral: 1.2123333 Número de observações: 60

Teste Scott-Knott (1974) para a FV TRAT

NMS: 0,05

Média harmonica do número de repetições (r): 3
Erro padrão: 0,19589963416675

Tratamentos	Médias	Resultados do teste
Utaf15	0.320000	a1
Utaf10	0.610000	a1
Utaf14	0.613333	a1
Utaf13	0.733333	a1
Utaf4	0.783333	a1
Utaf3	0.806667	a1
Utaf17	0.956667	a2
Utaf16	0.963333	a2
Utaf18	1.023333	a2
Utaf7	1.066667	a2
Utaf2	1.230000	a2
Utaf6	1.233333	a2
Utaf19	1.316667	a2
Utaf12	1.426667	a3
Utaf9	1.490000	a3
Utaf1	1.656667	a3
Utaf8	1.696667	a3
Utaf5	1.713333	a3
Utaf20	1.850000	a3
Utaf11	2.756667	a4

Variável analisada: CONC

Opção de transformação: Raiz quadrada - SQRT (Y)

TABELA DE ANÁLISE DE VARIÂNCIA

FV	GL	SQ	QM	Fc	Pr>Fc
TRAT	19	3.413978	0.179683	7.138	0.0000
erro	40	1.006956	0.025174		
Total corrigido	59	4.420934			

CV (%) = 14.87
Média geral: 1.0670760 Número de observações: 60

Teste Scott-Knott (1974) para a FV TRAT

NMS: 0,05

Média harmonica do número de repetições (r): 3
Erro padrão: 0,0916040595415593

Tratamentos	Médias	Resultados do teste
Utaf15	0.563299	a1
Utaf10	0.779819	a1
Utaf14	0.782903	a1
Utaf13	0.856213	a1
Utaf4	0.885057	a1
Utaf3	0.893266	a1
Utaf17	0.977400	a1
Utaf16	0.981169	a1
Utaf18	1.011301	a1
Utaf7	1.032778	a1
Utaf2	1.106938	a2
Utaf6	1.110213	a2
Utaf19	1.147269	a2
Utaf12	1.193772	a2
Utaf9	1.216303	a2
Utaf20	1.249114	a2
Utaf1	1.284580	a2
Utaf8	1.302500	a2
Utaf5	1.307731	a2
Utaf11	1.659892	a3

Arquivo analisado:

F:\Dados Tese\estatísticas\Estatistica UT\Fitoquímica\utcs iso.dbf

Variável analisada: CONC

Opção de transformação: Variável sem transformação (Y)

TABELA DE ANÁLISE DE VARIÂNCIA

FV	GL	SQ	QM	Fc	Pr>Fc
TRAT	19	114.415698	6.021879	27.734	0.0000
erro	40	8.685267	0.217132		
Total corrigido	59	123.100965			

CV (%) = 20.88
Média geral: 2.2315000 Número de observações: 60

Teste Scott-Knott (1974) para a FV TRAT

NMS: 0,05

Média harmonica do número de repetições (r): 3
Erro padrão: 0,269030151139649

Tratamentos	Médias	Resultados do teste
Utcs18	0.463333	a1
Utcs7	1.076667	a1
Utcs17	1.150000	a1
Utcs20	1.326667	a1
Utcs3	1.506667	a1
Utcs2	1.550000	a1
Utcs9	1.736667	a2
Utcs1	1.823333	a2
Utcs6	1.896667	a2
Utcs8	1.923333	a2
Utcs11	2.023333	a2
Utcs14	2.113333	a2
Utcs5	2.190000	a2
Utcs13	2.200000	a2
Utcs4	2.220000	a2
Utcs15	2.523333	a3
Utcs19	2.710000	a3
Utcs12	2.920000	a3
Utcs10	3.903333	a4
Utcs16	7.373333	a5

Variável analisada: CONC

Opção de transformação: Raiz quadrada - SQRT (Y)

TABELA DE ANÁLISE DE VARIÂNCIA

FV	GL	SQ	QM	Fc	Pr>Fc
TRAT	19	9.416739	0.495618	18.092	0.0000
erro	40	1.095776	0.027394		
Total corrigido	59	10.512515			

CV (%) = 11.54
Média geral: 1.4339775 Número de observações: 60

Teste Scott-Knott (1974) para a FV TRAT

NMS: 0,05

Média harmonica do número de repetições (r): 3
Erro padrão: 0,0955587026268597

Tratamentos	Médias	Resultados do teste
Utcs18	0.680298	a1
Utcs7	1.026284	a2
Utcs17	1.072049	a2
Utcs20	1.147950	a2
Utcs3	1.223832	a2
Utcs2	1.239345	a2
Utcs9	1.317621	a3
Utcs1	1.337898	a3
Utcs6	1.355835	a3
Utcs8	1.386748	a3
Utcs11	1.399171	a3
Utcs14	1.449487	a3
Utcs13	1.465311	a3
Utcs4	1.474448	a3
Utcs5	1.479241	a3
Utcs15	1.585325	a3
Utcs19	1.645827	a3
Utcs12	1.704470	a3
Utcs10	1.973657	a4
Utcs16	2.714753	a5

Arquivo analisado:

F:\Dados Tese\estatísticas\Estatistica UT\Fitoquímica\utfj iso.dbf

----

Variável analisada: CONC

Opção de transformação: Variável sem transformação (Y)

TABELA DE ANÁLISE DE VARIÂNCIA

FV	GL	SQ	QM	Fc	Pr>Fc
AMOSTRA	18	21.625825	1.201435	4.242	0.0001
erro	38	10.763200	0.283242		
Total corrigido	56	32.389025			

CV (%) = 33.23
Média geral: 1.6017544 Número de observações: 57

Teste Scott-Knott (1974) para a FV AMOSTRA

NMS: 0,05

Média harmonica do número de repetições (r): 3
Erro padrão: 0,307268669225678

Tratamentos	Médias	Resultados do teste
Utfj4	0.736667	a1
Utfj5	0.833333	a1
Utfj11	0.940000	a1
Utfj2	1.053333	a1
Utfj19	1.220000	a1
Utfj9	1.260000	a1
Utfj17	1.273333	a1
Utfj15	1.300000	a1
Utfj12	1.446667	a1
Utfj8	1.450000	a1
Utfj1	1.510000	a1
Utfj6	1.620000	a1
Utfj20	1.750000	a1
Utfj3	1.750000	a1
Utfj10	1.836667	a1
Utfj7	2.230000	a2
Utfj14	2.493333	a2
Utfj13	2.606667	a2
Utfj18	3.123333	a2

Variável analisada: CONC

Opção de transformação: Raiz quadrada - SQRT (Y)

TABELA DE ANÁLISE DE VARIÂNCIA

FV	GL	SQ	QM	Fc	Pr>Fc

AMOSTRA	18	3.159172	0.175510	4.519	0.0000
erro	38	1.475695	0.038834		
Total corrigido	56	4.634867			

CV (%) = 15.98
Média geral: 1.2330616 Número de observações: 57

Teste Scott-Knott (1974) para a FV AMOSTRA

NMS: 0,05

Média harmonica do número de repetições (r): 3
Erro padrão: 0,113774740273936

Tratamentos	Médias	Resultados do teste
Utfj4	0.852685	a1
Utfj5	0.907210	a1
Utfj11	0.967114	a1
Utfj2	1.025384	a1
Utfj19	1.104437	a1
Utfj9	1.121583	a1
Utfj17	1.123463	a1
Utfj15	1.134951	a1
Utfj6	1.158527	a1
Utfj12	1.188721	a1
Utfj8	1.202994	a1
Utfj1	1.228261	a1
Utfj3	1.317647	a2
Utfj20	1.322419	a2
Utfj10	1.347547	a2
Utfj7	1.472036	a2
Utfj14	1.576430	a3
Utfj13	1.612923	a3
Utfj18	1.763839	a3

Arquivo analisado:

F:\Dados Tese\estatísticas\Estatistica UT\Fitoquímica\utmc iso.dbf

Variável analisada: CONC

Opção de transformação: Variável sem transformação (Y)

TABELA DE ANÁLISE DE VARIÂNCIA

FV	GL	SQ	QM	Fc	Pr>Fc
TRAT	19	83.214898	4.379731	2.087	0.0250

erro	40	83.954200	2.098855
Total corrigido	59	167.169098	

CV (%) = 71.20
Média geral: 2.0348333   Número de observações: 60

Teste Scott-Knott (1974) para a FV TRAT

NMS: 0,05

Média harmonica do número de repetições (r): 3
Erro padrão: 0,836431905975216

Tratamentos	Médias	Resultados do teste
Utmc19	0.000000	a1
Utmc20	0.000000	a1
Utmc18	0.230000	a1
Utmc17	0.253333	a1
Utmc15	1.603333	a1
Utmc9	1.713333	a1
Utmc1	1.733333	a1
Utmc6	1.810000	a1
Utmc7	2.160000	a1
Utmc14	2.183333	a1
Utmc3	2.183333	a1
Utmc2	2.406667	a1
Utmc12	2.463333	a1
Utmc16	2.493333	a1
Utmc13	2.603333	a1
Utmc10	2.766667	a1
Utmc11	2.983333	a1
Utmc5	3.016667	a1
Utmc8	3.276667	a1
Utmc4	4.816667	a1

Variável analisada: CONC

Opção de transformação: Raiz quadrada - SQRT (Y)

TABELA DE ANÁLISE DE VARIÂNCIA

FV	GL	SQ	QM	Fc	Pr>Fc
TRAT	19	18.569007	0.977316	6.447	0.0000
erro	40	6.064035	0.151601		
Total corrigido	59	24.633041			

CV (%) = 30.55
Média geral: 1.2744735   Número de observações: 60

Teste Scott-Knott (1974) para a FV TRAT

NMS: 0,05

Média harmonica do número de repetições (r): 3
Erro padrão: 0,224796846889216

Tratamentos	Médias	Resultados do teste
Utmc19	0.000000	a1
Utmc20	0.000000	a1
Utmc18	0.476506	a1
Utmc17	0.502982	a1
Utmc9	1.181169	a2
Utmc15	1.266025	a2
Utmc1	1.316398	a2
Utmc6	1.345095	a2
Utmc14	1.424289	a2
Utmc7	1.461680	a2
Utmc3	1.476790	a2
Utmc2	1.550994	a2
Utmc12	1.569337	a2
Utmc16	1.578821	a2
Utmc11	1.580381	a2
Utmc13	1.582708	a2
Utmc10	1.663167	a2
Utmc5	1.699168	a2
Utmc8	1.809497	a2
Utmc4	2.004463	a2

Arquivo analisado:

F:\Dados Tese\estatísticas\Estatistica UT\Fitoquímica\utml iso.dbf

---

Variável analisada: CONC

Opção de transformação: Variável sem transformação (Y)

TABELA DE ANÁLISE DE VARIÂNCIA

FV	GL	SQ	QM	Fc	Pr>Fc
TRAT	19	548.715485	28.879762	3.901	0.0001
erro	40	296.144333	7.403608		
Total corrigido	59	844.859818			

CV (%) = 112.34
Média geral: 2.4221667 Número de observações: 60

Teste Scott-Knott (1974) para a FV TRAT

NMS: 0,05

Média harmonica do número de repetições (r): 3
Erro padrão: 1,57094539830143

Tratamentos	Médias	Resultados do teste
Utml17	0.000000	a1
Utml16	0.000000	a1
Utml19	0.000000	a1
Utml18	0.000000	a1
Utml13	0.000000	a1
Utml11	0.000000	a1
Utml12	0.003333	a1
Utml20	0.013333	a1
Utml4	1.643333	a1
Utml10	1.963333	a1
Utml7	2.163333	a1
Utml6	2.363333	a1
Utml9	2.400000	a1
Utml1	2.503333	a1
Utml2	2.796667	a1
Utml8	3.486667	a1
Utml3	3.750000	a1
Utml5	5.293333	a1
Utml14	8.023333	a2
Utml15	12.040000	a2

Variável analisada: CONC

Opção de transformação: Raiz quadrada - SQRT (Y)

TABELA DE ANÁLISE DE VARIÂNCIA

FV	GL	SQ	QM	Fc	Pr>Fc
TRAT	19	55.798246	2.936750	7.237	0.0000
erro	40	16.230916	0.405773		
Total corrigido	59	72.029162			

CV (%) = 57.63
Média geral: 1.1052966 Número de observações: 60

Teste Scott-Knott (1974) para a FV TRAT

NMS: 0,05

Média harmonica do número de repetições (r): 3
Erro padrão: 0,36777388756095

Tratamentos	Médias	Resultados do teste
Utml17	0.000000	a1
Utml16	0.000000	a1
Utml19	0.000000	a1
Utml18	0.000000	a1
Utml13	0.000000	a1
Utml11	0.000000	a1
Utml12	0.033333	a1
Utml20	0.066667	a1
Utml4	1.281730	a2
Utml10	1.400397	a2
Utml7	1.470493	a2
Utml6	1.536757	a2
Utml9	1.548945	a2
Utml1	1.578959	a2
Utml2	1.672006	a2
Utml8	1.867095	a2
Utml3	1.933839	a2
Utml5	2.300439	a3
Utml14	2.582683	a3
Utml15	2.832590	a3

Arquivo analisado:

F:\Dados Tese\estatísticas\Estatistica UT\Fitoquímica\utmz iso.dbf

Variável analisada: CONC

Opção de transformação: Variável sem transformação (Y)

TABELA DE ANÁLISE DE VARIÂNCIA

FV	GL	SQ	QM	Fc	Pr>Fc
TRAT	19	12.201107	0.642164	12.969	0.0000
erro	40	1.980667	0.049517		
Total corrigido	59	14.181773			

CV (%) = 19.74
Média geral: 1.1273333 Número de observações: 60

Teste Scott-Knott (1974) para a FV TRAT

NMS: 0,05

Média harmonica do número de repetições (r): 3
Erro padrão: 0,128473948937345

Tratamentos	Médias	Resultados do teste
Utmz18	0.476667	a1
Utmz15	0.516667	a1
Utmz17	0.596667	a1
Utmz16	0.653333	a1
Utmz13	0.760000	a1
Utmz20	0.773333	a1
Utmz12	0.783333	a1
Utmz2	0.960000	a2
Utmz10	1.036667	a2
Utmz11	1.060000	a2
Utmz8	1.123333	a2
Utmz9	1.150000	a2
Utmz7	1.206667	a2
Utmz5	1.260000	a2
Utmz4	1.340000	a3
Utmz14	1.416667	a3
Utmz6	1.676667	a4
Utmz1	1.850000	a4
Utmz19	1.913333	a4
Utmz3	1.993333	a4

Variável analisada: CONC

Opção de transformação: Raiz quadrada - SQRT (Y)

TABELA DE ANÁLISE DE VARIÂNCIA

FV	GL	SQ	QM	Fc	Pr>Fc
TRAT	19	2.789297	0.146805	11.138	0.0000
erro	40	0.527233	0.013181		
Total corrigido	59	3.316530			

CV (%) = 11.09
Média geral: 1.0354023 Número de observações: 60

Teste Scott-Knott (1974) para a FV TRAT

NMS: 0,05

Média harmonica do número de repetições (r): 3

Erro padrão: 0,0662843180082573

Tratamentos	Médias	Resultados do teste
Utmz15	0.675129	a1
Utmz18	0.685385	a1
Utmz17	0.770109	a1
Utmz16	0.808191	a2
Utmz13	0.870359	a2
Utmz20	0.878711	a2
Utmz12	0.882775	a2
Utmz2	0.977639	a2
Utmz10	1.014667	a3
Utmz11	1.029106	a3
Utmz8	1.057718	a3
Utmz9	1.071890	a3
Utmz7	1.095170	a3
Utmz5	1.120132	a3
Utmz4	1.147521	a3
Utmz14	1.183400	a3
Utmz6	1.291021	a4
Utmz1	1.355086	a4
Utmz19	1.382314	a4
Utmz3	1.411722	a4

Arquivo analisado:

F:\Dados Tese\estatísticas\Estatistica UT\Fitoquímica\utsa iso.dbf

Variável analisada: CONC

Opção de transformação: Variável sem transformação (Y)

TABELA DE ANÁLISE DE VARIÂNCIA

FV	GL	SQ	QM	Fc	Pr>Fc
TRAT	19	19.498832	1.026254	21.260	0.0000
erro	40	1.930867	0.048272		
Total corrigido	59	21.429698			

CV (%) = 15.15
Média geral: 1.4501667 Número de observações: 60

Teste Scott-Knott (1974) para a FV TRAT

NMS: 0,05

Média harmonica do número de repetições (r): 3
Erro padrão: 0,126848553620274

Tratamentos	Médias	Resultados do teste
Utsa17	0.646667	a1
Utsa2	0.740000	a1
Utsa15	0.756667	a1
Utsa13	0.946667	a1
Utsa5	1.046667	a2
Utsa11	1.060000	a2
Utsa3	1.083333	a2
Utsa12	1.190000	a2
Utsa10	1.216667	a2
Utsa16	1.263333	a2
Utsa9	1.296667	a2
Utsa8	1.493333	a2
Utsa19	1.626667	a3
Utsa4	1.686667	a3
Utsa6	1.706667	a3
Utsa14	1.766667	a3
Utsa18	1.866667	a3
Utsa7	2.436667	a4
Utsa1	2.580000	a4
Utsa20	2.593333	a4

Variável analisada: CONC

Opção de transformação: Raiz quadrada - SQRT (Y)

TABELA DE ANÁLISE DE VARIÂNCIA

FV	GL	SQ	QM	Fc	Pr>Fc
TRAT	19	3.595232	0.189223	8.318	0.0000
erro	40	0.909900	0.022748		
Total corrigido	59	4.505132			

CV (%) = 12.86
Média geral: 1.1726385 Número de observações: 60

Teste Scott-Knott (1974) para a FV TRAT

NMS: 0,05

Média harmonica do número de repetições (r): 3
Erro padrão: 0,0870775531719493

Tratamentos	Médias	Resultados do teste
Utsa15	0.709744	a1

Utsa17	0.803874	a1
Utsa2	0.859871	a1
Utsa13	0.972874	a2
Utsa5	1.020962	a2
Utsa11	1.029288	a2
Utsa3	1.040276	a2
Utsa12	1.087300	a2
Utsa10	1.102111	a2
Utsa16	1.123690	a2
Utsa9	1.136851	a2
Utsa8	1.220489	a3
Utsa19	1.274596	a3
Utsa4	1.297991	a3
Utsa6	1.306265	a3
Utsa14	1.329159	a3
Utsa18	1.365719	a3
Utsa7	1.560266	a4
Utsa1	1.604807	a4
Utsa20	1.606639	a4

Arquivo analisado:

F:\Dados Tese\estatísticas\Estatistica UT\Fitoquímica\utta iso.dbf

Variável analisada: CONC

Opção de transformação: Variável sem transformação (Y)

TABELA DE ANÁLISE DE VARIÂNCIA

FV	GL	SQ	QM	Fc	Pr>Fc
TRAT	19	203.525867	10.711888	1.229	0.2836
erro	40	348.555933	8.713898		
Total corrigido	59	552.081800			

CV (%) = 99.73
Média geral: 2.9600000 Número de observações: 60

Teste Scott-Knott (1974) para a FV TRAT

NMS: 0,05

Média harmonica do número de repetições (r): 3
Erro padrão: 1,70429832417267

Tratamentos	Médias	Resultados do teste
Utta12	0.356667	a1
Utta18	1.663333	a1

Utta10	1.796667 a1
Utta7	1.850000 a1
Utta2	1.980000 a1
Utta5	2.026667 a1
Utta16	2.070000 a1
Utta14	2.073333 a1
Utta15	2.276667 a1
Utta19	2.690000 a1
Utta9	3.146667 a1
Utta1	3.150000 a1
Utta11	3.156667 a1
Utta20	3.160000 a1
Utta4	3.236667 a1
Utta6	3.470000 a1
Utta8	3.573333 a1
Utta17	3.673333 a1
Utta3	3.760000 a1
Utta13	10.090000 a1

Variável analisada: CONC

Opção de transformação: Raiz quadrada - SQRT (Y)

TABELA DE ANÁLISE DE VARIÂNCIA

FV	GL	SQ	QM	Fc	Pr>Fc
TRAT	19	9.469656	0.498403	2.282	0.0140
erro	40	8.736630	0.218416		
Total corrigido	59	18.206286			

CV (%) = 28.67
Média geral: 1.6298963 Número de observações: 60

Teste Scott-Knott (1974) para a FV TRAT

NMS: 0,05

Média harmonica do número de repetições (r): 3
Erro padrão: 0,269824483631399

Tratamentos	Médias	Resultados do teste
Utta12	0.596583	a1
Utta18	1.284619	a1
Utta10	1.338555	a1
Utta7	1.358780	a1
Utta2	1.406303	a1
Utta5	1.418279	a1
Utta14	1.429330	a1
Utta16	1.431439	a1

Utta15	1.508725 a1
Utta19	1.631850 a1
Utta20	1.748220 a1
Utta9	1.773733 a1
Utta11	1.774530 a1
Utta1	1.774689 a1
Utta4	1.798742 a1
Utta6	1.858456 a1
Utta8	1.884196 a1
Utta17	1.915218 a1
Utta3	1.926339 a1
Utta13	2.739339 a1

Mitraphyline
Among population

Arquivo analisado:

C:\Users\ISABELA\Documents\Isabela\Isabela\Doutorado\Tese\Estatistica UT\pop2.dbf

Variável analisada: CONC

Opção de transformação: Variável sem transformação (Y)

TABELA DE ANÁLISE DE VARIÂNCIA

FV	GL	SQ	QM	Fc	Pr>Fc
TRAT	7	191.719529	27.388504	5.473	0.0024
erro	16	80.067067	5.004192		
Total corrigido	23	271.786596			

CV (%) = 39.07
Média geral: 5.7254167 Número de observações: 24

Teste Scott-Knott (1974) para a FV TRAT

NMS: 0,05

Média harmonica do número de repetições (r): 3
Erro padrão: 1,29153547720877

Tratamentos	Médias	Resultados do teste
Utaf	1.310000	a1
Utfj	3.266667	a1
Utml	4.726667	a1
Utsa	4.930000	a1
Utmc	5.646667	a1
Utmz	6.473333	a1

Utcs	8.280000	a2
Utta	11.170000	a2

Mitraphyline
Within population

Arquivo analisado:

C:\Users\bbertoni\Desktop\BIANCA\DOUTORADO\uncaria\tomentosa\quimica\utaf.dbf

Variável analisada: COM

Opção de transformação: Variável sem transformação (Y)

TABELA DE ANÁLISE DE VARIÂNCIA

FV	GL	SQ	QM	Fc	Pr>Fc
TRAT	19	164.120181	8.637904	90.221	0.0000
erro	39	3.733911	0.095741		
Total corrigido	58	167.854092			

CV (%) = 24.11
Média geral: 1.2833898 Número de observações: 59

Teste Scott-Knott (1974) para a FV TRAT

NMS: 0,05

Média harmonica do número de repetições (r): 2,92682926829268
Erro padrão: 0,180863513571175

Tratamentos	Médias	Resultados do teste
Utaf18	0.000000	a1
Utaf17	0.000000	a1
Utaf16	0.000000	a1
Utaf19	0.000000	a1
Utaf9	0.000000	a1
Utaf7	0.000000	a1
Utaf20	0.000000	a1
Utaf11	0.000000	a1
Utaf10	0.000000	a1
Utaf15	0.000000	a1
Utaf14	0.782333	a2
Utaf13	0.814000	a2

Utaf4	1.144000	a2
Utaf12	1.809000	a3
Utaf6	2.163333	a3
Utaf3	2.490333	a4
Utaf5	2.731333	a4
Utaf1	3.319667	a5
Utaf8	3.987667	a6
Utaf2	5.998333	a7

Variável analisada: COM

Opção de transformação: Raiz quadrada - SQRT (Y)

TABELA DE ANÁLISE DE VARIÂNCIA

FV	GL	SQ	QM	Fc	Pr>Fc
TRAT	19	40.460522	2.129501	304.508	0.0000
erro	39	0.272737	0.006993		
Total corrigido	58	40.733259			

CV (%) = 10.86
Média geral: 0.7700621 Número de observações: 59

Teste Scott-Knott (1974) para a FV TRAT

NMS: 0,05

Média harmonica do número de repetições (r): 2,92682926829268
Erro padrão: 0,0488810877473007

Tratamentos	Médias	Resultados do teste
Utaf18	0.000000	a1
Utaf17	0.000000	a1
Utaf16	0.000000	a1
Utaf19	0.000000	a1
Utaf9	0.000000	a1
Utaf7	0.000000	a1
Utaf20	0.000000	a1
Utaf11	0.000000	a1
Utaf10	0.000000	a1
Utaf15	0.000000	a1
Utaf14	0.884424	a2
Utaf13	0.899649	a2
Utaf4	1.069573	a3
Utaf12	1.344207	a4
Utaf6	1.470252	a4
Utaf3	1.568601	a5
Utaf5	1.649162	a5
Utaf1	1.818867	a6

Utaf8	1.995771	a7
Utaf2	2.444048	a8

Arquivo analisado:

C:\Users\bbertoni\Desktop\BIANCA\DOUTORADO\uncaria\tomentosa\quimica\utcs.dbf

Variável analisada: COM

Opção de transformação: Variável sem transformação (Y)

TABELA DE ANÁLISE DE VARIÂNCIA

FV	GL	SQ	QM	Fc	Pr>Fc
TRAT	19	2836.425050	149.285529	124.797	0.0000
erro	40	47.849119	1.196228		
Total corrigido	59	2884.274169			

CV (%) = 13.26
Média geral: 8.2509000 Número de observações: 60

Teste Scott-Knott (1974) para a FV TRAT

NMS: 0,05

Média harmonica do número de repetições (r): 3
Erro padrão: 0,631460736001148

Tratamentos	Médias	Resultados do teste
Utcs18	0.000000	a1
Utcs19	0.000000	a1
Utcs1	4.048000	a2
Utcs15	4.266667	a2
Utcs20	4.899000	a2
Utcs17	5.007333	a2
Utcs7	5.228333	a2
Utcs5	5.790000	a3
Utcs9	5.930000	a3
Utcs11	6.108333	a3
Utcs3	6.183000	a3
Utcs2	6.212000	a3
Utcs8	7.392333	a3
Utcs6	9.131667	a4
Utcs14	10.158667	a4
Utcs13	11.212000	a5
Utcs12	11.645000	a5
Utcs4	12.322000	a5
Utcs10	16.541000	a6

Utcs16	32.942667 a7

Variável analisada: COM

Opção de transformação: Raiz quadrada - SQRT (Y)

TABELA DE ANÁLISE DE VARIÂNCIA

FV	GL	SQ	QM	Fc	Pr>Fc
TRAT	19	87.708452	4.616234	125.404	0.0000
erro	40	1.472438	0.036811		
Total corrigido	59	89.180890			

CV (%) = 7.38
Média geral: 2.6008752 Número de observações: 60

Teste Scott-Knott (1974) para a FV TRAT

NMS: 0,05

Média harmonica do número de repetições (r): 3
Erro padrão: 0,110771445856066

Tratamentos	Médias	Resultados do teste
Utcs18	0.000000	a1
Utcs19	0.000000	a1
Utcs1	1.992228	a2
Utcs15	2.060530	a2
Utcs20	2.209404	a2
Utcs17	2.237584	a2
Utcs7	2.264898	a2
Utcs5	2.405554	a3
Utcs9	2.434739	a3
Utcs11	2.469600	a3
Utcs3	2.479717	a3
Utcs2	2.480822	a3
Utcs8	2.718617	a3
Utcs6	3.021305	a4
Utcs14	3.180237	a4
Utcs13	3.348381	a5
Utcs12	3.405954	a5
Utcs4	3.507299	a5
Utcs10	4.062239	a6
Utcs16	5.738397	a7

Arquivo analisado:

C:\Users\bbertoni\Desktop\BIANCA\DOUTORADO\uncaria\tomentosa\quimica\utfj.dbf

----

Variável analisada: COM

Opção de transformação: Variável sem transformação (Y)

TABELA DE ANÁLISE DE VARIÂNCIA

FV	GL	SQ	QM	Fc	Pr>Fc
TRAT	18	228.731140	12.707286	33.187	0.0000
erro	38	14.550300	0.382903		
Total corrigido	56	243.281440			
CV (%) =	20.70				
Média geral:	2.9894737	Número de observações:	57		

Teste Scott-Knott (1974) para a FV TRAT

NMS: 0,05

Média harmonica do número de repetições (r): 3
Erro padrão: 0,357259304324346

Tratamentos	Médias	Resultados do teste
Utfj4	0.639000	a1
Utfj2	0.904333	a1
Utfj5	1.093333	a1
Utfj9	1.100000	a1
Utfj11	1.245667	a1
Utfj1	1.347667	a1
Utfj8	1.511000	a1
Utfj12	2.252333	a2
Utfj15	2.287000	a2
Utfj3	2.584000	a2
Utfj7	2.603000	a2
Utfj17	2.811000	a2
Utfj19	2.879000	a2
Utfj20	3.937333	a3
Utfj6	4.051667	a3
Utfj14	5.449333	a4
Utfj13	6.227333	a4
Utfj18	6.676000	a5
Utfj10	7.201000	a5

Variável analisada: COM

Opção de transformação: Raiz quadrada - SQRT (Y)

TABELA DE ANÁLISE DE VARIÂNCIA

FV	GL	SQ	QM	Fc	Pr>Fc
TRAT	18	18.113451	1.006303	38.182	0.0000
erro	38	1.001506	0.026355		
Total corrigido	56	19.114957			

CV (%) = 9.96
Média geral: 1.6291481 Número de observações: 57

Teste Scott-Knott (1974) para a FV TRAT

NMS: 0,05

Média harmonica do número de repetições (r): 3
Erro padrão: 0,0937290830047894

Tratamentos	Médias	Resultados do teste
Utfj4	0.795181	a1
Utfj2	0.950597	a1
Utfj5	1.038124	a1
Utfj9	1.048595	a1
Utfj11	1.114662	a1
Utfj1	1.160199	a1
Utfj8	1.227262	a1
Utfj12	1.500562	a2
Utfj15	1.504618	a2
Utfj7	1.577577	a2
Utfj3	1.600173	a2
Utfj17	1.666176	a2
Utfj19	1.696532	a2
Utfj20	1.983763	a3
Utfj6	2.011469	a3
Utfj14	2.330767	a4
Utfj13	2.491944	a4
Utfj18	2.579286	a4
Utfj10	2.676330	a4

Arquivo analisado:

C:\Users\bbertoni\Desktop\BIANCA\DOUTORADO\uncaria\tomentosa\quimica\utmc.dbf

---

Variável analisada: COM

Opção de transformação: Variável sem transformação (Y)

TABELA DE ANÁLISE DE VARIÂNCIA

FV	GL	SQ	QM	Fc	Pr>Fc
TRAT	19	607.062664	31.950667	95.955	0.0000
erro	40	13.319023	0.332976		
Total corrigido	59	620.381686			

CV (%) = 9.92
Média geral: 5.8174000 Número de observações: 60

Teste Scott-Knott (1974) para a FV TRAT

NMS: 0,05

Média harmonica do número de repetições (r): 3
Erro padrão: 0,333154401975354

Tratamentos	Médias	Resultados do teste
Utmc19	0.000000	a1
Utmc20	0.000000	a1
Utmc18	0.567333	a1
Utmc17	0.632333	a1
Utmc13	4.165333	a2
Utmc14	4.370000	a2
Utmc15	5.356000	a3
Utmc10	5.622667	a3
Utmc1	5.812000	a3
Utmc3	6.612667	a4
Utmc16	6.959333	a5
Utmc2	7.091667	a5
Utmc4	7.381000	a5
Utmc6	7.955000	a6
Utmc12	8.039333	a6
Utmc7	8.044333	a6
Utmc5	8.245667	a6
Utmc11	9.411333	a7
Utmc9	9.736333	a7
Utmc8	10.345667	a7

Variável analisada: COM

Opção de transformação: Raiz quadrada - SQRT (Y)

TABELA DE ANÁLISE DE VARIÂNCIA

FV	GL	SQ	QM	Fc	Pr>Fc
TRAT	19	56.909249	2.995224	251.156	0.0000
erro	40	0.477029	0.011926		
Total corrigido	59	57.386279			

CV (%) = 4.95

Média geral: 2.2047589 Número de observações: 60

Teste Scott-Knott (1974) para a FV TRAT

NMS: 0,05

Média harmonica do número de repetições (r): 3

Erro padrão: 0,0630495337340046

Tratamentos	Médias	Resultados do teste
Utmc19	0.000000	a1
Utmc20	0.000000	a1
Utmc18	0.747172	a2
Utmc17	0.787136	a2
Utmc13	2.040526	a3
Utmc14	2.090433	a3
Utmc15	2.314300	a4
Utmc10	2.370818	a4
Utmc1	2.408515	a4
Utmc3	2.570227	a5
Utmc16	2.636923	a5
Utmc2	2.662381	a5
Utmc4	2.716445	a5
Utmc6	2.815753	a6
Utmc7	2.829613	a6
Utmc12	2.835249	a6
Utmc5	2.870927	a6
Utmc11	3.066271	a7
Utmc9	3.117559	a7
Utmc8	3.214929	a7

Arquivo analisado:

C:\Users\bbertoni\Desktop\BIANCA\DOUTORADO\uncaria\tomentosa\quimica\utml.dbf

Variável analisada: COM

Opção de transformação: Variável sem transformação (Y)

TABELA DE ANÁLISE DE VARIÂNCIA

FV	GL	SQ	QM	Fc	Pr>Fc
TRAT	19	1795.061275	94.476909	254.477	0.0000
erro	40	14.850374	0.371259		
Total corrigido	59	1809.911649			

CV (%) = 12.70
Média geral: 4.7991333 Número de observações: 60

Teste Scott-Knott (1974) para a FV TRAT

NMS: 0,05

Média harmonica do número de repetições (r): 3
Erro padrão: 0,351785611795972

Tratamentos	Médias	Resultados do teste
Utml17	0.000000	a1
Utml16	0.000000	a1
Utml15	0.000000	a1
Utml20	0.000000	a1
Utml19	0.000000	a1
Utml18	0.000000	a1
Utml12	0.000000	a1
Utml11	0.000000	a1
Utml14	0.000000	a1
Utml13	0.000000	a1
Utml10	5.176333	a2
Utml7	5.288000	a2
Utml4	5.390000	a2
Utml1	9.012333	a3
Utml9	9.212000	a3
Utml6	9.912667	a3
Utml8	10.623000	a4
Utml2	11.119333	a4
Utml3	12.222333	a5
Utml5	18.026667	a6

Variável analisada: COM

Opção de transformação: Raiz quadrada - SQRT (Y)

TABELA DE ANÁLISE DE VARIÂNCIA

FV	GL	SQ	QM	Fc	Pr>Fc
TRAT	19	148.971548	7.840608	835.988	0.0000
erro	40	0.375154	0.009379		
Total corrigido	59	149.346702			

CV (%) = 6.37
Média geral: 1.5198755 Número de observações: 60

Teste Scott-Knott (1974) para a FV TRAT

NMS: 0,05

Média harmonica do número de repetições (r): 3
Erro padrão: 0,0559131671471256

Tratamentos	Médias	Resultados do teste
Utml17	0.000000	a1
Utml16	0.000000	a1
Utml15	0.000000	a1
Utml20	0.000000	a1
Utml19	0.000000	a1
Utml18	0.000000	a1
Utml12	0.000000	a1
Utml11	0.000000	a1
Utml14	0.000000	a1
Utml13	0.000000	a1
Utml10	2.273526	a2
Utml7	2.298722	a2
Utml4	2.321441	a2
Utml1	2.994948	a3
Utml9	3.033870	a3
Utml6	3.147013	a3
Utml8	3.258540	a4
Utml2	3.333879	a4
Utml3	3.490067	a5
Utml5	4.245506	a6

Arquivo analisado:

C:\Users\bbertoni\Desktop\BIANCA\DOUTORADO\uncaria\tomentosa\quimica\utmz.dbf

Variável analisada: COM

Opção de transformação: Variável sem transformação (Y)

TABELA DE ANÁLISE DE VARIÂNCIA

FV	GL	SQ	QM	Fc	Pr>Fc
TRAT	19	342.704720	18.037091	12.006	0.0000
erro	40	60.093913	1.502348		
Total corrigido	59	402.798633			

CV (%) = 18.69
Média geral: 6.5586667 Número de observações: 60

Teste Scott-Knott (1974) para a FV TRAT

NMS: 0,05

Média harmonica do número de repetições (r): 3
Erro padrão: 0,707659954435116

Tratamentos	Médias	Resultados do teste
Utmz18	2.280333	a1
Utmz15	3.596333	a1
Utmz17	3.629000	a1
Utmz16	3.929667	a1
Utmz13	4.495000	a1
Utmz20	4.819333	a1
Utmz12	4.857333	a1
Utmz2	5.683000	a1
Utmz10	6.549000	a2
Utmz11	6.610667	a2
Utmz8	6.760667	a2
Utmz5	7.054667	a2
Utmz4	7.203667	a2
Utmz14	7.487667	a2
Utmz9	7.581000	a2
Utmz7	7.674000	a2
Utmz3	8.920667	a3
Utmz6	10.262333	a3
Utmz19	10.732667	a3
Utmz1	11.046333	a3

Variável analisada: COM

Opção de transformação: Raiz quadrada - SQRT (Y)

TABELA DE ANÁLISE DE VARIÂNCIA

FV	GL	SQ	QM	Fc	Pr>Fc
TRAT	19	13.948265	0.734119	11.843	0.0000
erro	40	2.479452	0.061986		
Total corrigido	59	16.427717			

CV (%) = 9.93
Média geral: 2.5069646 Número de observações: 60

Teste Scott-Knott (1974) para a FV TRAT

NMS: 0,05

Média harmonica do número de repetições (r): 3
Erro padrão: 0,143743162918088

Tratamentos	Médias	Resultados do teste
Utmz18	1.492891	a1
Utmz15	1.838568	a1
Utmz17	1.901629	a1
Utmz16	1.982333	a1
Utmz13	2.116238	a2
Utmz20	2.194658	a2
Utmz12	2.198534	a2
Utmz2	2.378998	a2
Utmz10	2.551712	a3
Utmz11	2.570151	a3
Utmz8	2.595374	a3
Utmz5	2.650924	a3
Utmz4	2.662874	a3
Utmz14	2.722748	a3
Utmz9	2.752605	a3
Utmz7	2.761429	a3
Utmz3	2.985144	a4
Utmz6	3.196594	a4
Utmz19	3.274502	a4
Utmz1	3.311384	a4

Arquivo analisado:

C:\Users\bbertoni\Desktop\BIANCA\DOUTORADO\uncaria\tomentosa\quimica\utsa.dbf

Variável analisada: CN

Opção de transformação: Variável sem transformação (Y)

TABELA DE ANÁLISE DE VARIÂNCIA

FV	GL	SQ	QM	Fc	Pr>Fc
TRAT	19	572.179860	30.114729	86.638	0.0000
erro	40	13.903627	0.347591		
Total corrigido	59	586.083487			

CV (%) = 12.15
Média geral: 4.8540167 Número de observações: 60

Teste Scott-Knott (1974) para a FV TRAT

NMS: 0,05

Média harmonica do número de repetições (r): 3
Erro padrão: 0,340387361039677

Tratamentos	Médias	Resultados do teste
Utsa8	0.000000	a1
Utsa2	0.000000	a1
Utsa1	0.000000	a1
Utsa17	1.782000	a2
Utsa5	2.534667	a3
Utsa13	3.229667	a3
Utsa15	3.383333	a3
Utsa11	3.682667	a3
Utsa3	3.883667	a3
Utsa12	4.577667	a4
Utsa16	4.683333	a4
Utsa10	5.234000	a4
Utsa9	6.126667	a5
Utsa4	6.876667	a5
Utsa19	7.110333	a5
Utsa6	7.607667	a6
Utsa18	7.746000	a6
Utsa14	7.923333	a6
Utsa20	9.544667	a7
Utsa7	11.154000	a8

Teste Scott-Knott (1974) para a FV TRAT

NMS: 0,05

Variável analisada: CN

Opção de transformação: Raiz quadrada - SQRT (Y)

TABELA DE ANÁLISE DE VARIÂNCIA

FV	GL	SQ	QM	Fc	Pr>Fc
TRAT	19	55.988279	2.946752	229.566	0.0000
erro	40	0.513448	0.012836		
Total corrigido	59	56.501727			

CV (%) = 5.73
Média geral: 1.9779589 Número de observações: 60

Teste Scott-Knott (1974) para a FV TRAT

NMS: 0,05

Média harmonica do número de repetições (r): 3
Erro padrão: 0,0654120078441096

Tratamentos	Médias	Resultados do teste
Utsa8	0.000000	a1
Utsa2	0.000000	a1
Utsa1	0.000000	a1
Utsa17	1.333966	a2
Utsa5	1.589915	a3
Utsa13	1.796208	a4
Utsa15	1.837209	a4
Utsa11	1.918685	a4
Utsa3	1.969710	a4
Utsa12	2.133158	a5
Utsa16	2.163435	a5
Utsa10	2.286020	a5
Utsa9	2.470429	a6
Utsa4	2.620848	a6
Utsa19	2.665113	a6
Utsa6	2.758014	a7
Utsa18	2.782761	a7
Utsa14	2.814792	a7
Utsa20	3.080997	a8
Utsa7	3.337917	a9

Teste Scott-Knott (1974) para a FV TRAT

NMS: 0,05

Arquivo analisado:

C:\Users\bbertoni\Desktop\BIANCA\DOUTORADO\uncaria\tomentosa\quimica\utta.dbf

---

Variável analisada: COM

Opção de transformação: Variável sem transformação (Y)

TABELA DE ANÁLISE DE VARIÂNCIA

FV	GL	SQ	QM	Fc	Pr>Fc
TRAT	19	1845.120156	97.111587	34.881	0.0000
erro	40	111.362969	2.784074		
Total corrigido	59	1956.483125			

CV (%) = 17.34
Média geral: 9.6203333 Número de observações: 60

Teste Scott-Knott (1974) para a FV TRAT

NMS: 0,05

Média harmonica do número de repetições (r): 3
Erro padrão: 0,963340409431912

Tratamentos	Médias	Resultados do teste
Utta4	0.000000	a1
Utta3	0.000000	a1
Utta2	0.000000	a1
Utta12	1.378667	a1
Utta10	6.907333	a2
Utta7	6.951000	a2
Utta18	9.164667	a3
Utta5	9.354667	a3
Utta16	9.627000	a3
Utta15	9.786000	a3
Utta14	9.806667	a3
Utta13	11.748000	a4
Utta1	11.878667	a4
Utta19	12.312667	a4
Utta11	12.865333	a4
Utta9	14.380667	a5
Utta8	14.974667	a5
Utta6	15.608000	a5
Utta17	16.217667	a5
Utta20	19.445000	a6

Variável analisada: COM

Opção de transformação: Raiz quadrada - SQRT (Y)

TABELA DE ANÁLISE DE VARIÂNCIA

FV	GL	SQ	QM	Fc	Pr>Fc
TRAT	19	108.008875	5.684678	93.751	0.0000
erro	40	2.425446	0.060636		
Total corrigido	59	110.434321			

CV (%) = 8.83
Média geral: 2.7892224 Número de observações: 60

Teste Scott-Knott (1974) para a FV TRAT

NMS: 0,05

Média harmonica do número de repetições (r): 3
Erro padrão: 0,142169087673223

Tratamentos	Médias	Resultados do teste
Utta4	0.000000	a1
Utta3	0.000000	a1
Utta2	0.000000	a1
Utta12	1.171524	a2
Utta10	2.624236	a3
Utta7	2.631575	a3
Utta18	3.016773	a4
Utta5	3.048984	a4
Utta16	3.091788	a4
Utta14	3.111578	a4
Utta15	3.127617	a4
Utta13	3.417849	a5
Utta1	3.446243	a5
Utta19	3.491278	a5
Utta11	3.586030	a5
Utta9	3.792019	a6
Utta8	3.856255	a6
Utta6	3.938335	a6
Utta17	4.024154	a6
Utta20	4.408210	a7
