# Supplementary material for: Genetic and chemical diversity of Uncaria tomentosa (Willd. ex. Schult.) DC. in the Brazilian Amazon
Source: PLoS One. 2017 May 5;12(5):e0177103. doi: 10.1371/journal.pone.0177103 (PMC5419575; doi:10.1371/journal.pone.0177103)
Supplement: S1 File — (PDF) [file pone.0177103.s003.pdf]

UNIVERSIDADE FEDERAL DO PARANÁ

GUSTAVO BERTOL

DESENVOLVIMENTO E VALIDAÇÃO DE MÉTODOS ANALÍTICOS PARA CONTROLE DE QUALIDADE DE MATÉRIAS-PRIMAS E PRODUTO CONTENDO U*ncaria tomentosa* (Willd.) DC. - Rubiaceae

CURITIBA
2010

GUSTAVO BERTOL

DESENVOLVIMENTO E VALIDAÇÃO DE MÉTODOS ANALÍTICOS PARA CONTROLE DE QUALIDADE DE MATÉRIAS-PRIMAS E PRODUTO CONTENDO *Uncaria tomentosa* (Willd.) DC. - Rubiaceae

Dissertação apresentada ao Programa de Pós-graduação em Ciências Farmacêuticas, Área de Concentração Insumos, Medicamentos e Correlatos, Departamento de Farmácia, Setor de Ciências da Saúde, Universidade Federal do Paraná, como parte das exigências para a obtenção do título de Mestre em Ciências Farmacêuticas.

Orientador: Prof. Dr. Brás Heleno de Oliveira

CURITIBA

2010

Para minha esposa, pelo seu amor incondicional.

Aos meus pais, por me propiciarem vida e amor.

AGRADECIMENTOS

À minha esposa, Merilin pela compreensão nos momentos de solidão, pelo carinho quando eu estava cansado, pela alegria quando eu desanimava.

Aos meus pais, Vera e Oromar, meu esteio, orientação e segurança para a vida.

Ao Eduardo e Henrique, meus queridos irmãos, pela amizade, carinho e respeito que nutrimos em nossa família.

A Deli e Lauri, meus sogros e grandes incentivadores.

Ao professor Dr. Brás Heleno de Oliveira pela orientação segura durante o andamento do trabalho.

Aos amigos de Desenvolvimento Analítico do Herbarium Laboratório Botânico: Cláudio S. de Souza Filho, Cristiane Mara Kopke, Luzia Franco, Melissa Ariadne e Marina Maciel Braz, pela compreensão e auxílio quando as tarefas se acumulavam.

Aos demais amigos do Herbarium pelo apoio e paciência durante a execução deste trabalho, em especial a Laerte Dall'Agnol, aos colegas do Controle de Qualidade físico-químico e Desenvolvimento de produtos.

À professora Dra. Tomoe Nakashima por ter permitido meu ingresso no laboratório de fitoquímica da UFPR e iniciação dos meus estudos com produtos naturais.

Aos colegas de mestrado Karina Bora e João Gasparetto pelo auxílio na realização das análises e amizade.

Ao Daniel Altino de Jesus do LACEN-PR por sua disposição e execução das análises de espectrometria de massas.

Ao Valdomiro Catani e João Alencar de Sousa, pesquisadores da EMBRAPA, pelas valiosas informações e fotos disponibilizadas sobre e planta objeto do estudo.

Enfim, obrigado Deus pela atual existência, pelas oportunidades, pelas dificuldades e principalmente pelo amor.

“O sacrifício pessoal é o preço do acesso
aos caminhos da Grande Luz”.
Bezerra de Menezes

RESUMO

Cascas do caule e raiz de *Uncaria tomentosa,* popularmente conhecida como unha-de-gato, são utilizadas no tratamento de diversos casos de inflamações, câncer e processos virais. Planta nativa da América do Sul possui várias de suas propriedades farmacológicas associadas aos alcalóides oxindólicos (AO). Os AO presentes na espécie são classificados em pentacíclicos (AOP) e tetracíclicos (AOT). Os AOT (rincofilina e isorincofilina) possuem acentuada ação no sistema nervoso central e são considerados antagonistas dos AOP (especiofilina, uncarina F, mitrafilina, isomitrafilina, pteropodina e isopteropodina), portanto devem ter seu teor controlado. O presente trabalho apresenta o desenvolvimento e validação de métodos para o controle de qualidade de *U. tomentosa* e seus derivados farmacêuticos tintura e gel, por CLAE. Entre os diferentes alcalóides constituintes da espécie, o alcalóide mitrafilina foi indicado após avaliações como o mais adequado para padronização do material utilizado. O método CLAE para casca e tintura e o preparo da amostra casca foram otimizados com planejamento fatorial de experimentos. A análise por espectrometria de massas de amostras da planta indicou presença de AOP ainda não descritos na espécie, provavelmente uncarinas A e B, e o oxindólico rauniticina *allo* A ou B. Para análise do gel foi desenvolvido método de preparo da amostra pela aplicação da técnica de dispersão de matriz em fase sólida (MSPD) e quantificação por CLAE. A seletividade / especificidade dos métodos foram avaliadas através da determinação da pureza dos picos, comparação dos perfis espectrais entre padrão e amostra, análise do placebo do gel, verificação do paralelismo das curvas de padrão e amostra adicionada de padrão para cascas e tintura e análise por espectrometria de massas para cada um dos alcalóides no método para casca. Linearidade com coeficiente de correlação (r) igual a 0,9998 e 0,9997 foi obtida com o padrão analítico para os métodos da casca e gel respectivamente. Limites de 0,80 e 1,41 $\mu g.mL^{-1}$ (detecção) e 2,40 e 4,27 $\mu g.mL^{-1}$ (quantificação) foram obtidos para método CLAE da casca e gel respectivamente. Precisão com desvio padrão relativo (DRP) inferior a 2,30%, recuperação superior a 98,37% para exatidão e r de 0,9979 foram obtidos para o método da casca. Para o método de análise da tintura valores de precisão com DPR inferior a 0,93%, linearidade com r de 0,9989 e recuperação superior a 98,37% para exatidão foram encontrados. Para o gel a validação apresentou valores de DPR inferior a 1,96% nos ensaios de precisão, r de 0,9927 para linearidade e mínimo de 98,78% de recuperação para a exatidão. Finalmente o trabalho demonstrou que os métodos permitem análises confiáveis da droga *U. tomentosa*, sua tintura e gel contendo como ativo a tintura da planta.

Palavras chave: *Uncaria tomentosa* DC. Mitrafilina. Validação analítica. CLAE. Alcalóides oxindólicos.

ABSTRACT

Stem and root barks of *Uncaria tomentosa*, popularly known as cat's claw, are used in the treatment of several cases of inflammations, cancer and viral processes. The plant is native from South America and possesses several of its pharmacological properties associated with the oxindole alkaloids (OA). The OA found in the specie are classified in pentacyclic (POA) and tetracyclic (TOA). TOA (rhynchophylline and isorhynchophylline) possess accentuated action in the central nervous system and are considered antagonists of POA (speciophylline, uncarine F, mitraphylline, isomitraphylline, pteropodine and isopteropodine), therefore their amount should be controlled. This work presents the development and validation of methods for quality control of *U. tomentosa* plant material and a gel formulation containing its tincture, by HPLC. Among the different alkaloids present in the specie, the alkaloid mitraphylline was indicated as the most suitable for standardization of the used samples. Both the bark sample preparation and the HPLC method for bark and tincture preparation were optimized with statistical experimental design. The mass spectrometry analysis of species sample indicated the presence of POA still not described in the specie, probably uncarines A and B, and the oxindolic rauniticine *allo* A or B. Sample preparation method for gel analysis was developed with the application of the technique of matrix solid-phase dispersion (MSPD) and HPLC quantification. The selectivity and specificity of the methods were evaluated through the determination of the peak purity, comparison of the spectral profiles between standard and sample, analysis of the placebo of the gel, verification of the parallelism of analytical curves and sample spiked with standard for bark and tincture and mass spectrometry analysis for each one of the alkaloids in the bark method. Correlation coefficients (r) equal to 0,9998 and 0,9997 were obtained with the analytical standard in the linearity test respectively for the methods of bark and tincture. Limits of 0,80 and 1,41 $\mu g.mL^{-1}$ (detection) and 2,40 and 4,27 $\mu g.mL^{-1}$ (quantification) were obtained respectively for HPLC method of bark and gel. Precision with relative standard deviation (RSD) lower than 2,30%, recovery higher than 98,37% for accuracy and r of 0,9979 were obtained for bark method. For tincture method of analysis values of precision with RSD lower than 0,93%, linearity with r value of 0,9989 and recovery higher than 98,37% for accuracy were found. For gel validation RSD values lower than 1,96% for precision tests, r value of 0,9927 for linearity and recovery higher than 98,78% for accuracy were found. Finally the present work demonstrated that the methods allowed reliable analyses of the drug *U. tomentosa,* its tincture and gel containing the plant tincture as active principle.

Key words: *Uncaria tomentosa* DC. Mitraphylline. Analytical validation. HPLC. Oxindole alkaloids.

LISTA DE FIGURAS

FIGURA 1 – ASPECTO DOS ESPINHOS DE *U. tomentosa* DC., RUBIACEAE.......21
FIGURA 2 – ASPECTO DO CAULE DE *U. tomentosa* DC., RUBIACEAE. 21
FIGURA 3 – FLORES DE *U. tomentosa* DC., RUBIACEAE.....................................22
FIGURA 4 – ASPECTO DA TREPADEIRA *U. tomentosa* DC., RUBIACEAE. 22
FIGURA 5 – ROTA BIOSSINTÉTICA DO l-TRIPTOFANO 29
FIGURA 6 – INTERCONVERSÃO DE ALCALÓIDE OXINDOLICO DO TIPO *PSEUDO* PARA O TIPO NORMAL..32
FIGURA 7 – MÉTODO DE DISPERSÃO DE MATRIZ EM FASE SÓLIDA 36
FIGURA 8 – DESIGN COMPOSTO CENTRAL PARA TRÊS FATORES AVALIADOS 39
FIGURA 9 – DETERMINAÇÃO DA EXATIDÃO PELA ADIÇÃO DE PADRÃO À AMOSTRA 46
FIGURA 10 – FLUXOGRAMA DAS ETAPAS SEGUIDAS NA PESQUISA...............50
FIGURA 11 – FLUXOGRAMA DO PREPARO DA AMOSTRA GEL 60
FIGURA 12 – CROMATOGRAMA DO ALCALÓIDE ISOLADO 68
FIGURA 13 – ESTRUTURA DA MITRAFILINA..69
FIGURA 14 – ESPECTRO DE RMN - ^{1}H (200 MHz) EM $CDCl_3$ DA MITRAFILINA ISOLADA 70
FIGURA 15 – ESPECTRO DE RMN – ^{13}C (200 MHz) EM $CDCl_3$ DA MITRAFILINA ISOLADA 71
FIGURA 16 – ESPECTRO DEPT (200 MHz) EM $CDCl_3$ DA MITRAFILINA ISOLADA 72
FIGURA 17 – ESPECTRO DE MASSAS DA MITRAFILINA ISOLADA - $[M+H]^+$ 73
FIGURA 18 – ESPECTRO DE ABSORÇÃO DA MITRAFILINA NO ULTRAVIOLETA (190 – 330nm)..74
FIGURA 19 – ESPECTRO EM 3D DA MITRAFILINA ISOLADA OBTIDO EM CLAE-DAD 74
FIGURA 20 – ANÁLISE DE DSC DA MITRAFILINA ISOLADA................................76
FIGURA 21 – EFEITOS PADRONIZADOS – PAR UNCARINA F - MITRAFILINA ...78
FIGURA 22 – EFEITOS PRINCIPAIS – PAR UNCARINA F – MITRAFILINA...........79
FIGURA 23 – SUPERFÍCIE DE RESPOSTA DAS CONDIÇÕES CLAE PARA CASCAS E TINTURA 80
FIGURA 24 – PERFIL CROMATOGRÁFICO DAS CONDIÇÕES OTIMIZADAS PARA CASCAS E TINTURA – PLANTA BRASILEIRA 81
FIGURA 25 – PERFIL CROMATOGRÁFICO DAS CONDIÇÕES OTIMIZADAS PARA CASCAS E TINTURA – PLANTA PERUANA.....................................81
FIGURA 26 – EM – MS1 DA PLANTA BRASILEIRA 83
FIGURA 27 – EM – MS1 DA PLANTA PERUANA...84
FIGURA 28 – MS2 DOS ALCALÓIDES OXINDÓLICOS 86
FIGURA 29 – PROPOSTA DE FRAGMENTAÇÃO PARA AOP................................90
FIGURA 30 – PROPOSTA DE FRAGMENTAÇÃO PARA AOT................................91
FIGURA 31 – ESTABILIDADE DAS SOLUÇÕES PADRÃO.....................................94
FIGURA 32 – PERFIL DE ELUIÇÃO DO PROPIL PARABENO NO METODO CLAE PARA CASCAS E TINTURA...96
FIGURA 33 – PERFIL CROMATOGRÁFICO DOS AO DO GEL APÓS OTIMIZAÇÕES 97

FIGURA 34 – GRÁFICO DE PARETO PARA AS CONDIÇÕES EXTRATIVAS DAS CASCAS 99
FIGURA 35 – EFEITOS PRINCIPAIS PARA AS CONDIÇÕES EXTRATIVAS DAS CASCAS 100
FIGURA 36 – SUPERFÍCIE DE RESPOSTA PARA OTMIZAÇÃO DA EXTRAÇÃO DAS CASCAS 101
FIGURA 37 – PERFIL CROMATOGRÁFICO DA TINTURA DE *U. tomentosa* 102
FIGURA 38 – DEMONSTRAÇÃO DAS ETAPAS DO PROCESSO DE PREPARO DA AMOSTRA GEL 105
FIGURA 39 – PERFIS ESPECTRAIS DOS PICOS DE AOP ANALISADOS 107
FIGURA 40 – LINEARIDADE COM PADRÃO MITRAFILINA, MÉTODO CLAE CASCAS E TINTURA 109
FIGURA 41 – CURVA ANALÍTICA PARA CÁLCULO DO LD PARA CASCAS E TINTURA 110
FIGURA 42 – LINEARIDADE – VALIDAÇÃO DAS CASCAS DE *U. tomentosa* 113
FIGURA 43 – DISPERSÃO DOS RESÍDUOS – VALIDAÇÃO DAS CASCAS DE *U. tomentosa* 114
FIGURA 44 – AVALIAÇÃO DO EFEITO MATRIZ – VALIDAÇÃO DAS CASCAS DE *U. tomentosa* 116
FIGURA 45 – LINEARIDADE – VALIDAÇÃO DA TINTURA 119
FIGURA 46 – DISPERSÃO DOS RESÍDUOS – VALIDAÇÃO DA TINTURA 120
FIGURA 47 – AVALIAÇÃO DO EFEITO MATRIZ – VALIDAÇÃO DA TINTURA 122
FIGURA 48 – SOBREPOSIÇÃO DOS CROMATOGRAMAS DO PLACEBO E AMOSTRA GEL 124
FIGURA 49 – FAIXA LINEAR DE TRABALHO OBTIDA PARA O MÉTODO GEL ..124
FIGURA 50 – LINEARIDADE – VALIDAÇÃO DO GEL 125
FIGURA 51 – DISPERSÃO DOS RESÍDUOS – VALIDAÇÃO DO GEL 125
FIGURA 52 – CURVA ANALÍTICA PARA CÁLCULO DO LD DO GEL 127

LISTA DE QUADROS

QUADRO 1 – ENQUADRAMENTO TAXONÔMICO SEGUNDO APG II (2003). 20
QUADRO 2 – CORRELAÇÃO ENTRE OS PRINCIPAIS METABÓLITOS DE *U. tomentosa* E SUAS PROPRIEDADES FARMACOLÓGICAS. 24
QUADRO 3 – PRINCIPAIS ALCALOIDES ENCONTRADOS EM *U. tomentosa*.......25
QUADRO 4 – ISÔMEROS POSSÍVEIS PARA ESTRUTURA GERAL DOS AOP 33
QUADRO 5 – CATEGORIAS DOS TESTES SUBMETIDOS À VALIDAÇÃO 40
QUADRO 6 – ENSAIOS NECESSÁRIOS PARA VALIDAÇÃO SEGUNDO CATEGORIA...40
QUADRO 7 – MODIFICAÇÕES POSSÍVEIS PARA AVALIAÇÃO DA ROBUSTEZ..48
QUADRO 8 – PARÂMETROS DE CONFORMIDADE DO SISTEMA PARA CLAE ..49
QUADRO 9 – COLUNAS TESTADAS NA VALIDAÇÃO DA ROBUSTEZ DOS MÉTODOS...65

LISTA DE TABELAS

TABELA 1 – LIMITES DE ACEITAÇÃO PARA AVALIAÇÃO DA EXATIDÃO 47
TABELA 2 – CONDIÇÕES UTILIZADAS NO ESPECTRÔMETRO DE MASSAS.....54
TABELA 3 – GRADIENTE UTILIZADO NAS ANÁLISES POR CLAE DAS CASCAS E TINTURA 56
TABELA 4 – GRADIENTE UTILIZADO NAS ANÁLISES POR CLAE DO GEL.........58
TABELA 5 – RESULTADOS OBTIDOS PARA RMN-^{1}H DA MITRAFILINA ISOLADA 69
TABELA 6 – DADOS OBTIDOS PARA RMN-^{13}C DA MITRAFILINA ISOLADA........70
TABELA 7 – CONDIÇÕES CROMATOGRÁFICAS PROPOSTAS POR GANZERA, (2004) 77
TABELA 8 – ETAPA DE TRIAGEM – PLANEJAMENTO FATORIAL PARA CLAE DAS CASCAS E TINTURA...77
TABELA 9 – ETAPA DE OTIMIZAÇÃO – PLANEJAMENTO FATORIAL CLAE DAS CASCAS E TINTURA 80
TABELA 10 – ÍONS MOLECULARES OBTIDOS EM CLAE-MS PARA A PLANTA BRASILEIRA E PERUANA 85
TABELA 11 – IONS PRODUTO DOS PRINCIPAIS ALCALÓIDES DE *U. tomentosa* 88
TABELA 12 – COMPARAÇÃO DAS CURVAS ANALÍTICAS AVALIADAS...............92
TABELA 13 – COMPARAÇÃO DOS DIFERENTES TEORES ALCALÓIDICOS OBTIDOS FRENTE ÀS DIFERENTES CURVAS ANALITICAS TESTADAS...92
TABELA 14 – ETAPA DE TRIAGEM – PLANEJAMENTO FATORIAL PARA EXTRAÇÃO DAS CASCAS 98
TABELA 15 – ETAPA DE OTIMIZAÇÃO – PLANEJAMENTO FATORIAL PARA EXTRAÇÃO DAS CASCAS 101
TABELA 16 – PUREZA DE PICO DOS ALCALÓIDES PARA AVALIAÇÃO DA SELETIVIDADE 108
TABELA 17 – AVALIAÇÃO DO LQ PARA CASCAS E TINTURA..........................111

TABELA 18 – RESULTADOS OBTIDOS COM AS DIFERENTES COLUNAS TESTADAS NA AVALIAÇÃO DA ROBUSTEZ PARA CLAE DAS CASCAS E TINTURA 112
TABELA 19 – REPETIBILIDADE – VALIDAÇÃO DAS CASCAS DE *U. tomentosa*114
TABELA 20 – PRECISÃO INTERMEDIÁRIA – VALIDAÇÃO DAS CASCAS DE *U. tomentosa* 115
TABELA 21 – DADOS PARA O TESTE DA ADIÇÃO DE PADRÃO À AMOSTRA CASCAS DE *U. tomentosa* 116
TABELA 22 – EXATIDÃO – VALORES DE RECUPERAÇÃO PARA OS AO DAS CASCAS DE *U. tomentosa* 117
TABELA 23 – REPETIBILIDADE – VALIDAÇÃO DA TINTURA..............................120
TABELA 24 – PRECISÃO INTERMEDIÁRIA – VALIDAÇÃO DA TINTURA 121
TABELA 25 – DADOS PARA O TESTE DA ADIÇÃO DE PADRÃO À AMOSTRA TINTURA 122
TABELA 26 – EXATIDÃO – VALORES DE RECUPERAÇÃO PARA OS AO DA TINTURA 123
TABELA 27 – REPETIBILIDADE – VALIDAÇÃO DO GEL 126
TABELA 28 – PRECISÃO INTERMEDIÁRIA – VALIDAÇÃO DO GEL 127
TABELA 29 – EXATIDÃO – VALORES DE RECUPERAÇÃO PARA OS AO DO GEL 128
TABELA 30 – RESULTADOS OBTIDOS COM AS DIFERENTES COLUNAS TESTADAS NA AVALIAÇÃO DA ROBUSTEZ PARA CLAE DO GEL 129

LISTA DE ABREVIATURAS, SIGLAS E SÍMBOLOS

ANOVA	- Análise de variância
ANVISA	- Agência Nacional de Vigilância Sanitária
AO	- Alcalóides oxindólicos
AOP	- Alcalóides oxindólicos pentacíclicos
AOT	- Alcalóides oxindólicos tetracíclicos
^{o}C	- Graus Celsius
CLAE	- Cromatografia líquida de alta eficiência
DAD	- Detector de arranjo de diodos
DEPT	- Distortion enhancement by polarization transfer
DP	- Desvio padrão
DPR	- Desvio padrão relativo
DSC	- Differential scanning calorimetry (calorimetria exploratória diferencial)
EM	- Espectrometria de massas
ICH	- International Conference of Harmonization
INMETRO	- Instituto Nacional de Metrologia, Normalização e Qualidade Industrial
IUPAC	- International Union of Pure and Applied Chemistry
LD	- Limite de Detecção
LQ	- Limite de Quantificação
mAU	- Milli absorbance units (mili unidades de absorbância)
MeCN	- Acetonitrila
min	- Minutos
MSPD	- Matrix solid phase dispersion (dispersão de matriz em fase sólida)
MtBE	- Éter metil *tert*-butílico
m/z	- Relação massa-carga
OALB	- Óxido de alumínio básico
OALN	- Óxido de alumínio neutro
ppm	- Partes por milhão
r	- Coeficiente de correlação de Pearson
RMN- ^{13}C	- Ressonância Magnética Nuclear de carbono 13
RMN- ^{1}H	- Ressonância Magnética Nuclear de hidrogênio
rpm	- Rotações por minuto

s	- Segundos
TMS	- Tetrametilsilano
USP	- United States Pharmacopeia

SUMÁRIO

1 INTRODUÇÃO ... 17
2 OBJETIVOS ... 19
2.1 OBJETIVO GERAL ... 19
2.2 OBJETIVOS ESPECÍFICOS ... 19
3 REVISÃO DA LITERATURA ... 20
3.1 DESCRIÇÃO DA ESPÉCIE ... 20
3.1.1 Propriedades farmacológicas e constituintes químicos ... 23
3.2 ALCALÓIDES OXINDÓLICOS ... 27
3.3 DISPERSÃO DE MATRIZ EM FASE SÓLIDA ... 35
3.4 PLANEJAMENTO FATORIAL DE EXPERIMENTOS ... 37
3.5 VALIDAÇÃO DE MÉTODOS ANALÍTICOS ... 39
3.5.1 Seletividade / Especificidade ... 41
3.5.2 Linearidade e intervalo ... 42
3.5.3 Precisão ... 42
3.5.3.1 Repetitividade ou repetibilidade ... 43
3.5.3.2 Precisão intermediária ... 43
3.5.3.3 Reprodutibilidade ... 43
3.5.4 Limite de Detecção e Limite de Quantificação ... 44
3.5.5 Exatidão ... 45
3.5.6 Robustez ... 47
3.5.7 Estabilidade das soluções ... 48
3.5.8 Conformidade do Sistema ... 49
4 MATERIAIS E MÉTODOS ... 50
4.1 DESENHO EXPERIMENTAL ... 50
4.2 REAGENTES, SOLVENTES E SOLUÇÕES ... 51
4.3 PADRÕES ANALÍTICOS ... 51
4.4 EQUIPAMENTOS ... 52
4.5 AMOSTRAS ... 52
4.6 ISOLAMENTO DO PADRÃO ANALÍTICO DE ALCALÓIDE OXINDÓLICO ... 53
4.6.1 Identificação e confirmação da pureza do padrão isolado ... 54
4.7 DESENVOLVIMENTO DO TAMPÃO ORGÂNICO E OTIMIZAÇÃO DOS MÉTODOS EM CLAE ... 55
4.7.1 Desenvolvimento do Tampão orgânico e Otimização do método em CLAE para cascas e tintura ... 55
4.7.2 Verificação da capacidade de distinção entre AOT e AOP ... 56
4.7.3 Escolha do padrão externo para quantificação dos AO ... 56
4.7.4 Estabilidade das soluções padrão ... 57
4.7.5 Otimização do método CLAE para análise do gel ... 57
4.7.6 Avaliação da conformidade do sistema ... 58
4.8 DESENVOLVIMENTO E OTIMIZAÇÃO DOS MÉTODOS DE PREPARO DAS AMOSTRAS CASCAS, TINTURA E GEL ... 58
4.8.1 Otimização do preparo da amostra cascas de *U. tomentosa* ... 58
4.8.2 Otimização do preparo da amostra tintura de *U. tomentosa* ... 59
4.8.3 Otimização do preparo da amostra gel ... 59
4.9 VALIDAÇÃO DOS METODOS ANALÍTICOS ... 60
4.9.1 Seletividade / especificidade ... 60
4.9.1.1 Método da adição de padrão ... 60

4.9.1.2 Comparação dos perfis espectrais....61
4.9.1.3 Avaliação da pureza de pico....61
4.9.1.4 Monitoramento dos íons produtos originários de $[M+H]^+$ = 369 e 385 Da....61
4.9.1.5 Análise do placebo....61
4.9.2 Linearidade e intervalo....62
4.9.3 Precisão....62
4.9.3.1 Repetitividade ou repetibilidade....62
4.9.3.2 Precisão intermediária....63
4.9.4 Limite de detecção....63
4.9.5 Limite de quantificação....63
4.9.6 Exatidão....64
4.9.7 Robustez....64
4.10 CÁLCULO DO TEOR DE ALCALÓIDES NAS AMOSTRAS....65
4.10.1 Amostra cascas....66
4.10.2 Amostra tintura....66
4.10.3 Amostra gel....66
5 RESULTADOS E DISCUSSÃO....67
5.1 ISOLAMENTO, CARACTERIZAÇÃO E CONFIRMAÇÃO DA PUREZA DO PADRÃO ANALÍTICO....67
5.1.1 RMN de ^1H e ^{13}C....68
5.1.2 Espectrometria de massas....72
5.1.3 Perfil espectral em UV....73
5.1.4 Análise da Pureza em CLAE-DAD....74
5.1.5 Confirmação da pureza por DSC....75
5.2 DESENVOLVIMENTO DO TAMPÃO ORGÂNICO E OTIMIZAÇÃO DOS MÉTODOS EM CLAE....76
5.2.1 Desenvolvimento do Tampão orgânico e Otimização do método em CLAE para pó e tintura....76
5.2.2 Verificação da capacidade de distinção entre AOT e AOP....82
5.2.3 Escolha do padrão externo....91
5.2.4 Estabilidade das soluções padrão....93
5.2.5 Conformidade do sistema método CLAE para cascas e tintura....94
5.2.6 Otimização do método CLAE análise do gel....95
5.2.7 Conformidade do sistema método CLAE para gel....97
5.3 MÉTODOS DE PREPARO DAS AMOSTRAS....98
5.3.1 Preparo da amostra cascas....98
5.3.2 Preparo da amostra tintura....102
5.3.3 Preparo da amostra gel....103
5.4 VALIDAÇÃO DOS METODOS ANALÍTICOS....106
5.4.1 Comparação dos perfis espectrais....106
5.4.2 Avaliação da pureza de pico....108
5.4.3 Condições cromatográficas das cascas e tintura....109
5.4.3.1 Linearidade com padrão....109
5.4.3.2 Limite de detecção....110
5.4.3.3 Limite de quantificação....110
5.4.3.4 Robustez das condições cromatográficas CLAE para cascas e tintura....111
5.4.4 Validação do método para cascas de *U. tomentosa*....112
5.4.4.1 Seletividade / especificidade....112
5.4.4.2 Linearidade e intervalo....113
5.4.4.3 Repetibilidade....114

5.4.4.4 Precisão intermediária 115
5.4.4.5 Exatidão 116
5.4.4.6 Robustez 118
5.4.5 Validação do método para tintura 118
5.4.5.1 Seletividade / especificidade 118
5.4.5.2 Linearidade e intervalo 119
5.4.5.3 Repetibilidade 120
5.4.5.4 Precisão intermediária 121
5.4.5.5 Exatidão 121
5.4.6 Validação do método para o gel 123
5.4.6.1 Seletividade / especificidade 123
5.4.6.2 Linearidade e intervalo 124
5.4.6.3 Repetibilidade 126
5.4.6.4 Precisão intermediária 126
5.4.6.5 Limite de detecção 127
5.4.6.6 Limite de quantificação 128
5.4.6.7 Exatidão 128
5.4.6.8 Robustez 129
6 CONCLUSÃO 130
REFERÊNCIAS 132
ANEXOS 140

1 INTRODUÇÃO

Produtos naturais são uma fonte atrativa de diferentes moléculas que possuem possíveis atividades biológicas, o que historicamente conduz esses produtos a serem uma fonte de novos medicamentos (BOLDI, 2004). Para se ter uma idéia do que isto representa Newman; Cragg e Snader (2003) citam que para as terapias de câncer e doenças infecciosas, entre 60 e 75% das drogas utilizadas tem origem em produtos naturais ao que Calixto (2005) corrobora, demonstrando que entre 25 a 30% de todas as drogas disponíveis para terapêutica possuem origem em produtos naturais, sejam eles plantas, micróbios ou animais.

De acordo com a organização mundial da saúde (OMS), aproximadamente 65 a 80% da população dos países em desenvolvimento dependem essencialmente de plantas medicinais, fato ocasionado pela pobreza e falta de acesso à moderna medicina. Todavia existe pouco estudo científico dessas plantas para se confirmar, a qualidade, segurança e eficácia (CALIXTO, 2005).

Uma exceção à regra *Uncaria tomentosa* (Willdenow ex Roemer and Shultes) DC., Rubiaceae, popularmente conhecida como unha-de-gato, é uma planta extensamente estudada por diversos pesquisadores e com suas propriedades terapêuticas já bastante divulgadas. Entre os diversos constituintes químicos já caracterizados, os alcalóides oxindólicos (SANDOVAL *et al.*) são reconhecidos como os marcadores fitoquímicos desta espécie por estarem associados a várias de suas ações farmacológicas (KEPLINGER *et al.*, 1999; HEITZMAN *et al.*, 2005; USP, 2008). Entretanto, existe nesta espécie variação sazonal na produção e armazenamento de diferentes tipos de alcalóides oxindólicos. A mesma planta pode em alguns momentos de sua vida produzir e armazenar alcalóides oxindólicos do tipo pentacíclico (AOP) em maior quantidade e em outros momentos movimentar sua rota metabólica em direção aos alcalóides oxindólicos do tipo tetracíclico (AOT). Aos AOP são atribuídas atividades imunomoduladoras, antitumoral e antinociceptiva, já para os AOT são atribuídas ações no sistema nervoso central e antagonismo em relação aos AOP na indução de células humanas a liberarem fatores reguladores da proliferação de linfócitos (LAUS; KEPLINGER, 1994; LAUS; BROSSNER; KEPLINGER, 1997; WURM *et al.*, 1998; JING-SHAN *et al.*, 2003; ZHANG; CHEN; SIM, 2004). Desta maneira pesquisadores e agências regulatórias passaram a

preocupar-se no sentido de identificar e utilizar para fins terapêuticos apenas plantas da espécie que apresentem majoritariamente o perfil pentacíclico de alcalóides oxindólicos.

A distinção entre planta contendo AOP e AOT é facilmente realizada pela análise do material vegetal por cromatografia líquida de alta eficiência (CLAE) com o emprego de tampões fosfato (potássio e sódio) como fase móvel em vários métodos descritos. (STUPPNER; STURM; KONWALINKA, 1992; LAUS; KEPLINGER, 1994; GANZERA *et al.*, 2001; VALENTE *et al.*, 2006; PEREIRA *et al.*, 2008; USP, 2008). O emprego destas fases móveis requer longo tempo de lavagem com água ao final da análise para garantir que não ocorra precipitação de sal na coluna, isso conduz a um tempo excessivamente longo de análise e redução da vida útil da coluna, tornando o controle de qualidade demorado e caro para laboratórios analíticos.

Além disso, a RDC-48 de 16 de março de 2004, legislação atual para registro de medicamentos fitoterápicos no Brasil, exige para deferimento do registro que sua solicitação seja acompanhada, entre outros documentos, por validação da metodologia analítica para quantificação dos princípios ativos e/ou marcadores de acordo com a Resolução-RE 899 de 29 de maio de 2003 (Guia para validação de métodos analíticos e bioanalíticos). Esta validação deve ser apresentada quando a metodologia analítica utilizada não estiver descrita em farmacopéias ou formulários oficiais, devidamente reconhecidos pela Agência Nacional de Vigilância Sanitária (ANVISA) (BRASIL, 2003; BRASIL, 2004).

Essas exigências constituem desafio para controle de qualidade de medicamentos fitoterápicos, pois, quando formulados em matrizes complexas, como no caso de gel, proporcionam um agravante ao desenvolvimento e validação destas metodologias analíticas para quantificação dos marcadores. Por si a planta e seus derivados, tinturas e extratos, já são considerados uma matriz complexa e quando associados à outra matriz, tão complexa quanto a sua, conduz o analista à necessidade de desenvolver técnicas analíticas que permitam solucionar o problema de Controle de Qualidade de maneira seletiva, reprodutível, robusta, exata e linear.

Assim o desafio de alcançar metodologias analíticas mais rápidas e eficientes para a análise de AO em *Uncaria tomentosa* e seus derivados farmacêuticos, bem como a necessidade de obtenção da renovação do registro de gel contendo tintura de *U. tomentosa* produzido pela empresa Herbarium Laboratório Botânico S.A., motivaram o desenvolvimento desta pesquisa.

2 **OBJETIVOS**

2.1 OBJETIVO GERAL

Desenvolver e validar metodologia analítica para extração e quantificação de alcalóides oxindólicos presentes em gel, para tratamento de *Herpes zoster* tipo 1, contendo tintura de *Uncaria tomentosa* (Willdenow ex Roemer and Shultes) DC., Rubiaceae, de forma a atender ao controle de qualidade do referido produto.

2.2 OBJETIVOS ESPECÍFICOS

- Isolar, caracterizar e determinar pureza de padrão analítico de AOP;
- Desenvolver tampão orgânico e otimizar método em CLAE capaz de separar e distinguir entre AOP e AOT na casca e tintura de *U. tomentosa*;
- Desenvolver e otimizar método do preparo das amostras casca, tintura e gel de *U. tomentosa*;
- Validar as metodologias analíticas desenvolvidas, conforme Resolução-RE 899 de 29 de maio de 2003 (Guia para validação de métodos analíticos e bioanalíticos).

3 REVISÃO DA LITERATURA

3.1 DESCRIÇÃO DA ESPÉCIE

Uncaria tomentosa (Willdenow ex Roemer e Shultes) DC., Rubiaceae, é um grande cipó ou arbusto que chega a atingir de 20 a 30 m e seu ramo principal pode ter mais de 30 cm de diâmetro. É uma trepadeira com espinhos característicos semelhantes a ganchos recurvados nas margens dos ramos, o que levou os espanhóis a chamar essa espécie "uña de gato", em português unha-de-gato. Esses espinhos quando florescem dão lugar a florescências que podem ser de coloração branca e amarela, e estão assentadas em formações circulares, possuindo odor semelhante ao de canela (REINHARD, 1997; KEPLINGER *et al.*, 1999; FALKIEWICZ; LUKASIAK, 2001; GATTUSO; DI SAPIO; LI PEREYRA, 2004).

A espécie em questão segue a classificação taxonômica apresentada no QUADRO 1.

TÁXON	NOMENCLATURA
Divisão	Magnoliophyta
Classe	Magnoliopsida
Subclasse	Asteridae
Ordem	Rubiales
Família	Rubiaceae
Gênero	*Uncaria*
Espécie	*Uncaria tomentosa* DC.

QUADRO 1 – ENQUADRAMENTO TAXONÔMICO SEGUNDO APG II (2003).
FONTE: CASTAÑEDA *et al.* (1998)

Nas regiões em que é encontrada e demais partes do mundo é conhecida por diversos nomes comuns, entre eles destacam-se: no Brasil unha-de-gato; nos demais países latino americanos uña de gato, garabato, vilcacora, samento, garabato amarillo; nos Estados Unidos da América e países da Europa Ocidental cat's claw, cat's crew, hawk's claw, saventaro; na Alemanha e Áustria katzenkralle (VILCHES, 1995; FALKIEWICZ; LUKASIAK, 2001; HEITZMAN *et al.*, 2005).

As espécies que compõe o gênero *Uncaria,* em um total de 34, possuem como habitat natural as regiões tropicais do globo e destas apenas duas são encontradas na América latina (*Uncaria tomentosa* e *Uncaria guianensis*), três na

África e as demais na Ásia (PHILLIPSON; HEMINGWAY; RIDSDALE, 1978; KEPLINGER *et al.*, 1999; HEITZMAN *et al.*, 2005).

As espécies latino americanas são encontradas naturalmente nas florestas tropicais da América Central (Guatemala, Belize, Honduras, El Salvador, Nicarágua, Costa Rica e Panamá) e América do Sul (Brasil, Colômbia, Peru, Equador, Guianas e Bolívia), sendo que ambas possuem ligeira diferença nas características fenotípicas (VILCHES, 1995; POLLITO; TOMAZELLO, 2006).

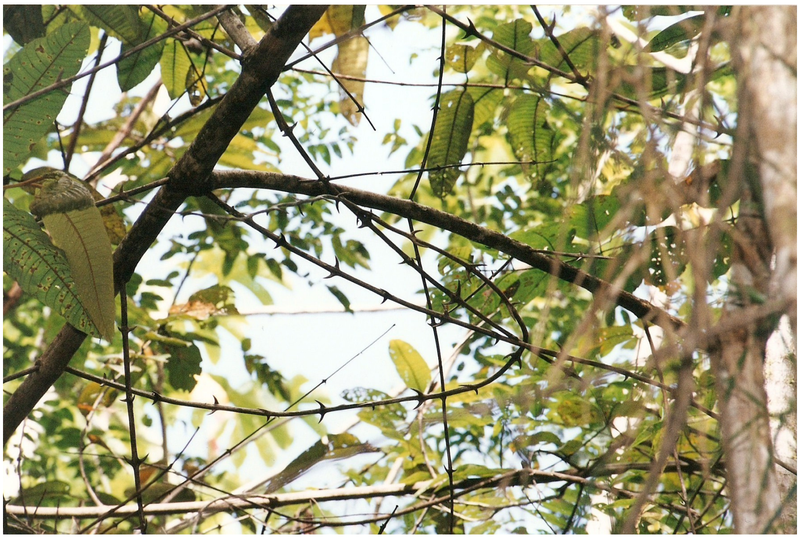

FIGURA 1 – ASPECTO DOS ESPINHOS DE *U. tomentosa* DC., RUBIACEAE.
FONTE: João Alencar de Sousa

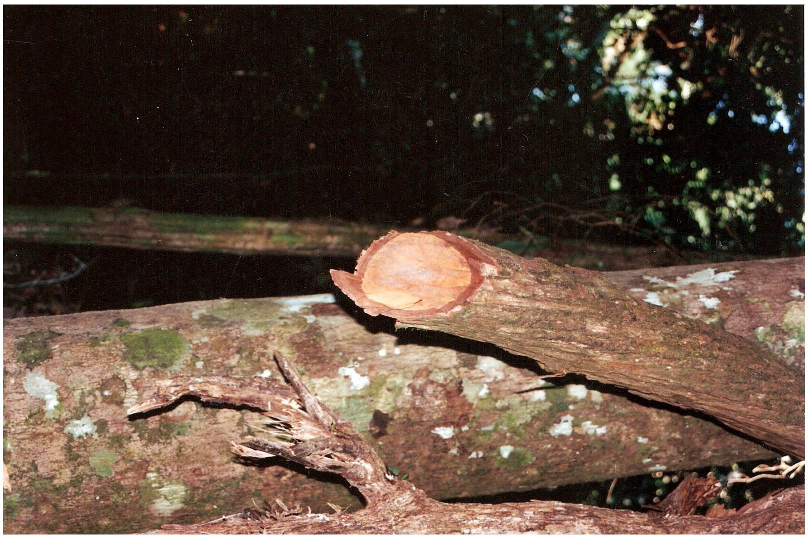

FIGURA 2 – ASPECTO DO CAULE DE *U. tomentosa* DC., RUBIACEAE.
FONTE: João Alencar de Sousa

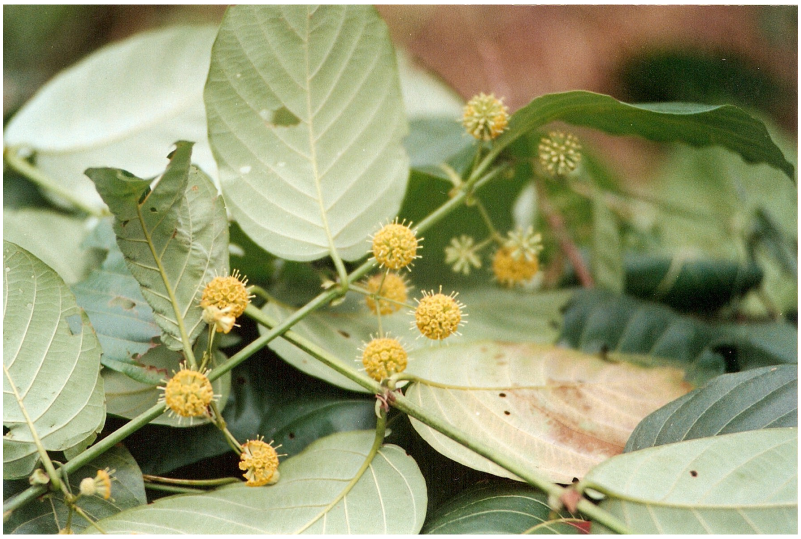

FIGURA 3 – FLORES DE *U. tomentosa* DC., RUBIACEAE.

FONTE: João Alencar de Sousa

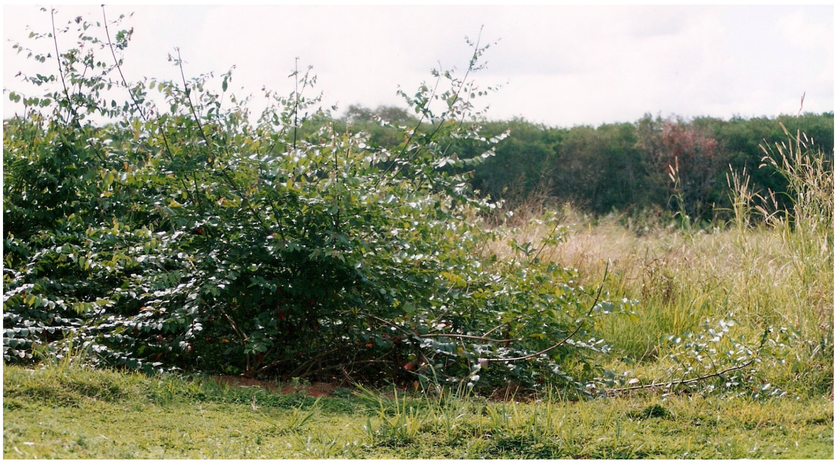

FIGURA 4 – ASPECTO DA TREPADEIRA *U. tomentosa* DC., RUBIACEAE.

FONTE: João Alencar de Sousa

Os autores Gattuso; Di Sapio e Li Pereyra (2004) apresentam a espécie *Uncaria tomentosa* como encontrada principalmente nas regiões de selva alta, entre 300 e 800 m em relação ao nível do mar, enquanto a espécie *Uncaria guianensis* é encontrada nas regiões de selva baixa, entre 100 e 500 m de altitude.

As duas espécies são utilizadas na medicina popular de maneira intercambiável, no entanto, seus constituintes químicos, mais notadamente o perfil alcalóidico e as características anatômicas, como já citado, são bastante diferentes. *U. tomentosa* apresenta concentração significativamente maior de alcalóides em

relação à *U. guianensis* (VILCHES, 1995; SANDOVAL *et al.*, 2002; GATTUSO; DI SAPIO; LI PEREYRA, 2004; POLLITO; TOMAZELLO, 2006)

Segundo Falkiewicz e Lukasiak (2001), uma característica de bastante relevância e que distingue a espécie em questão das demais espécies do gênero *Uncaria* é a grande variação no conteúdo individual de alcalóides entre as diferentes partes da planta, sendo as maiores concentrações encontradas nas cascas do caule e raiz.

Os pesquisadores Laus e Keplinger (1994) acompanharam o desenvolvimento de algumas plantas da espécie mencionada por um período de até sete anos e encontraram variação sazonal no conteúdo alcaloídico bastante significativa. Foi percebido que a mesma planta com o decorrer do tempo alternava entre diferentes alcalóides majoritários. Os mesmos autores estabeleceram após conversas com habitantes nativos da selva peruana, existir três variedades de *U. tomentosa*, que quando tem suas raízes vivas cortadas, exibem colorações diferentes: cinza claro, amarelo amarronzado e vermelho escuro.

3.1.1 Propriedades farmacológicas e constituintes químicos

A utilização da planta com fins medicinais pelos povos nativos da região Amazônica (Asháninka - Peru) data em mais de 2.000 anos. É cultuada por este povo como planta sagrada que possui grandes propriedades terapêuticas (KEPLINGER *et al.*, 1999; WILLIAMS, 2001).

Preparada na medicina tradicional como um decocto das cascas dos ramos e da raiz, o extrato aquoso é utilizado em casos de doenças inflamatórias (artrite, gastrite, inflamações do trato geniturinário e inflamações dérmicas), asma, úlceras gástricas, diabetes, tumores, câncer, processos virais, irregularidades no ciclo menstrual, sensação de fraqueza e gonorréia (VILCHES, 1995; HEITZMAN *et al.*, 2005).

As diferentes propriedades terapêuticas desta planta são atribuídas a uma diversidade de metabólitos secundários já identificados e estudados nesta espécie, que até hoje passam de cinqüenta, entre eles alcalóides oxindólicos e indólicos, polifenóis (flavonóides, proantocianidinas, taninos), glicosídeos triterpenos derivados

do ácido quinico e quinóvico e saponinas (AGUILAR *et al.*, 2002; PILARSKI *et al.*, 2007).

AÇÃO	METABÓLITO	COMPROVAÇÃO DA ATIVIDADE	REFERÊNCIA
Estimulação da atividade fagocítica de macrófagos	Alcalóides	*In vitro / In vivo*	WAGNER, et al. (1985)
Atividade antiviral	Glicosideos do ácido quinovico / alcalóides	*In vitro*	AQUINO, et al. (1989); REIS, et al. (2008)
Atividade antiinflamatória	Glicosideo do ácido quinovico	*In vivo*	AQUINO, et al. (1991)
Atividade antimutagênica	Diferentes frações	*In vitro*	RIZZI, et al. (1993)
Antinociceptiva	Fração enriquecida de alcalóides	*In vitro / In vivo*	JURGENSEN, et al. (2005)
Antioxidante	Proantocianidinas, ácidos fenólicos e alcalóides	*In vitro*	GONÇALVES; DINIS; BATISTA (2005); PILARSKI (2006)
Atividade antileucêmica	Alcalóides	*In vitro*	STUPPNER, et al.(1993); PILARSKI (2007)
Atividade pró-apoptótica em células leucêmicas	Alcalóides	*In vitro*	BACHER (2005)

QUADRO 2 – CORRELAÇÃO ENTRE OS PRINCIPAIS METABÓLITOS DE *U. tomentosa* E SUAS PROPRIEDADES FARMACOLÓGICAS.

Notadamente os AO são os constituintes mais extensamente estudados na planta. Esses alcalóides não são característicos da espécie, uma vez que são encontrados em espécies do gênero *Mitragyna* (Ex. *Mitragyna hirsuta*), além de outras espécies do gênero *Uncaria* como *U. canescens, U. glabatra, U. attenuata, U. orientalis* entre outras e já possuem suas estruturas bem caracterizadas. (JOLLIFFE; SHELLARD, 1973; PHILLIPSON; HEMINGWAY, 1975b; PHILLIPSON; HEMINGWAY, 1975a; PHILLIPSON; HEMINGWAY; RIDSDALE, 1978; PHILLIPSON; SUPAVITA; ANDERSON, 1982; WAGNER; KREUTZKAMP; JURCIC, 1985; LAUS; KEPLINGER, 1994; WAGNER; BLADT, 1996; LAUS; BROSSNER; KEPLINGER, 1997; LAUS; KEPLINGER, 1997).

CLASSE	ALCALÓIDES PENTACÍCLICOS	ALCALÓIDES TETRACÍCLICOS
Alcalóides oxindólicos	Pteropodina (Uncarina C)	Rincofilina
	Isopteropodina (Uncarina E)	Isorincofilina
	Especiofilina (Uncarina D)	Corinoxeina
	Uncarina F	Isocorinoxeina
	Mitrafilina	
	Isomitrafilina	
Alcalóides indólicos	Akuammigina	Hirsutina
	Tetrahidroalstonina	Dihidrocorinantheina
	Isoajmacilina	Hirsuteina
		Corinantheina

QUADRO 3 – PRINCIPAIS ALCALOIDES ENCONTRADOS EM *U. tomentosa*
FONTE: KEPLINGER *et al.* (1999)

O estudo exaustivo desses alcalóides levou recentemente alguns autores a inferirem sobre a existência de dois quimiotipos de *Uncaria tomentosa* ocorrendo naturalmente na natureza, os quais podem ser diferenciados pelo seu conteúdo alcaloídico. Um dos quimiotipos conteria principalmente alcalóides oxindólicos pentacíclicos (especiofilina, uncarina F, mitrafilina, isomitrafilina, pteropodina e isopteropodina) e o outro conteria grande quantidade de alcalóides oxindólicos tetracíclios (rincofilina e isorincofilina) (LAUS; BROSSNER; KEPLINGER, 1997; WURM *et al.*, 1998).

No entanto, alguns pesquisadores questionam a classificação adotada de dois quimiotipos diferentes para a mesma espécie, sustentam que esses perfis alcaloídicos podem ser intercambiáveis sob condições sazonais (PILARSKI *et al.*, 2007). Conforme já mencionado, o trabalho publicado por Laus e Keplinger (1994) descreve que a mesma planta pode alterar seu conteúdo alcaloídico, apresentando em determinado ano majoritariamente alcalóides tetracíclicos e, em outro ano, majoritariamente alcalóides pentacíclicos.

Estes dados são importantes, pois ainda segundo Laus e Keplinger (1997), as principais formas de adulterações/falsificações da planta contendo majoritariamente AOP são exatamente a mistura de partes da planta com o perfil tetracíclico e partes de *Uncaria guianensis*, assim como partes de madeira, lenho e cascas de plantas indeterminadas. Reinhard (1997) fortalece a preocupação na distinção entre plantas contendo diferentes perfis alcaloídicos, descrevendo que somente a planta com o perfil pentacíclico, possui valor terapêutico, ao que corroboram as informações de Keplinger e colaboradores (1999), citando os autores que os curandeiros da tribo Asháninka na selva peruana utilizam apenas plantas que apresentam o perfil pentacíclico de alcalóides no tratamento dos diferentes males.

Após os estudos de Wagner; Kreutzkamp e Jurcic (1985) foram atribuídas importantes propriedades imunomoduladoras aos AOP por sua capacidade de estimular a fagocitose em macrófagos. Além disso, é conhecido que os AOT possuem atividade acentuada no Sistema Nervoso Central – SNC (redução da pressão sanguínea, inibição de arritmia cardíaca, inibição da condução do estímulo nervoso) (WAGNER; KREUTZKAMP; JURCIC, 1985; WURM *et al.*, 1998; JING-SHAN *et al.*, 2003).

Já em 1998, pesquisadores austríacos encontraram antagonismo na ação dos AOT em relação aos AOP na ação de estímulo para liberação de fator regulador da proliferação de linfócitos por células endoteliais humanas, *in vitro*. Como conseqüência, misturas do material dessa planta não controladas em seu conteúdo alcaloídico poderiam ser colocadas em dúvida quanto ao seu efeito terapêutico, caso os resultados *in vitro* repitam-se *in vivo* (WURM *et al.*, 1998).

Considerando todas essas informações, recentemente a farmacopéia dos Estados Unidos XXXI (USP) publicou monografia para esta droga definindo como limite máximo para AOT 0,05% e limite mínimo para os AOP de 0,3% (USP, 2008).

Devido a esses estudos iniciais, pensou-se que as principais atividades farmacológicas da unha-de-gato estivessem associadas a estes componentes alcaloídicos, com o que corrobora o estudo de alguns autores que encontraram correlação entre a atividade antiinflamatória e antiviral, por exemplo, e a quantidade de alcalóides pentacíclicos presente no extrato da planta (AGUILAR *et al.*, 2002; REIS *et al.*, 2008).

Contrariando os dados citados anteriormente, vários estudos posteriores aos de Wagner e colaboradores relataram a existência de associação entre a concentração de glicosídeos triterpênicos derivados do ácido quinóvico e a atividade antiinflamatória da unha-de-gato. Os autores, no entanto, não descartaram que esta atividade fosse em decorrência da ação sinérgica entre diferentes constituintes químicos da planta (AQUINO; SIMONE; PIZZA, 1989; AQUINO *et al.*, 1991).

Outros estudos com extratos aquosos da planta isentos de alcalóides, comprovaram a atividade antiinflamatória do extrato mesmo na ausência destes componentes assim colocou-se em dúvida a correlação entre a atividade antiinflamatória da planta e os AOP (SANDOVAL *et al.*, 2002; AKESSON *et al.*, 2003; SHENG *et al.*, 2005).

Entretanto ainda hoje esta planta é marcada e seu controle de qualidade é realizado em função da sua composição e concentração de AO. Existem várias razões que explicam o fato, desde a grande concentração destas moléculas na espécie, a quantidade de métodos descritos para análise via CLAE, até a já citada e comprovada correlação entre algumas atividades farmacológicas, como imunomodulação, atividade antileucêmica e antinociceptiva e estes alcalóides (AGUILAR *et al.*, 2002; PILARSKI *et al.*, 2006; PILARSKI *et al.*, 2007; REIS *et al.*, 2008).

3.2 ALCALÓIDES OXINDÓLICOS

Os alcalóides são bases nitrogenadas encontradas principalmente em plantas, mas também podem ser encontrados em menores proporções em microorganismos e animais. Podem apresentar um ou mais átomos de nitrogênio, geralmente como aminas primárias, secundárias ou terciárias, o que geralmente confere características básicas aos alcalóides, facilitando seu isolamento e purificação, uma vez que sais hidrossolúveis podem ser formados na presença de ácidos minerais. O nome alcalóide deriva consequentemente de álcali (DEWICK, 1997).

Ainda segundo Dewick (1997), os átomos de nitrogênio constituintes dos diversos alcalóides existentes, são provenientes de suas moléculas precursoras na rota biossintética, os aminoácidos. Geralmente os esqueletos carbônicos destes aminoácidos precursores são bastante conservados na estrutura final do alcalóide, apesar do carbono relativo ao ácido carboxílico ser geralmente perdido via descarboxilação. Os principais aminoácidos precursores dos alcalóides são ornitina, lisina, ácido nicotínico, tirosina, triptofano, ácido antranílico e histidina, estes podem receber a incorporação de blocos derivados das vias do acetato, chiquimato ou mevalonato para originarem as diversas classes de alcalóides. Todavia existe exceção a esta regra, pois alguns alcalóides incorporam seus átomos de nitrogênio por transaminação, assimilando apenas o átomo de nitrogênio do aminoácido, o restante da molécula é derivado do acetato, chiquimato ou tem origem nos terpenoides, esteroides.

No gênero *Uncaria* todos os alcalóides encontrados possuem um núcleo indólico ou oxindólico, com exceção do harmana e são derivados da condensação da triptamina (grupamento indólico derivado do triptofano) com o monoterpeno ciclopentanoide secologanina. A resultante estrictosidina dá origem aos heterohiohimbinicos pentacíclicos e tetracíclicos e aos correspondentes oxindólicos (PHILLIPSON; HEMINGWAY; RIDSDALE, 1978).

Portanto, os alcalóides oxindólicos tem sua gênese na via do ácido chiquímico (1), que por sucessivas reações com o fosfoenol piruvato (PEP / 2) origina o intermediário ácido chorísmico (3), posteriormente tornando-se ácido antranílico (4) e por sucessivos rearranjos, após condensação com fosforibosil PP (5), dá origem ao grupamento indólico (6) desta classe de alcalóides. Finalmente o grupamento indólico condensado à l-serina (7) origina o l-triptofano (8), intermediário chave na via dos alcalóides indólicos terpenoides, FIGURA 5 (DEWICK, 1997).

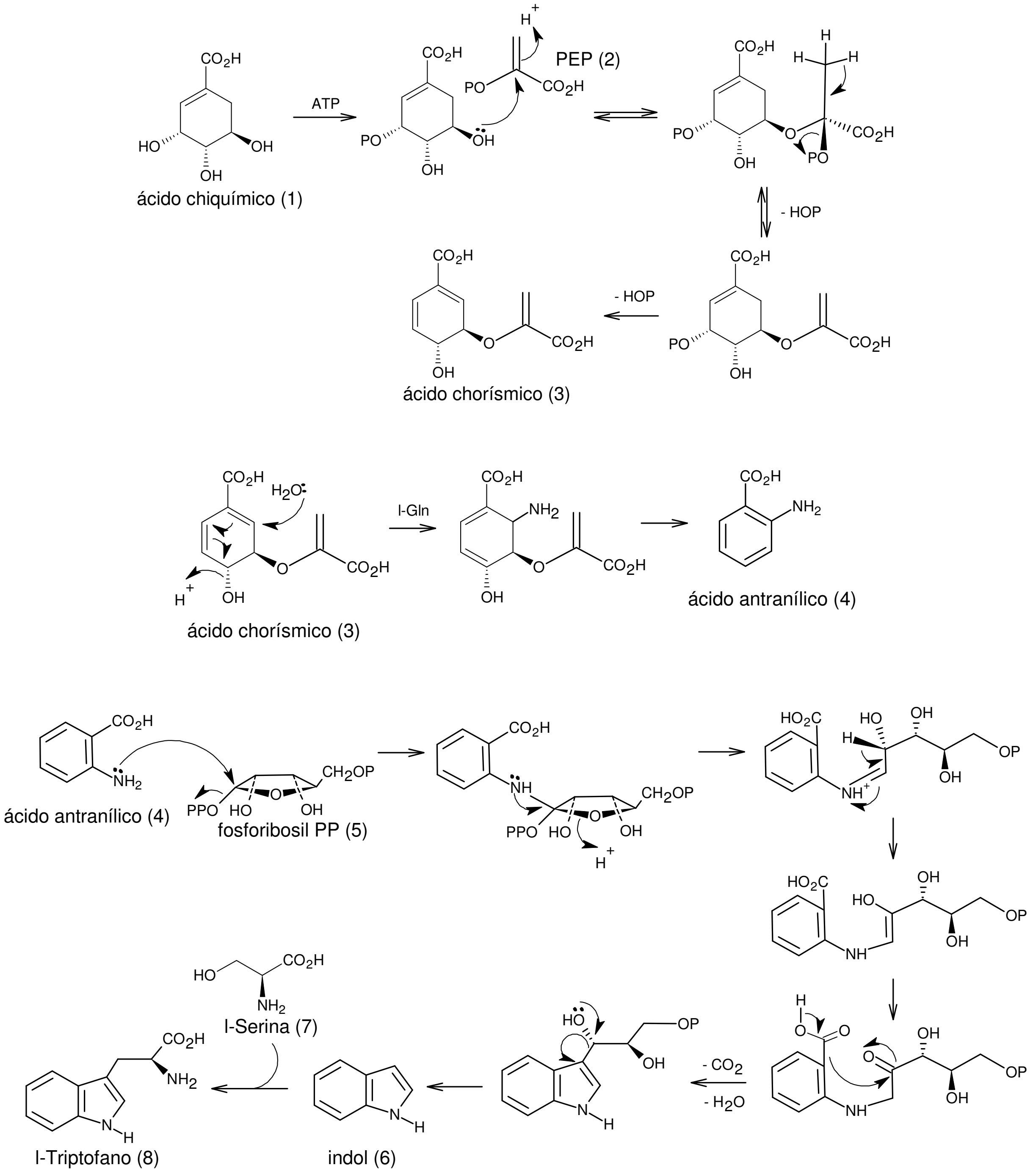

FIGURA 5 – ROTA BIOSSINTÉTICA DO l-TRIPTOFANO

Cabe ressaltar que segundo Dewick (1997), os alcalóides indólicos terpenoides são um dos maiores grupos dessa classe química no reino Plantae. Majoritariamente são encontrados em oito famílias de plantas, das quais os principais exemplos são Apocynaceae, Loganiaceae e Rubiaceae. Praticamente todas as estruturas são compostas de uma porção triptamina e outro fragmento geralmente C_9 ou C_{10} dos quais três tipos principais são distinguidos: 1) *Corynanthe*

como ajmalicina e akuammicina; 2) *Aspidosperma* como a tabersonina; 3) *Iboga* como a catarantina. A porção C_9 ou C_{10} tem origem nos terpenoides e o secoiridoide secologanina (9) é o que geralmente se liga ao triptofano, via reação de Mannich, para originar esta classe em especial.

Os AOP e AOT são alcalóides do tipo heterohiohimbina, muito próximos ao tipo *Corynanthe*, sendo variantes oxidadas dos alcalóides iohimbinicos (10), como a ajmalicina (11). As partes em negrito das estruturas apresentadas a seguir evidenciam relação precursor-produto na rota biossintética.

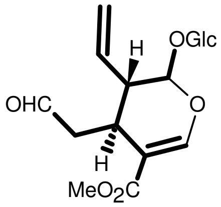

secologanina (9)

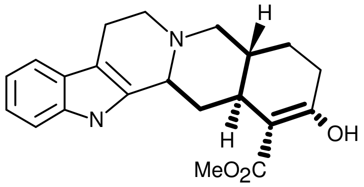

iohimbina (10)

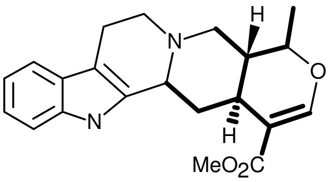

ajmalicina (11)

Os alcalóides oxindólicos pentacíclicos (12) e tetracíclicos (13), derivados das estruturas apresentadas anteriormente, possuem as estruturas gerais representadas abaixo.

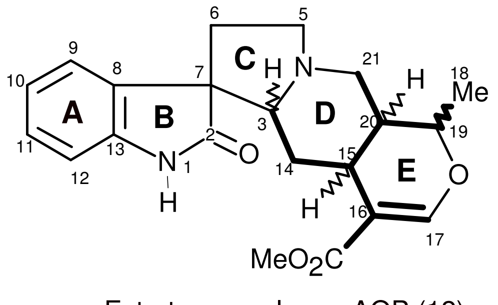

Estrutura geral para AOP (12)

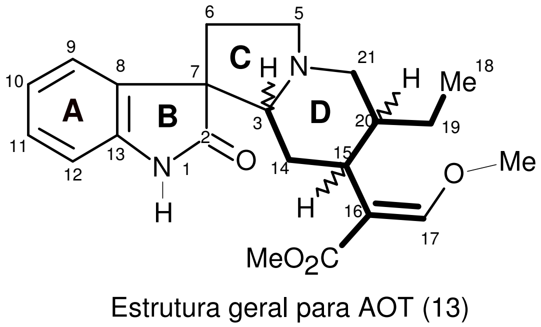

Estrutura geral para AOT (13)

Como citado, esses alcalóides são encontrados em várias plantas do gênero *Mitragyna* e *Uncaria*, utilizadas com freqüência na medicina popular. Teoricamente trinta e dois esteroisômeros da fórmula geral apresentada para AOP são possíveis, pois sua estrutura possui cinco centros assimétricos ou estereogênicos. No entanto,

biogeneticamente a configuração do centro assimétrico na posição C-15 é fixo na forma (*S*) devido ao precursor comum strictosidina, assim o número de possíveis isômeros pode ser restrito a dezesseis (LAUS; WURST, 2003). A classificação diasteroisomérica dos alcalóides oxindólicos do tipo heterohiohimbina foi estabelecida em oito grupos: normal *S* (C-3 Hα, C-20 Hβ), normal *R* (C-3 Hα, C-20 Hβ), *pseudo S* (C-3 Hβ, C-20 Hβ), *pseudo R* (C-3 Hβ, C-20 Hβ), *allo S* (C-3 Hα, C-20 Hα), *allo R* (C-3 Hα, C-20 Hα), *epiallo S* (C-3 Hβ, C-20 Hα) *e epiallo R* (C-3 Hβ, C-20 Hα). Os diferentes tipos *S* ou *R* são relativos à conformação em C-7, sendo que *R* é referente à carbonila lactâmica acima do plano dos anéis C-D e *S* o oposto. (SHAMMA *et al.*, 1967; PHILLIPSON; HEMINGWAY, 1975a; SEKI *et al.*, 1993).

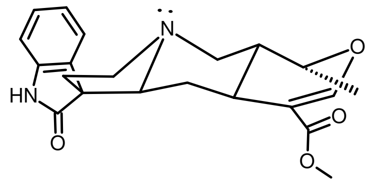

normal *S*

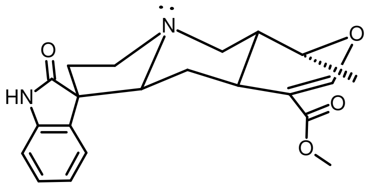

normal *R*

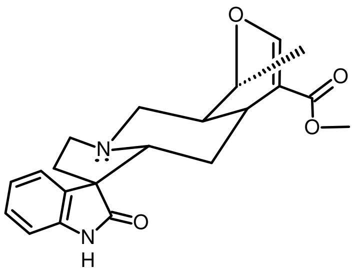

epiallo S

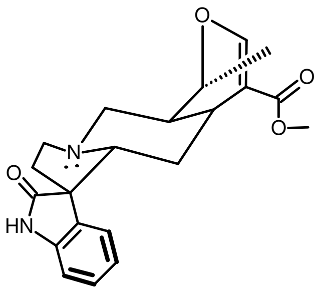

epiallo R

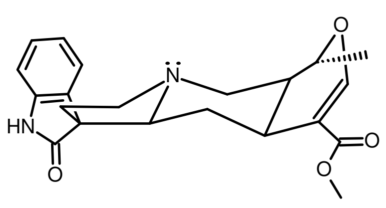

allo S

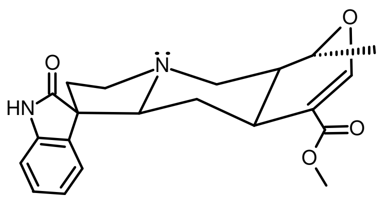

allo R

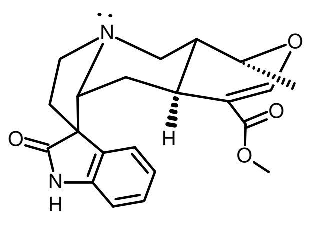

pseudo S

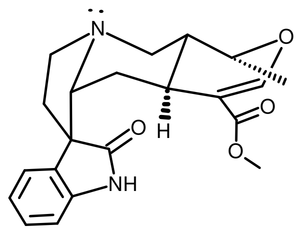

pseudo R

As estruturas anteriores demonstram que os alcalóides do tipo normal, são caracterizados pela relação do tipo *E (trans)* nas junções dos anéis C/D e D/E, já os representantes do tipo *epiallo* são caracterizados por relação do tipo *Z (cis)* na junção dos anéis D/E, enquanto que os alcalóides do tipo *allo* são caracterizados por relação do tipo *E (trans)* e *Z (cis)* nas junções dos anéis C/D e D/E respectivamente (SEKI *et al.*, 1993).

Importante observar que todas as estruturas relacionadas ocorrem como par de epímeros em C(7) ou mesmo em C(3), esses pares em C(7) podem por diferentes rearranjos se interconverterem (SHAMMA *et al.*, 1967; PHILLIPSON; HEMINGWAY; RIDSDALE, 1978; LAUS *et al.*, 1996; LAUS; WURST, 2003). Epímeros segundo Solomons e Fryhle (2006), são diasteroisômeros que diferem na configuração em apenas um centro estereogênico como por exemplo d-Glucose e d-Manose.

Dessas estruturas a de tipo *pseudo* ainda não foi encontrada na natureza, provavelmente devido à séria interferência estérica existente entre a parte oxindólica e a parte inferior do anel D da molécula, logo, esta estrutura pode epimerizar facilmente para compostos do tipo normal, FIGURA 6 (SEKI *et al.*, 1993).

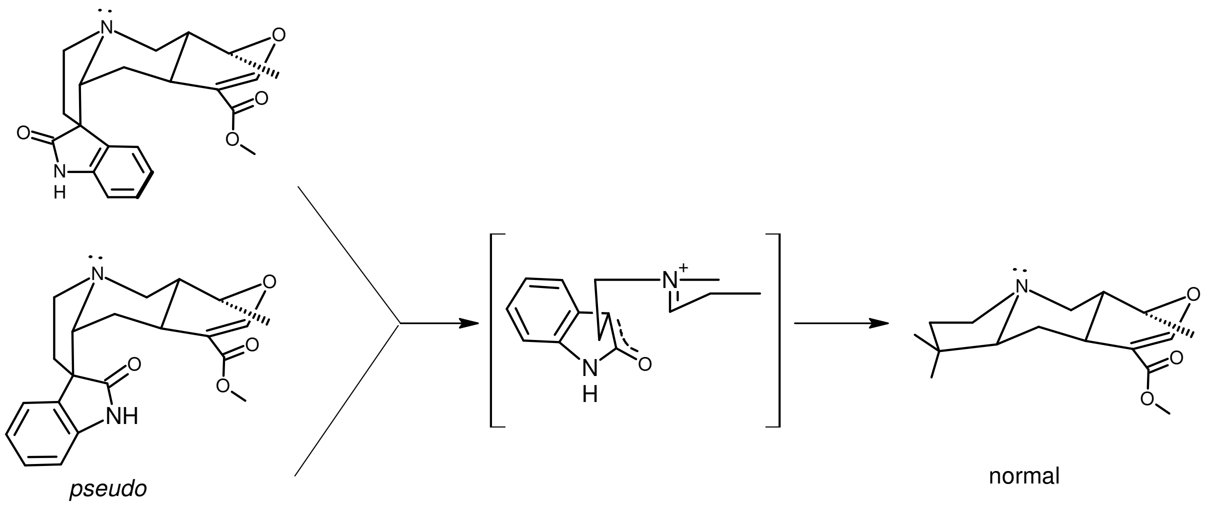

FIGURA 6 – INTERCONVERSÃO DE ALCALÓIDE OXINDOLICO DO TIPO *PSEUDO* PARA O TIPO NORMAL

Deriva do citado anteriormente que o número de isômeros considerados viáveis da estrutura geral apresentada para AOP, pode ser limitado a doze, QUADRO 4.

TIPO	CONFIG. DO C_3-H	CONFIG. DO C_{20}-H	RELAÇÃO DOS ANÉIS D/E	CONFIG. DO C_{19}-H	CONFIG. DO C_7	EXEMPLO
Normal	*S* (α)	*R* (β)	*E*	*S* (β)	*S*	Isomitrafilina
					R	Mitrafilina
				R (α)	*S*	Uncarina A (isoformosanina)
					R	Uncarina B (formosanina)
Epiallo	*R* (β)	*S* (α)	*Z*	*S* (β)	*S*	Uncarina D (especiofilina)
					R	Uncarina F
				R (α)	*S*	Rauniticina-epiallo-oxindol A
					R	Rauniticina-epiallo-oxindol B
Allo	*S (α)*	*S* (α)	*Z*	*S* (β)	*S*	Uncarina E (isopteropodina)
					R	Uncarina C (pteropodina)
				R (α)	*S*	Rauniticina-allo-oxindol A
					R	Rauniticina-allo-oxindol B
Pseudo	*R* (β)	*R* (β)	*E*	*S* (β)	*S*	----
					R	----
				R (α)	*S*	----
					R	----

QUADRO 4 – ISÔMEROS POSSÍVEIS PARA ESTRUTURA GERAL DOS AOP

FONTE: SEKI *et al.* (1993)

NOTA: Config.: Configuração

Os dados apresentados no quadro foram posteriormente confirmados por análises cristalográfica por Laus e Wurst (2003) e Muhammad e colaboradores (2001).

Da análise do quadro pode ser depreendido que para todas as estruturas a diferença entre uma substância e outra é alteração espacial do esqueleto oxindólico do tipo heterohiohimbina, o que torna distinguível os isômeros apenas com a associação de diferentes técnicas analíticas, principalmente com a utilização de ressonância magnética nuclear (RMN) e perfis de fragmentação por espectrometria de massas (EM).

Na *Uncaria tomentosa*, os pares epiméricos em C(7), que ocorrem naturalmente são: especiofilina (14) / uncarina F (15), mitrafilina (16) / isomitrafilina (17), pteropodina (18) / isopteropodina (19) e rincofilina (20) / isorincofilina (21).

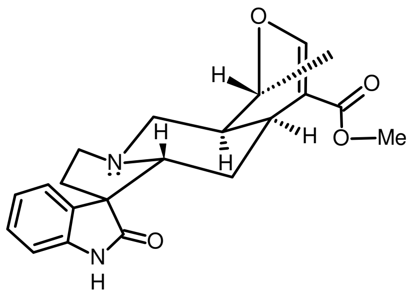

especiofilina (14)

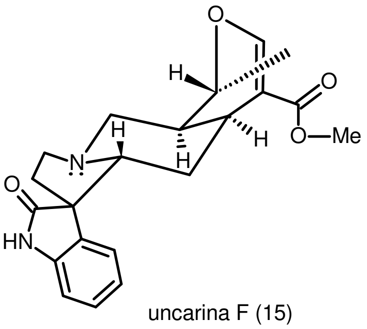

uncarina F (15)

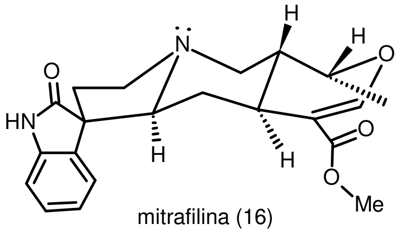

mitrafilina (16)

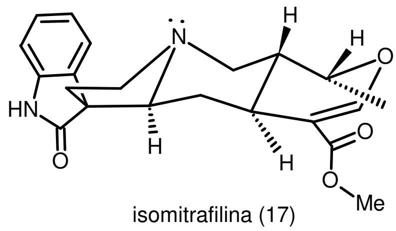

isomitrafilina (17)

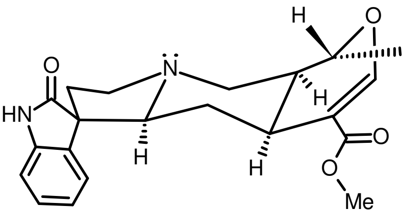

pteropodina (18)

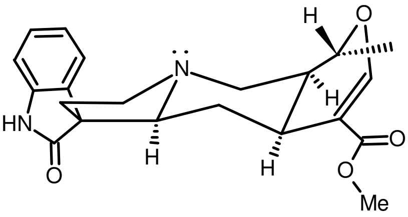

isopteropodina (19)

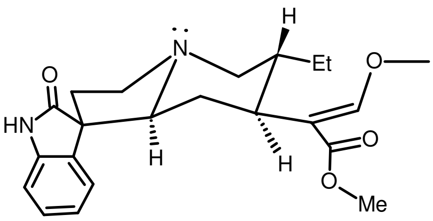

rincofilina (20)

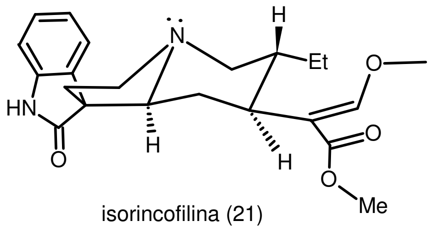

isorincofilina (21)

Esses alcalóides, como já citado, são associados a algumas das ações farmacológicas da *Uncaria tomentosa* e tradicionalmente analisados com o emprego de CLAE em fase reversa e fase móvel composta de tampão fosfato (JOLLIFFE; SHELLARD, 1973; PHILLIPSON; SUPAVITA; ANDERSON, 1982; STUPPNER; STURM; KONWALINKA, 1992; LAUS; KEPLINGER, 1994; TIRILLINI, 1996; LAUS; BROSSNER; KEPLINGER, 1997; GANZERA *et al.*, 2001; PEREIRA *et al.*, 2008).

O emprego de CLAE em fase reversa para análise desses alcalóides pode ser explicado pela capacidade das estruturas apresentadas realizarem interações hidrofóbicas e do tipo dipolo-dipolo com os grupamentos C-18 das colunas. Além disso, possuem excelentes grupos cromóforos como anel aromático do grupamento indólico e duplas ligações conjugadas que facilitam a detecção em ultravioleta no comprimento de onda próximo aos 245 nm.

3.3 DISPERSÃO DE MATRIZ EM FASE SÓLIDA

A preparo de amostras tem sido o grande limitador de procedimentos analíticos, principalmente aqueles voltados à análise de traços de componentes, concentrações na ordem de partes por milhão (ppm) (GARCIA-LOPEZ; CANOSA; RODRIGUEZ, 2008).

Ainda segundo o mesmo autor, o sucesso de um determinado método de análise, para compostos orgânicos presentes em matrizes complexas, depende de um adequado balaço entre o rendimento e a seletividade do processo de extração.

Todavia métodos clássicos para determinação de traços de compostos são geralmente constituídos de várias etapas, tipicamente baseados em extração exaustiva da matriz e subseqüente remoção do material co-extraído por sucessivas etapas de limpeza (KRISTENSON; RAMOS; BRINKMAN, 2006).

Cabe enfatizar que tais métodos empregam grande quantidade de amostra, sorbentes e solventes orgânicos de alta qualidade, requerem muito manuseamento da amostra, são caros em termos de tempo e consumo de material e a sua velocidade no preparo de amostras é muito baixa, não sendo condizente com os desafios das análises modernas nos laboratórios de controle de qualidade das indústrias (KRISTENSON; RAMOS; BRINKMAN, 2006).

Desta forma, Dispersão de Matriz em Fase Sólida (MSPD) tem encontrado grande aplicação como processo analítico para a preparação, extração e fracionamento de amostras biológicas sólidas, semi-sólidas e altamente viscosas (BARKER, 2007).

Segundo Garcia-Lopez; Canosa e Rodriguez (2008), MSPD possui características únicas como uma técnica de preparação de amostra. O uso de condições brandas na análise como temperatura ambiente e pressão atmosférica com uma combinação adequada do sorbente dispersante e do solvente de eluição, geralmente fornece recuperações adequadas e média seletividade.

Outras vantagens em relação às formas clássicas de preparo da amostra seriam (BOGIALLI; DI CORCIA, 2007):

1) Baixo custo por extração
2) Procedimento analítico é drasticamente simplificado e reduzido
3) A possibilidade de formação de emulsão é eliminada
4) Não necessidade de instrumentação

5) Moderado consumo de solventes orgânicos

Estes pontos levantados corroboram com os dados de que desde sua introdução em 1989 por Steven A. Barker, MSPD foi citada até o momento como o método de extração empregado em mais de 250 publicações (BARKER, 2007).

A técnica consiste em basicamente triturar uma amostra viscosa, sólida ou semi-sólida com um suporte sólido, geralmente sílica derivatizada com uma fase orgânica (C_{18}) em sua superfície, em um graal de vidro. O suporte sólido serve como abrasivo para romper a arquitetura celular da amostra, quebrando o material em pequenos pedaços. A presença da fase orgânica ligada à sílica confere uma propriedade especial ao sorbente, a de dissolver e dispersar de acordo com seus coeficientes de distribuição. Após esta etapa a amostra já dispersa no suporte sólido é introduzida em uma seringa de polipropileno contendo um filtro em sua extremidade inferior para evitar perda de amostra. Finalmente a amostra é compactada com o êmbolo de uma seringa e sobre a mesma, colocada outro filtro. A coluna finalizada está pronta para eluição conforme o solvente, ou solventes, selecionados (BARKER, 2000; BARKER, 2007; GARCIA-LOPEZ; CANOSA; RODRIGUEZ, 2008).

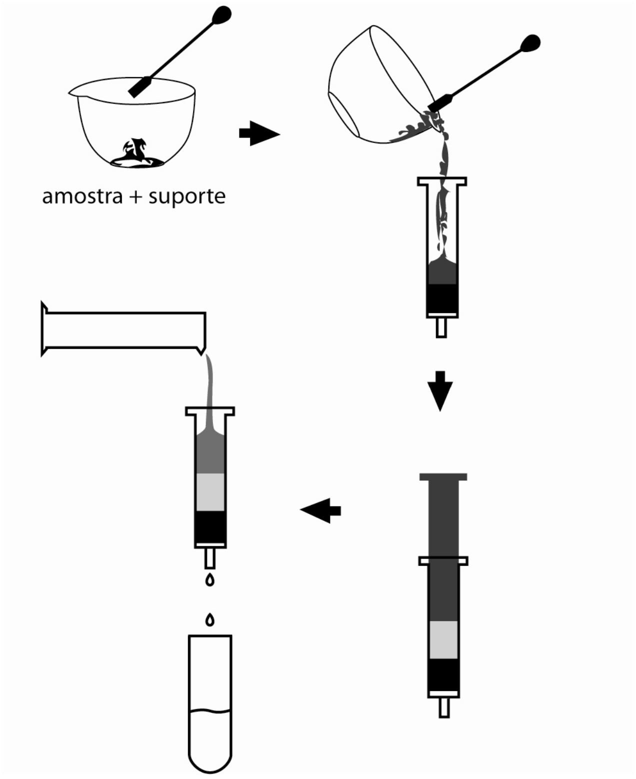

FIGURA 7 – MÉTODO DE DISPERSÃO DE MATRIZ EM FASE SÓLIDA

O equilíbrio de partição e adsorção, similares àqueles que ocorrem na coluna cromatográfica, são responsáveis pela distribuição do analito entre a amostra e o solvente de eluição (GARCIA-LOPEZ; CANOSA; RODRIGUEZ, 2008).

Atualmente diferentes tipos de suportes sólidos são utilizados no desenvolvimento de métodos para MSPD, entre eles podem ser citados amino-propil sílica, florisil, aluminas, sílica e terra diatomácea (BARKER, 2000; KRISTENSON; RAMOS; BRINKMAN, 2006; BARKER, 2007; GARCIA-LOPEZ; CANOSA; RODRIGUEZ, 2008). Entretanto, é preciso lembrar que para fases normais a interação com os componentes da amostra é realizada apenas pela adsorção e estas não são capazes de dissolver a matriz da amostra. Ligações de hidrogênio entre amino grupos na estrutura dos analitos e os silanóis da sílica, contribuem para a retenção de espécies alvo na MSPD (GARCIA-LOPEZ; CANOSA; RODRIGUEZ, 2008).

Como é um método extrativo muito versátil, rápido e de fácil execução, possui um grande atrativo para aplicação no controle de qualidade de medicamentos fitoterápicos, de maneira a se eliminar a matriz complexa que caracteriza esses produtos e alcançar uma extração seletiva das moléculas de interesse.

3.4 PLANEJAMENTO FATORIAL DE EXPERIMENTOS

A análise cromatográfica usualmente envolve três etapas: preparo da amostra, separação e quantificação dos componentes (FERREIRA *et al.*, 2007).

Destas, as etapas de preparo da amostra e separação dos componentes podem sofrer duas abordagens para estabelecimento das melhores condições de análise. A primeira seria avaliar o efeito da alteração de uma variável por vez, mantendo todas as demais constantes durante o experimento, modelo univariado (GARRIDO-LOPEZ; TENA, 2005).

O modelo univariado apresenta como inconvenientes não permitir a predição do que ocorreria se as outras variáveis também fossem alteradas, não fornece informação sobre os valores ótimos para cada parâmetro, apresenta um conjunto de dados desconectados, não faz um mapeamento do espaço experimental, exige muitas análises e é pouco eficiente (PELLATI *et al.*, 2005).

A segunda abordagem seria a utilização da ferramenta estatística planejamento fatorial de experimentos, modelo multivariado. Esta metodologia permite configurar os testes de maneira a estudar todas as variáveis ao mesmo tempo. As principais vantagens deste tipo de experimento seriam a redução do número de análises necessárias e uma completa exploração do domínio experimental (GARRIDO-LOPEZ; TENA, 2005).

Basicamente a análise multivariada é realizada em dois momentos. Na etapa de triagem modelos lineares indicam quais fatores, dentro de valores máximos e mínimos estabelecidos pelo pesquisador, influenciam a análise e são importantes investigar. Para uma triagem de n variáveis e avaliação de dois níveis para cada uma delas (valores máximos e mínimos), são necessários $2 \times 2 \times ... \times 2 = 2^n$.

Em seguida a etapa de otimização emprega modelos quadráticos para criar um mapa válido do domínio experimental, ou seja, indicar o valor ótimo para cada parâmetro apontado como estatisticamente significativo na etapa de triagem. Idealmente os testes em cada uma das etapas devem ser randomicamente executados para assegurar boa estimativa do erro experimental. (FERREIRA *et al.*, 2007).

A criação de um mapa válido do domínio experimental é obtida eficientemente por gráficos de superfície de resposta, que permitem a sinalização do valor ótimo para cada parâmetro avaliado. Para tratamento e desenho dos mapas os resultados são processados por diferentes modelos quadráticos, entre eles os mais comuns são design composto central, Box-Behnken e Doehlert.

Design composto central é um tipo de design composto completo de três níveis nos quais os fatores são avaliados em níveis que formam um cubo, com pelo menos um valor constituindo o ponto central (o), outros formando os vértices (-1 ou +1) e os demais os pontos axiais ou estrela (α ou $-\alpha$) (FERREIRA *et al.*, 2007).

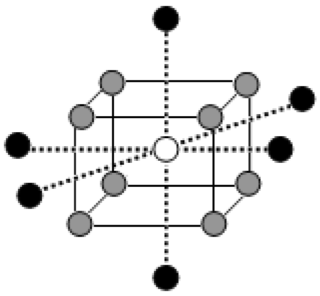

FIGURA 8 – DESIGN COMPOSTO CENTRAL PARA TRÊS FATORES AVALIADOS
FONTE: FERREIRA *et al.* (2007)
NOTA: Pontos negros – pontos axiais; pontos cinzas – pontos cúbicos; ponto branco – ponto central

O número total de ensaios neste modelo é dado por $n = 2^k + 2k + m$, em que 2^k representa o número de pontos fatoriais, 2k o número de pontos axiais e m o numero de replicatas centrais (NETO; SCARMINO; BRUNS, 2003).

Já Box-Behnken não é frequentemente utilizado para otimização de etapas em análises cromatográficas, embora constitua uma alternativa para o design composto central. Para este modelo são cruzadas as informações dos fatores (variáveis) em níveis extremos com os valores em níveis médios dos demais fatores.

Finalmente o Doehlert constitui outra classe de planejamento fatorial na qual é possível estudar fatores diversos com diferentes números de níveis para cada um. É útil em situações onde alguns fatores merecem maior atenção do que outros (FERREIRA *et al.*, 2007).

3.5 VALIDAÇÃO DE MÉTODOS ANALÍTICOS

Para se garantir que um novo método analítico gere informações confiáveis e interpretáveis sobre a amostra, ele deve sofrer uma avaliação denominada validação (RIBANI *et al.*, 2004).

Com a Resolução RE n° 899 de 29 de maio de 2003 – Guia para validação de métodos analíticos e bioanalíticos – a ANVISA passou a exigir para os produtos farmacêuticos a serem registrados no Brasil, estudos de validação das suas metodologias analíticas de controle de qualidade dos princípios ativos. As metodologias precisam ser avaliadas segundo critérios previamente definidos nos parâmetros de Seletividade, Linearidade, Intervalo, Precisão, Limite de detecção,

Limite de quantificação, Exatidão e Robustez de acordo com a finalidade da metodologia avaliada, QUADRO 5.

CATEGORIA	FINALIDADE DO TESTE
I	Testes quantitativos para a determinação do princípio ativo em produtos farmacêuticos ou matérias-primas
II	Testes quantitativos ou ensaio limite para a determinação de impurezas e produtos de degradação em produtos farmacêuticos e matérias-primas
III	Testes de performance (por exemplo: dissolução, liberação do ativo)
IV	Testes de identificação

QUADRO 5 – CATEGORIAS DOS TESTES SUBMETIDOS À VALIDAÇÃO

FONTE: BRASIL (2003)

Assim para cada categoria são exigidos que se avaliem determinados parâmetros de maneira a comprovar a capacidade do método em fornecer resultados confiáveis, QUADRO 6.

PARÂMETRO	CATEGORIA I	CATEGORIA II		CATEGORIA III	CATEGORIA IV
		QUANTITATIVO	ENSAIO LIMITE		
Especificidade/ Seletividade	***Sim***	Sim	Sim	*	Sim
Linearidade	***Sim***	Sim	Não	*	Não
Intervalo	***Sim***	Sim	*	*	Não
Precisão - Repetibilidade	***Sim***	Sim	Não	Sim	Não
Precisão - Intermediária	********	**	Não	**	Não
Limite de detecção	***Não***	Não	Sim	*	Não
Limite de quantificação	***Não***	Sim	Não	*	Não
Exatidão	***Sim***	Sim	*	*	Não
Robustez	***Sim***	Sim	Sim	Não	Não

QUADRO 6 – ENSAIOS NECESSÁRIOS PARA VALIDAÇÃO SEGUNDO CATEGORIA

FONTE: BRASIL (2003)

NOTA: Destaque à categoria em que se enquadram as amostras avaliadas neste trabalho. * Pode ser necessário, dependendo da natureza do teste específico; **Se houver comprovação da reprodutibilidade não é necessária a comprovação da Precisão.

Basicamente os critérios adotados pela ANVISA seguem parâmetros internacionais estabelecidos pela conferência internacional de harmonização (ICH) e hoje seguidos pelos Estados Unidos da América, União Européia e Japão (ICH, 2005).

3.5.1 Seletividade / Especificidade

Por definição um método analítico é dito específico quando ele apenas mensura o analito desejado sem nenhuma forma de interferência, o que é muito raro. A União Internacional de Química Pura e Aplicada (IUPAC) considera que a especificidade seria a seletividade em seu grau máximo. Entretanto, é muito comum que métodos analíticos sofram interferências de uma diversidade de componentes externos presentes nas diversas matrizes que compõem as amostras. Essas interferências podem ser estimadas e, por isso, é utilizado o termo seletividade (PERSON; VESSMAN, 1998).

A seletividade de um método instrumental refere-se à capacidade que ele possui em determinar o analito de interesse com exatidão mesmo em presença dos demais componentes da amostra conhecidos como matriz da amostra. A matriz, reconhecidamente uma mistura complexa de componentes, pode incluir o placebo da formulação, intermediários de síntese, excipientes, impurezas e produtos de degradação (RIBANI *et al.*, 2004; ICH, 2005; CHANDRAN; SINGH, 2007).

Esse parâmetro pode ser atestado de diferentes maneiras conforme o guia de validação seguido.

Para métodos desenvolvidos em CLAE, a primeira forma de se avaliar a seletividade é analisando a matriz isenta da substância de interesse e a matriz adicionada com a substância padrão. Nesse caso não deve ser observada co-eluição de substâncias no tempo de retenção do analito.

Quando a matriz isenta do analito não é possível de ser obtida, como no caso da análise de drogas vegetais e seus extratos, o método da adição de padrão pode ser utilizado. Esse método consiste em preparar uma curva analítica e outra com a adição do padrão analítico à amostra e verificação do paralelismo entre as curvas obtidas. Se forem paralelas, pode-se considerar que não existe interferência da matriz na determinação do analito. Além disso, testes de pureza espectral através do detector de arranjo de diodos (DAD) auxiliam a comprovar que os picos obtidos em CLAE são puros e não apresentam substâncias co-eluindo (RIBANI *et al.*, 2004).

A analise das amostras após terem sido submetidas a diversas condições de estresse, como aquecimento entre 50 – 60 °C, luminosidade intensa, ácidos (HCl 0,1M) ou bases (NaOH 0,1M) e oxidantes (H_2O_2 3%) também podem ser utilizadas

de maneira a demonstrar que o método é capaz de seletivamente discriminar entre o analito de interesse e sustâncias de degradação (SHABIR, 2003).

3.5.2 Linearidade e intervalo

A linearidade de um método analítico é definida como a capacidade do método em fornecer resultados que são diretamente, ou por transformações matemáticas bem definidas, proporcionais à concentração do analito na amostra ao longo de uma faixa de interesse (CHANDRAN; SINGH, 2007). Segundo a ANVISA para determinações quantitativas do analito em matérias-primas ou em formas farmacêuticas o alcance da faixa deve abranger de 80 a 120% da concentração teórica da amostra e deve ser avaliada com no mínimo cinco concentrações diferentes (BRASIL, 2003).

Já intervalo é definido como a faixa de concentração da molécula alvo na amostra, incluindo os extremos, para o qual o método gera resultados precisos, exatos e lineares (USP, 2008).

A linearidade pode ser avaliada pela verificação do coeficiente angular e pelo coeficiente de correlação (r) da reta obtida para as cinco concentrações do analito. Um r maior que 0,99 é recomendado pela ANVISA e considerado como evidência de ajuste ideal dos dados para a linha de regressão (RIBANI *et al.*, 2004).

A homocedasticidade, ou variância constante dos resíduos, deve ser analisada através do gráfico de resíduos de maneira a garantir que os erros obtidos nas análises são aleatórios e não provenientes de tendências do método (CHANDRAN; SINGH, 2007).

3.5.3 Precisão

O termo precisão deve ser compreendido como a dispersão de resultados entre ensaios independentes, repetidos de uma mesma amostra, amostras semelhantes ou padrões sob condições definidas (INMETRO, 2007).

Pode ser atestada em três níveis distintos: repetitividade ou repetibilidade, precisão intermediária e reprodutibilidade.

Para a ANVISA a precisão pode ser avaliada pelo desvio padrão relativo (DPR), também conhecido como coeficiente de variação (CV) através da equação 1. Não são admitidos valores superiores a 5% (BRASIL, 2003).

$$DPR = DP / CMD \times 100 \qquad \text{(Equação 1)}$$

CMD representa a concentração média calculada.

3.5.3.1 Repetitividade ou repetibilidade

Conhecida como precisão intra-corrida pode ser definida como a concordância entre os resultados dentro de um curto período de tempo, efetuadas sob as mesmas condições, mesmo local, mesmo analista e mesmo instrumento. A ANVISA e ICH recomendam que a repetibilidade seja avaliada em um mínimo de nove determinações cobrindo o intervalo do método ou em seis determinações com uma concentração similar ao valor esperado (RIBANI *et al.*, 2004).

3.5.3.2 Precisão intermediária

Também chamada de precisão inter-corridas, é a concordância entre os resultados do mesmo laboratório, obtidos em dias diferentes, com analistas diferentes e/ou equipamentos diferentes (BRASIL, 2003). É reconhecido como o teste de maior valor para estabelecer a variabilidade dos resultados dentro de um mesmo laboratório e pode ser avaliada conforme condições descritas para repetibilidade (RIBANI *et al.*, 2004).

3.5.3.3 Reprodutibilidade

Parâmetro a ser testado quando do interesse em tornar o método oficial como, por exemplo, em farmacopéias. Avalia o grau de concordância entre os resultados obtidos por diferentes laboratórios. Para este fim é utilizada a mesma amostra em todos os laboratórios que visam à padronização. A IUPAC, recomenda que os resultados de oito laboratórios sejam avaliados para se tirar conclusões confiáveis sobre a reprodutibilidade do método (RIBANI *et al.*, 2004).

3.5.4 Limite de Detecção e Limite de Quantificação

Limite de detecção (LD) é a menor concentração do analito presente na amostra que pode ser detectada, mas não necessariamente quantificado. Já limite de quantificação (LQ) é a menor concentração do analito presente na amostra que pode ser determinado com precisão e exatidão aceitáveis. Em ambos os casos o resultado é usualmente expresso como a concentração do analito na amostra (BRASIL, 2003; ICH, 2005).

Usualmente são utilizados três procedimentos distintos para atestar os parâmetros acima descritos: método visual, método da relação sinal ruído e método baseado em parâmetros da curva analítica.

O método visual consiste em adicionar concentrações conhecidas da substância de interesse à matriz e verificar em qual concentração é possível a distinção entre o sinal analítico e o ruído naturalmente gerado pelo equipamento (RIBANI *et al.*, 2004).

A relação sinal ruído somente pode ser utilizada em métodos que apresentem o ruído da linha de base, a concentração do analito que gere uma reposta do método três vezes superior ao ruído é considerada o LD. Já o LQ é considerado a concentração do analito que gere resposta dez vezes superior ao ruído do método (SHABIR, 2003).

Finalmente o método baseado em parâmetros da curva analítica utiliza as relações descritas nas equações 2 e 3.

$$LD = 3,3 \times DP / IC \quad \text{(Equação 2)}$$

$$LQ = 10 \times DP / IC \quad \text{(Equação 3)}$$

O desvio padrão (DP) da resposta pode ser originário da estimativa do desvio padrão do branco, da equação da linha de regressão ou do coeficiente linear da equação, já IC é o coeficiente angular da curva analítica (RIBANI *et al.*, 2004).

Como por definição LQ representa a menor concentração do analito em que o método responde com precisão e exatidão é necessário que, os limites calculados por estes métodos citados sejam validados através da análise de amostras em

concentrações próximas ao valor encontrado e assim seja estabelecida a dispersão e assertividade dos resultados no LQ (ERMER, 2001).

3.5.5 Exatidão

Exatidão de um método analítico expressa a proximidade da concordância entre um valor tido como verdadeiro e o valor encontrado na análise. O ICH e a ANVISA determinam que este parâmetro seja avaliado com um mínimo de nove determinações, envolvendo um mínimo de três diferentes níveis de concentração (BRASIL, 2003; ICH, 2005).

Este parâmetro é calculado pela porcentagem de recuperação de uma quantidade conhecida do analito adicionado à amostra, conforme Equação 4, ou como a diferença entre a média e o valor aceito como verdadeiro (USP, 2008).

$$\% \text{ de recuperação} = \frac{\text{Concentração experimental média}}{\text{Concentração teórica}} \times 100 \quad \text{(Equação 4)}$$

Entre os métodos utilizados para avaliação da exatidão são descritos basicamente quatro procedimentos, a serem utilizados de acordo com a finalidade da metodologia, características da amostra e disponibilidade dos padrões analíticos.

O primeiro deles seria determinação da exatidão pela utilização de um único material de referência certificado, neste método o valor obtido na análise é comparado com aquele tido como referência (CHANDRAN; SINGH, 2007).

O segundo procedimento é a comparação entre resultados obtidos empregando-se o método em desenvolvimento e os resultados conseguidos através de um método de referência, com incerteza e precisão conhecidas, e avaliação do grau de proximidade entre os resultados obtidos pelos dois métodos (RIBANI *et al.*, 2004).

Os dois procedimentos anteriormente descritos não são aplicáveis se materiais de referência certificados ou método de referência não existirem. Nesta situação pode ser executado um ensaio de recuperação, no qual quantidades conhecidas do analito são inseridas “spiked” na matriz cobrindo toda a faixa da linearidade, seguida pela quantificação empregando-se o método extrativo e técnica

de medida propostos. O resultado então é expresso como porcentagem de recuperação (CHANDRAN; SINGH, 2007).

A adição de padrão é o quarto método utilizado para determinação da exatidão. Este procedimento é adotado quando não existe possibilidade de se preparar um branco da matriz, como no caso da análise de drogas vegetais e suas formas extrativas. Neste método, quantidades conhecidas da substância de interesse são adicionadas em diferentes concentrações na amostra que já contenha o analito em concentração desconhecida. A análise é realizada e são construídas duas curvas analíticas, FIGURA 9. Uma curva do padrão e outra com o padrão adicionado à amostra em que se relaciona as quantidades adicionadas do analito à amostra *versus* as respostas obtidas. O ponto da reta de padrão mais amostra que corta o eixo das ordenadas, corresponde à resposta da substância que está sendo determinada sem qualquer adição do padrão e a extrapolação da reta no eixo das abscissas a concentração da substância na amostra analisada. Este resultado pode então ser utilizado para avaliar a recuperação do método (RIBANI *et al.*, 2004).

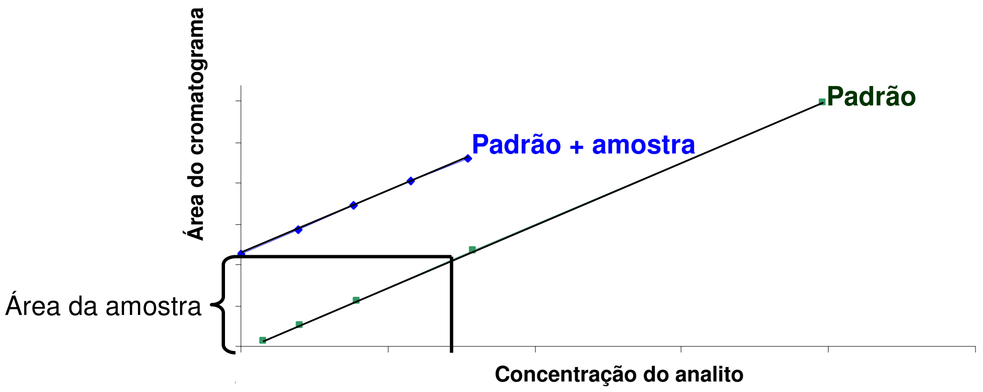

FIGURA 9 – DETERMINAÇÃO DA EXATIDÃO PELA ADIÇÃO DE PADRÃO À AMOSTRA

Importante ressaltar que as normas nacionais e internacionais não estabelecem parâmetros de aceitação para os valores de recuperação obtidos na avaliação da exatidão (THOMPSON; ELLISON; WOOD, 2002; BRASIL, 2003; ICH, 2005; INMETRO, 2007; USP, 2008). Já o autor Huber (2001) cita que a recuperação esperada depende da matriz da amostra, do preparo de amostra e da concentração

do analito. O referido autor apresenta tabela estabelecendo critérios de aceitação em função da concentração do analito na amostra, TABELA 1.

TABELA 1 – LIMITES DE ACEITAÇÃO PARA AVALIAÇÃO DA EXATIDÃO

CONCENTRAÇÃO DO ANALITO (%)	FAIXA DO ANALITO	UNIDADE	RECUPERAÇÃO MÉDIA (%)
100	1	100%	98-102
≥10	10^{-1}	10%	98-102
≥1	10^{-2}	1%	97-103
≥0,1	10^{-3}	0,1%	95-105
0,01	10^{-4}	100 ppm	90-107
0,001	10^{-5}	10 ppm	80-110
0,0001	10^{-6}	1 ppm	80-110
0,00001	10^{-7}	100 ppb	80-110
0,000001	10^{-8}	10 ppb	60-115
0,0000001	10^{-9}	1 ppb	40-120

FONTE: HUBER (2001)

NOTA: ppb – partes por bilhão

Consequentemente, os limites para as amostras utilizadas no presente trabalho seriam:

Cascas – 95 – 105%, concentração de mitrafilina entre 0,2 e 0,6%;

Tintura – 90 – 107%, concentração de mitrafilina entre 115 e 180 $\mu g.mL^{-1}$ (115 e 180 ppm);

Gel – 80 – 110%, concentração de alcalóides entre 28 e 45 $\mu g.g^{-1}$ (28 e 45 ppm).

3.5.6 Robustez

Este parâmetro avalia a capacidade do método analítico em resistir a pequenas e deliberadas variações dos parâmetros analíticos, indica sua confiança durante o uso normal. A robustez deve ser testada geralmente durante o desenvolvimento do método e quando constatada a susceptibilidade do método à variações nas condições analíticas, estas deverão ser controladas e precauções incluídas no procedimento (BRASIL, 2003).

As mudanças deliberadas devem refletir as alterações que podem ocorrer quando um método é transferido para outros laboratórios, analistas ou equipamentos e o método é dito ser robusto quando não é afetado por estas pequenas modificações (RIBANI *et al.*, 2004).

TIPO DE MÉTODO	CONDIÇÕES PASSÍVEIS DE SEREM ALTERADAS
Preparo das amostras	Estabilidade das soluções analíticas
	Tempo de extração
Espectrofotometria	Variação do pH da solução
	Temperatura
	Diferentes fabricantes de solventes
Cromatografia líquida	Variação do pH da fase móvel
	Variação na composição da fase móvel
	Diferentes lotes ou fabricantes de colunas
	Temperatura
	Fluxo da fase móvel
Cromatografia gasosa	Diferentes lotes ou fabricantes de colunas
	Temperatura
	Velocidade do gás de arraste

QUADRO 7 – MODIFICAÇÕES POSSÍVEIS PARA AVALIAÇÃO DA ROBUSTEZ
FONTE: BRASIL (2003)

O Instituto Nacional de Metrologia, Normalização e Qualidade Industrial (INMETRO) é a única agência reguladora que determina como executar esse teste através do teste de Youden. O teste de Youden permite avaliar a robustez do método bem como ordenar a influência que cada variação em suas condições analíticas causa nos resultados finais, indicando o tipo de influência de cada uma das variações (RIBANI *et al.*, 2004).

3.5.7 Estabilidade das soluções

Este item não é contemplado pela RE 899 da ANVISA e pelo ICH, no entanto para gerar resultados confiáveis e reprodutíveis, as amostras , os padrões e reagentes usados devem ser estáveis por um período razoável que depende da necessidade, um dia, uma semana, um mês ou mais. Este parâmetro deve ser avaliado durante as etapas iniciais dos estudos de validação para orientação das demais etapas (RIBANI *et al.*, 2004; CHANDRAN; SINGH, 2007).

As amostras e padrões devem ser testadas ao menos por um período 24 horas e a quantificação dos componentes deve ser realizada frente a padrões recentemente preparados. Uma estabilidade aceitável é uma mudança de 2% na resposta do padrão ou amostra. A fase móvel deve ter estabilidade aceitável, avaliada pela obtenção de cromatogramas equivalentes e resultados dentro de 2% de variação quando comparada a fase móvel velha em relação à fase móvel recém preparada (SHABIR, 2003).

3.5.8 Conformidade do Sistema

Segundo Ribani e colaboradores (2004), antes de iniciar a validação ou mesmo a análise de amostras, deve-se avaliar se o sistema utilizado é capaz de fornecer dados de qualidade aceitável. Esta avaliação é chamada conformidade do sistema (system suitability), que pode ser definida como um conjunto de testes para garantir que o equipamento utilizado está apto a gerar resultados de precisão e exatidão aceitáveis. Existem duas abordagens para comprovação da conformidade do sistema.

A primeira abordagem contempla critérios selecionados do desempenho do método determinados durante a validação. Segundo a USP (2008) os critérios estabelecidos devem ser atingidos durante as análises de rotina de maneira a ser garantida a confiabilidade dos resultados gerados, QUADRO 8.

PARÂMETRO	CRITÉRIO
Fator de retenção (k)	O pico deve estar bem separado de outros picos e do pico correspondente ao tempo de retenção de um composto não retido, $k > 2$
Repetitividade	DPR < 1% para $n > 5$
Resolução (Rs)	$Rs > 2$ entre o pico de interesse e o interferente potencial mais próximo (impureza, produto de degradação)
Fator de alargamento (TF)	$TF \leq 2$
Número de pratos teóricos da coluna (N)	$N > 2000$ para CLAE

QUADRO 8 – PARÂMETROS DE CONFORMIDADE DO SISTEMA PARA CLAE

FONTE: USP (2008)

A outra abordagem considera o sistema cromatográfico como um todo e inclui, além do sistema cromatográfico, a calibração e manutenção dos equipamentos e instrumentos utilizados em todo o procedimento analítico (RIBANI *et al.*, 2004).

4 MATERIAIS E MÉTODOS

4.1 DESENHO EXPERIMENTAL

A pesquisa seguiu cinco etapas principais: 1) isolamento e caracterização do padrão analítico; 2) desenvolvimento do método em CLAE para cascas e tintura de *U. tomentosa*; 3) otimização do preparo de amostra cascas; 4) desenvolvimento de método CLAE para o gel e 5) desenvolvimento do preparo de amostra do gel.

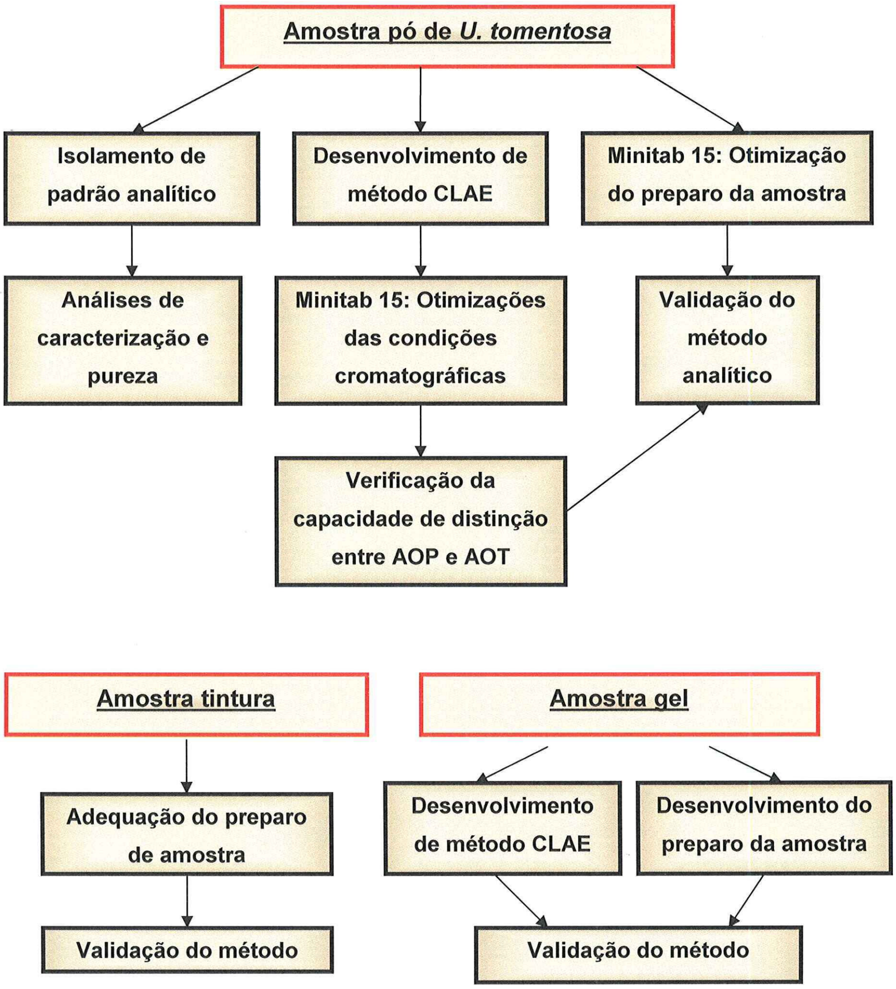

FIGURA 10 – FLUXOGRAMA DAS ETAPAS SEGUIDAS NA PESQUISA

4.2 REAGENTES, SOLVENTES E SOLUÇÕES

Os reagentes utilizados foram todos grau analítico (PA): acetato de etila, acetato de amônio, ácido clorídrico, ácido fórmico, ácido sulfúrico, éter metil *tert*-butílico (MtBE), clorofórmio, etanol, éter etílico, fosfato de potássio monobásico, fosfato de sódio dibásico, hexano, hidróxido de amônio, hidróxido de sódio, metanol e peróxido de hidrogênio.

Quando utilizados como fase móvel nas análises cromatográficas os solventes atenderam aos requisitos de grau CLAE: acetonitrila (MeCN), ácido acético, água ultra pura, metanol e trietilamina.

Para análise de RMN foi utilizado clorofórmio deuterado 99,96% D, contendo tetrametilsilano (TMS).

A poliamida utilizada na purificação das amostras para análise em CLAE foi grau cromatográfico.

Os suportes testados no desenvolvimento da metodologia para o gel possuíam as seguintes especificações:

Óxido de alumínio 90 neutro (OALN) para cromatografia, partículas de 0,05 a 0,2 mm (70 a 270 mesh).

Óxido de alumínio 90 básico (OALB) para cromatografia, partículas de 0,05 a 0,2 mm (70 a 270 mesh).

4.3 PADRÕES ANALÍTICOS

A Mitrafilina utilizada como padrão analítico de referência foi obtida através de isolamento a partir do material vegetal.

Os padrões de Isomitrafilina, Pteropodina e Isopteropodina foram adquiridos da empresa Chromadex.

Isomitrafilina, lote: 09417-101; pureza: 80,5%.

Pteropodina, lote: 21220-101; pureza: 99,9%.

Isopteropodina, lote: 21225-101; pureza: 98,6%.

4.4 EQUIPAMENTOS

- Balança analítica Mettler Toledo® modelo AB204-S (0,1 mg).
- Balança analítica AND® modelo HR-202 (0,01 mg).
- Rota evaporador Fisatom® modelo 803.
- Manta de aquecimento Fisatom® modelo 102.
- Dessecador Arsec® modelo DCV 040.
- Aparelho de ultrassom Unique® modelo USC 5000A, 40 Khz.
- Potenciômetro Tecnal® modelo TEC-3MP.
- Centrífuga Eppendorf® modelo 5416.
- Espectrofotômetro UV-VIS Shimadzu® modelo UV-1601.
- Cromatógrafo líquido de alta eficiência Agilent® 1100, degasser (G1379A), bomba quaternária (G1311A), injetor automático (G1313A), compartimento de coluna termostatizado (G1316), detector de comprimento de onda variável UV-VIS (G1314A), detector de arranjo de diodos (G1315B) (G1315B). O equipamento foi controlado pelo software Chemistation (Rev. A. 06.0x).
- Ultra-purificador de água Milli-Q®, modelo BOKN32002.C.
- Espectrômetro de massas Applied Biosystems®/MSD SCIEX, modelo API 3000 equipado com fonte de ionização do tipo “eletrospray”.
- Espectrômetro de RMN Bruker® modelo AC 200, 4,7 Tesla (200 MHz), equipado com sonda dual (^{1}H e ^{13}C) de observação direta de 5 mm.
- Sistema de Calorimetria Exploratória Diferencial Shimadzu® modelo DSC 60.

4.5 AMOSTRAS

Para todos os ensaios apresentados nesta dissertação foram utilizados pó das cascas do caule de *Uncaria tomentosa* (Willdenow ex Roemer and Shultes) DC., família Rubiaceae. As amostras foram coletadas na região de Porto Walter, estado do Acre (Brasil) e adquiridas da empresa Centroflora com concentração de 1,0% de

AOP. O material foi identificado pelo Instituto de Botânica de São Paulo onde está depositada exsicata sob número SP373337 (ANEXO 1).

Para o ensaio de verificação da capacidade do método analítico em distinguir entre AOP e AOT foi utilizado, além do material anterior, o pó das cascas do caule de *Uncaria tomentosa* (Willdenow ex Roemer and Shultes) DC., família Rubiaceae coletada no Peru e adquirida da empresa Peruvian Heritage. O material foi identificado pelo Museu de Historia Natural da Universidade Nacional Maior de San Marcos onde está depositada exsicata sob número 047-USM-2008 (ANEXO 2).

Foi utilizada tintura 10% em etanol 60% (v/v), produzida pelo Herbarium Laboratório Botânico, com concentração alcaloídica de 700 $\mu g.mL^{-1}$.

O gel utilizado foi produzido pelo Herbarium Laboratório Botânico com concentração alcaloídica de 30 $\mu g.g^{-1}$.

4.6 ISOLAMENTO DO PADRÃO ANALÍTICO DE ALCALÓIDE OXINDÓLICO

As cascas reduzidas a pó foram extraídas conforme metodologia previamente descrita por Phillipson (1973) e Herath (1979), com adaptações.

O pó (300 g) foi previamente umedecido com hidróxido de amônio 10% (300 mL) e posteriormente macerado em sonicação (30 min) com acetato de etila (1500 mL).

Após o tempo de sonicação, o acetato de etila foi filtrado, concentrado e submetido à extração líquido-líquido com ácido sulfúrico 2%. O líquido extrator foi tornado alcalino com hidróxido de amônio (pH 9 a 10) e extraído com clorofórmio.

O clorofórmio foi levado à secura originando 4,3669 g da fração alcaloídica bruta (Ab).

A fração Ab foi re-suspendida em MtBE e levada a refluxo (10 min), conforme Mazzei (2004). A solução foi resfriada à temperatura ambiente e posteriormente em freezer (-18 ^{o}C). O precipitado formado foi filtrado em papel de filtro, lavado com MtBE frio e repetida a etapa de refluxo e posterior filtração.

Finalmente o material retido no filtro (1,0125 g) foi solubilizado em clorofórmio (30 mL) e cristalizado com a adição lenta de hexano (10 mL) e etapas de

aquecimento e resfriamento. O alcalóide assim obtido foi seco em dessecador com corrente de ar por 24 horas.

4.6.1 Identificação e confirmação da pureza do padrão isolado

A identificação da molécula isolada foi realizada por RMN, EM e perfil de absorção em espectrofotômetro. Para RMN a molécula alvo (30 mg) foi dissolvida em clorofórmio deuterado (2 mL) e realizadas as técnicas de ^{1}H, ^{13}C desacoplado e “distortion enhancement by polarization transfer” (DEPT).

Para EM foi realizada a injeção por bomba seringa da amostra (5 mg) dissolvida em etanol (10 mL), adicionada dos aditivos acetato de amônio 5 mM e ácido fórmico 1% e testadas as condições de fragmentação positiva e negativa. Primeiramente foi monitorado o íon molecular da amostra injeta e após confirmação da massa foram monitorados os íons produto do íon molecular encontrado.

TABELA 2 – CONDIÇÕES UTILIZADAS NO ESPECTRÔMETRO DE MASSAS

PARÂMETROS	MS1 – Q1 – 367,2 $[M-H]^+$	MS1 + Q1 – 369,3 $[M+H]^+$	MS2
Fonte de ions	electrospray	electrospray	electrospray
Temperatura da interface (^{o}C)	500	500	500
Gás nebulizador (Ar sintético)	6	6	6
Cortina de gás (N_2 - 6.0)	6	6	6
Voltagem do spray	-3000	5500	5500
Potencial de declustering	-50	50	50
Potencial de focalização	-200	200	200
Gás de colisão	N.A.	N.A.	4
Energia de colisão	N.A.	N.A.	35
Produto	N.A.	N.A.	369,4

O perfil espectral foi obtido com a amostra (2 mg) diluída no solvente etanol (100 mL) para varredura de 190 a 330 nm.

A pureza da molécula foi avaliada por calorimetria diferencial exploratória (DSC) e pureza espectral em CLAE equipado com DAD. Para DSC a amostra (2,70 mg) foi submetida à atmosfera de nitrogênio com fluxo de 100 mL/min e programação de temperatura com início em 40 ^{o}C e aumento de 20 ^{o}C/min até 350 ^{o}C.

A análise de pureza espectral em CLAE equipado com DAD foi realizada com a amostra (5 mg) dissolvida em etanol (10 mL) e 10 µL injetados no cromatógrafo conforme condições otimizadas. Todos os espectros dentro da área do pico foram obtidos pelo software entre os comprimentos de onda de 200 e 400 nm.

4.7 DESENVOLVIMENTO DO TAMPÃO ORGÂNICO E OTIMIZAÇÃO DOS MÉTODOS EM CLAE

4.7.1 Desenvolvimento do Tampão orgânico e Otimização do método em CLAE para cascas e tintura

O método CLAE descrito por Ganzera e colaboradores (2001) foi otimizado através de planejamento fatorial de experimentos realizado em pacote estatístico Minitab 15 (Minitab Inc. 2006). A avaliação dos resultados quimiométricos obtidos foi feita no mesmo programa.

Na etapa de triagem foi utilizado um delineamento fatorial 2^3, com repetições completamente randomizadas realizadas em duplicata, resultando em um total de dezesseis experimentos. Os parâmetros analisados foram: natureza do tampão, pH do tampão e temperatura de coluna.

Os parâmetros indicados como estatisticamente significativos pela análise de variância (ANOVA), intervalo de confiança 95%, foram otimizados com aplicação de design composto central. Os resultados obtidos foram utilizados na construção dos gráficos de superfície de resposta.

O método definido segue abaixo.

- Eluição: gradiente, TABELA 3
- Fase aquosa: ácido acético 0,2% em água ajustada a pH 6,90 com trietilamina (Tampão acetato)

- Fase orgânica: MeCN
- Coluna: Zorbax Eclipse XDB C-18 (150 x 4,6 mm; 3,5 µm), Agilent
- Temperatura da coluna: 15 ^{o}C
- Volume de injeção: 10 µL
- Detecção: 245 nm

TABELA 3 – GRADIENTE UTILIZADO NAS ANÁLISES POR CLAE DAS CASCAS E TINTURA

TEMPO (min)	FASE AQUOSA (%)	FASE ORGÂNICA (%)	FLUXO (mL/min)
0	65	35	0,80
18	65	35	0,80
29	50	50	0,80
31	50	50	0,80
32	65	35	0,80
38	65	35	0,80

4.7.2 Verificação da capacidade de distinção entre AOT e AOP

Para verificar a capacidade do método em distinguir entre AOT e AOP, o método cromatográfico definido foi transposto para cromatógrafo líquido de alta eficiência acoplado a detector de espectrometria de massas.

Para este teste foram utilizadas amostras das cascas da planta adquirida no Peru e no Brasil, preparadas conforme condições definidas após otimização do método extrativo.

Em ambas as amostras foi realizada análise preliminar em MS1 pela detecção de todos os íons presentes na amostra com relação *m/z* entre 150 e 400 Da. As amostras foram posteriormente submetidas à análise MS2 pelo monitoramento dos íons produto originários de $[M+H]^+$ 369,00 para AOP e $[M+H]^+$ 385,00 para AOT. As condições do equipamento foram as mesmas do modo MS2 já descrito (item 4.6.1).

4.7.3 Escolha do padrão externo para quantificação dos AO

Foram avaliados os padrões: mitrafilina, isomitrafilina, pteropodina e isopteropodina.

Para cada padrão em separado foi construída uma curva analítica com diluição em etanol nas concentrações 8,0; 20,0; 40,0; 83,0 e 210,0 $\mu g.mL^{-1}$.

Primeiramente, amostras das cascas (três) foram analisadas e todos os AO obtidos nos cromatogramas foram integrados e calculados considerando uma única curva analítica, esse procedimento foi realizado de maneira que cada amostra apresentasse quatro resultados, um para cada curva. Posteriormente foi obtido o valor considerado verdadeiro para cada amostra, calculando cada alcalóide das amostras frente à curva analítica obtida de seu respectivo padrão analítico. Como não existiam padrões de especiofilina e uncarina F, esses alcalóides foram calculados com referência nas curvas de isopteropodina e mitrafilina respectivamente.

As médias dos resultados obtidos para cada curva em separado e para cada alcalóide com sua curva (valor verdadeiro) foram comparadas por ANOVA com execução do teste de Tukey, limite confiança de 95%.

4.7.4 Estabilidade das soluções padrão

As soluções utilizadas para construção da curva analítica da mitrafilina, nas concentrações apresentadas no item anterior, foram armazenadas em freezer a -20 ^{o}C. Cada uma das soluções foi analisada em triplicata nos tempos 0, 15, 30, 60, 90 e 120 dias.

As curvas obtidas nos tempos 15, 30, 60, 90 e 120 dias foram comparadas à curva obtida no tempo zero através da execução do teste t para amostras pareadas.

4.7.5 Otimização do método CLAE para análise do gel

O método CLAE foi definido após adequação do solvente orgânico e composição do gradiente, TABELA 4. Foi escolhida a mistura MeCN : metanol (2:1) em gradiente com o tampão orgânico desenvolvido. As demais condições cromatográficas foram as mesmas do método para casca e tintura (item 4.7.1).

TABELA 4 – GRADIENTE UTILIZADO NAS ANÁLISES POR CLAE DO GEL

TEMPO (min)	FASE AQUOSA (%)	FASE ORGÂNICA (%)	FLUXO (mL/min)
0	90	10	0,80
2	90	10	0,80
3	70	30	0,80
12	60	40	0,80
35	50	50	0,80
42	40	60	0,80
44	30	70	0,80
47	30	70	0,80
49	90	10	0,80
55	90	10	0,80

4.7.6 Avaliação da conformidade do sistema

A conformidade dos sistemas CLAE desenvolvidos foi avaliada por cinco injeções da mesma amostra casca e gel nos seus respectivos métodos cromatográficos. A avaliação foi realizada pela obtenção de resolução maior que 2,0 entre o par problema pteropodina – isomitrafilina em ambos os métodos. Além disso, desvio padrão relativo inferior a 1% para as áreas de cada um dos alcalóides e simetria de pico inferior a dois foram considerados para confirmação de conformidade do sistema (USP, 2008).

4.8 DESENVOLVIMENTO E OTIMIZAÇÃO DOS MÉTODOS DE PREPARO DAS AMOSTRAS CASCAS, TINTURA E GEL

4.8.1 Otimização do preparo da amostra cascas de *U. tomentosa*

A otimização da extração das cascas de *U. tomentosa* foi realizada a partir do método proposto por Ganzera e colaboradores (2001).

Para o desenvolvimento diferentes parâmetros considerados críticos na etapa: tempo de extração, tipo de solvente extrator, tamanho da partícula da amostra, temperatura do banho de extração e massa da amostra submetida à extração, foram avaliados através do pacote estatístico Minitab 15 (Minitab Inc. 2006).

Os parâmetros descritos foram avaliados em etapa de triagem através da execução de delineamento fatorial 2^5, com repetições completamente randomizadas realizadas em duplicata, resultando em um total de 64 experimentos.

Os parâmetros críticos indicados por ANOVA, intervalo de confiança 95%, foram otimizados pela aplicação de design composto central. Abaixo segue o método otimizado.

Amostra (200 mg) em balão volumétrico de 10 mL, diluição com solvente etanol 60% (v/v). Extração em banho de ultrassom (20 min) a 30 ^{o}C. Após a extração o sobrenadante (2 mL) foi passado por coluna contendo poliamida (200 mg) e o eluato analisado em CLAE.

4.8.2 Otimização do preparo da amostra tintura de *U. tomentosa*

O preparo de amostra foi definido pela transferência da tintura (2 mL) para balão volumétrico de 5 mL e adição de etanol 60% (v/v) ao volume. A diluição (2 mL) transferida para coluna com poliamida (200 mg) e o eluato levado ao cromatógrafo.

4.8.3 Otimização do preparo da amostra gel

Para otimização do método foram testados diferentes sorbentes, proporções entre o sorbente e amostra, recipientes para mistura da amostra com sorbente, solventes de limpeza e remoção dos alcalóides. O método foi definido conforme FIGURA 11.

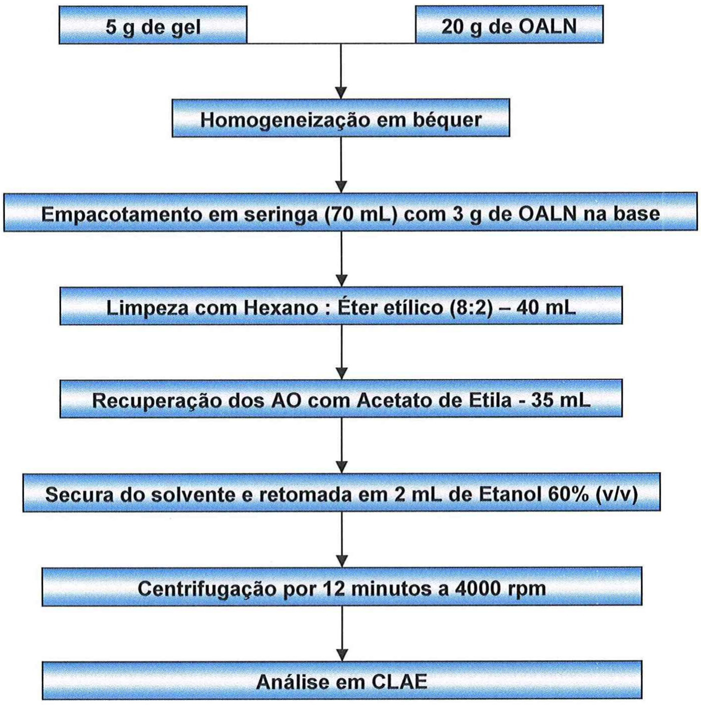

FIGURA 11 – FLUXOGRAMA DO PREPARO DA AMOSTRA GEL

4.9 VALIDAÇÃO DOS METODOS ANALÍTICOS

Conforme descrito no item 3.5, os métodos analíticos do presente trabalho podem ser classificados na categoria 1 da RE 899 da ANVISA (2003). Além dos parâmetros exigidos os limites de detecção e quantificação foram avaliados como complementação.

4.9.1 Seletividade / especificidade

4.9.1.1 Método da adição de padrão

Este procedimento foi utilizado para as cascas e tintura. O preparo das amostras e padrão está descrito na exatidão do método. Foi verificado o paralelismo entre as curvas obtidas com padrão e amostra + padrão, através da comparação dos respectivos coeficientes angulares.

4.9.1.2 Comparação dos perfis espectrais

Os picos referentes aos AO tiveram seus perfis espectrais (200 a 400 nm) comparados aos perfis dos padrões de mitrafilina, isomitrafilina, pteropodina e isopteropodina, através de DAD.

4.9.1.3 Avaliação da pureza de pico

Os picos dos alcalóides foram submetidos à análise de pureza espectral em DAD. As purezas dos espectros analisados ao longo de todo o pico foram comparadas às obtidas com os padrões de mitrafilina, isomitrafilina, pteropodina e isopteropodina. Além disso, foram testadas amostras submetidas à degradação (SHABIR, 2003):

Ácida – amostras foram adicionadas de quantidades de HCl para perfazer a concentração de 0,1 M na solução final de leitura;

Alcalina – amostras foram adicionadas de quantidades de NaOH 1,0 M para perfazer a concentração de 0,1 M na solução final de leitura;

Peróxido – foi utilizado peróxido de hidrogênio 3% (2 mL) no preparo das amostras.

4.9.1.4 Monitoramento dos íons produtos originários de $[M+H]^+$ = 369 e 385 Da

Realizado apenas para as cascas da planta. As condições foram as mesmas das descritas na TABELA 2 (item 4.6.1), a amostra teve monitorados os íons produto de $[M+H]^+$ = 369,00 e 385,00 Da.

4.9.1.5 Análise do placebo

Teste realizado apenas para o gel. A formulação contendo todos os componentes da amostra à exceção da tintura de *U. tomentosa* (placebo), foi submetida à análise conforme procedimento descrito (item 4.8.3) e submetido à análise cromatográfica (item 4.7.5).

4.9.2 Linearidade e intervalo

Para determinação da linearidade com o padrão analítico foram avaliadas três curvas analíticas, cinco pontos cada em um total de 15 injeções, com concentrações entre 8,0 e 210,0 $\mu g.mL^{-1}$ para os dois métodos cromatográficos desenvolvidos.

A linearidade com amostra das cascas da planta foi testada com pesagens de 100, 150, 200, 250 e 300 mg de amostra abrangendo a faixa de 50 a 150% do teor da casca.

Volumes de amostra de 1,6; 1,8; 2,0; 2,2 e 2,4 mL foram tomados de maneira a abranger a faixa de 80 a 120% do teor da tintura.

Para o gel foram utilizadas amostras preparadas com quantidades proporcionais de tintura de maneira a perfazer as concentrações 80 a 120% do teor médio especificado para o produto. Em todos os testes de linearidade com amostra cada concentração foi analisada com uma triplicata. Todas as curvas foram plotadas no programa Microsoft Excel®, através da correlação da concentração final de AO presente no volume de injeção ($\mu g.mL^{-1}$) versus a área somada dos alcalóides ($mAU.s^{-1}$). Pelo software foram obtidos os coeficientes angular e linear, além do coeficiente de correlação, coeficiente de determinação e gráfico de resíduos.

O intervalo de aplicação foi avaliado pelas faixas testadas na linearidade, exatidão e precisão para cada uma das metodologias.

4.9.3 Precisão

4.9.3.1 Repetitividade ou repetibilidade

Foi avaliada em cada um dos métodos pela análise de seis amostras diferentes representando a concentração 100%, executadas por um mesmo analista, com o mesmo equipamento em um curto período de tempo. Além disso, foram utilizadas as triplicatas de cada concentração mínima e máxima do teste de linearidade com amostra para confirmar a repetibilidade nos teores 50 e 150% para cascas e 80 e 120% para tintura e gel. Os resultados foram avaliados em função do DP e DPR.

4.9.3.2 Precisão intermediária

Atestada pela comparação dos resultados das seis amostras a 100% ensaiadas na repetibilidade, com outras seis amostras analisadas por outro analista, no mesmo equipamento e em dia diferente. Os resultados foram avaliados em função do DP e DPR.

4.9.4 Limite de detecção

Os dados de inclinação e desvio obtidos com a curva analítica dos dois métodos cromatográficos foram utilizados na Equação 2, item 3.5.4, para se obter o valor de LD para análise em CLAE das cascas, tintura e gel. Posteriormente foram realizadas diluições do padrão até alcançar o limite indicado, a análise em CLAE desta concentração foi utilizada como confirmação do resultado.

4.9.5 Limite de quantificação

Os dados de inclinação e desvio obtidos com a curva analítica dos dois métodos cromatográficos foram inseridos na Equação 3, item 3.5.4, de maneira a ser gerado o LQ para cada um dos métodos. Posteriormente foram realizadas diluições do padrão até alcançar o limite indicado, a análise em CLAE desta solução foi utilizada como um indicativo da confiabilidade do resultado. A confirmação da precisão e exatidão no limite encontrado foi realizada pela análise de seis replicatas com tomadas de amostra que fornecessem concentrações próximas às calculadas como LQ com o padrão.

cascas: 90 mg foram analisados conforme o procedimento do item 4.8.1;

tintura: 0,10 mL da tintura foi analisada conforme procedimento do item 4.8.2;

gel: 1,1 g de gel foram analisados conforme procedimento do item 4.8.3.

4.9.6 Exatidão

Para as amostras de casca e tintura foi utilizado o método de adição de padrão à amostra, devido à impossibilidade de se obter branco da matriz da amostra (SHABIR, 2003; RIBANI *et al.*, 2004).

A curva analítica foi construída nas concentrações 8,0; 20,0; 40,0; 83,0 e 210,0 $\mu g.mL^{-1}$, a equação da reta dessa curva foi utilizada para inserção do valor do coeficiente linear obtido da curva da amostra adicionada de quantidades crescentes de padrão, isso permitiu encontrar o valor real do analito na amostra.

As curvas de amostra adicionada de padrão foram construídas com 5 pontos cada, pela contraposição da concentração final do analito adicionado ($\mu g.mL^{-1}$) versus área do pico (mAU x s).

Casca de *U. tomentosa*: à tomada de amostra inicial (200 mg) foram adicionados 1, 2, 3 e 4 mL de solução 210,0 $\mu g.mL^{-1}$ do padrão mitrafilina,

Tintura de *U. tomentosa:* à tomada de amostra inicial (2 mL) foram adicionados 0,5, 1, 2 e 3 mL de solução padrão mitrafilina com a mesma concentração anterior.

O valor verdadeiro do analito presente na amostra foi utilizado para calcular a recuperação (Equação 4, item 3.5.5) de amostras preparadas conforme indicado no teste de linearidade (item 4.9.2) nas concentrações 50, 100 e 150% para o pó e 80, 100 e 120% para a tintura.

O teste do gel foi executado com placebo (5 g) contaminado por quantidades de mitrafilina de maneira a perfazer as concentrações 80, 100 e 120% de AO na amostra. A avaliação da recuperação da mitrafilina foi utilizada para indicar a exatidão do método.

4.9.7 Robustez

Para as cascas foram considerados os dados obtidos durante o planejamento fatorial de experimentos realizado para otimização do método cromatográfico e preparo de amostra. Foram avaliados ainda os parâmetros estabilidade da amostra preparada após um e dois dias armazenada em freezer (-20 ^{o}C), além de colunas diferentes, QUADRO 9.

COLUNA	DIMENSÕES	CARACTERÍSTICAS	END CAPPING	FABRICANTE
Luna C-18 (2)	150 x 4,6 mm	100 Å de poro, 17,5% de Carbono, 5 µm partícula	Sim	Phenomenex
Hipersil	150 x 4,6 mm	120 Å de poro, 10% de Carbono, 5 µm partícula	Sim	Agilent
Zorbax Eclipse XDB	150 x 4,6 mm	80 Å de poro, 10% de Carbono, 5 µm partícula	Duplo	Agilent
Zorbax Eclipse XDB (controle)	150 x 4;6 mm	80 Å de poro, 10% de Carbono, 3,5 µm partícula	Duplo	Agilent

QUADRO 9 – COLUNAS TESTADAS NA VALIDAÇÃO DA ROBUSTEZ DOS MÉTODOS

A Robustez da tintura considerou os resultados obtidos em planejamento fatorial de experimentos para otimização do método CLAE e diferentes colunas avaliadas para as cascas, por se tratar de matriz semelhante à obtida após o preparo das cascas.

Estabilidade das amostras após um dia de preparo, 2,5 g de OALN na base da coluna, dispersão do gel em OALN e eluição após um dia e as colunas apresentadas no QUADRO 9 foram os parâmetros alterados na avaliação da robustez do gel.

À exceção dos parâmetros avaliados por planejamento fatorial de experimentos, os demais foram avaliados em triplicata e as médias dos resultados para os teores de AO comparados às médias de uma triplicata controle, condições normais da análise, através de ANOVA, execução do teste de Tukey com 95% de limite de confiança.

4.10 CÁLCULO DO TEOR DE ALCALÓIDES NAS AMOSTRAS

Para todas as amostras utilizadas no presente trabalho, os picos referentes aos AOP foram integrados e calculados frente à curva analítica de mitrafilina expressa em $\mu g.mL^{-1}$. Desta forma para gerar os teores finais de alcalóides para cada uma das amostras (cascas, tintura e gel) nas unidades apresentadas (%, $\mu g.mL^{-1}$ e $\mu g.g^{-1}$) foi necessário considerar as diluições efetuadas, o peso realizado para o preparo da amostra e fatores para a conversão de unidades conforme apresentado a seguir.

4.10.1 Amostra cascas

Conforme apresentado no item 4.8.1, as cascas foram diluídas a 10 mL e analisadas em CLAE, logo o resultado do cromatograma ($\mu g.mL^{-1}$) foi multiplicado por 10 para encontrar a quantidade de alcalóides no volume final de amostra. Posteriormente o resultado foi multiplicado por 100, dividido pelo peso da amostra (mg) e finalmente dividido por 1000 para apresentar o resultado em %.

4.10.2 Amostra tintura

Conforme apresentado no item 4.8.2, a amostra tintura (2 mL) foi diluída a 5 mL e analisada em CLAE. Portanto, o resultado do cromatograma ($\mu g.mL^{-1}$) foi multiplicado por 5 para encontrar a quantidade de alcalóides no volume final de amostra e finalmente dividido pela quantidade de tintura utilizada na análise (2 mL) para apresentar o resultado em $\mu g.mL^{-1}$.

4.10.3 Amostra gel

Para o preparo da amostra o item 4.8.3 apresenta o uso de 5 g de gel e após a extração a obtenção de 2 mL para análise em CLAE. Esse volume final é acrescido de 0,2 mL provenientes de componentes hidrossolúveis do gel, remanescentes do processo de limpeza do preparo da amostra, o que eleva o volume final de análise para 2,2 mL.

Assim o resultado do cromatograma ($\mu g.mL^{-1}$) foi multiplicado por 2,2 para encontrar a quantidade de alcalóides no volume final de amostra e finalmente dividido pela quantidade de gel utilizada na análise (g) para apresentar o resultado em $\mu g.g^{-1}$.

5 RESULTADOS E DISCUSSÃO

A revisão de literatura permitiu demonstrar que os métodos para análise de AO nas cascas de *U. tomentosa* são bastante difundidos na literatura científica, no entanto, em sua quase totalidade são descritas fases móveis compostas por tampão inorgânico o que exige limpeza da coluna e sistema cromatográfico após as análises, o que não realizado adequadamente reduz muito o tempo de vida da coluna. Além disso, a complexidade da matriz de análise do gel exige abordagem mais elaborada para a extração seletiva e posterior quantificação dos analitos. Estes desafios permitiram a realização do trabalho para desenvolvimento de métodos de análise para casca, tintura e produto contendo tintura de *Uncaria tomentosa.*

Para evitar repetições desnecessárias em algumas tabelas foi utilizada a seguinte numeração para designar os nomes dos alcalóides:

1 – especiofilina; 2 – uncarina F; 3 – mitrafilina; 4 – isomitrafilina; 5 – pteropodina; 6 – isopteropodina; 7 – rincofilina; 8 – isorincofilina.

5.1 ISOLAMENTO, CARACTERIZAÇÃO E CONFIRMAÇÃO DA PUREZA DO PADRÃO ANALÍTICO

Os 300 g de planta utilizados no isolamento forneceram 4,3669 g de fração Ab o que, se considerado o teor médio obtido na validação das cascas de 1,05% de alcalóides oxindólicos totais (precisão intermediária, item 5.4.4.4), reflete em rendimento de 138,63%. O valor pode ser atribuído ao grande residual de clorofórmio, solvente muito denso, que não foi possível de ser totalmente eliminado da fração.

A fração Ab após sucessivas cristalizações originou 0,6128 g de material cristalino branco. Este material analisado em CLAE apresentou pureza cromatográfica de 99,0% e tempo de retenção 15,789 min tempo atribuído à mitrafilina, FIGURA 12.

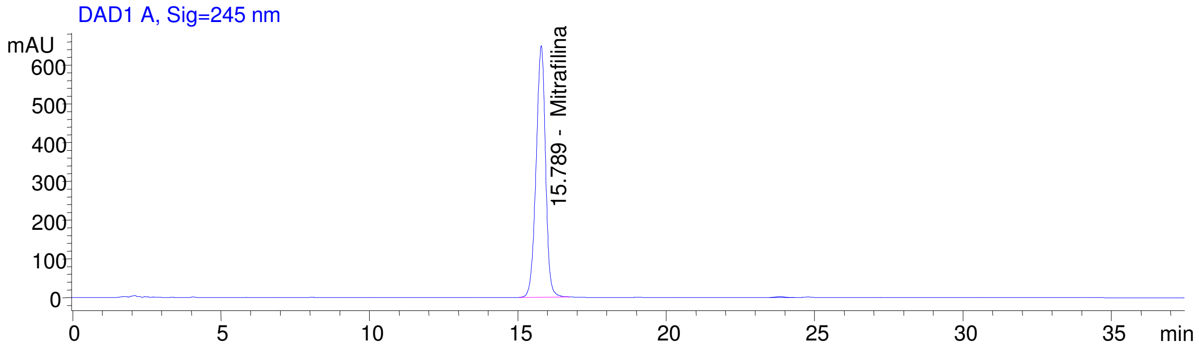

FIGURA 12 – CROMATOGRAMA DO ALCALÓIDE ISOLADO

Se for considerado que o valor médio de mitrafilina encontrado no pó durante a validação das cascas foi de 0,38% (exatidão, item 5.4.4.5), o isolamento apresenta rendimento de 53,75%.

Posteriormente o alcalóide isolado foi identificado e teve sua pureza atestada por RMN ^{1}H e ^{13}C, CLAE/DAD, EM e DSC.

5.1.1 RMN de ^{1}H e ^{13}C

Os dados obtidos nestes testes foram todos comparados aos previamente apresentados pelos grupos de pesquisa representados por Seki (1993), Toure (1992) e Carbonezi (2004). Todos os dados foram obtidos com zero em TMS.

Pela observação da estrutura da mitrafilina, FIGURA 13, pode ser depreendida a complexidade das interações H-H no espectro de ^{1}H, o que levou Seki e colaboradores (1993) com equipamento de 500 MHz e diferentes técnicas bi-dimensionais a classificar alguns sinais obtidos apenas como m (multipletos), sem tentar apresentar suas correlações.

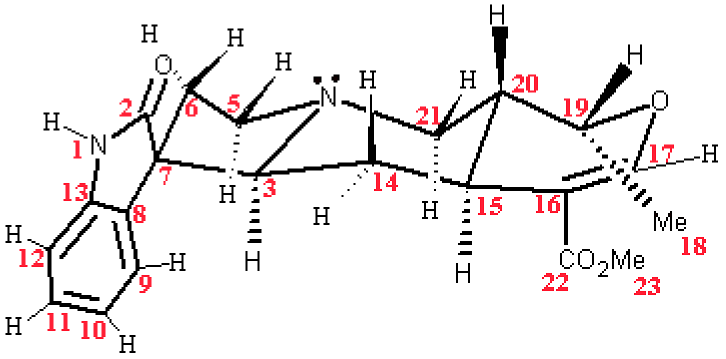

FIGURA 13 – ESTRUTURA DA MITRAFILINA

Em equipamento de 200 MHz foram observados sinais para os hidrogênios próximos aos obtidos pelo referido autor, FIGURA 14, não sendo possível comparação com os dados do trabalho de Toure e colaboradores (1992), pois os autores utilizaram como solvente DMSO-d_6. Na TABELA 5 são apresentados os deslocamentos e constantes de acoplamento dos principais H que diferenciam a mitrafilina de seu epímero em C-7, isomitrafilina. As discrepâncias observadas entre as constantes de acoplamento e deslocamentos podem ser atribuídas à dificuldade em se integrar corretamente os sinais observados em um equipamento de menor resolução.

TABELA 5 – RESULTADOS OBTIDOS PARA RMN-^{1}H DA MITRAFILINA ISOLADA

H	SEKI, *et al.* (1993)		MITRAFILINA	
	δ	CONSTANTE DE ACOPLAMENTO	δ	CONSTANTE DE ACOPLAMENTO
3	2,41	dd $J_{3\text{-}14\beta}$ = 11,1; $J_{3\text{-}14\alpha}$ = 2,4	2,40	dd $J_{3\text{-}14\beta}$ = 11,4; $J_{3\text{-}14\alpha}$ = 2,5
5 β	3,39	m	3,40	m
9	7,19	d $J_{9\text{-}10}$ = 7,8	7,20	d $J_{9\text{-}10}$ = 7,6
10	7,00	ddd $J_{10\text{-}9}$ = 7,8; $J_{10\text{-}11}$ = 7,8; $J_{10\text{-}12}$ = 0,9		ddd $J_{10\text{-}9}$ = 8,0; $J_{10\text{-}11}$ = 8,0; $J_{10\text{-}12}$ = 0,9
12	6,89	d $J_{12\text{-}11}$ = 7,6	6,90	d $J_{12\text{-}11}$ = 7,2
14 β	1,21	ddd $J_{14\beta\text{-}14\alpha}$ = 11,1; $J_{14\alpha\text{-}15}$ = 11,1; $J_{14\beta\text{-}3}$ = 11,1	1,22	d J = 11,1
17	7,43	d $J_{17\text{-}15}$ = 1,3	7,43	Sinal alargado
18 (Me)	1,11	d $J_{18\text{-}19}$ = 6,6	1,11	d $J_{18\text{-}19}$ = 6,6
19	4,37	qd $J_{19\text{-}18Me}$ = 6,6; $J_{19\text{-}20}$ = 3,2	4,37	$J_{19\text{-}18Me}$ = 6,6; $J_{19\text{-}20}$ = 2,9
21 α	1,85	dd $J_{21\alpha\text{-}21\beta}$ = 10,5; $J_{21\alpha\text{-}20}$ = 10,4	1,88	dd $J_{21\alpha\text{-}21\beta}$ = 10,4; $J_{21\alpha\text{-}20}$ = 10,3
21 β	3,22	dd $J_{21\beta\text{-}21\alpha}$ = 10,5; $J_{21\beta\text{-}20}$ = 2,2	3,23	dd $J_{21\beta\text{-}21\alpha}$ = 8,7; $J_{21\beta\text{-}20}$ = 1,0
23 (PEREIRA *et al.*)	3,57	s	3,59	s
NH	8,40	Sinal alargado	8,54	Sinal alargado

NOTA: m: multipleto

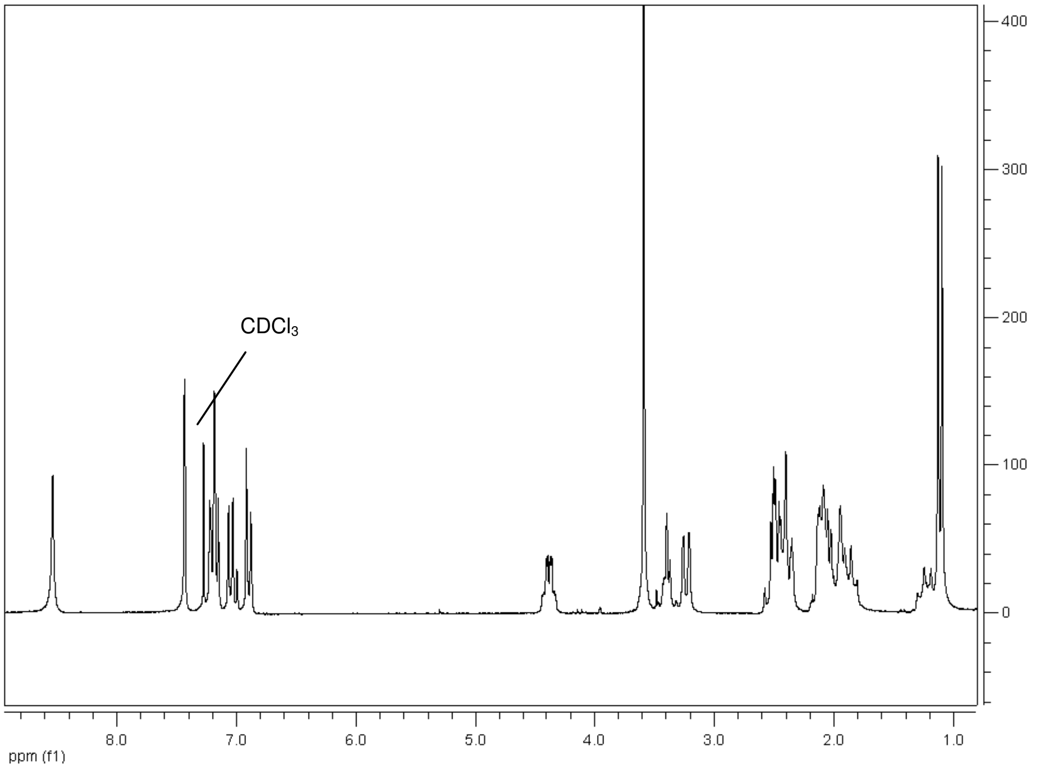

FIGURA 14 – ESPECTRO DE RMN - ^{1}H (200 MHz) EM $CDCl_3$ DA MITRAFILINA ISOLADA

Os dados obtidos para RMN-^{13}C, TABELA 6, confirmam de maneira inequívoca a identidade da molécula, todos os deslocamentos são condizentes com aqueles apresentados pelos três autores já citados.

TABELA 6 – DADOS OBTIDOS PARA RMN-^{13}C DA MITRAFILINA ISOLADA

C	SEKI, *et al.* (1993)	TOURE, *et al.* (1992)	CARBONEZI, *et al.*. (2004)	MITRAFILINA
2	181,33	179,44	180,90	181,39
3	74,58	73,62	74,60	74,60
5	54,28	52,79	54,30	54,31
6	35,16	34,35	35,10	35,18
7	55,56	54,85	55,50	55,60
8	133,35	133,65	133,30	133,35
9	122,89	122,85	123,00	122,93
10	122,54	121,40	122,60	122,59
11	128,00	127,50	128,00	128,05
12	109,74	108,69	109,50	109,80
13	140,87	141,80	140,60	140,91
14	28,37	28,01	28,30	28,39
15	30,44	29,73	30,40	30,47
16	106,92	106,74	106,90	106,93
17	154,05	153,29	154,00	154,10
18	14,84	14,53	14,80	14,88
19	73,81	73,22	73,80	73,85
20	40,48	40,23	40,50	40,50
21	54,33	53,31	54,20	54,36
22	167,10	165,94	167,10	167,14
23	50,71	50,30	50,70	50,77

As pequenas divergências entre os valores encontrados por Toure e colaboradores (1992) daqueles obtidos no presente trabalho podem ser atribuídas ao já citado emprego de DMSO-d_6 pelo referido autor.

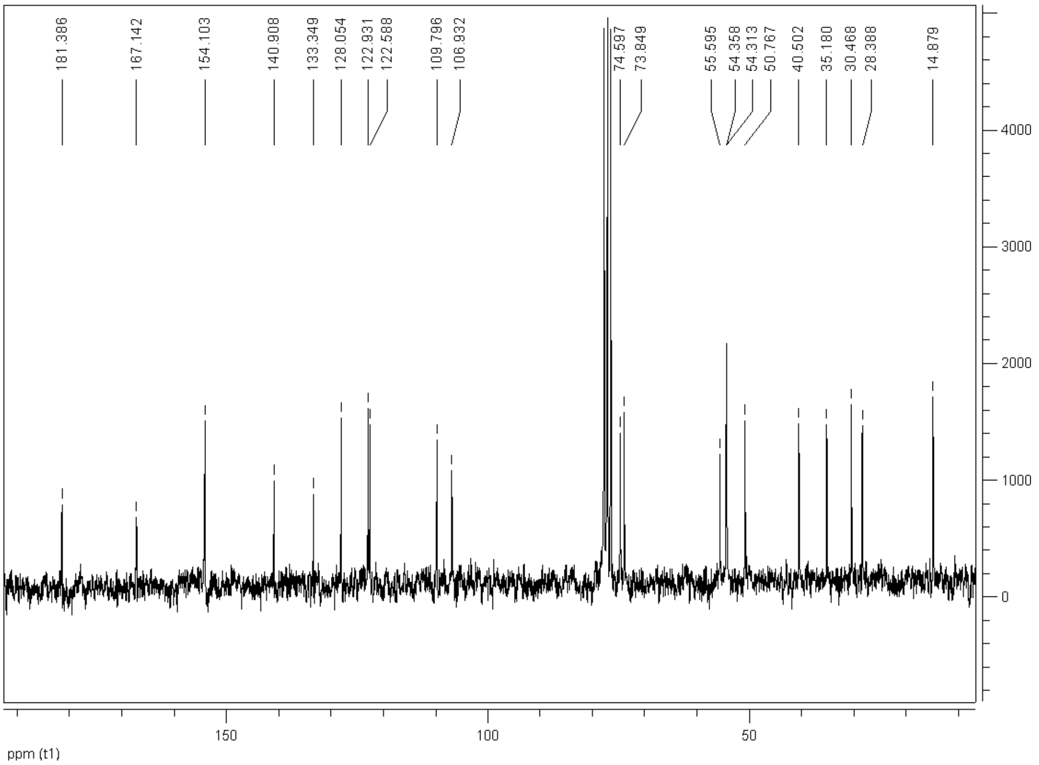

FIGURA 15 – ESPECTRO DE RMN – ^{13}C (200 MHz) EM $CDCl_3$ DA MITRAFILINA ISOLADA

O espectro obtido em DEPT, FIGURA 16, confirma a atribuição de cada carbono, uma vez que não são observados no espectro os carbonos quaternários 2, 7, 8, 13, 16 e 22. Além disso, são encontrados na fase inferior do espectro os carbonos CH_2 5, 6, 14 e 21, todos os sinais estão com seus deslocamentos em regiões muito semelhantes às já apresentadas no espectro de RMN de ^{13}C, FIGURA 15.

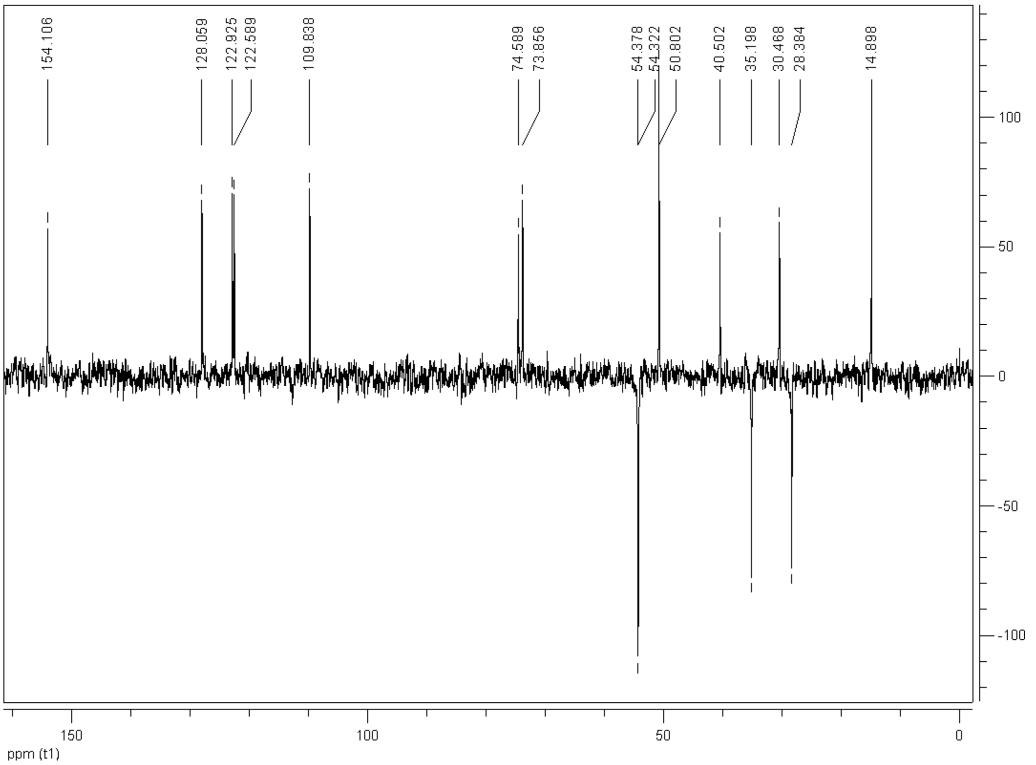

FIGURA 16 – ESPECTRO DEPT (200 MHz) EM $CDCl_3$ DA MITRAFILINA ISOLADA

5.1.2 Espectrometria de massas

A fórmula molecular da mitrafilina $C_{21}H_{24}N_2O_4$ apresenta massa de 368,4263 Da, conforme apresentado por diversos autores (PHILLIPSON; HEMINGWAY, 1975b; WAGNER; KREUTZKAMP; JURCIC, 1985; LOPEZ-AVILA; BENEDICTO, 1997; CARBONEZI *et al.*, 2004). A análise em MS1 modo positivo $[M+H]^+$ com aditivo acetato 5 mM apresentou melhores resultados em relação ao modo negativo e aditivo ácido fórmico.

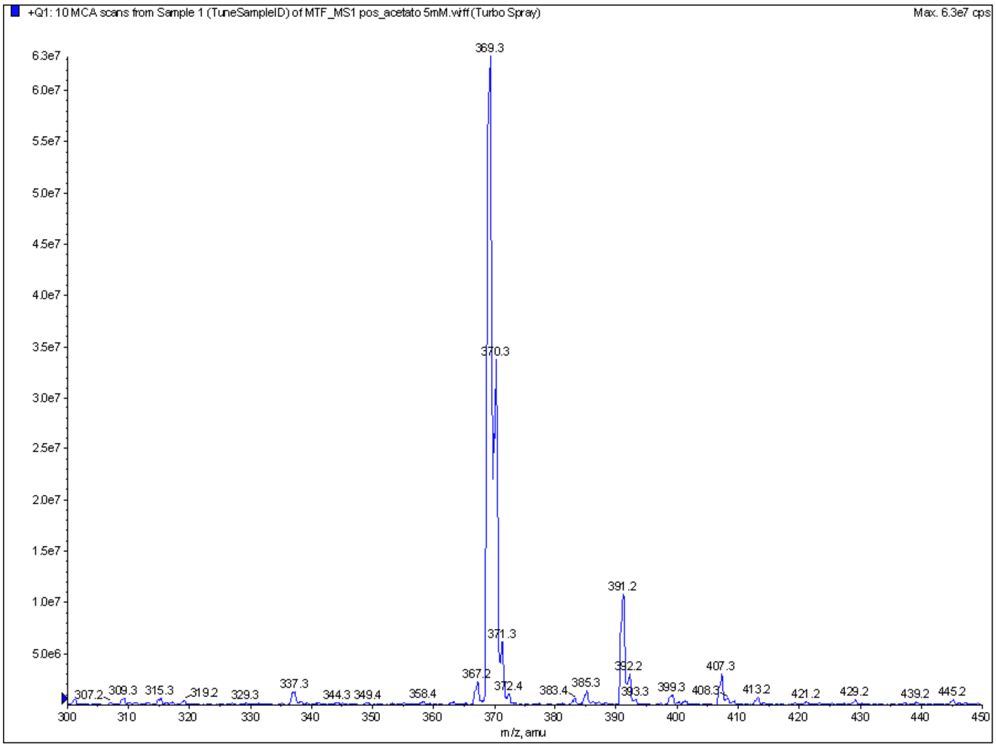

FIGURA 17 – ESPECTRO DE MASSAS DA MITRAFILINA ISOLADA - $[M+H]^+$

A elevada abundância do sinal *m/z* 369,3 Da em relação aos demais sinais indica a pureza do material isolado e confirma a massa do alcalóide investigado.

A análise em MS2 apresentou íons produto característicos de AOP e serão discutidos posteriormente (item 5.2.2).

5.1.3 Perfil espectral em UV

A Varredura realizada entre 190 e 330 nm apresentou máximos de absorção em 213,2; 242,6 e 282,8 nm com valores de absorvância 1,265; 0,903 e 0,080 respectivamente. Os resultados estão em acordo com aqueles relatados por Herath e colaboradores (1979) utilizando o mesmo solvente e por Wagner; Kreutzkamp e Jurcic (1985) utilizando metanol como solvente, FIGURA 18.

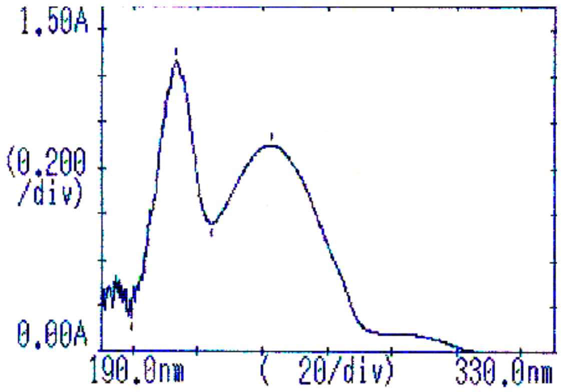

FIGURA 18 – ESPECTRO DE ABSORÇÃO DA MITRAFILINA NO ULTRAVIOLETA (190 – 330nm)

5.1.4 Análise da Pureza em CLAE-DAD

A análise da substância em CLAE-DAD apresentou o perfil da FIGURA 19. O pico (15,789 min) referente à substância em análise apresentou área que corresponde a 99,4% da área total do cromatograma e pureza de pico indicado pelo software de 99,25%. Além deste pico o cromatograma apresentou outro sinal em 23,838 min (0,6% da área total), identificado como pteropodina após injeção de padrão autêntico dessa molécula e comparação dos tempos de retenção.

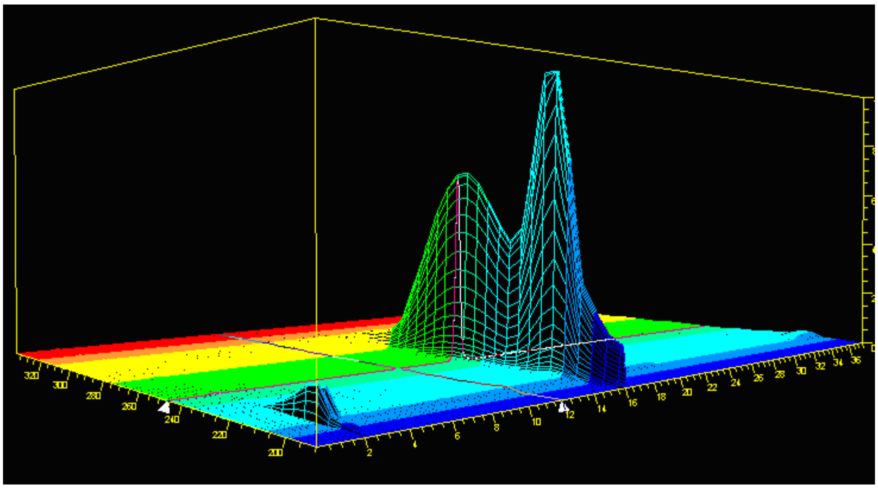

FIGURA 19 – ESPECTRO EM 3D DA MITRAFILINA ISOLADA OBTIDO EM CLAE-DAD

A presença do alcalóide pteropodina não foi possível de ser eliminada mesmo após as sucessivas etapas de cristalização. A similaridade da conformação dos anéis C,D e E da classe *allo* e normal associada à mesma conformação em C-7

(*R*) para mitrafilina e pteropodina, podem explicar o comportamento relativamente semelhante nos solventes utilizados para o isolamento.

É importante destacar que a pequena quantidade observada de pteropodina não inviabiliza o uso como padrão analítico da mitrafilina isolada, pois a pureza da última é bastante elevada e a possível interconversão entre esses alcalóides em solução foi acompanhada pela avaliação da estabilidade da solução analítica executada no item 5.2.4.

5.1.5 Confirmação da pureza por DSC

A DSC pode ser um método simples e rápido para estimar a pureza de materiais (FAROONGSARNG; KADEJINDA; SUNTHORNPIT, 2000). A técnica fornece informações sobre as propriedades físicas e energéticas da substância em análise como ponto de fusão, pureza e polimorfismo (CLAS; DALTON; HANCOCK, 1999).

O gráfico obtido da análise indica evento endotérmico, referente à fusão, ocorrido em 276,47 ^{o}C. O resultado é semelhante aos valores 275 e 272 ^{o}C publicados respectivamente por Herath *et al.* (1979) e Carbonezi *et al.* (2004), porém diverso de 265 ^{o}C publicado por Shellard, Tantivatana e Becket (1967) e 264 - 268 ^{o}C por Wagner; Kreutzkamp e Jurcic (1985). Os menores valores apresentados por último podem indicar que as amostras continham impurezas, pois a presença destas conduz o processo de fusão a menores temperaturas.

Além do citado, a alta pureza indicada pelo gráfico (98,25%) e o sinal bastante fino do evento de fusão, FIGURA 20, auxiliam na comprovação da pequena presença de impurezas na amostra, uma vez que conteúdos elevados destas causam alargamento do sinal do pico. A pureza apresentada é coerente com 99,4% obtido pela análise cromatográfica, uma vez que a DSC detectada a presença de interferentes como água, solventes residuais, metais e outras impurezas com pontos de fusão diferentes à substância em análise, enquanto por CLAE as mesmas não são consideradas.

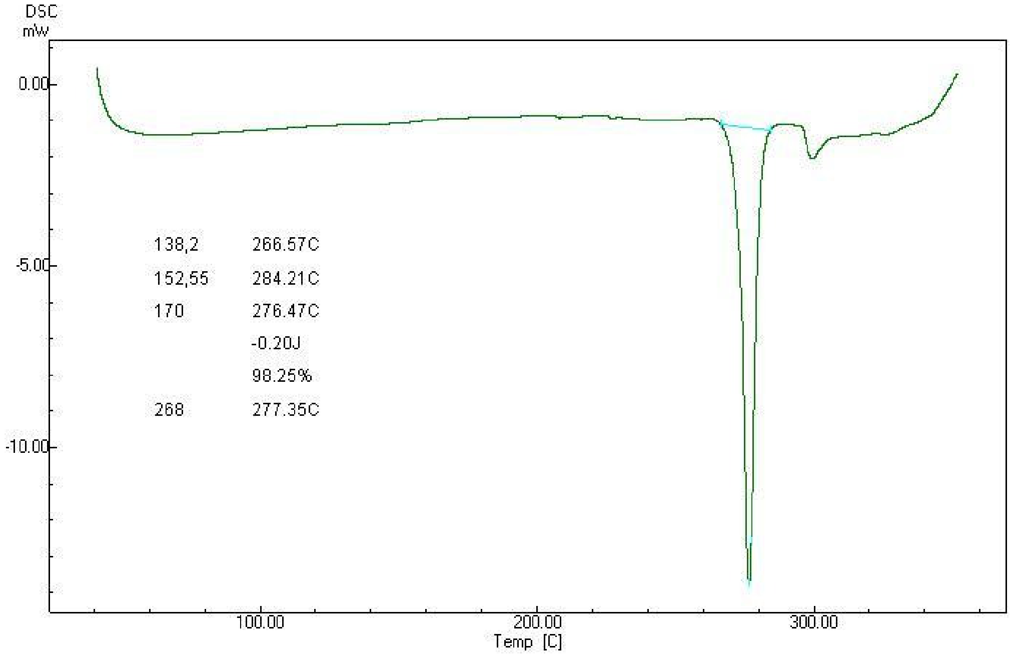

FIGURA 20 – ANÁLISE DE DSC DA MITRAFILINA ISOLADA

5.2 DESENVOLVIMENTO DO TAMPÃO ORGÂNICO E OTIMIZAÇÃO DOS MÉTODOS EM CLAE

A semelhança da matriz da tintura em relação à da casca após preparada e pronta para inserção no sistema cromatográfico, permitiu o desenvolvimento de um mesmo método CLAE para ambas. Isso foi possível, pois a tintura foi obtida após extração da planta com etanol 60% (v/v), mesmo solvente utilizado no método do preparo da amostra cascas (item 5.3.1).

A complexidade que caracteriza a matriz do gel impossibilitou a utilização do mesmo método cromatográfico empregado nas amostras já citadas e, assim, exigiu a elaboração de outro procedimento para CLAE (item 5.2.6).

5.2.1 Desenvolvimento do Tampão orgânico e Otimização do método em CLAE para pó e tintura

Inicialmente as condições de gradiente e fluxo propostas por Ganzera e colaboradores (2001), TABELA 7, foram otimizadas para as colunas disponíveis no laboratório, Luna C-18 (150 x 4,6 mm; 5 µm tamanho de partícula, 100 Å de poro e

17,5% de Carbono) Marca Phenomenex e Zorbax XDB C-18 (150 x 4,6mm; 3,5 µm tamanho de partícula, 80 Å de poro e 10% de Carbono) Marca Agilent.

TABELA 7 – CONDIÇÕES CROMATOGRÁFICAS PROPOSTAS POR GANZERA, (2004)

TEMPO (min)	TAMPÃO FOSFATO 10 mM pH 7,0 (%)	ACETONITRILA (%)	ÁCIDO ACÉTICO 1% EM METANOL (%)
0	65	35	0
17	65	35	0
25	50	50	0
30	50	50	0
31	0	0	100
36	0	0	100
37	65	35	0
47	65	35	0

NOTA: Coluna utilizada pelo autor: Luna C-18 (100 x 4,6 mm, 3 µm tamanho de partícula) Marca Phenomenex. Temperatura da coluna: ambiente. Fluxo: 0,75 mL/min. Injeção: 10 µL. Detecção: 245 nm.

As condições otimizadas (item 4.7.1) apresentaram melhor resolução entre os picos dos alcalóides com a coluna Zorbax fato explicado pelos menores diâmetros de partícula e poro desta em relação à Luna.

A necessidade de aumentar o tempo de vida da coluna e reduzir o tempo de análise conduziu ao desenvolvimento de tampão orgânico, acetato, em substituição ao tampão inorgânico, fosfato. Esse fator associado às condições consideradas críticas para a análise por Stuppner; Sturm e Konwalinka (1992) foram avaliadas em planejamento fatorial de experimentos, TABELA 8.

TABELA 8 – ETAPA DE TRIAGEM – PLANEJAMENTO FATORIAL PARA CLAE DAS CASCAS E TINTURA

FATOR	MÍNIMO	MÁXIMO
Natureza Tampão (A)	Acetato	Fosfato
pH do tampão (B)	6	7
Temperatura da coluna (C)	15 ^{o}C	35 ^{o}C

Os resultados foram avaliados em função da resolução entre os pares de picos críticos: uncarina F – mitrafilina e isomitrafilina – pteropodina.

O gráfico dos efeitos padronizados, FIGURA 21, demonstra a avaliação dos resultados para os picos uncarina F – mitrafilina e apresenta como fatores mais significativos o pH e temperatura da coluna. No gráfico os pontos afastados da reta descrita pelos valores pertencentes a uma mesma população, são considerados significativamente diferentes dos demais. Os resultados para o segundo par de picos críticos avaliados seguiu a mesma tendência do anterior.

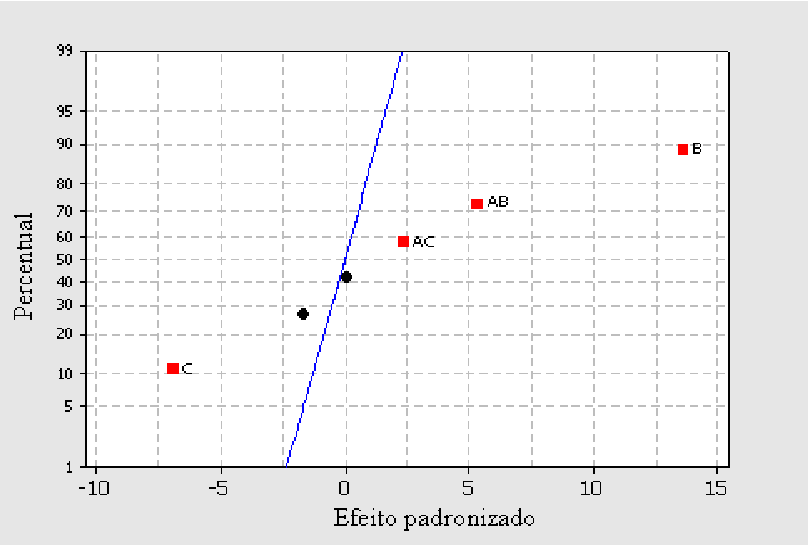

FIGURA 21 – EFEITOS PADRONIZADOS – PAR UNCARINA F - MITRAFILINA

LEGENDA: A: Natureza do tampão, B: pH, C: Temperatura da coluna. Vermelho – Significativo, Preto – Não significativo

Já o gráfico dos efeitos principais, FIGURA 22, caracteriza pela inclinação da linha a magnitude do efeito e pela direção da linha especifica se o efeito exerce influência positiva ou negativa na resolução dos pares de picos avaliados (GFRERER; LANKMAYR, 2005).

É possível evidenciar melhores valores de resolução em valores mais elevados de pH dos tampões e menores temperaturas da coluna para o par uncarina F - mitrafilina. Os resultados para o par isomitrafilina – pteropodina seguiram o mesmo padrão de resposta do par citado.

O comportamento frente ao pH do tampão pode ser explicado pelo pKa desses alcalóides. O valor de 5,7 a 6,7 citado por Laus (1996) para o pKa dos AO, indica que valores de pH do tampão acima de 6,7 mantêm o nitrogênio heterocíclico das moléculas em sua forma alcalina, diminuindo a afinidade pelos resíduos de silanóis da coluna. Isso conduz a um favorecimento nas interações do tipo hidrofóbicas e dipolo-dipolo entre os alcalóides e as cadeias C-18 da sílica, aumentando a eficiência da separação.

Já para o parâmetro temperatura da coluna, segundo Jardim, Collins e Guimarães (2006), não é possível estabelecer uma regra a respeito da influência da temperatura em separações por CLAE. O aumento da temperatura freqüentemente aumenta o desempenho da coluna por diminuir a viscosidade da fase móvel, o que melhora a transferência de massas entre essa fase e a fase estacionária, resultando em um melhor processo de separação. Entretanto, segundo Skoog e colaboradores

(2009), seu aumento também pode levar à redução dos estados de equilíbrio entre o analito e a fase estacionária, ocorrendo aumento do coeficiente de difusão das moléculas de interesse na fase móvel o que aumenta a altura equivalente dos pratos teóricos da coluna reduzindo assim a eficiência da separação, como no método avaliado.

O tampão acetato desenvolvido além de sua capacidade tamponante, realiza, pela presença da trietilamina, competição pelos resíduos de silanóis da coluna, evitando interações secundárias destes com porções mais polares dos alcalóides, aumentando assim a eficiência cromatográfica.

Como a natureza do tampão utilizado não resultou em melhoras para a resolução dos pares de picos estudados, o tampão acetato foi escolhido para a análise em substituição ao tampão inorgânico. A substituição por tampão acetato permitiu a eliminação da etapa final de limpeza da coluna com 1% de ácido acético em metanol, reduzindo o tempo total de análise.

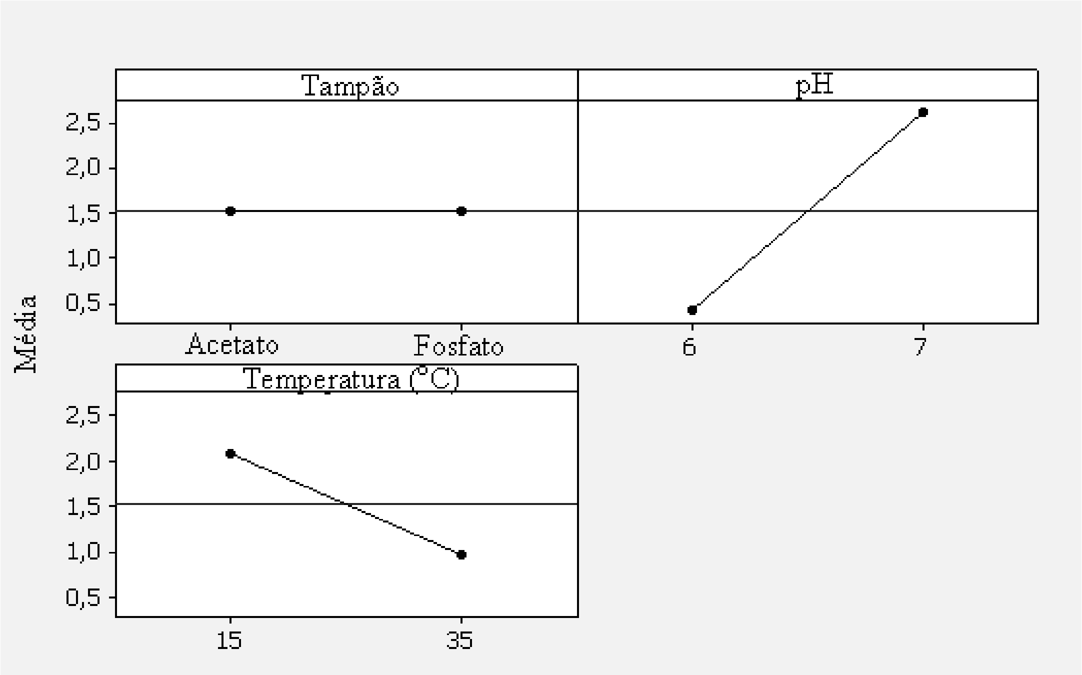

FIGURA 22 – EFEITOS PRINCIPAIS – PAR UNCARINA F – MITRAFILINA

Conforme discutido, os parâmetros pH do tampão e temperatura da coluna foram otimizados em design composto central através de design fatorial (2^2) com quatro pontos axiais e cinco replicatas no centro do delineamento, TABELA 9.

TABELA 9 – ETAPA DE OTIMIZAÇÃO – PLANEJAMENTO FATORIAL CLAE DAS CASCAS E TINTURA

FATOR	DOMÍNIO EXPERIMENTAL				
	$-\alpha^a$	-1	0	1	α^a
pH do Tampão	6.5	6.8	7.5	8.2	8.5
Temperatura da coluna (^{o}C)	10	12.2	17.5	22.8	25

Os gráficos de superfície de resposta obtidos permitem visualizar as melhores resoluções para os picos estudados nas zonas de tonalidades verdes mais escuras, FIGURA 23. Segundo Ciola (1998), para análises quantitativas é necessário um mínimo de resolução do pico de 1,5 e para a USP XXXI (2008) maior que 2,0, assim para se atender ao último critério, mais restritivo, foram selecionados valores de pH 6,9 e temperatura da coluna 15 ^{o}C com base no par de picos mais crítico isomitrafilina – pteropodina.

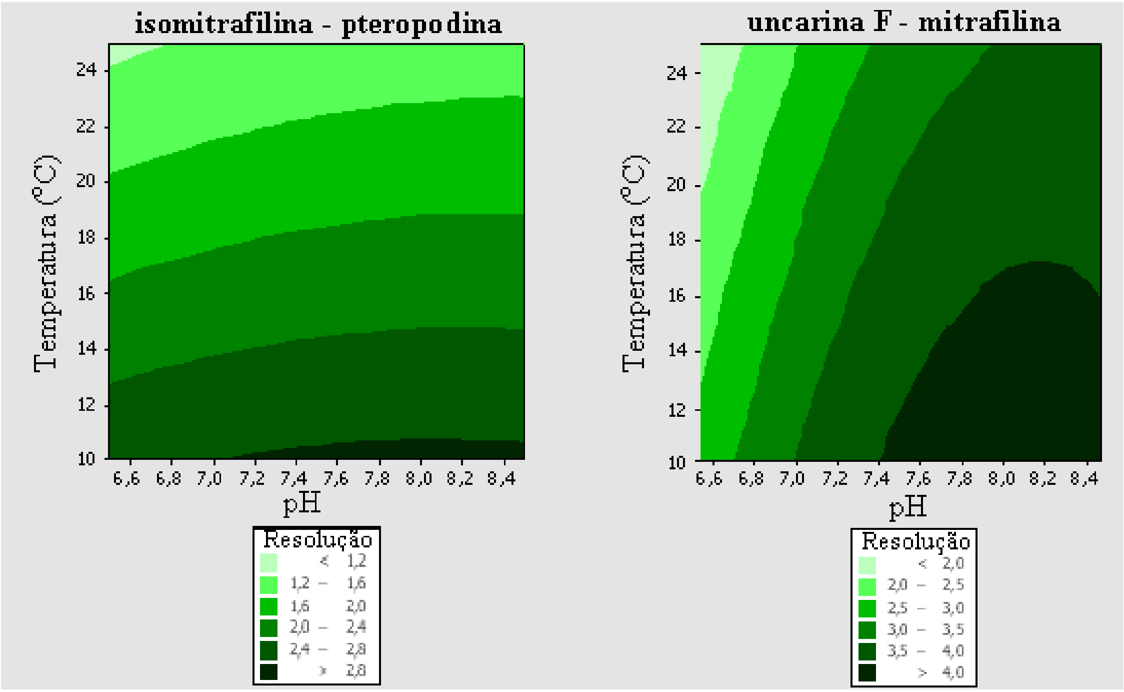

FIGURA 23 – SUPERFÍCIE DE RESPOSTA DAS CONDIÇÕES CLAE PARA CASCAS E TINTURA

Os resultados alcançados são semelhantes aos previamente apresentados por Stuppner e colaboradores (1992) obtidos em experimento univariado, no entanto a eficiência cromatográfica do trabalho citado está bem aquém daquelas apresentadas nas FIGURAS 24 e 25.

A escolha do pH 6,9 em detrimento dos demais valores é explicada pelo fato de em pH maior que 7,0 a rincofilina co-eluir com isomitraflina, já menores valores de pH não foram escolhidos para se garantir boa resolução entre os picos.

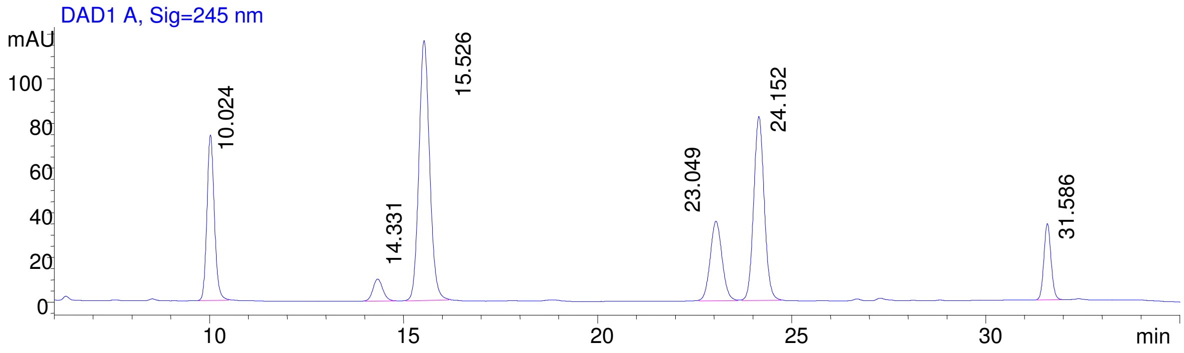

FIGURA 24 – PERFIL CROMATOGRÁFICO DAS CONDIÇÕES OTIMIZADAS PARA CASCAS E TINTURA – PLANTA BRASILEIRA

NOTA: especiofilina (10,02 min), uncarina F (14,33 min), mitrafilina (15,52 min), isomitrafilina (23,05 min), pteropodina (24,15 min) e isopteropodina (31,59 min).

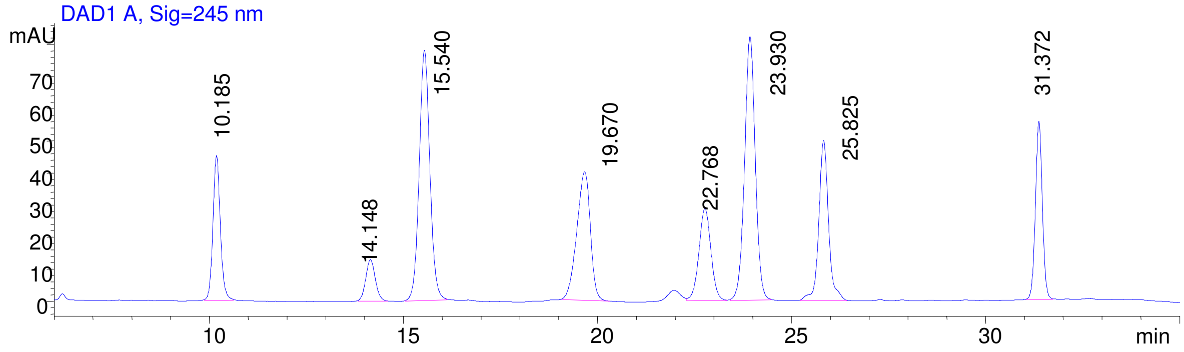

FIGURA 25 – PERFIL CROMATOGRÁFICO DAS CONDIÇÕES OTIMIZADAS PARA CASCAS E TINTURA – PLANTA PERUANA

NOTA: especiofilina (10,19 min), uncarina F (14,15 min), mitrafilina (15,54 min), rincofilina (19,67 min), isomitrafilina (22,77 min), pteropodina (23,93 min), isorincofilina (25, 82 min) e isopteropodina (31,37 min).

A identificação dos picos obtidos nos cromatogramas acima foi estabelecida por similaridade dos tempos de retenção e perfis espectrais em relação a padrões analíticos autênticos de mitrafilina, isomitrafilina, pteropodina e isopteropodina. Os demais alcalóides foram identificados por comparação com trabalhos previamente publicados por Ganzera e colaboradores (2001) e por Laus e Keplinger (1994) que utilizaram condições cromatográficas semelhantes às otimizadas no presente trabalho. Os testes de robustez indicaram não haver alteração na ordem de eluição

em função da fase estacionária utilizada. Além disso, a análise por espectrometria de massas, detalhada a seguir, auxiliou na identificação dos picos obtidos.

A planta peruana, FIGURA 25, apresentou teor de 0,88% para AOP e 0,31% para AOT. O resultado encontrado para AOT foi superior ao limite de 0,05% estabelecido pela USP XXXI. Dessa forma essa planta, como citado no item 4.5, foi utilizada apenas nos testes de verificação da capacidade do sistema CLAE em distinguir entre AOP e AOT, não sendo utilizada nas análises de validação.

5.2.2 Verificação da capacidade de distinção entre AOT e AOP

Vários estudos nas décadas de 1960, 1970 e mais recentemente 2004, relatam análise por EM para AOT e AOP, com utilização como fonte de íons impacto eletrônico (BECKETT; DWUMA-BADU; HADDOCK, 1969; PHILLIPSON; HEMINGWAY, 1975a; PHILLIPSON; HEMINGWAY, 1975b; HERATH *et al.*, 1979; CARBONEZI *et al.*, 2004). Embora o modo de ionização empregado pelos autores seja, provavelmente, o mais utilizado ainda hoje, frequentemente é desejável reduzir a fragmentação na fonte de íons de modo a simplificar a interpretação de moléculas de peso molecular elevado (MENDHAM *et al.*, 2002).

Ainda segundo Mendham e colaboradores (2002), ionizações mais brandas podem ser alcançadas com o emprego de outras fontes de íons entre elas a ionização por nebulização elétrica (electrospray), que operam à pressão atmosférica. Os processos de ionização à pressão atmosférica são mais eficientes por um fator de até 10^4 em relação aos processos que ocorrem sob vácuo, como o impacto eletrônico, e como conseqüência resultam em maior sensibilidade.

O trabalho publicado por Lopez-Avila e Bendicto (1997) fornece informações sobre o espectro de fragmentação dos AO em condições de ionização mais brandas (APCI). Informações sobre fragmentação com electrospray ainda não são disponíveis em literatura, embora esta seja hoje a fonte de ionização mais versátil existente para acoplamento com sistemas CLAE.

Conforme já discutido para a massa molecular da mitrafilina, 368,4263 Da, a relação *m/z* esperada para o seu íon molecular em modo positivo de fragmentação seria 361,4 Da. Essa relação esperada pode ser estendida aos demais AOP tendo em vista serem todos isômeros conformacionais. Já para os AOT como a massa

molecular é de 384,4687 Da, a relação *m/z* esperada para o íon molecular em modo positivo de fragmentação é de 385,4 Da.

O monitoramento em MS1 das razões *m/z* efetuado entre 150 e 400 Da, FIGURAS 26 e 27, para as injeções em CLAE-EM das amostras de cascas da planta brasileira e peruana, exigiu a substituição da trietilamina por hidróxido de amônio para o ajuste do pH do tampão.

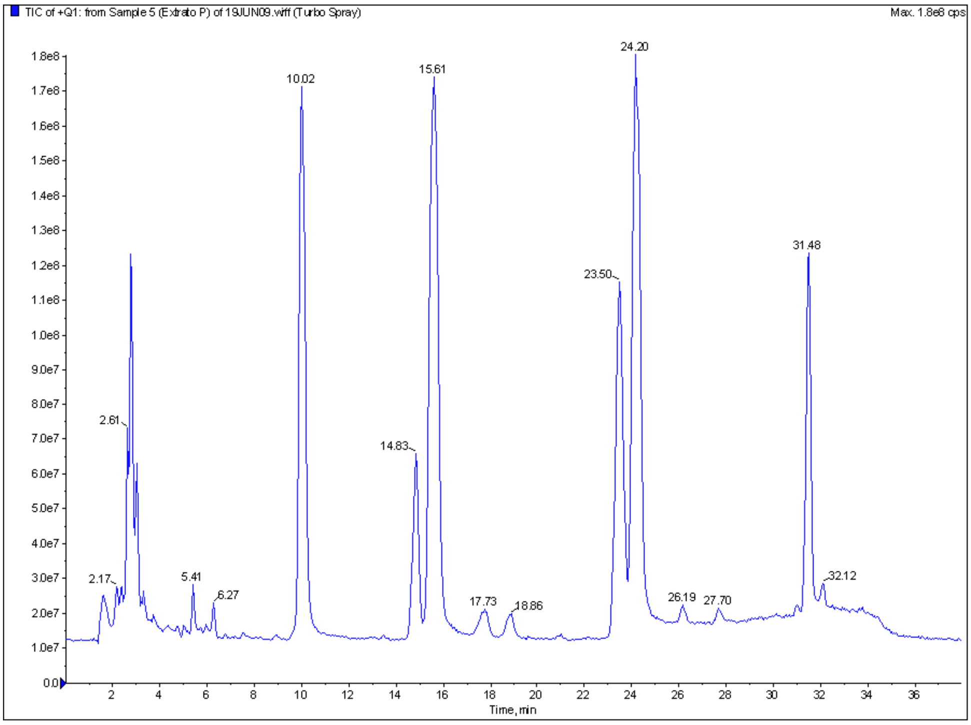

FIGURA 26 – EM – MS1 DA PLANTA BRASILEIRA

NOTA: especiofilina (10,02 min), uncarina F (14,83 min), mitrafilina (15,61 min), rincofilina (17,73 min), pico desconhecido (18,86), isomitrafilina (23,50 min), pteropodina (24,20 min), isorincofilina (26,19 min), pico desconhecido (27,70 min) e isopteropodina (31,48 min).

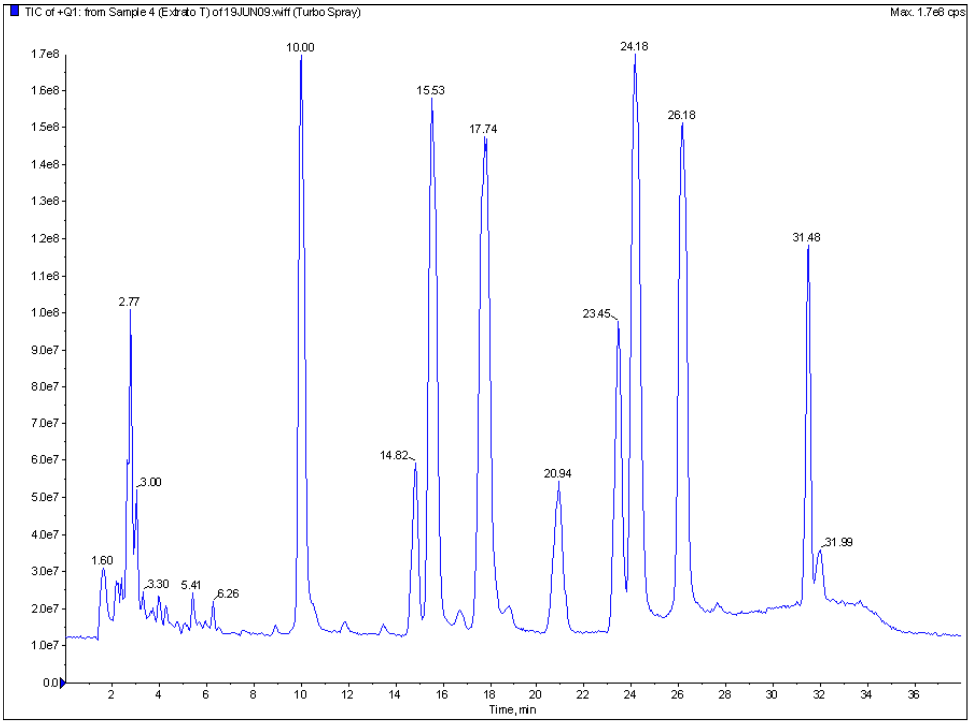

FIGURA 27 – EM – MS1 DA PLANTA PERUANA

NOTA: especiofilina (10,00 min), uncarina F (14,82 min), mitrafilina (15,53 min), rincofilina (17,74 min), isomitrafilina (23,45 min), pteropodina (24,18 min), isorincofilina (26,18 min), isopteropodina (31,48 min) e pico desconhecido (31,99 min).

As figuras acima apresentam os mesmos perfis e ordem de eluição dos cromatogramas obtidos com detecção UV, FIGURAS 24 e 25 (item 5.2.1).

Na TABELA 10 são apresentadas apenas as relações *m/z* observadas para sinais de AO, $[M+H]^+$ 369 e 385 Da. Pelos dados apresentados evidencia-se que a planta brasileira, embora não tenha apresentado AOT em análise CLAE-UV, não é destituída desses alcalóides. A análise confirma os sinais em tempos de retenção 17,7 e 26,3 como AOT, bem como demonstra boa separação e confiável distinção entre AOT e AOP pelo método cromatográfico desenvolvido.

TABELA 10 – ÍONS MOLECULARES OBTIDOS EM CLAE-MS PARA A PLANTA BRASILEIRA E PERUANA

Uncaria tomentosa – PLANTA BRASILEIRA			*Uncaria tomentosa* – PLANTA PERUANA		
TR (min)	RELAÇÃO *m/z*	% ÁREA	TR (min)	RELAÇÃO *m/z*	% ÁREA
10,02	369,4	16,68	10,00	369,4	11,00
14,83	369,0	5,40	14,82	369,1	3,44
15,61	369,4	23,98	15,53	368,8	13,80
17,73*	***385,2***	***1,08***	***17,74***	***385,5***	***15,69***
18,86*	368,9	0,82	23,45	369,0	6,84
23,50	369,1	13,71	24,18	369,1	15,89
24,20	368,7	23,81	***26,18***	***385,5***	***12,68***
26,19*	***385,2***	***0,41***	31,48	368,9	5,52
27,70*	368,9	0,33	31,99*	369,0	1,01
31,48	369,0	9,03			

NOTA: *Sinais não observados por CLAE-UV; ***Destacado os dados relativos aos AOT***

Não foi encontrada na literatura descrição da possível identidade dos picos observados em 18,86; 27,70 e 31,99 min, mas fica evidente pelos dados obtidos se tratarem de AOP.

Embora na planta brasileira, FIGURA 26, tenha sido evidenciado pico em 32,12 min, este não apresentou *m/z* característico de AOP ou AOT, não podendo ser o AOP com tempo de retenção 31,99 min observado na planta peruana, FIGURA 27. Além disso, o pico de grande intensidade em 20,94 min presente na planta peruana não apresentou relação *m/z* característica de AOP ou AOT.

O monitoramento em MS2 dos íons produto de 369 e 385 Da forneceu os perfis de fragmentação para todos AOP e AOT presentes nos cromatogramas, conforme modelos da FIGURA 28.

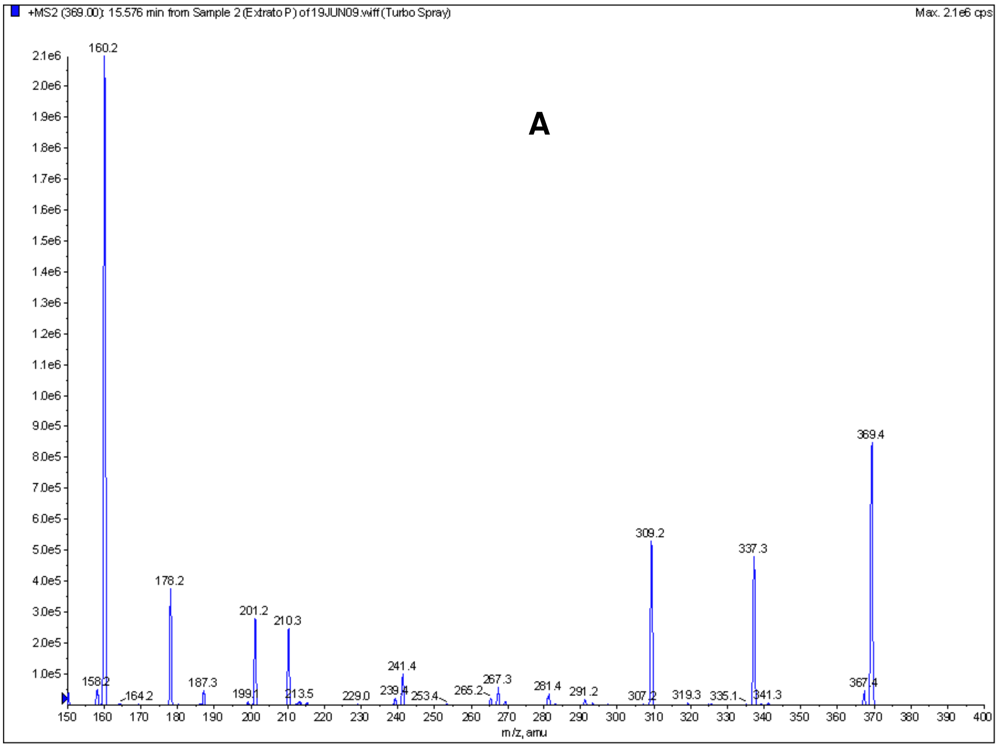

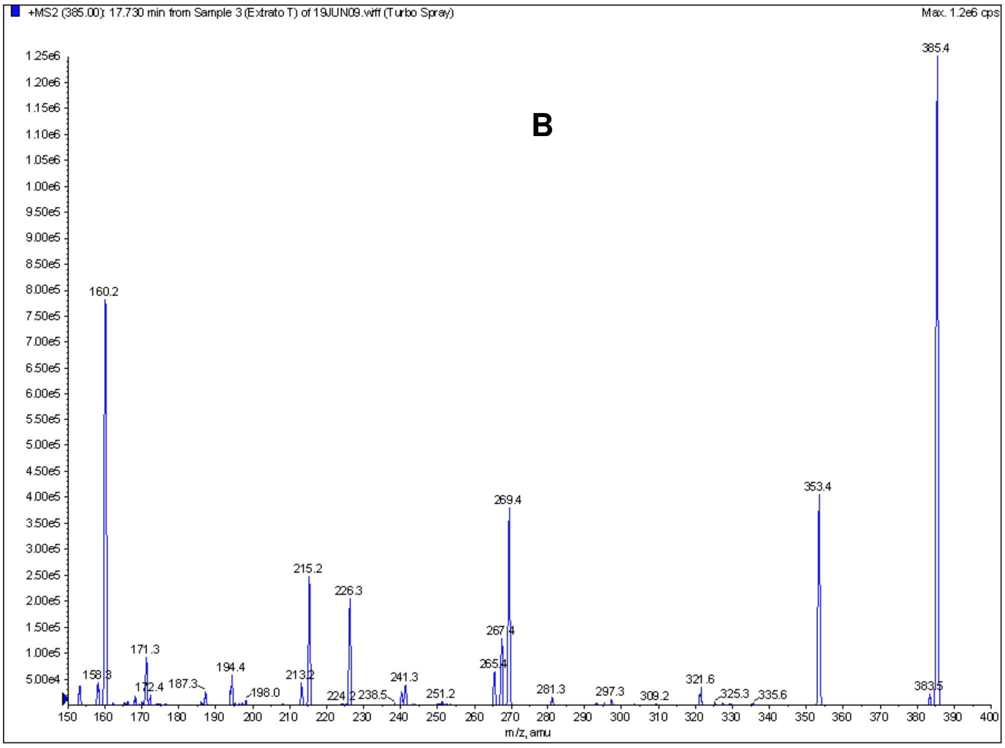

FIGURA 28 – MS2 DOS ALCALÓIDES OXINDÓLICOS

LEGENDA: A - MS2 mitrafilina; B – MS2 rincofilina

Os demais perfis de fragmentação para cada alcalóide seguiram o mesmo padrão apresentado em A e B, respeitando-se a classe AOT ou AOP. Os seis principais íons obtidos em cada caso encontram-se na TABELA 11.

TABELA 11 – IONS PRODUTO DOS PRINCIPAIS ALCALÓIDES DE *U. tomentosa*

AOP									AOT	
especiofilina (10,02 min)	uncarina D (14,83 min)	mitrafilina (15,61 min)	18,86 min	isomitrafilina (23,50 min)	pteropodina (24,20 min)	27,70 min	isopteropodina (31,48 min)	31,99 min	rincofilina (17,74 min)	isorincofilina (26,18 min)
160,2[1]	160,3	160,2	160,3	160,2	160,3	160,1	160,2	160,2	160,2	160,2
(65,25)[2]	(69,83)	(100,00)	(100,00)	(100,00)	(80,27)	(100,00)	(81,59)	(87,96)	(54,22)	(35,28)
201,3	178,2	178,2	178,1	178,2	178,1	178,0	178,2	201,2	215,2	215,3
(14,85)	(7,88)	(16,65)	(16,05)	(17,82)	(9,08)	(15,29)	(8,10)	(24,07)	(16,22)	(9,61)
213,1	201,3	201,1	201,3	201,2	201,2	201,3	201,3	226,3	226,3	241,3
(8,38)	(12,31)	(11,63)	(18,72)	(10,90)	(16,75)	(18,24)	(20,54)	(30,56)	(13,75)	(18,03)
239,3	213,1	309,4	309,3	309,3	213,3	309,5	213,3	238,5	269,4	269,4
(7,54)	(7,52)	(26,99)	(21,40)	(28,31)	(9,73)	(20,00)	(8,49)	(26,85)	(24,08)	(17,68)
337,4	337,2	337,4	337,1	337,4	337,4	337,3	337,3	337,2	353,4	353,4
(100,00)	(79,11)	(22,89)	(36,83)	(24,54)	(100,00)	(53,53)	(100,00)	(100,00)	(29,31)	(47,48)
369,1	369,3	369,1	369,2	369,1	369,2	369,3	369,3	369,4	385,4	385,4
(46,76)	(100,00)	(42,72)	(43,21)	(67,08)	(56,19)	(65,59)	(54,18)	(81,48)	(100,00)	(100,00)

LEGENDA: [1]razão *m/z*; [2](intensidade %)

Pela análise dos dados fica claro que os alcalóides seguem um perfil de fragmentação conforme a classe representada (AOP ou AOT) e o tipo (normal*, allo* ou *epiallo*) em que são classificados (item 3.2). Pelo padrão de fragmentação apresentado os alcalóides em 18,86 e 27,70 min são provavelmente do tipo normal, pois apresentam os mesmos fragmentos, em intensidades semelhantes, aos alcalóides do mesmo tipo mitrafilina e isomitrafilina. Esses alcalóides podem ser as uncarinas A ou B descritas no gênero *Uncaria*, contudo não descritas na espécie em estudo (HEITZMAN *et al.*, 2005).

Já o alcalóide em 31,99 min, presente na planta peruana, apresentou perfil de fragmentação muito semelhante aos alcalóides do tipo *allo* (pteropodina e isopteropodina), com pico base de *m/z* 337,2. Esse alcalóide pode ser tanto o oxindólico rauniticina-*allo* A quanto o B, já descritos no gênero (*U. elliptica*), mas ainda não na unha-de-gato (HEITZMAN *et al.*, 2005).

A não descrição desses alcalóides por outros autores pode ser resultado do uso do modo de ionização impacto eletrônico na análise das amostras estudadas de *U. tomentosa*, modo esse que como já citado é menos sensível que electrospray.

Os novos alcalóides encontrados na espécie *U. tomentosa* foram detectados com pequena intensidade de sinal, o que pode explicar a diferença de fragmentação do alcalóide em 31,99 min em relação aos estruturalmente relacionados do tipo *allo*.

A pequena intensidade dos sinais obtidos para esses novos alcalóides não altera de maneira significativa o teor calculado para AO por CLAE – UV na espécie *U. tomentosa*.

Na FIGURA 29 segue proposta dos fragmentos formulada com base na abundância obtida para os íons produto de *m/z* 369.

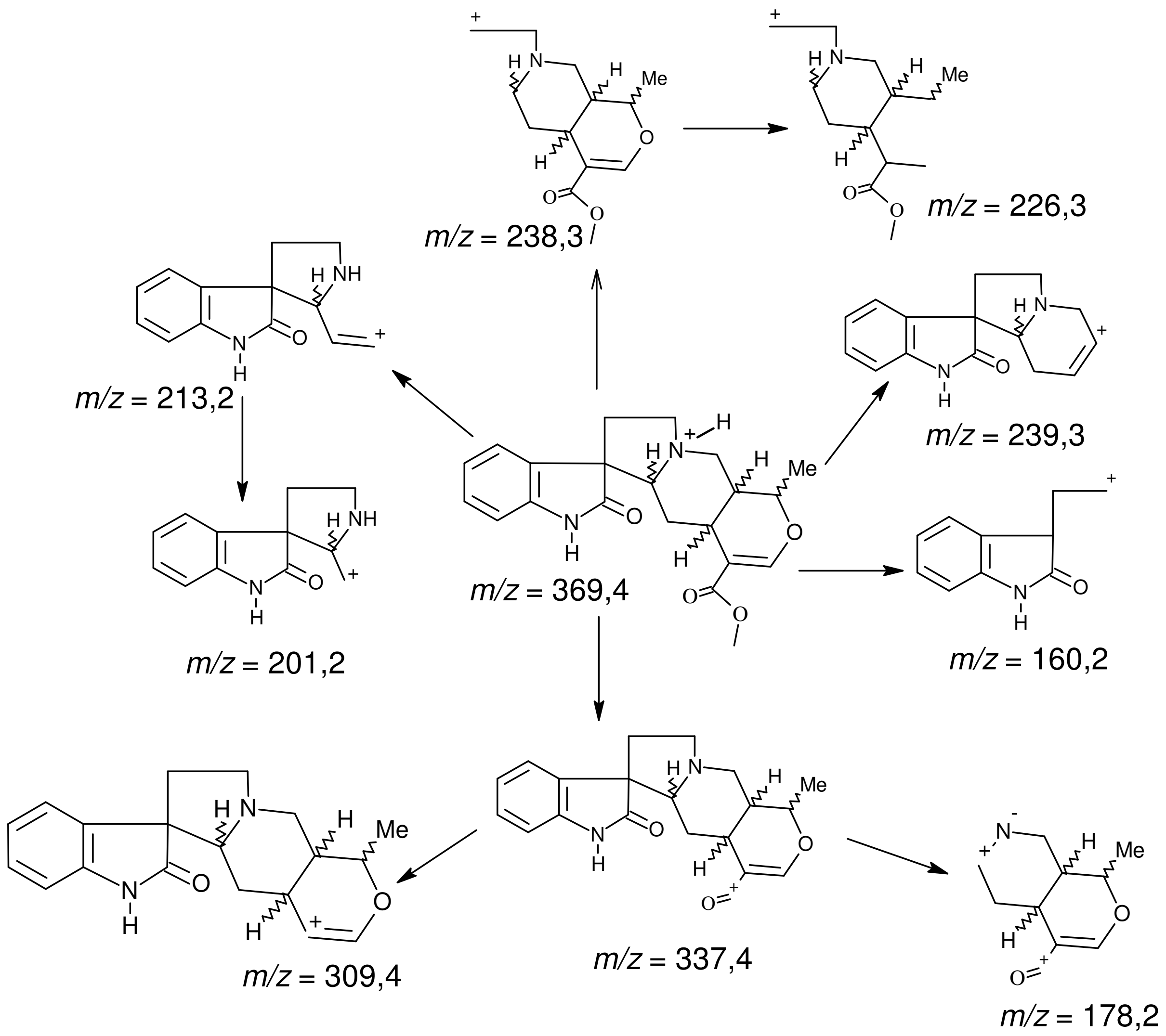

FIGURA 29 – PROPOSTA DE FRAGMENTAÇÃO PARA AOP

Em todos os tipos de AOP foram evidenciados fragmentos de *m/z* 160,2 Da correspondente à porção indólica do alcalóide, decorrente da ruptura no anel de cinco membros mais tencionado da porção terpênica. Os sinais de *m/z* 178,2 e 238,3 Da decorrem da fragmentação em porção distinta do mesmo anel de cinco membros. Todos esses fragmentos além do *m/z* 201,2 e 213,2 Da são quebras adjacentes ao nitrogênio básico da molécula. Os demais sinais de *m/z* 309,4 e 337,4 Da são perdas da porção éster e metila respectivamente, próximas à região bastante eletrofílica das moléculas.

Os fragmentos de *m/z* 226,3 e 239,3 Da são resultantes da fragmentação da porção do éter cíclico derivado da secologanina.

Para AOT foi observado o mesmo fragmento de *m/z* 160,2 Da derivado da porção indólica. Os demais fragmentos estão relacionados à fragmentação dos

grupamentos éster e éter da porção terpênica da molécula (*m/z* = 215,3; 241,3; 269,4 e 353,4 Da).

Já o fragmento de *m/z* 226,3 Da é originado pela fragmentação das porções indólica e terpênica (éter) da molécula, FIGURA 30.

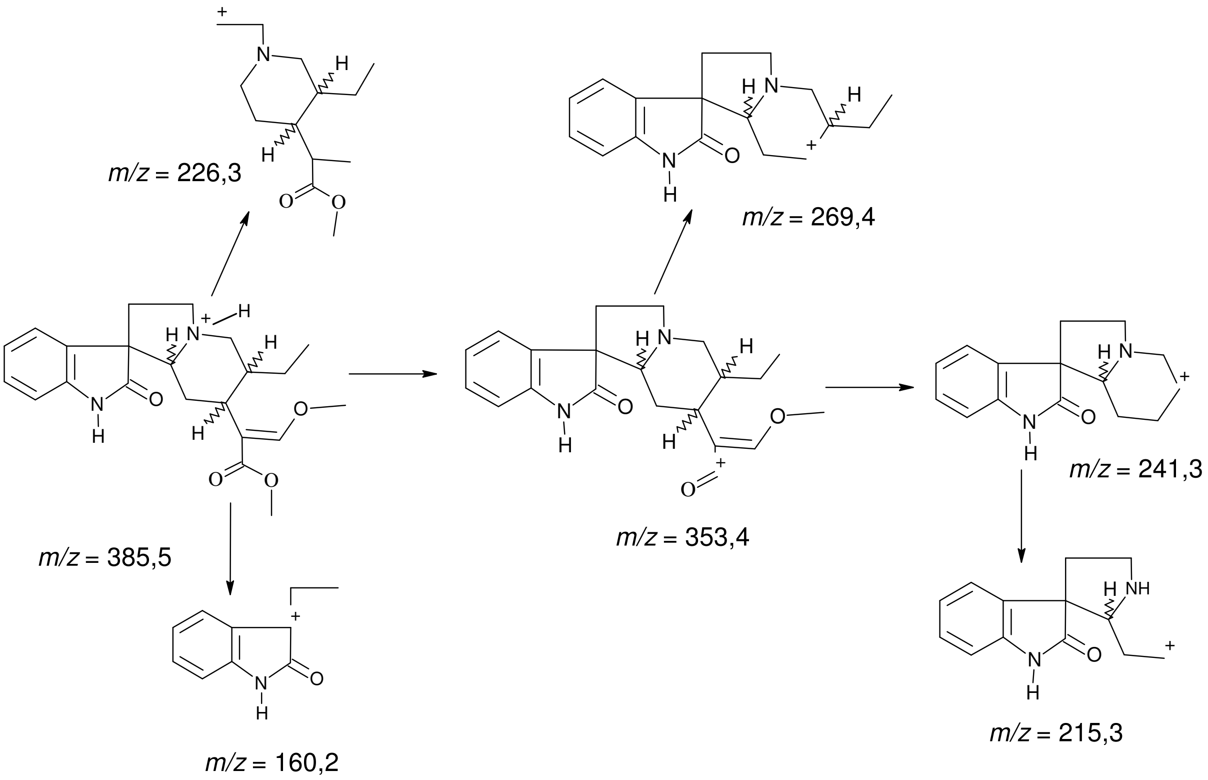

FIGURA 30 – PROPOSTA DE FRAGMENTAÇÃO PARA AOT

5.2.3 Escolha do padrão externo

A maior parte dos trabalhos que descrevem métodos de análise para *U. tomentosa* utilizam como padronização externa o alcalóide pteropodina, culminando com a recente monografia da USP XXXI estabelecendo esse mesmo alcalóide para padronização da planta (STUPPNER; STURM, 1992; LAUS; KEPLINGER, 1994; GANZERA *et al.*, 2001; VALENTE *et al.*, 2006; USP, 2008).

Tendo em vista a complexidade da composição alcalóidica da espécie, seis AOP e dois AOT, foi analisada qual a melhor alternativa para padronização das cascas, ou seja, qual dos alcalóides isolados disponíveis forneceria resultados mais confiáveis para expressar o teor alcaloídico.

Os dados obtidos para cada curva analítica preparada, conforme item 4.7.3, estão compilados na TABELA 12. Pelos resultados pode ser inferido absortividades semelhantes para alcalóides com mesma conformação em C-7. Mitrafilina e pteropodina, conformação *R* em C-7, apresentam valores menores para a razão concentração/área em relação aos alcalóides de conformação *S* em C-7, isomitrafilina e isopteropodina. Esta evidência suporta a afirmação de que a porção indólica influi de maneira significativa no comportamento da absorção da molécula no ultra-violeta.

TABELA 12 – COMPARAÇÃO DAS CURVAS ANALÍTICAS AVALIADAS

ALCALÓIDE	CONCENTRAÇÃO ($\mu g.mL^{-1}$)	ÁREA (mAU.s)	CONCENTRAÇÃO / ÁREA
Mitrafilina (3)	8,10	240,15	$3,37 \times 10^{-2}$
	20,30	604,56	$3,36 \times 10^{-2}$
	40,60	1.221,78	$3,32 \times 10^{-2}$
	81,20	2.447,29	$3,32 \times 10^{-2}$
	203,00	6.089,92	$3,33 \times 10^{-2}$
Isomitrafilina (4)	6,50	178,66	$3,64 \times 10^{-2}$
	16,30	452,16	$3,60 \times 10^{-2}$
	32,60	908,34	$3,59 \times 10^{-2}$
	65,20	1.830,99	$3,56 \times 10^{-2}$
	163,0	4.565,42	$3,57 \times 10^{-2}$
Pteropodina (5)	8,60	264,26	$3,25 \times 10^{-2}$
	21,40	655,95	$3,26 \times 10^{-2}$
	42,80	1.323,82	$3,23 \times 10^{-2}$
	85,60	2.651,68	$3,23 \times 10^{-2}$
	214,00	6.787,08	$3,15 \times 10^{-2}$
Isopteropodina (6)	8,10	221,82	$3,65 \times 10^{-2}$
	20,30	564,16	$3,60 \times 10^{-2}$
	40,60	1.138,01	$3,57 \times 10^{-2}$
	81,20	2.267,92	$3,58 \times 10^{-2}$
	203,00	5.716,02	$3,55 \times 10^{-2}$

Os valores da TABELA 13 apresentam os resultados obtidos de amostras calculadas frente a cada uma das curvas.

TABELA 13 – COMPARAÇÃO DOS DIFERENTES TEORES ALCALÓIDICOS OBTIDOS FRENTE ÀS DIFERENTES CURVAS ANALITICAS TESTADAS

	TEOR DE ALCALÓIDES (%)				
AMOSTRA	CURVA 3	CURVA 4	CURVA 5	CURVA 6	VALOR VERDADEIRO*
1	1,09	1,17	1,05	1,17	1,11
2	1,06	1,14	1,03	1,14	1,08
3	1,07	1,15	1,04	1,15	1,09
Média	1,07	1,15	1,04	1,15	1,09

NOTA: *Cada alcalóide calculado em função da sua respectiva curva analítica, especiofilina calculada com curva da isopteropodina e uncarina F com curva da mitrafilina.

Os resultados da última coluna são considerados como os valores verdadeiros, pois cada alcalóide da amostra foi calculado frente ao seu padrão analítico, ou seja, o pico da mitrafilina com a curva analítica da mitrafilina, o pico da isomitrafilina com a curva da isomitrafilina e assim sucessivamente. Especiofilina e uncarina F, alcalóides sem padrão analítico disponível, foram calculadas frente a padrão de alcalóide com mesma conformação em C-7, nesse caso isopteropodina e mitrafilina respectivamente.

A análise de variância, execução do teste de Tukey com 95% de confiança apresenta valores semelhantes para resultados obtidos entre curvas analíticas de alcalóides com mesma conformação em C-7, mitrafilina e pteropodina (conformação *R*) e isomitrafilina e isopteropodina (conformação *S*).

Além disso, o teste acusa diferença significativa quando comparados entre si resultados obtidos com curvas analíticas provenientes de alcalóides com diferentes conformações em C-7.

Finalmente a análise estatística aponta como semelhante aos valores considerados verdadeiros, apenas os valores obtidos com a curva da mitrafilina, assim para a amostra utilizada foi estabelecida padronização externa em mitrafilina como a mais adequada.

5.2.4 Estabilidade das soluções padrão

A mitrafilina isolada e selecionada como padrão externo ideal para quantificação dos AOP, foi preparada, estocada e analisada conforme item 4.7.4.

Para os tempos analisados, 15, 30, 60, 90 e 120 dias, os valores de t calculados foram inferiores ao t tabelado (n=4), confirmando a estabilidade da curva analítica por até 120 dias quando armazenada nas condições descritas. Além disso, os coeficientes de correlação superiores a 0,9999 obtidos para todas as curvas confirmam a estabilidade da solução analítica de mitrafilina por esse período, FIGURA 31.

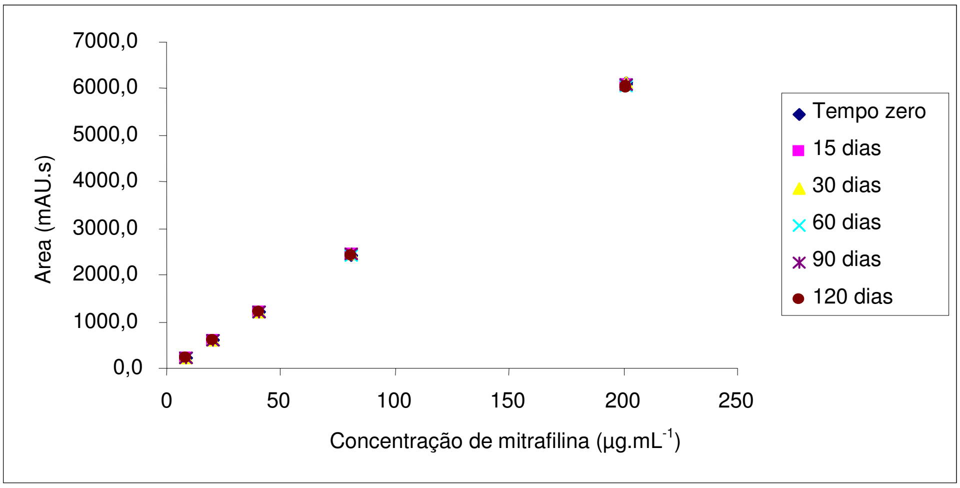

FIGURA 31 – ESTABILIDADE DAS SOLUÇÕES PADRÃO

Esses resultados permitem afirmar que não existe interconversão entre mitrafilina e a pteropodina residual presente no padrão isolado dentro do intervalo de tempo avaliado.

5.2.5 Conformidade do sistema método CLAE para cascas e tintura

Segundo Ermer (2001), durante o desenvolvimento e validação do método analítico, parâmetros de performance devem ser identificados para orientar limites de conformidade do sistema.

A USP (2008) recomenda que os parâmetros acompanhados sejam resolução entre picos, precisão de pelo menos 5 replicatas e o fator cauda ou simetria do pico. Dos três parâmetros citados a farmacopéia afirma que a resolução é uma função da eficiência da coluna e assim a forma mais confiável de se realizar a avaliação da conformidade do sistema. Estabelece ainda que os testes devam ser executados com injeções do padrão analítico.

A realização deste procedimento com a amostra tentou garantir a adequabilidade do sistema mesmo em presença da matriz responsável pela dificuldade nos procedimentos cromatográficos associados a drogas vegetais. Conforme orientado pelo ICH (2005), devido à sua natureza complexa, procedimentos analíticos para produtos biológicos e biotecnológicos podem, em

alguns casos, sofrer abordagens diferentes do descrito pelo documento para comprovação da confiabilidade do método.

As injeções realizadas apresentaram simetrias entre 0,91 e 0,98 para todos os picos relativos aos AO, resolução mínima de 2,25 entre os picos pteropodina – isomitrafilina e DPR inferior a 0,96% entre as áreas dos picos.

As simetrias de pico encontradas permitem correta integração dos picos analisados e contribuem para a eficiência cromatográfica. Como o resultado encontrado para resolução entre os pares de picos críticos foi superior ao limite estabelecido pela USP XXXI, mínimo 2,0, pode ser atestada a conformidade do sistema cromatográfico.

A avaliação não foi realizada com a amostra tintura por se tratar de matriz idêntica à obtida com o preparo de amostra das cascas.

5.2.6 Otimização do método CLAE análise do gel

O método CLAE desenvolvido para pó e tintura não foi possível de ser utilizado no gel. O parahidroxibenzoato de metila (metil parabeno) e parahidroxibenzoato de propila (propil parabeno) utilizados como conservantes na formulação, apresentaram característica de absorção no ultravioleta muito próxima, $\lambda_{máx}$ = 260 nm em etanol, em relação aos AO. Além disso, o comportamento do propil parabeno no método CLAE desenvolvido foi muito semelhante à mitrafilina, comprovado pela contaminação de 0,5 mL de tintura com 0,5 mL de propil parabeno em etanol (5 mg/mL), FIGURA 32. O incremento da área da mitrafilina comprova a co-eluição.

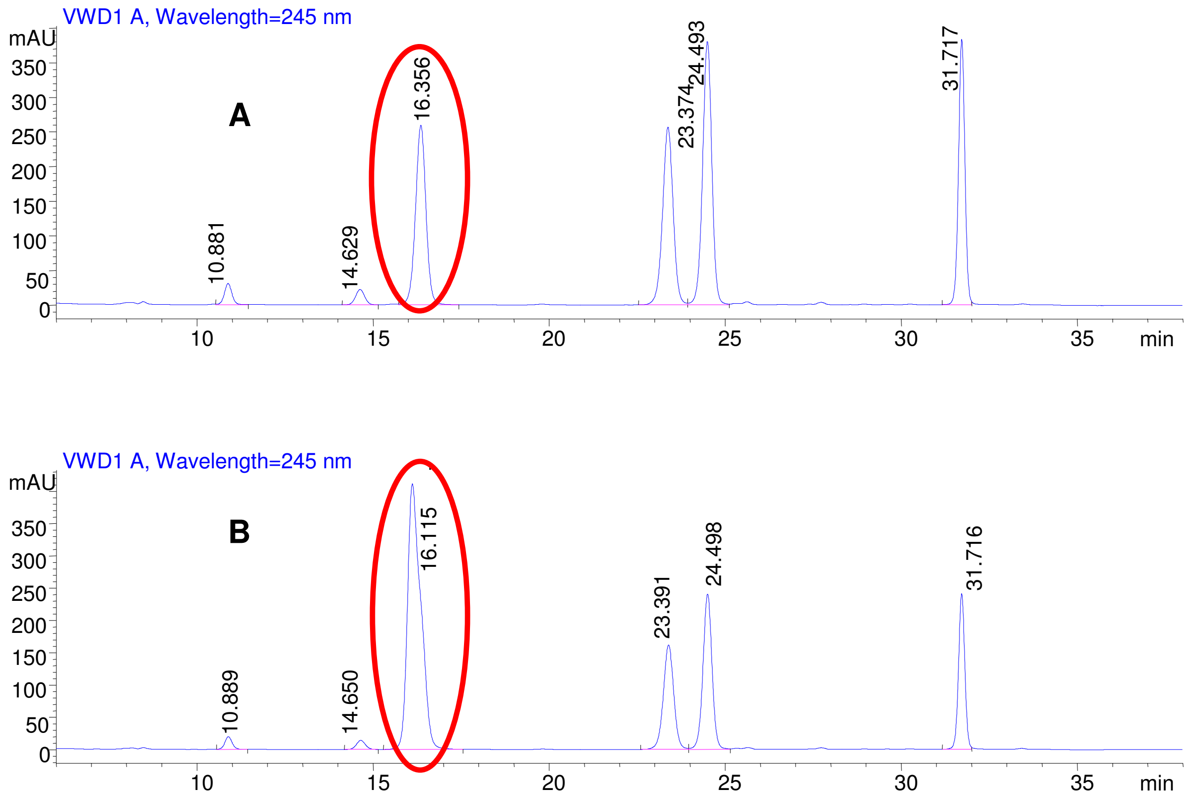

FIGURA 32 – PERFIL DE ELUIÇÃO DO PROPIL PARABENO NO METODO CLAE PARA CASCAS E TINTURA

LEGENDA: A – Perfil da tintura não contaminada; B – Perfil da tintura contaminada
NOTA: Destacada co-eluição do propil parabeno sobre a mitrafilina

As tentativas de otimização foram realizadas no sentido de manter o tampão orgânico e alterar apenas o gradiente, de maneira a se obter separação do conservante. Os testes iniciais primaram pela redução da força da fase móvel reduzindo a quantidade inicial de MeCN no gradiente. Estes testes conduziram a tempos de análise de até 53 min sem, contudo, separar o conservante dos alcalóides, ora parabeno co-eluia com mitrafilina ora com uncarina F.

A alternativa encontrada foi, além de reduzir a força da fase móvel, alterar a sua seletividade pela utilização de outro solvente. Segundo Jardim, Colins e Guimarães (2006), quando em uma separação cromatográfica, após o ajuste da força cromatográfica, existirem picos sobrepostos no cromatograma, a solução é alterar a seletividade da fase móvel até se obter fator de separação suficiente. Ciola (1998) cita resultados muito interessantes com misturas de metanol em MeCN para a melhora da seletividade da fase móvel na separação de multicomponentes.

Desta maneira, a MeCN utilizada no gradiente foi substituída primeiramente por mistura 1:1 desse solvente em metanol e posteriormente, após sucessivas

otimizações, relação 2:1 da mistura apresentada, conforme item 4.7.5, sendo obtido o perfil cromatográfico apresentado na FIGURA 33.

Separação cromatográfica completa foi obtida em 47 min de análise. O metil e propil parabenos eluiram em 12,6 e 25,3 min respectivamente.

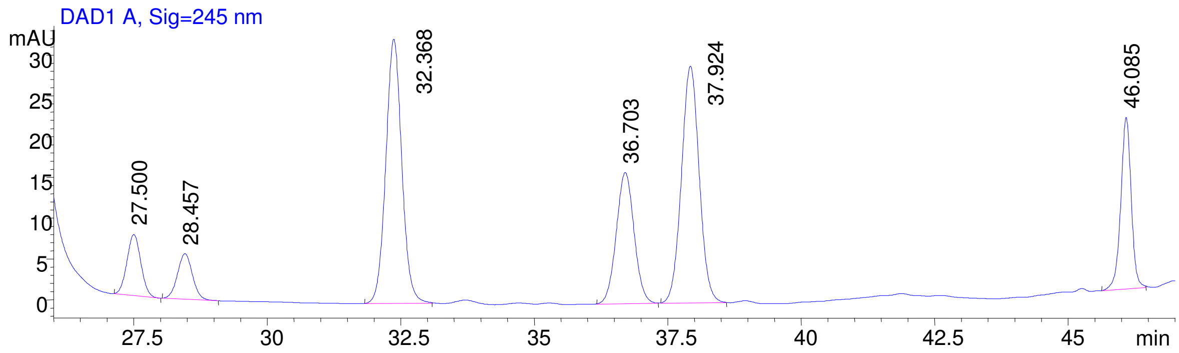

FIGURA 33 – PERFIL CROMATOGRÁFICO DOS AO DO GEL APÓS OTIMIZAÇÕES

NOTA: especiofilina (27,50 min), uncarina F (28,46 min), mitrafilina (32,37 min), isomitrafilina (36,70 min), pteropodina (37,92 min) e isopteropodina (46,08 min).

5.2.7 Conformidade do sistema método CLAE para gel

A resolução encontrada para o par pteropodina – isomitrafilina foi superior a 2,34 e os valores de simetrias de todos os picos entre 0,92 e 1,00. Como os valores de simetrias e resolução dos picos foram satisfatórios, o método pode ser dito eficiente e seletivo na separação dos AOP (JARDIM; COLLINS; GUIMARÃES, 2006).

O DPR entre as áreas dos picos referentes à especiofilina, uncarina F, mitrafilina, isomitrafilina e pteropodina foi inferior a 0,97%, apenas para o alcalóide isopteropodina as áreas variaram com DPR de 1,05%. No entanto, esse valor é muito próximo ao limite de 1,0% estabelecido pela USP XXXI e face à complexidade da matriz analisada pode ser aceito como comprovação da conformidade do sistema cromatográfico.

5.3 MÉTODOS DE PREPARO DAS AMOSTRAS

5.3.1 Preparo da amostra cascas

Para a extração dos AO das cascas foi selecionado o método de ultrassom por ser mais prático e rápido em relação ao tradicionalmente usado extração por refluxo. A extração por ultrassom é atribuída ao fenômeno de cavitação do solvente, causado pela passagem de uma onda ultrassonica.

Bolhas de cavitação são produzidas e comprimidas no processo e com o aumento da pressão e temperatura ocorre rompimento das mesmas, uma onda de choque passa então pelo solvente aumentando a mistura e penetração do solvente na matriz. Este efeito aumenta a área superficial de contato do líquido com a amostra, aumentando a transferência de massas pela desruptura das células. Nesse processo, maiores temperaturas aumentam o número de bolhas de cavitação formadas (LIAZID *et al.*, 2007).

Diferentemente dos métodos descritos em literatura que aplicam múltiplas etapas no processo de extração da planta, com o ultrassom foi testado o esgotamento dos AO presentes na amostra em uma única etapa extrativa (STUPPNER; STURM; KONWALINKA, 1992; LAUS; KEPLINGER, 1994; GANZERA *et al.*, 2001).

Devido a todo preparo de amostra ser afetado pelo tempo, tamanho da partícula, massa de amostra submetida à extração e ao solvente extrator utilizado, esses fatores foram testados na etapa de triagem, TABELA 14. Além disso, como o método de ultrassom é afetado pela temperatura, esse fator também foi incluído para teste na mesma etapa. Os resultados foram avaliados em termos do teor obtido de AO (%).

TABELA 14 – ETAPA DE TRIAGEM – PLANEJAMENTO FATORIAL PARA EXTRAÇÃO DAS CASCAS

FATOR	MÍNIMO	MÁXIMO
Tempo de extração (A)	10 min.	30 min.
% de etanol em água (B)	50	90
Tamanho de partícula do pó - mesh (C)	> 60	< 80
Temperatura de extração (D)	25 °C	45 °C
Massa da amostra (E)	100 mg	200 mg

Os resultados plotados no gráfico de Pareto, FIGURA 34, permitem inferir forte influência dos fatores tamanho de partícula, % de etanol em água, temperatura e tempo de extração, com $p < 0,05$. A influência muito grande do tamanho da partícula na extração está associada à característica do material fibroso lignificado constituinte das cascas da planta, o que impede a entrada do solvente nas células e a conseqüente extração do seu conteúdo. Logo menores tamanhos de partículas aumentam a superfície de contato da amostra, permitindo maior possibilidade de penetração do solvente nas células.

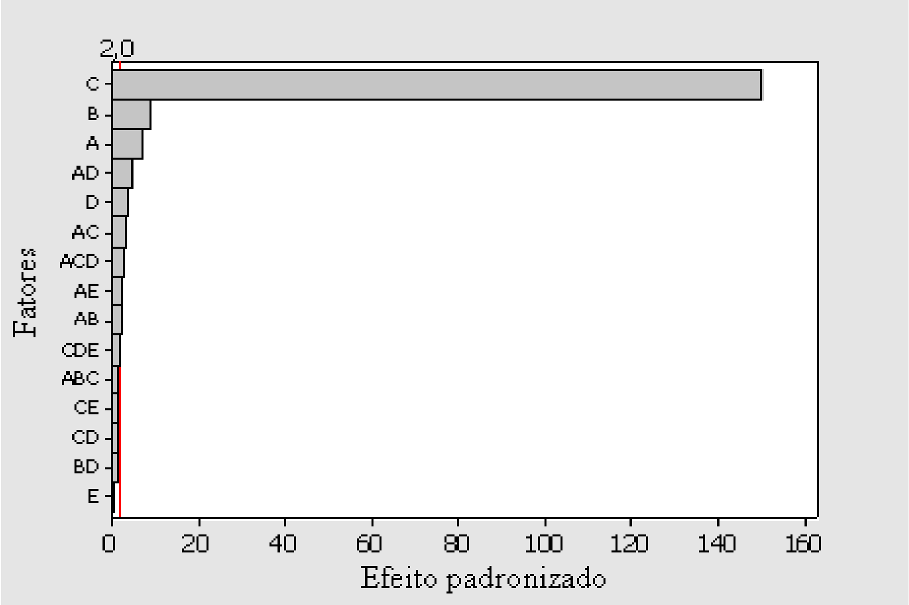

FIGURA 34 – GRÁFICO DE PARETO PARA AS CONDIÇÕES EXTRATIVAS DAS CASCAS

LEGENDA: A: Tempo de extração, B: % de Etanol em água, C: Tamanho de partícula do pó, D: Temperatura de extração, E: Massa da amostra.

O gráfico dos efeitos principais, FIGURA 35, torna evidente melhores valores de extração dos AO para tempos próximos a 30 min, menores quantidades de etanol em água como solvente extrator, partículas com granulometria < 80 mesh e temperatura de extração próxima a 45 ^{o}C.

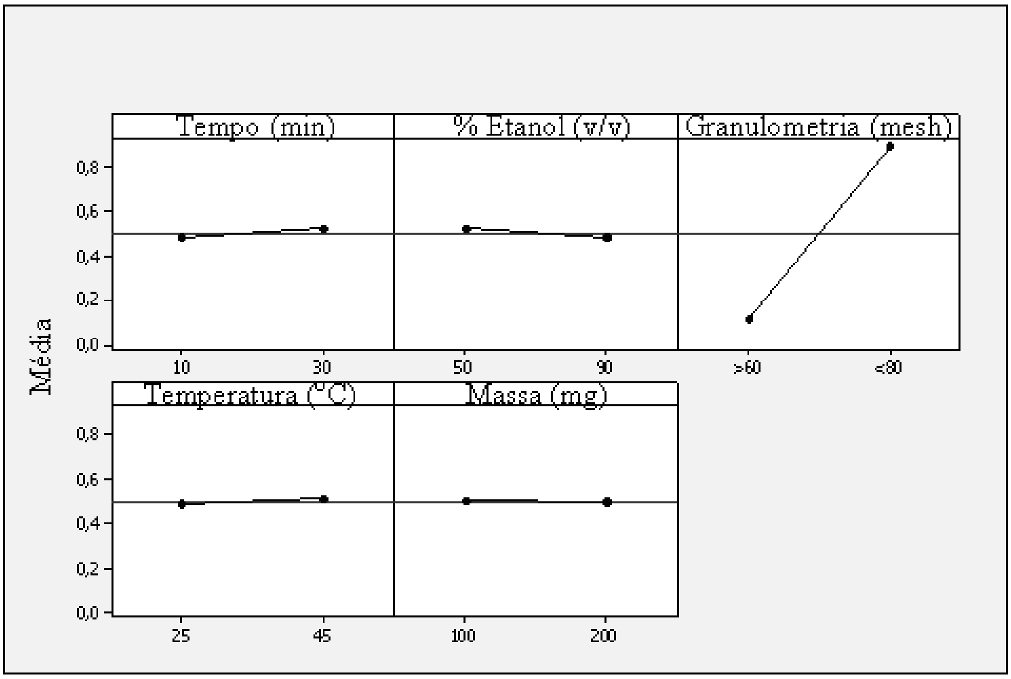

FIGURA 35 – EFEITOS PRINCIPAIS PARA AS CONDIÇÕES EXTRATIVAS DAS CASCAS

Conforme observado em ambos os gráficos a massa de amostra utilizada não interfere de maneira estatisticamente significativa no teor final obtido de AO, assim foi estabelecida a massa de 200 mg para análise em função de 100 mg de amostra ter apresentado áreas muito pequenas, próximas ao LQ, para os alcalóides minoritários especiofilina e uncarina F.

Para o fator tamanho da partícula foi estabelecido como valor ótimo para extração dos AO, partículas das cascas inferiores a 80 mesh. Isso pôde ser estabelecido uma vez que, após todas as condições otimizadas, testes em triplicata de amostras em partículas inferiores a 120 mesh não terem apresentado resultados estatisticamente diferentes no teor de AO quando comparadas a amostras com partículas inferiores a 80 mesh.

Os demais fatores foram otimizados pela aplicação de design composto central com design fatorial (2^3) composto por doze pontos axiais e dezesseis replicatas no centro do delineamento, TABELA 15.

TABELA 15 – ETAPA DE OTIMIZAÇÃO – PLANEJAMENTO FATORIAL PARA EXTRAÇÃO DAS CASCAS

FATOR	DOMÍNIO EXPERIMENTAL				
	$-\alpha^a$	-1	0	1	α^a
Tempo de extração (A)	20	22	25	28	30
% de Etanol em água (B)	30	38	50	62	70
Temperatura de extração (D)	30	33	38	42	45

Os gráficos de superfície de resposta apresentados suportam como melhores regiões para as condições extrativas os valores próximos às tonalidades de verde mais intenso.

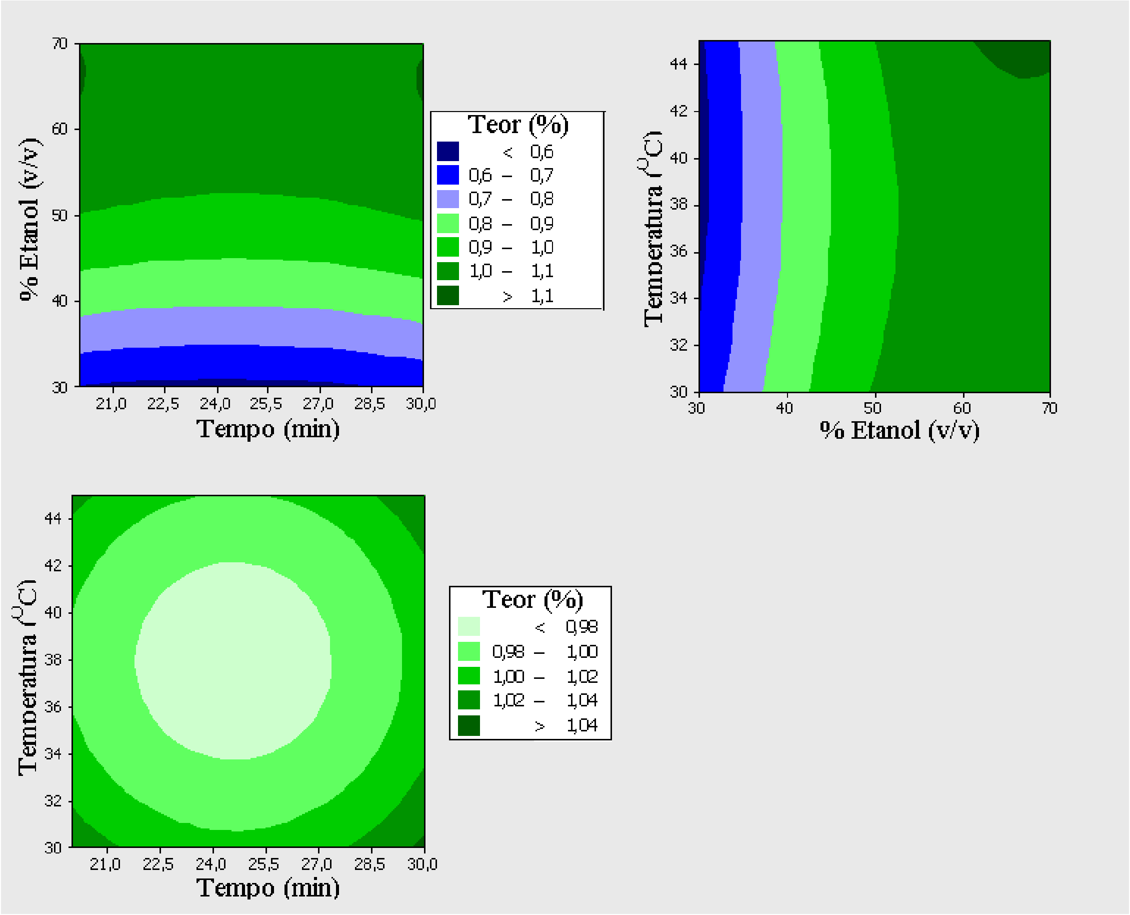

FIGURA 36 – SUPERFÍCIE DE RESPOSTA PARA OTMIZAÇÃO DA EXTRAÇÃO DAS CASCAS

O perfil atípico do gráfico de interação Temperatura Vs Tempo pode ser justificado pelo erro experimental, pois pequena diferença entre os valores mínimos (<0,98) e máximos (>1,04) é observada.

Fica evidente pela análise dos gráficos que dentro dos valores explorados para o domínio experimental o fator que rege predominantemente a extração é a

porcentagem de etanol utilizada como solvente extrator. Com esse fator devidamente otimizado, não existe diferença significativa na aplicação de 30 e 20 min no tempo ou 30 e 45 ^{o}C na temperatura de extração do método. Dessa forma os menores valores de temperatura e tempo foram selecionados como condições ótimas para remoção dos AO das cascas de *U. tomentosa*, já como solvente extrator foi estabelecido etanol 60% para evitar a extração de outros componentes menos polares da planta e melhor simular a força da fase móvel.

Finalmente os fatores otimizados foram confrontados com o solvente extrator metanol e três etapas extrativas, condições citadas por Ganzera e colaboradores (2004) e utilizadas pela USP XXXI (2008), no intuito de se verificar se essas condições resultavam em maiores teores de AO extraídos da planta. Os resultados demonstraram que com os parâmetros otimizados, os teores de AO são estatisticamente semelhantes aos obtidos com os fatores descritos em literatura, quando avaliados pelo teste de Tukey com 95% de confiança.

5.3.2 Preparo da amostra tintura

Como os alcalóides encontravam-se solúveis e disponíveis para análise nessa matriz, foi necessária apenas a adequação da concentração da amostra para que as áreas dos alcalóides ficassem contidas entre os pontos mínimo e máximo da curva analítica. Foi estabelecida diluição de 2 mL de tintura ao volume final de 5 mL com o solvente previamente utilizado na análise do pó, etanol 60% (v/v).

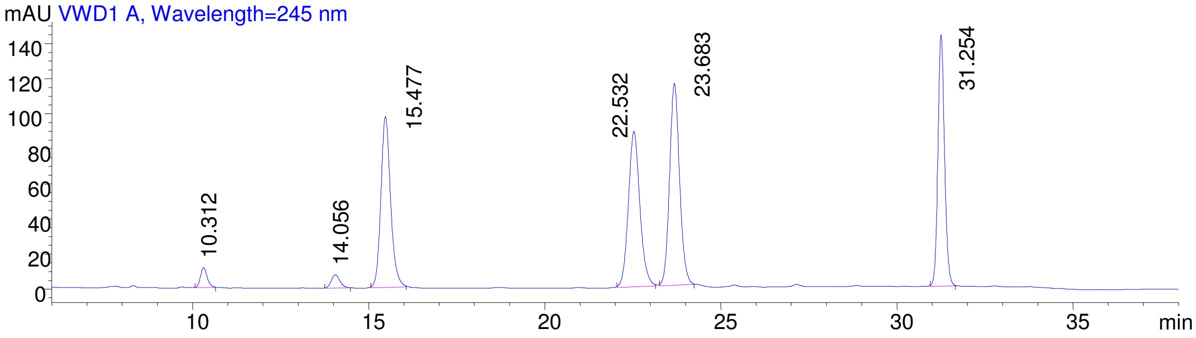

FIGURA 37 – PERFIL CROMATOGRÁFICO DA TINTURA DE *U. tomentosa*

NOTA: especiofilina (10,31 min), uncarina F (14,06 min), mitrafilina (15,48 min), isomitrafilina (22,53 min), pteropodina (23,68 min) e isopteropodina (31,25 min)

5.3.3 Preparo da amostra gel

As principais etapas críticas para o desenvolvimento de um método por MSPD foram avaliadas na seguinte seqüência: 1) natureza do sorbente para mistura com amostra; 2) solvente para limpeza da amostra; 3) relação sorbente : amostra; 4) solvente para eluição final das moléculas alvo.

O objetivo principal no desenvolvimento da metodologia extrativa foi remover os interferentes oleosos e os conservantes utilizados na formulação do gel.

Conforme descrito por Rowe, Sheskey e Weller (2003) os conservantes metil e propil parabeno possuem pKa = 8,4, valor significativamente diferente dos relatados para AOP por Laus e colaboradores (1996) como 5,7 a 6,7 e 4,7 por Chan, Morsingh e Yeoh (1966). Tendo em vista essa diferença de pKa e sabendo-se que o nitrogênio heterocíclico dos alcalóides possui grande afinidade de interação com óxido de alumínio, foram testados como sorbentes OALB e OALN. Outra característica muito importante neste caso é capacidade do óxido de alumínio adsorver água, pois 85% da matriz do gel é composta por esse solvente.

Como o OALB em presença de água confere pH 9,5 à solução, o uso deste foi descartado logo após os primeiros testes, pois causou efeito nivelador na eluição das moléculas dos conservantes e AOP, não permitindo seletividade na extração. Já o OALN confere pH 7,0 à solução em presença de água, desta maneira os AOP em sua forma molecular poderiam ser removidos da coluna com solvente orgânico apropriado.

A escolha do solvente de limpeza foi efetuada tendo-se em vista que metil e propil parabeno são consideravelmente solúveis em éter etílico e que os demais componentes oleosos da amostra tem grande afinidade pelo solvente hexano. Desta maneira foram avaliadas as proporções de hexano : éter etílico (5:5; 7:3 e 8:2) em diferentes volumes (20, 30, 40, 50 e 80 mL). As quantidades mais elevadas de éter na mistura de limpeza foram capazes de remover também os alcalóides, assim foi escolhida e menor proporção de éter na mistura em um volume de 40 mL para limpeza da amostra.

Para fixar a melhor proporção OALN : gel, partiu-se da relação 1:4 citada por Barker (2007) como a mais frequentemente utilizada na relação amostra : suporte sólido e testadas as proporções 1:4, 1:5 e 1:6. O ajuste foi realizado pela obtenção das misturas visualmente mais homogêneas e das melhores recuperações de AOP

obtidas. Foi verificada a necessidade do uso de OALN na base da coluna como mecanismo de evitar perda de alcalóides durante a etapa de limpeza, as quantidades de 1, 2, 3 e 4 g do óxido foram avaliadas para esse propósito. Para evitar respostas em CLAE muito baixas para os AOP, não foram utilizadas quantidades de gel inferiores a 5 g.

A mistura sorbente e gel foi executada em béquer com bastão de vidro, pois a mistura em gral e pistilo não resultou em maior homogeneidade da amostra.

Finalmente o solvente de eluição dos alcalóides foi definido com base na afinidade que os AOP possuem com acetato de etila e boa compatibilidade deste com OALN. O volume de 35 mL encontrado como ótimo condiz com os dados citados por Barker (2007) que descreve a eluição dos analitos de 0,5 g de amostra misturada com 2 g de sorbente nos primeiros 4 mL do solvente passado pela coluna, aproximadamente um volume de coluna.

Todas as etapas acima descritas não foram capazes de eliminar por completo os conservantes e o óleo da amostra, embora os tenham reduzido significativamente. Isso pode ser atribuído à maior concentração de propil parabeno e componentes oleosos em relação aos AOP no gel, 38 e 1625 vezes superior respectivamente. Dessa forma quando se tentava a eliminação desses componentes, acabavam por carrear os AOP presentes na ordem de ppm.

Assim separação completa dos AOP e parabenos foi obtida em CLAE conforme descrito no item 5.2.6.

A eliminação do óleo residual proveniente da amostra foi obtida pelo emprego de etanol 60% (v/v) como solvente para retomada da amostra após secagem do acetato de etila, em virtude de possuir baixa miscibilidade com o óleo. A centrifugação final permitiu melhor separação das fases e injeção da amostra límpida no cromatógrafo.

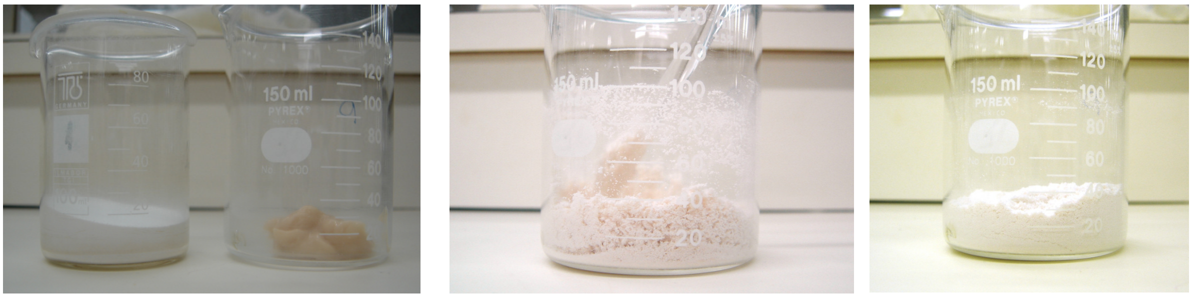

A B C

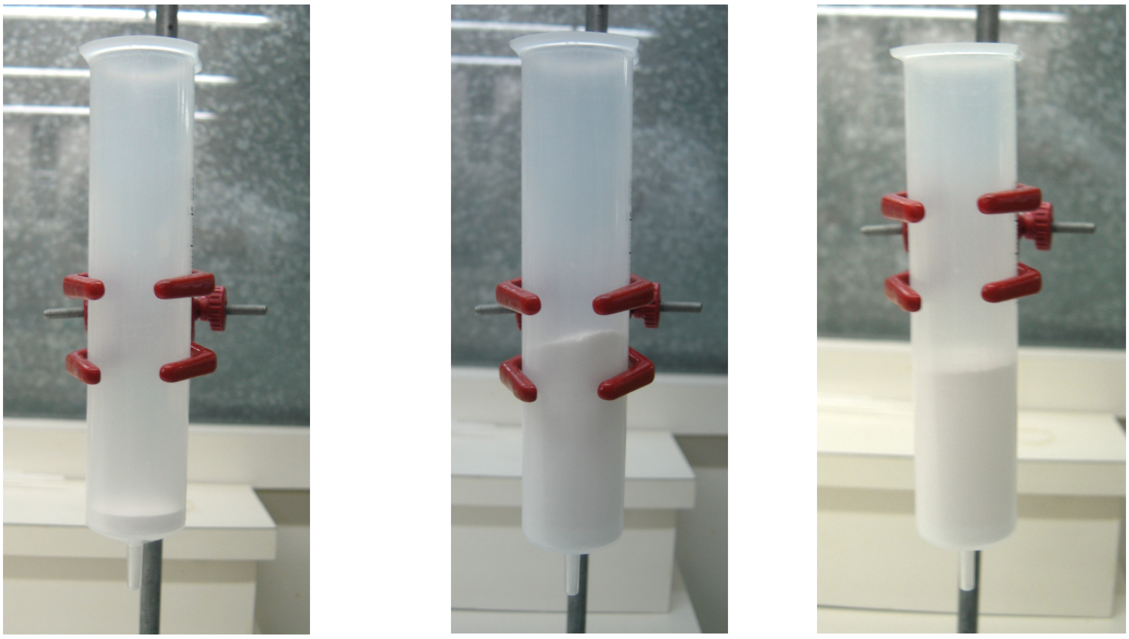

D E F

FIGURA 38 – DEMONSTRAÇÃO DAS ETAPAS DO PROCESSO DE PREPARO DA AMOSTRA GEL

LEGENDA : A) aspecto do sorbente e gel; B) etapa inicial da mistura; C) mistura finalizada; D) 3 g de OALN na base da coluna; E) introdução da amostra na coluna; F) coluna empacotada.

Embora não tenha sido conseguida completa limpeza da amostra em relação aos conservantes, os volumes de solvente utilizados foram bastante reduzidos, além do tempo de preparo da amostra em comparação às técnicas usualmente empregadas para amostras semi-sólidas, geralmente múltiplas etapas de agitação com solvente de extração.

5.4 VALIDAÇÃO DOS METODOS ANALÍTICOS

Os resultados para os ensaios de validação estão reunidos para cada amostra avaliada: cascas, tintura e gel. Os ensaios de perfil e pureza espectral estão reunidos para todas as amostras para permitir melhor comparação dos resultados. Como o método cromatográfico utilizado foi o mesmo nas cascas e tintura, os testes referentes às condições CLAE para linearidade com padrão, limites de detecção e quantificação e robustez para as duas amostras são apresentados em conjunto.

5.4.1 Comparação dos perfis espectrais

Em cada método cromatográfico os perfis espectrais dos picos de alcalóides foram comparados aos picos obtidos pela injeção de soluções dos padrões analíticos disponíveis de AOP, FIGURA 39.

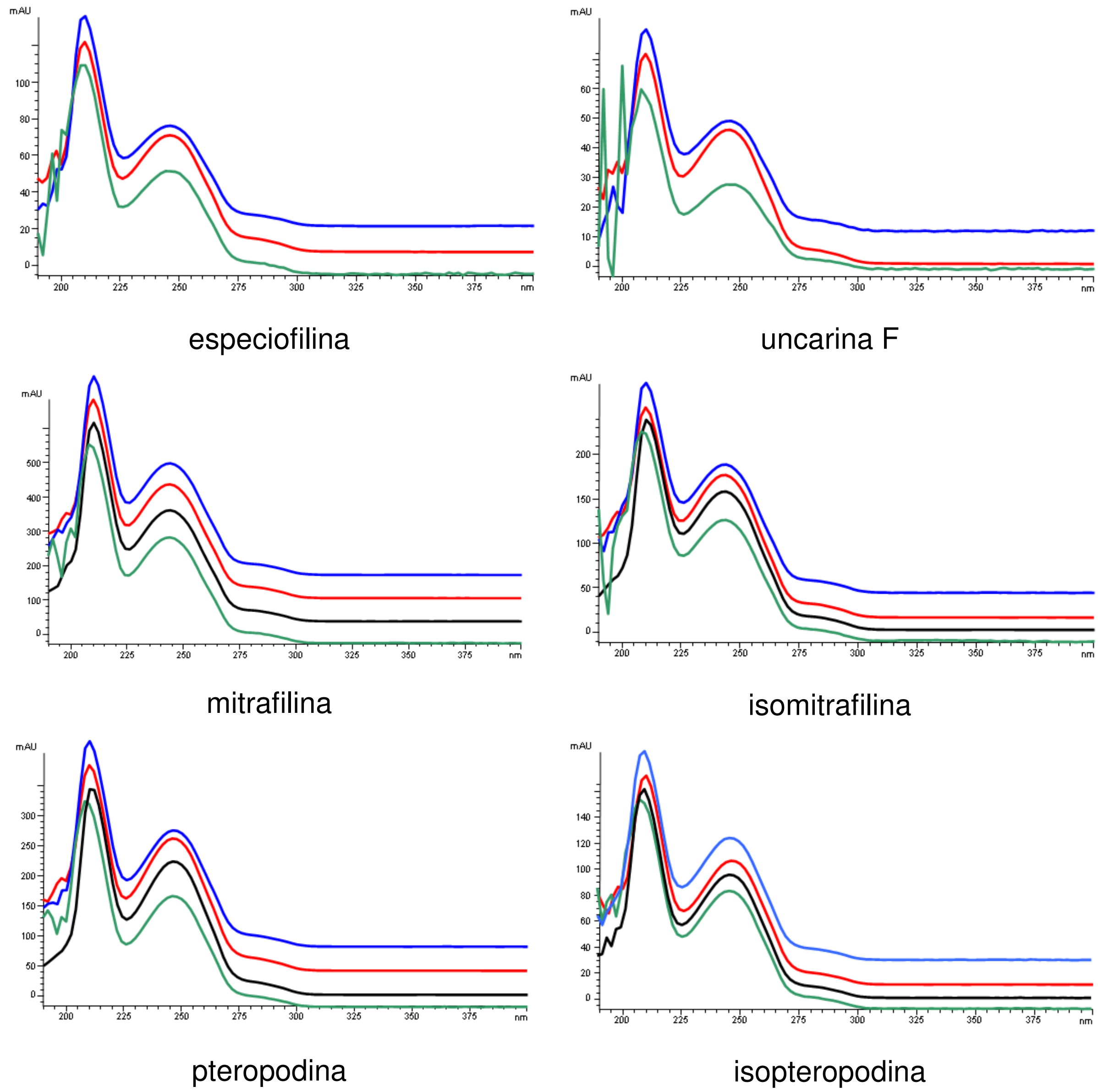

FIGURA 39 – PERFIS ESPECTRAIS DOS PICOS DE AOP ANALISADOS

LEGENDA: Azul – cascas; Vermelho – tintura; Preto – padrão; Verde - gel

Os perfis espectrais semelhantes das amostras em relação aos padrões constituem forte indicativo da identidade dos picos analisados.

Embora não tenham sido comparados a padrões analíticos, os perfis da especiofilina e uncarina F são muito semelhantes aos demais alcalóides oxindólicos, indicando pertencerem à mesma classe química.

5.4.2 Avaliação da pureza de pico

A pureza de pico determinada com utilização de DAD, avalia a sobreposição de vários espectros adquiridos no pico de interesse. Um alto fator de similaridade, em uma escala de 0 a 1000, indica inexistência de interferentes co-eluindo ao analito.

TABELA 16 – PUREZA DE PICO DOS ALCALÓIDES PARA AVALIAÇÃO DA SELETIVIDADE

		PUREZAS DOS PICOS					
AMOSTRA	TRATAMENTO	1	2	3	4	5	6
Cascas	Não degradado	998,816	996,850	994,050	972,122	881,842	999,677
	Ácido	877,516	857,812	990,656	845,093	952,382	939,268
	Base	974,111	938,312	858,237	997,397	900,657	852,041
	Peróxido	884,491	792,516	995,856	979,799	998,688	999,714
Tintura*	Não degradado	996,742	996,991	998,817	937,581	995,791	933,649
Gel	Não degradado	999,821	999,859	998,107	999,576	998,681	999,571
	Ácido	999,800	999,898	998,084	999,615	998,776	999,645
	Base	999,911	999,891	998,607	999,479	998,699	999,291
	Peróxido	999,847	999,893	998,257	999,626	998,873	999,655
Padrões (CLAE pó)	Não degradado	N.A.	N.A.	985,863	995,789	998,739	999,841
Padrões (CLAE gel)	Não degradado	N.A.	N.A.	940,909	997,222	993,903	992,518

NOTA: 1 – especiofilina; 2 – uncarina F; 3 – mitrafilina; 4 – isomitrafilina; 5 – pteropodina; 6 – isopteropodina; N.A. – não aplicável;
*Os tratamentos de degradação para a tintura não foram realizados devido à semelhança da amostra em relação às cascas.

Os valores apresentam bons resultados para pureza de pico em todas as condições analisadas. As condições de degradação testadas conforme Shabir (2003) reduziram os teores de pureza apenas para a amostra pó, no entanto os teores podem ser considerados elevados, semelhantes até aos padrões analíticos. As resoluções entre todos os picos ficaram acima de 1,5, critério utilizado pelo mesmo autor para manter a confiabilidade analítica.

Nenhuma das condições de degradação realizada conduziu ao surgimento de picos estranhos à amostra, indicando não terem sido formados compostos de degradação. O fato pode apresentar duas explicações plausíveis: a capacidade dos alcalóides permanecerem estáveis mesmo frente aos parâmetros utilizados ou as condições testadas terem sido muito brandas para as amostras analisadas.

5.4.3 Condições cromatográficas das cascas e tintura

5.4.3.1 Linearidade com padrão

O teste de linearidade com padrão permite avaliar a concordância da resposta do sistema cromatográfico frente a diferentes concentrações do analito de interesse. O INMETRO (2007) denomina este ensaio como “escolha da faixa linear de trabalho”, e estabelece que a concentração mais esperada da amostra deva, sempre que possível, se situar no centro da faixa de trabalho.

Três curvas analíticas com concentrações semelhantes foram analisadas em dias diferentes, total de 15 pontos. O coeficiente de correlação (r) apresentado na FIGURA 40 é superior ao exigido pela ANVISA (0,99) (BRASIL, 2003).

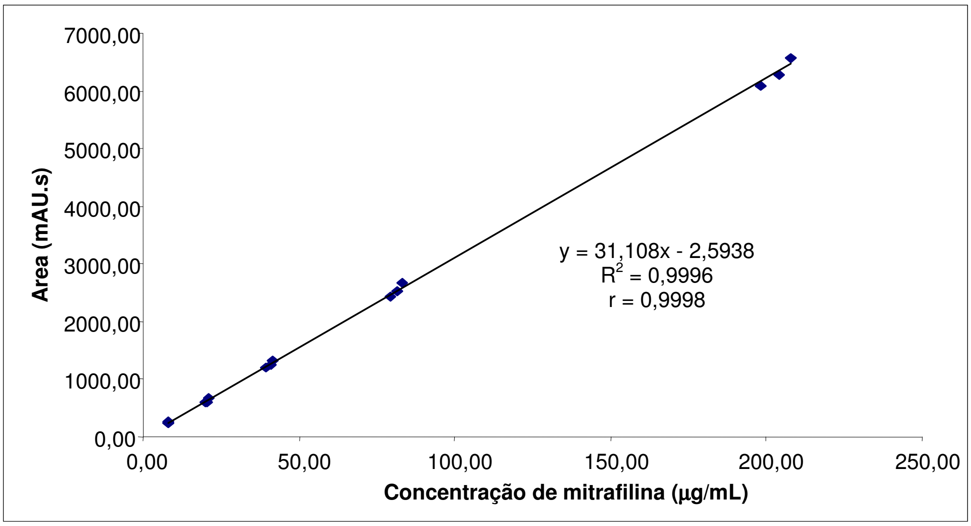

FIGURA 40 – LINEARIDADE COM PADRÃO MITRAFILINA, MÉTODO CLAE CASCAS E TINTURA

Além disso, segundo Ribani e colaboradores (2004) um coeficiente de correlação maior que 0,999 é considerado como evidência de um ajuste ideal dos dados para a linha de regressão. Para esta análise o método apresentou resposta linear na faixa de 8,32 a 208,00 $\mu g.mL^{-1}$ de mitrafilina.

5.4.3.2 Limite de detecção

Conforme descrito pelo ICH (2005) e Brasil (2003), a inclinação e o desvio encontrado para uma curva analítica injetada foram utilizados na Equação 2, item 3.5.4, e encontrado o valor de 1,00 μg.mL^{-1} de mitrafilina para LD.

Para confirmação desse valor a solução do padrão analítico foi diluída até a concentração calculada e analisada. Esse ponto inserido na curva analítica anterior forneceu novos valores de inclinação e desvio que inseridos na Equação 2 geraram limite de 0,80 μg.mL^{-1} de mitrafilina confirmando este valor como LD, FIGURA 41.

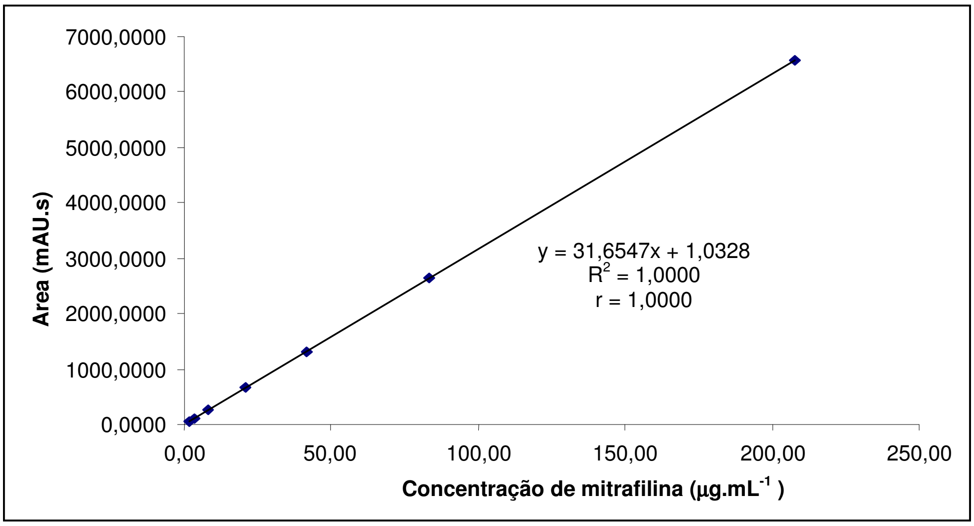

FIGURA 41 – CURVA ANALÍTICA PARA CÁLCULO DO LD PARA CASCAS E TINTURA

5.4.3.3 Limite de quantificação

Os resultados do desvio e inclinação da mesma curva analítica do item anterior inseridos na Equação 3 geraram o valor de 2,90 μg.mL^{-1} de mitrafilina para LQ. A diluição do padrão até a concentração encontrada e posterior inclusão na curva analítica confirmou o valor de 2,40 μg.mL^{-1} de mitrafilina para LQ.

As sextuplicatas utilizadas na avaliação da precisão e exatidão no LQ para a mitrafilina, apresentaram valores de DPR de 4,28 e 3,24% respectivamente para cascas e tintura, resultados adequados comparados ao limite de 11% publicado para a concentração testada (TAVERNIERS; LOOSE; BOCKSTELE, 2004).

TABELA 17 – AVALIAÇÃO DO LQ PARA CASCAS E TINTURA

AMOSTRA	ÁREA (mAU.s)	RESULTADO ($\mu g.mL^{-1}$)	DPR (%)	RECUPERAÇÃO* (%)
Cascas (n=6)	136,790 ± 5,915	4,07 ± 0,19	4,28	104,13
Tintura (n=6)	79,990 ± 2,510	2,81 ± 0,09	3,24	101,91

NOTA: *Calculada considerando o valor médio de 3,91 e 2,75 $\mu g.mL^{-1}$ para a mitrafilina, obtido na avaliação da repetibilidade das cascas e tintura respectivamente.

As recuperações entre 101,91 e 104,13% estão contidas dentro do limite de 80 a 110% descrito por Huber (2001) como aceitável para as concentrações avaliadas.

5.4.3.4 Robustez das condições cromatográficas CLAE para cascas e tintura

Para este teste foram considerados os valores obtidos na planejamento fatorial de experimentos do método CLAE. Segundo Chandran e Singh (2007) os parâmetros avaliados na robustez podem ser analisados um fator por vez ou simultaneamente como parte de um experimento fatorial.

Os gráficos de superfície de resposta já apresentados no item 5.2.1 permitem afirmar a robustez do método cromatográfico em manter resolução > 1,5, entre os pares de picos problema isomitrafilina – pteropodina e uncarina F – mitrafilina para pH de tampão entre 6,6 e 8,4 e temperatura da coluna entre 10 e 20 ^{o}C. Além disso, é possível considerar o método robusto para o tipo de tampão empregado, fosfato ou acetato.

O limite de 1,5 foi escolhido para atestar a robustez do método frente à resolução dos picos, pois Ciola (1998) e Collins (2006) citam que resoluções acima deste valor garantem separação completa entre os picos, boa integração e eficiência na quantificação.

Amostras preparadas em triplicata foram analisadas em diferentes colunas (item 4.9.7) para avaliar a interferência desse fator na análise.

Embora a coluna Hypersil tenha apresentado os piores valores de simetria para os picos avaliados, a resolução entre os sinais não foi prejudicada significativamente, logo foi possível a adequada integração de todos os picos. Todos os resultados de teor não são significativamente diferentes quando avaliados pelo

teste-t com 95% de confiança. Na TABELA 18 são apresentados apenas os resultados para os picos críticos mitrafilina e pteropodina.

TABELA 18 – RESULTADOS OBTIDOS COM AS DIFERENTES COLUNAS TESTADAS NA AVALIAÇÃO DA ROBUSTEZ PARA CLAE DAS CASCAS E TINTURA

		MITRAFILINA[1]		PTEROPODINA[1]	
COLUNAS (n=3)	TEOR DE ALCALÓIDES (%)[1]	RESOLUÇÃO[2]	SIMETRIA	RESOLUÇÃO[3]	SIMETRIA
Luna C-18 (2)	1,06 ± 0,01	3,04	0,87	3,04	0,87
Hipersil	1,06 ± 0,01	2,84	0,78	2,84	0,78
Zorbax Eclipse XDB	1,05 ± 0,01	2,51	0,90	2,51	0,90
Zorbax Eclipse XDB (controle)	1,05 ± 0,02	3,25	0,91	2,17	0,94

Nota: [1]Valores médios; [2]em relação ao pico da uncarina F; [3]em relação ao pico da isomitrafilina.

Os piores resultados encontrados para a coluna Hypersil para simetria são explicados pela pior tecnologia na obtenção das partículas de sílica, demonstrado pelo maior diâmetro médio de poro (120 Å), em relação às demais, item 4.9.7. Os valores acima permitem atestar que a eficiência e seletividade do método foram mantidas mesmo quando utilizadas as diferentes colunas, consequentemente o método permaneceu robusto frente às colunas avaliadas.

5.4.4 Validação do método para cascas de *U. tomentosa*

5.4.4.1 Seletividade / especificidade

O gráfico obtido no método da adição de padrão para avaliação do efeito matriz é apresentado na avaliação da exatidão da metodologia (item 5.4.4.5). O valor de 30,44, obtido para o coeficiente angular da curva adicionada de padrão apresenta pouco desvio, 1,08%, em relação ao coeficiente angular de 30,77, obtido para a curva analítica. Segundo Ribani e colaboradores (2004) a similaridade dos coeficientes angulares indica ausência de interferência da matriz na análise.

Os perfis de fragmentação e espectros de massa já apresentados (item 5.2.2) constituem evidência de que os sinais obtidos no cromatograma são resultados de AO e não possuem interferentes co-eluindo.

5.4.4.2 Linearidade e intervalo

Embora não seja descrito em literatura, a avaliação da linearidade com a amostra melhor representa o analito em seu ambiente químico. Em produtos de origem vegetal os diferentes componentes da matriz podem causar interferências que não são detectadas quando a avaliação se faz com o padrão analítico. Desta forma, a linearidade acaba avaliando o método como um todo, processo de preparo de amostra e cromatográfico.

Como se trata de material vegetal, portanto sem padronização, a escolha da faixa de 50 a 150% da concentração visou abranger a maior variabilidade possível do teor de alcalóides na planta, 105 a 315 μg de alcalóides em 1 mL de solução injetada (THOMPSON; ELLISON; WOOD, 2002). O gráfico plota o somatório da área dos alcalóides (mAU.s) Vs concentração (μg.mL^{-1}), FIGURA 42.

O valor apresentado para o coeficiente de correlação é superior ao exigido pela ANVISA (0,99), embora seja inferior ao citado como ideal pelo FDA (0,999). (FDA, 2001; BRASIL, 2003). No entanto, segundo Pimentel e Neto (1996) a linearidade não deve ser avaliada apenas pelo coeficiente de correlação, a análise do gráfico de resíduos segundo esses autores é de suma importância para predizer tendências no método.

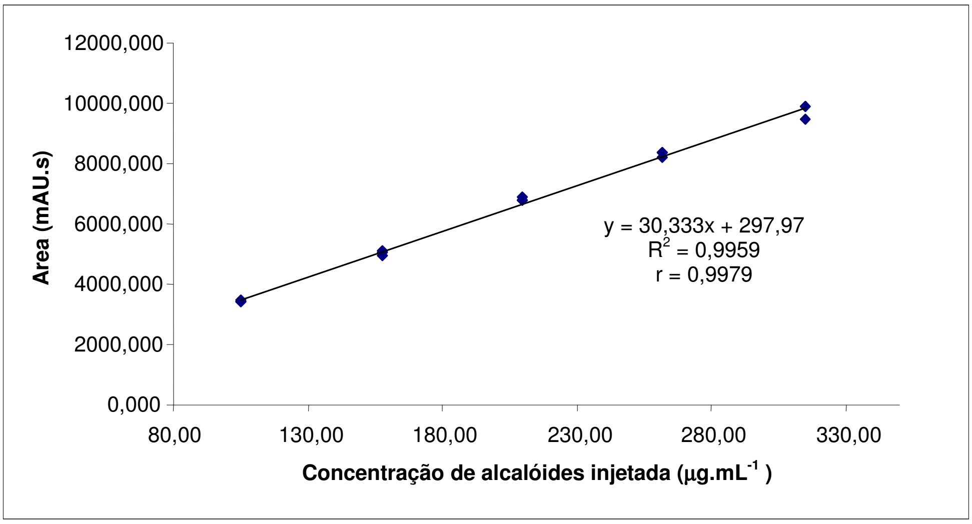

FIGURA 42 – LINEARIDADE – VALIDAÇÃO DAS CASCAS DE *U. tomentosa*

O teste de resíduos conforme descrito por Taverniers, Loose e Bockstaele (2004), representa a diferença entre os valores de y encontrados e os preditos pela

equação de regressão gerada. Se os valores de resíduos são randomicamente distribuídos, a linearidade é confirmada.

O gráfico de resíduos gerado com os dados da linearidade demonstrou valores randomicamente dispersos ao longo do eixo y, comprovando assim a linearidade do método, FIGURA 43.

É preciso considerar que todas as concentrações de cada alcalóide separadamente estão contidas na faixa apresentada no item 5.4.3.1. Apenas pelo somatório dessas concentrações, FIGURA 42, é observada a extrapolação da faixa da curva analítica.

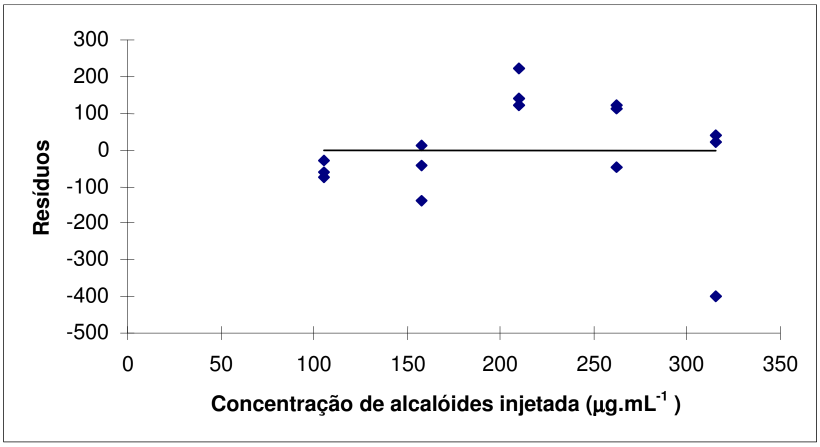

FIGURA 43 – DISPERSÃO DOS RESÍDUOS – VALIDAÇÃO DAS CASCAS DE *U. tomentosa*

5.4.4.3 Repetibilidade

O teste de precisão intra-dia, avaliado com seis amostras na mesma concentração apresentou os resultados da TABELA 19.

TABELA 19 – REPETIBILIDADE – VALIDAÇÃO DAS CASCAS DE *U. tomentosa*

REPLICATA	ÁREA (mAU.s)	RESULTADO CROMATOGRAMA (μg.mL^{-1})	TEOR DE AOP NAS CASCAS (%)
1	6705,031	211,62	1,05
2	6843,541	215,99	1,07
3	6664,292	210,33	1,04
4	6790,553	214,32	1,07
5	6808,684	214,89	1,07
6	6891,933	217,52	1,09
Média	N.A.	N.A.	1,07
DP	N.A.	N.A.	0,02
DPR (%)	N.A.	N.A.	1,45

NOTA: DP – desvio padrão; DPR – desvio padrão relativo; N.A. – não aplicável

Além da confirmação da repetibilidade com amostras a 100% da concentração teórica do teste, a avaliação em triplicata para cada concentração no teste de linearidade, item 5.4.4.2, permitiu a confirmação da repetibilidade também nas concentrações 50 e 150%. Para a concentração 50% o valor de DPR para a triplicata analisada foi de 0,68%, já a triplicata da concentração 150% apresentou DPR de 2,56% em relação à somatória das áreas dos alcalóides.

Os resultados confirmam o valor de DPR abaixo do limite estabelecido pela ANVISA como 5% (BRASIL, 2003). Os valores encontrados também são inferiores ao limite mais restritivo de 2,7% citado por Huber (2001).

5.4.4.4 Precisão intermediária

A precisão intermediária foi avaliada com os resultados das análises de dois analistas diferentes em dias diferentes e utilização do mesmo equipamento.

A TABELA 20 contém valor de DPR inferior a 5%, limite estabelecido pela ANVISA para o teste.

TABELA 20 – PRECISÃO INTERMEDIÁRIA – VALIDAÇÃO DAS CASCAS DE *U. tomentosa*

REPLICATA	ÁREA (mAU.s)	RESULTADO CROMATOGRAMA ($\mu g.mL^{-1}$)	TEOR DE AOP NAS CASCAS (%)
1	6705,031	211,62	1,05
2	6843,541	215,99	1,07
3	6664,292	210,33	1,04
4	6790,553	214,32	1,07
5	6808,684	214,89	1,07
6	6891,933	217,52	1,09
7	6736,296	210,33	1,05
8	6490,829	209,17	1,05
9	6587,630	204,57	1,02
10	6537,390	203,01	1,02
11	6506,613	202,03	1,01
12	6678,289	207,46	1,04
Média	N.A.	N.A.	1,05
DP	N.A.	N.A.	0,02
DPR (%)	N.A.	N.A.	2,29

NOTA: DP – desvio padrão; DPR – desvio padrão relativo; N.A. – não aplicável

5.4.4.5 Exatidão

A indisponibilidade de obtenção da matriz, cascas de *U. tomentosa*, sem os analitos de interesse, AO, levou à necessidade de executar o teste de exatidão pelo procedimento da adição de padrão à amostra.

Conforme já explicado, item 4.9.6, as amostras sofreram adições de padrão segundo TABELA 21.

TABELA 21 – DADOS PARA O TESTE DA ADIÇÃO DE PADRÃO À AMOSTRA CASCAS DE *U. tomentosa*

	PADRÃO		AMOSTRA + PADRÃO	
PONTO	CONCENTRAÇÃO DE MITRAFILINA (μg.mL^{-1})	ÁREA (mAU.s)	CONCENTRAÇÃO DE MITRAFILINA ADICIONADA (μg.mL^{-1})	ÁREA (mAU.s)
1	7,92	238,338	0,00	2371,237
2	19,80	598,632	19,20	2975,555
3	39,60	1204,806	38,50	3576,248
4	79,20	2433,933	57,80	4153,534
5	198,10	6085,422	77,00	4713,653

Com os dados acima foram construídas as curvas pela correlação da Concentração de mitrafilina (μg.mL^{-1}) Vs Área (mAU.s).

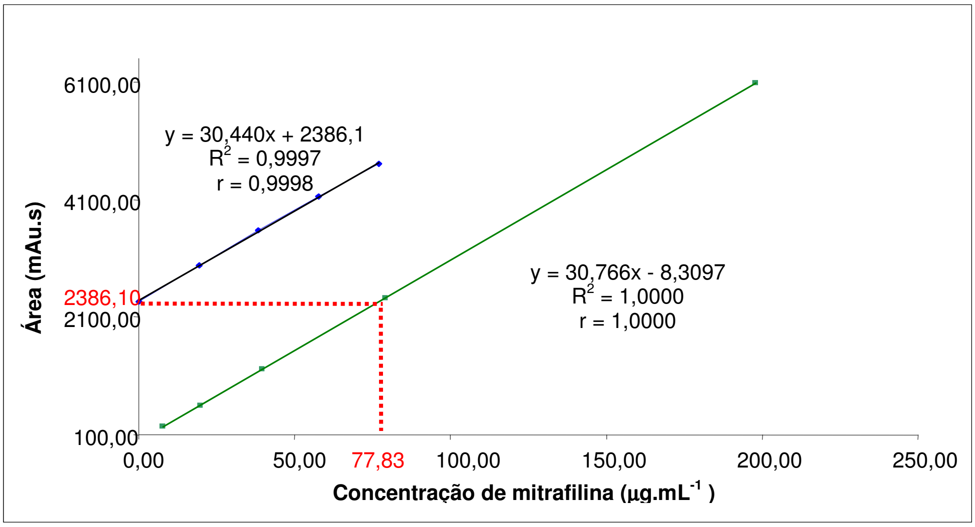

FIGURA 44 – AVALIAÇÃO DO EFEITO MATRIZ – VALIDAÇÃO DAS CASCAS DE *U. tomentosa*
LEGENDA: Azul – curva amostra + padrão; Verde – curva analítica

Como para a curva da amostra + padrão foram computadas apenas as concentrações adicionadas de mitrafilina, pode-se considerar que o ponto onde a

reta corta o eixo das ordenadas (ponto 1) corresponde à área do pico da mitrafilina que está sendo determinada, sem qualquer adição de padrão (indicada em vermelho). Assim, a extrapolação desse valor para a reta da curva analítica define, no eixo das abscissas, a concentração da substância na amostra analisada, como indicado na FIGURA 44.

Em termos matemáticos a inserção de 2386,1 mAU.s de área na equação da reta da curva analítica e posterior considerações das diluições efetuadas e tomada de amostra (200 mg), fornece o valor real da concentração de mitrafilina na amostra.

Equação da reta da curva padrão: $y = 30{,}766x - 8{,}3097$

$2386{,}1 = 30{,}766x - 8{,}3097$

$X = 77{,}83\ \mu g.mL^{-1}\ \therefore\ 77{,}83\ \mu g / 200\ mg = 0{,}39\%$ de mitrafilina nas cascas

O valor de 0,39% foi considerado o valor verdadeiro para a concentração de mitrafilina na amostra. A tomada de amostra das cascas como explicado no item 4.9.6, permitiu avaliar a recuperação nas concentrações 50, 100 e 150%, TABELA 22.

TABELA 22 – EXATIDÃO – VALORES DE RECUPERAÇÃO PARA OS AO DAS CASCAS DE *U. tomentosa*

NÍVEL (%)	CONCENTRAÇÃO ESPERADA DE MITRAFILINA (%)	ÁREA (mAU.s)	CONCENTRAÇÃO OBTIDA DE MITRAFILINA (%)	RECUPERAÇÃO MÉDIA ± DP (%)
		1241,828	0,197	
50	0,20	1243,515	0,197	98,43 ± 0,07
		1253,912	0,197	
		2421,887	0,381	
100	0,39	2430,098	0,383	98,37 ± 0,87
		2458,874	0,387	
		3621,649	0,569	
150	0,58	3492,593	0,549	98,89 ± 1,90
		3605,964	0,567	

Os valores de recuperação apresentados estão dentro da faixa de 95 – 105% citada por Huber (2001) como aceitável para as concentrações analisadas.

Como a mitrafilina é um alcalóide de polaridade intermediária em relação aos demais AO, os valores obtidos de recuperação para essa molécula podem ser melhor extrapolados para os demais alcalóides. Além disso, a USP XXXI (2008) e o

ICH (2005) estabelecem que a exatidão possa ser inferida uma vez que a precisão, linearidade e especificidade tenham sido demonstradas.

5.4.4.6 Robustez

Além dos testes já apresentados para avaliação do método CLAE, foi verificada a estabilidade das soluções da amostra após um e dois dias de preparo. A comparação foi efetuada em relação aos valores dos teores de AO (%) obtidos da análise realizada no dia do preparo das amostras, a execução do teste-t com 95% de confiança não apresentou diferenças significativas entre as médias dos resultados.

Os resultados do planejamento fatorial de experimentos, item 5.3.1, permitem apresentar a robustez do preparo de amostra, considerando teores > 1,00% de AO, para tempos de extração de 20 a 30 min, concentrações de etanol de 55 a 70% em água, temperatura de extração ente 30 e 45 ^{o}C e granulometria das cascas menores que 80 mesh.

5.4.5 Validação do método para tintura

5.4.5.1 Seletividade / especificidade

As curvas obtidas para padrão e amostra + padrão estão apresentadas no teste da exatidão, item 5.4.5.5.

Foi encontrado coeficiente angular de 30,89 para a curva analítica e 31,12 para a curva da amostra adicionada de padrão, correspondendo a um desvio de 0,72% de uma em relação à outra. Esse resultado permite confirmar a relação paralela entre as duas curvas, e admitir como inexistente a interferência da matriz na análise.

5.4.5.2 Linearidade e intervalo

A avaliação foi realizada com tomadas de amostra proporcionais da tintura de maneira a perfazer as concentrações 80, 90, 100, 110 e 120% de AO presentes na amostra, conforme descrito no item 4.9.2.

Os resultados da análise em triplicata de cada concentração foram plotados em gráfico Concentração de alcalóides injetada (μg.mL^{-1}) Vs Área dos picos (mAU.s), FIGURA 45.

O coeficiente de correlação obtido, r = 0,9989, é superior ao exigido pela ANVISA (0,99) e inferior ao exigido pelo FDA (0,999), embora esteja muito próximo a esse último. É de se considerar que o valor de r foi obtido com a análise de uma forma extrativa de planta, matriz muito mais complexa do que as soluções de padrões analíticos geralmente empregadas para execução destes testes (FDA, 2001; BRASIL, 2003).

Como a análise dos resíduos apresentou pontos aleatoriamente dispersos, FIGURA 46, é possível confirmar a capacidade do método em apresentar resultados lineares na faixa de 220 a 332 μg de AO / mL da solução injetada.

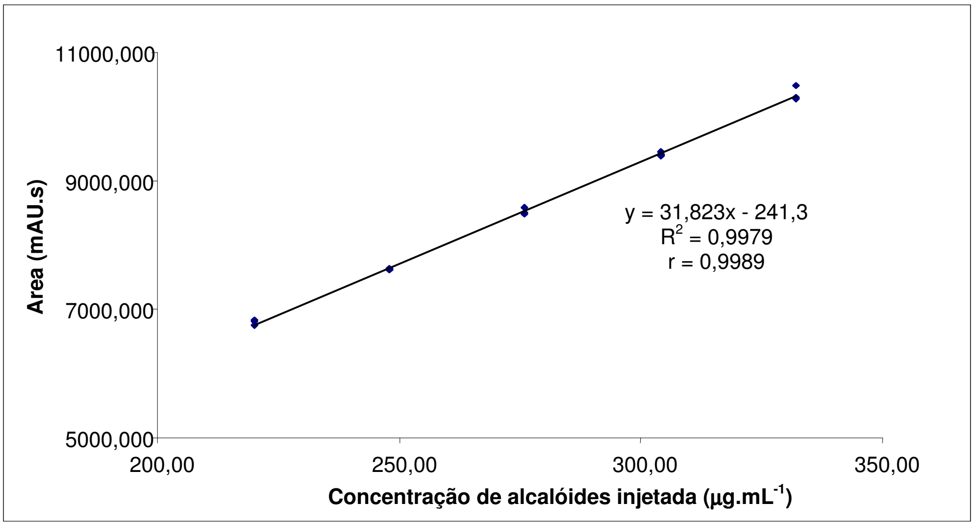

FIGURA 45 – LINEARIDADE – VALIDAÇÃO DA TINTURA

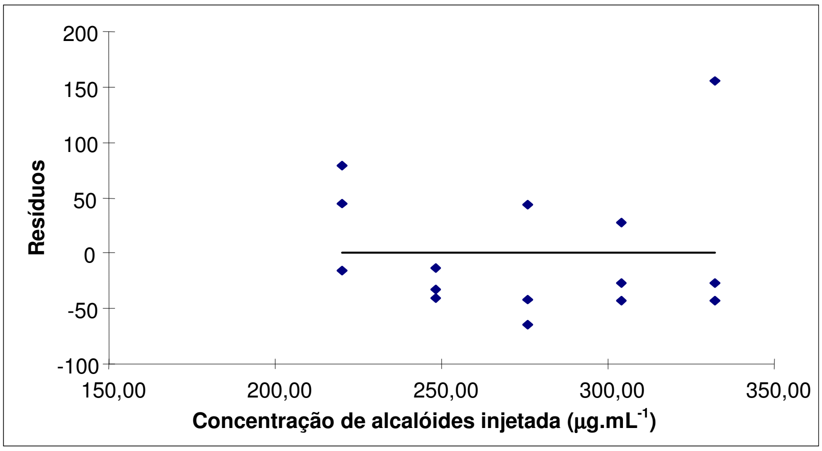

FIGURA 46 – DISPERSÃO DOS RESÍDUOS – VALIDAÇÃO DA TINTURA

5.4.5.3 Repetibilidade

O DPR de 0,66%, obtido do ensaio de seis amostras a 100% da concentração para teste da repetibilidade, permite afirmar que o resultado é inferior ao limite estabelecido de 5% para a concentração avaliada segundo os documentos consultados, TABELA 23 (HUBER, 2001, BRASIL, 2003).

TABELA 23 – REPETIBILIDADE – VALIDAÇÃO DA TINTURA

REPLICATA	ÁREA (mAU.s)	RESULTADO CROMATOGRAMA (µg.mL^{-1})	TEOR DE AOP NA TINTURA (µg.mL^{-1})
1	8585,211	279,30	698,25
2	8499,722	276,49	691,23
3	8604,825	279,89	699,74
4	8538,940	277,76	694,40
5	8471,737	275,58	688,96
6	8476,873	275,75	689,38
Média	N.A.	N.A.	*693,66*
DP	N.A.	N.A.	*4,58*
DPR (%)	N.A.	N.A.	*0,66*

NOTA: DP – desvio padrão; DPR – desvio padrão relativo; N.A. – não aplicável

Mais uma vez, o teste de linearidade, item 5.4.5.2, permitiu comprovar a precisão nos níveis extremos de concentração 80 e 120%. Para a concentração 80% o valor de DPR na triplicata analisada foi de 0,71%, já a triplicata da concentração 120% apresentou DPR de 1,07% em relação à somatória das áreas dos alcalóides.

5.4.5.4 Precisão intermediária

O teste realizado em dias e por analistas diferentes, forneceu DPR de 0,93% confirmando a precisão da metodologia.

TABELA 24 – PRECISÃO INTERMEDIÁRIA – VALIDAÇÃO DA TINTURA

REPLICATA	ÁREA (mAU.s)	RESULTADO CROMATOGRAMA ($\mu g.mL^{-1}$)	TEOR DE AOP NA TINTURA ($\mu g.mL^{-1}$)
1	8585,211	279,30	698,25
2	8499,722	276,49	691,23
3	8604,825	279,89	699,74
4	8538,940	277,76	694,40
5	8471,737	275,58	688,96
6	8476,873	275,75	689,38
7	8305,273	270,19	675,48
8	8473,685	275,65	689,12
9	8526,349	277,35	693,38
10	8443,292	274,66	686,66
11	8459,513	275,19	687,97
12	8412,295	273,66	684,15
Média	N.A.	N.A.	689,88
DP	N.A.	N.A.	6,44
DPR (%)	N.A.	N.A.	0,93

NOTA: DP – desvio padrão; DPR – desvio padrão relativo; N.A. – não aplicável

5.4.5.5 Exatidão

Assim como para as cascas, é impossível se obter a matriz isenta de AO para a tintura de *U. tomentosa.* Portanto, foi utilizado o método de adição de padrão à amostra para teste da exatidão.

Como a mitrafilina é um alcalóide de média polaridade em relação aos demais AO, seu comportamento no método CLAE pode, com maior segurança, ser estendido para confirmação da recuperação dos AO presentes na amostra.

Os dados para construção das curvas, conforme apresentado no item 4.9.6, encontram-se na TABELA 25.

TABELA 25 – DADOS PARA O TESTE DA ADIÇÃO DE PADRÃO À AMOSTRA TINTURA

	PADRÃO		AMOSTRA + PADRÃO	
PONTO	CONCENTRAÇÃO DE MITRAFILINA (µg.mL^{-1})	ÁREA (mAU.s)	CONCENTRAÇÃO DE MITRAFILINA ADICIONADA (µg.mL^{-1})	ÁREA (mAU.s)
1	8,16	238,293	0	1817,501
2	20,40	610,505	20,40	2491,867
3	40,80	1251,640	40,80	3101,458
4	81,60	2537,048	81,60	4406,845
5	204,00	6285,140	122,40	5626,736

Com os resultados das análises cromatográficas, apresentados acima, foram construídas as curvas analítica e da amostra + padrão através da correlação da Concentração de mitrafilina (µg.mL^{-1}) Vs Área (mAU.s), FIGURA 47.

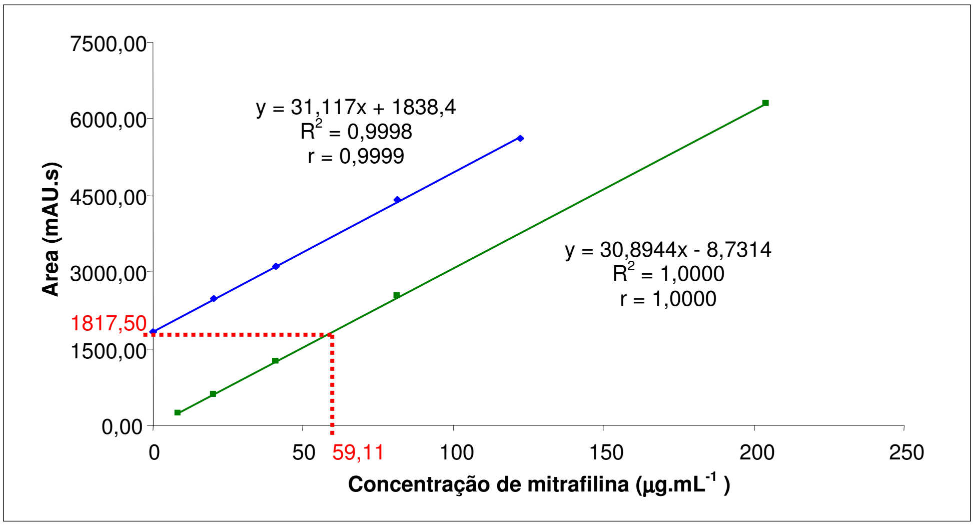

FIGURA 47 – AVALIAÇÃO DO EFEITO MATRIZ – VALIDAÇÃO DA TINTURA
LEGENDA: Azul – curva amostra + padrão; Verde – curva analítica

Conforme já exposto, item 5.4.4.5, o ponto da reta da amostra + padrão que corta o eixo das ordenadas (ponto 1) corresponde à área do pico da Mitrafilina que está sendo determinada, sem qualquer adição de padrão (indicada em vermelho). Pela extrapolação desse valor para a reta da curva analítica foi encontrado, no eixo das abscissas, o valor de 59,11 µg.mL^{-1} para a concentração de mitrafilina injetada. Considerando as diluições efetuadas a quantidade de mitrafilina presente na tintura é de 148,00 µg.mL^{-1}.

O valor de 148,00 µg.mL^{-1} foi considerado o valor esperado para o nível 100% no cálculo da recuperação. A tomada de amostra da tintura como explicado no item 4.9.6, permitiu avaliar a recuperação nas concentrações 80, 100 e 120%. A

faixa de 80 a 120% foi escolhida em conformidade com ANVISA, ICH e USP XXXI, por se tratar de amostra possível de ser padronizada. (BRASIL, 2003; ICH, 2005; USP, 2008).

TABELA 26 – EXATIDÃO – VALORES DE RECUPERAÇÃO PARA OS AO DA TINTURA

NÍVEL (%)	CONCENTRAÇÃO ESPERADA DE MITRAFILINA (µg.mL^{-1})	ÁREA (mAU.s)	CONCENTRAÇÃO OBTIDA DE MITRAFILINA (µg.mL^{-1})	RECUPERAÇÃO MÉDIA ± DP (%)
		1456,319	118,419	
80	118,00	1447,860	117,734	100,38 ± 0,61
		1465,766	119,184	
		1890,359	153,561	
100	148,00	1889,761	153,513	102,45 ± 2,24
		1819,261	147,805	
		2194,707	178,204	
120	177,00	2189,088	177,749	101,09 ± 0,95
		2227,544	180,862	

Os valores de recuperação apresentados, TABELA 26, estão dentro da faixa de 90 – 107% apresentada por Huber (2001) como aceitável para as concentrações analisadas.

5.4.6 Validação do método para o gel

5.4.6.1 Seletividade / especificidade

Frente à possibilidade de se obter a matriz na ausência dos analitos de interesse, foi avaliada a possível existência de interferentes provenientes desta na detecção e quantificação dos alcalóides. Assim, a análise do placebo foi confrontada a uma amostra, FIGURA 48, para observar co-eluição nos tempos de retenção dos alcalóides.

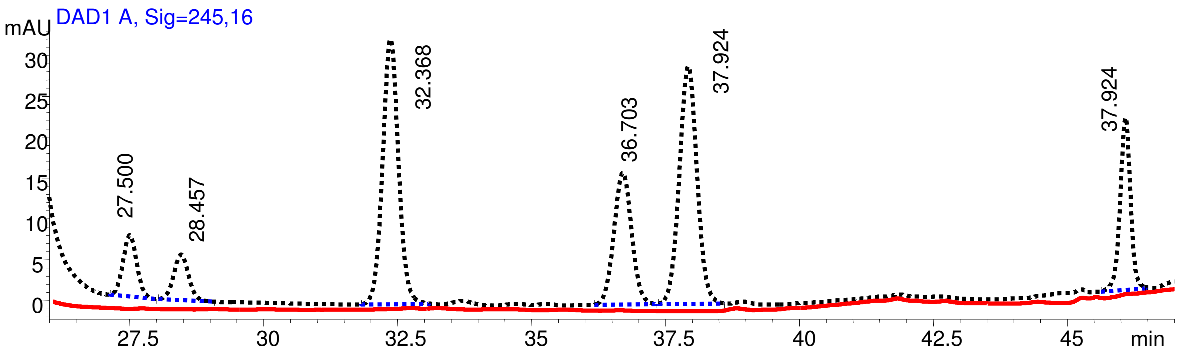

FIGURA 48 – SOBREPOSIÇÃO DOS CROMATOGRAMAS DO PLACEBO E AMOSTRA GEL
LEGENDA: Pontilhado – amostra; Vermelho – placebo

A inexistência de interferentes no cromatograma do placebo em tempos de retenção dos AOP, associado aos testes já descritos de comparação dos perfis espectrais e avaliação de pureza de pico, itens 5.4.1 e 5.4.2, comprova a especificidade/seletividade do método.

5.4.6.2 Linearidade e intervalo

Para verificar a possibilidade de empregar a mesma faixa linear de trabalho utilizada no método CLAE das cascas e tintura, foram avaliadas as respostas de três curvas analíticas de mitrafilina na faixa de 8,0 a 210,0 μg.mL^{-1}. O resultado obtido apresentou coeficiente de correlação de 0,9997, superior ao exigido pela ANVISA (0,99), FIGURA 49 (BRASIL, 2003). O método apresentou linearidade na faixa de concentração da mitrafilina de 8,29 a 207,31 μg.mL^{-1}.

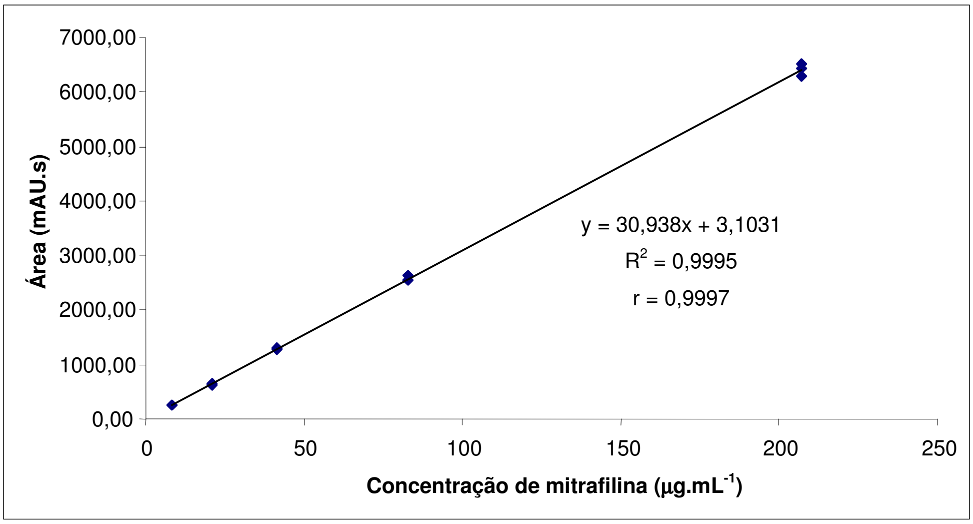

FIGURA 49 – FAIXA LINEAR DE TRABALHO OBTIDA PARA O MÉTODO GEL

Já a linearidade foi obtida com amostras preparadas de maneira a simular as concentrações contidas no intervalo de 80 a 120% para o teor de AOP no gel. O gráfico obtido pela correlação da Concentração de alcalóides injetada (µg.mL^{-1}) Vs Área dos picos (mAU.s), apresentou r = 0,9927, FIGURA 50.

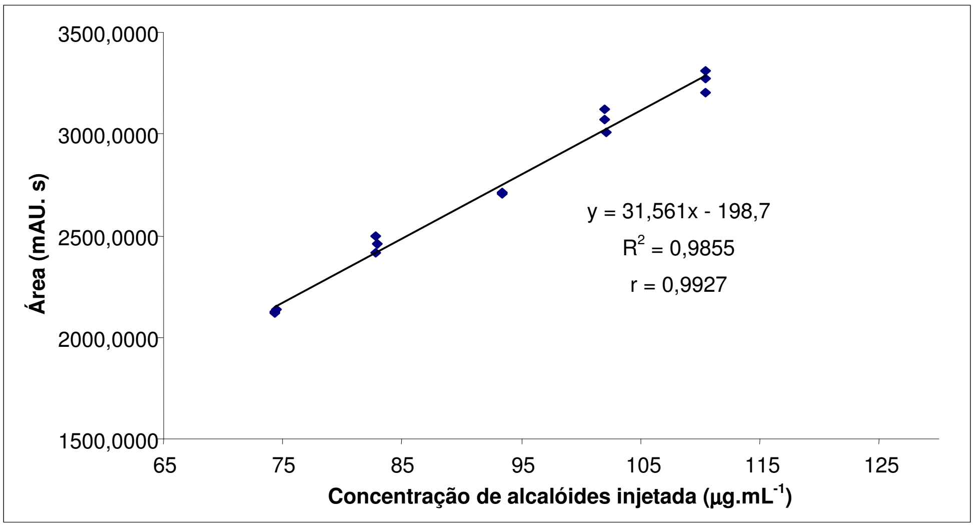

FIGURA 50 – LINEARIDADE – VALIDAÇÃO DO GEL

Mais uma vez, o coeficiente de correlação é superior ao exigido pela ANVISA (0,99) e inferior ao exigido pelo FDA (0,999) (FDA, 2001; BRASIL, 2003). No entanto, conforme Pimentel e Neto (1996), a avaliação do coeficiente de correlação deve ser sucedida pela elaboração e avaliação do gráfico de resíduos, se este apresentar pontos aleatoriamente dispersos, existe forte evidência da adequabilidade do modelo proposto.

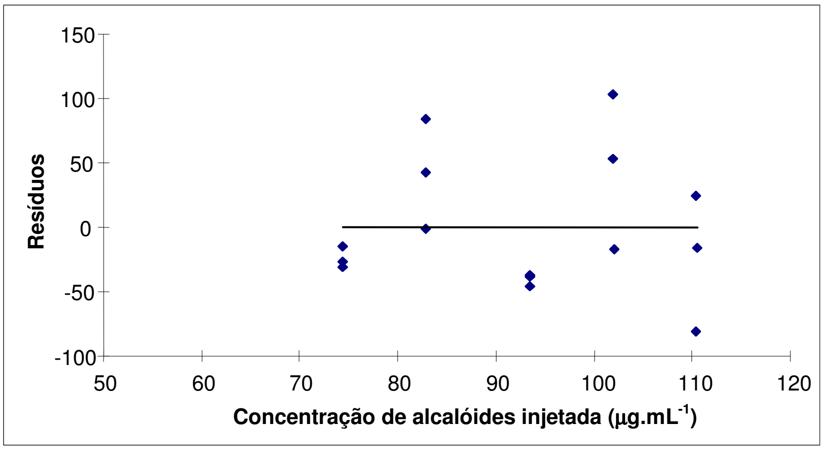

FIGURA 51 – DISPERSÃO DOS RESÍDUOS – VALIDAÇÃO DO GEL

O gráfico de resíduos, FIGURA 51, apresentou pontos aleatoriamente dispersos, portanto é possível confirmar a capacidade do método em fornecer resultados lineares na faixa de 68,87 a 105,00 μg de AOP / mL de solução injetada, o que corresponde a 30,21 a 45,43 μg de AOP / g de amostra.

5.4.6.3 Repetibilidade

A avaliação da repetibilidade a 100% da concentração esperada para AOP na amostra apresentou os resultados da TABELA 27.

TABELA 27 – REPETIBILIDADE – VALIDAÇÃO DO GEL

REPLICATA	ÁREA (mAU.s)	RESULTADO CROMATOGRAMA ($\mu g.mL^{-1}$)	TEOR DE AOP NO GEL ($\mu g.g^{-1}$)
1	2334,756	77,39	33,94
2	2328,678	77,20	33,71
3	2327,195	77,15	33,82
4	2345,546	77,74	33,96
5	2397,314	79,40	34,90
6	2380,448	78,86	34,63
Média	N.A.	N.A.	34,16
DP	N.A.	N.A.	0,48
DPR (%)	N.A.	N.A.	1,42

NOTA: DP – desvio padrão; DPR – desvio padrão relativo; N.A. – não aplicável

O valor encontrado para o DPR (1,42%) é inferior aos valores aceitos pela ANVISA (5%) e Huber (7,3%) nas concentrações analisadas (HUBER, 2001; BRASIL, 2003).

As triplicatas analisadas para as concentrações 80 e 120% do teste de linearidade, item 5.4.6.2, permitiram comprovar a repetibilidade do método também em presença de maiores ou menores quantidades de alcalóides. Para a concentração 80% o DPR entre a somatória das áreas dos alcalóides na triplicata foi de 0,45%, já para a concentração 120% o DPR foi de 1,64% (BRASIL, 2004; ICH, 2005; USP, 2008).

5.4.6.4 Precisão intermediária

Os dados obtidos para a precisão intermediária, avaliada em dias diferentes por analistas diferentes, apresentaram DPR de 1,96%, confirmando a precisão da metodologia analítica desenvolvida.

TABELA 28 – PRECISÃO INTERMEDIÁRIA – VALIDAÇÃO DO GEL

REPLICATA	ÁREA (mAU.s)	RESULTADO CROMATOGRAMA (μg.mL^{-1})	TEOR DE AOP NO GEL (μg.g^{-1})
1	2334,756	77,39	33,94
2	2328,678	77,20	33,71
3	2327,195	77,15	33,82
4	2345,546	77,74	33,96
5	2397,314	79,40	34,90
6	2380,448	78,86	34,63
7	2407,591	75,92	33,30
8	2394,363	75,50	33,13
9	2533,330	79,90	34,89
10	2539,747	80,12	35,08
11	2516,014	79,40	34,66
12	2511,963	79,24	34,80
Média	N.A.	N.A.	34,24
DP	N.A.	N.A.	0,67
DPR (%)	N.A.	N.A.	1,96

NOTA: DP – desvio padrão; DPR – desvio padrão relativo; N.A. – não aplicável

5.4.6.5 Limite de detecção

A inserção do desvio padrão e coeficiente de angular da curva analítica na Equação 2, item 3.5.4, forneceu o valor de 1,68 μg.mL^{-1} como LD para mitrafilina. A confirmação foi realizada pela diluição da solução padrão até esta concentração, análise e inclusão na curva analítica, FIGURA 52, os novos valores de coeficiente angular e desvio padrão forneceram valor de 1,41 μg.mL^{-1} para LD do alcalóide.

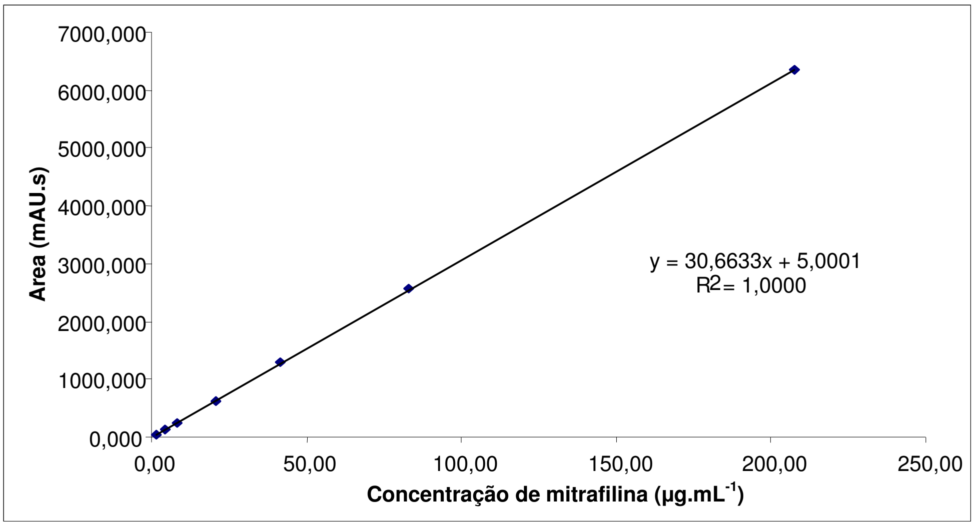

FIGURA 52 – CURVA ANALÍTICA PARA CÁLCULO DO LD DO GEL

5.4.6.6 Limite de quantificação

Os mesmos resultados do item anterior, quando inseridos na Equação 3, item 3.5.4, geraram o valor de 5,10 $\mu g.mL^{-1}$ de mitrafilina para LQ. A diluição do padrão até a concentração encontrada e posterior inclusão na curva analítica confirmou o valor de 4,27 $\mu g.mL^{-1}$ de mitrafilina para LQ.

As sextuplicatas utilizadas para avaliação da precisão e exatidão no nível do LQ, apresentaram 2,13% de DPR e 82,35% de recuperação para área média de 141,404 ± 3,118 mAU.s com resultado médio de 4,36 ± 0,09 $\mu g.mL^{-1}$. A recuperação foi calculada considerando o valor de médio 5,30 $\mu g.mL^{-1}$ para a mitrafilina obtido na avaliação da repetibilidade.

Os valores acima estão dentro dos limites previamente descritos por Huber (2001) como 11% para precisão e 80 a 110% para exatidão na concentração avaliada.

5.4.6.7 Exatidão

Foi utilizada solução de mitrafilina na concentração de 393,00 $\mu g.mL^{-1}$ para contaminar o placebo, perfazendo as concentrações 80, 100 e 120% de AOP esperadas na amostra, TABELA 29.

A mitrafilina foi escolhida por ser o alcalóide de média polaridade em relação aos AO presentes em *U. tomentosa* e assim a observação de seu comportamento no preparo de amostra pode ser mais fidedignamente extrapolado para os demais alcalóides.

TABELA 29 – EXATIDÃO – VALORES DE RECUPERAÇÃO PARA OS AO DO GEL

NÍVEL (%)	MITRAFILINA ADICIONADA (μg)	TEOR ESPERADO ($\mu g.g^{-1}$)	ÁREA (mAU.s)	TEOR OBTIDO ($\mu g.g^{-1}$)	RECUPERAÇÃO MÉDIA ± DP (%)
			2071,781	28,82	
80	149,34	29,87	2150,958	29,92	98,78 ± 2,01
			2140,462	29,77	
			2637,948	36,70	
100	184,71	36,94	2660,995	37,02	100,23 ± 0,89
			2685,428	37,36	
			3228,392	44,91	
120	220,08	44,02	3211,007	44,67	101,82 ± 0,29
			3225,362	44,87	

Os resultados obtidos estão contidos dentro da faixa de recuperação de 80 a 110%, estabelecida como aceitável por Huber (2001) para as concentrações testadas.

5.4.6.8 Robustez

A análise de variância, limite de aceitação de 0,05%, não indicou diferença significativa dos resultados obtidos para o teor de alcalóides ($\mu g.g^{-1}$) das amostras em condições alteradas em relação as amostras controle.

Assim, são atestadas as possibilidades de eluição da amostra após um dia do seu empacotamento na coluna, o uso de 2,5 g de OALN na base da coluna, a estabilidade da amostra após um dia de preparo e emprego de colunas de outros fabricantes ou tamanhos de partículas diferentes para análise cromatográfica (QUADRO 9, item 4.9.7). Na TABELA 30 são apresentados apenas os resultados para os picos críticos uncarina F e pteropodina para as diferentes colunas avaliadas.

TABELA 30 – RESULTADOS OBTIDOS COM AS DIFERENTES COLUNAS TESTADAS NA AVALIAÇÃO DA ROBUSTEZ PARA CLAE DO GEL

		UNCARINA F[1]		PTEROPODINA[1]	
COLUNAS (n=3)	TEOR DE ALCALÓIDES ($\mu g.g^{-1}$)[1]	RESOLUÇÃO[2]	SIMETRIA	RESOLUÇÃO[2]	SIMETRIA
Luna C-18 (2)	34,31 ± 0,56	1,56	0,79	1,76	0,91
Hipersil	34,99 ± 1,03	1,43	0,80	1,74	0,83
Zorbax Eclipse XDB	35,41 ± 0,84	1,63	0,96	2,16	0,99
Zorbax Eclipse XDB (controle)	35,17 ± 1,35	1,99	1,04	2,09	1,07

Nota: [1]Valores médios; [2]em relação ao pico da especiofilina; [3]em relação ao pico da isomitrafilina.

6 CONCLUSÃO

O padrão analítico mitrafilina foi caracterizado com resultados semelhantes aos previamente descritos em literatura. A pureza determinada de 98,25% permite sua utilização como padrão externo em análises quantitativas de *Uncaria tomentosa.*

O método CLAE otimizado mostrou ser adequado para a quantificação de alcalóides oxindólicos em amostras de *U. tomentosa*. Os valores encontrados na validação demonstram adequabilidade do método com boa especificidade / seletividade, linearidade com r >0,998, exatidão próxima a 99%, precisão com DPR inferior a 5%. O tampão orgânico desenvolvido permite separação cromatográfica semelhante ao comumente empregado tampão fosfato, com a vantagem de aumentar o tempo de vida da coluna e reduzir o tempo da análise cromatográfica.

Os resultados obtidos com a curva analítica da mitrafilina demonstraram serem mais próximos ao valor real para alcalóides oxindólicos na planta. A padronização em isopteropodina, amplamente utilizada na Europa e Estados Unidos da América, apresentou resultados superiores aos considerados verdadeiros, superestimando a resposta. O presente trabalho sugere a adoção da padronização da planta frente ao alcalóide mitrafilina ou pelo menos sua inclusão junto à isopteropodina para construção da curva analítica. Dessa forma, os alcalóides da série *R* (mitrafilina, uncarina F, pteropodina e rincofilina) podem ser calculados em função da curva analítica da mitrafilina, e os alcalóides da série *S* (especiofilina, isomitrafilina, isopteropodina e isorincofilina) calculados frente à curva analítica da isopteropodina.

O planejamento fatorial de experimentos executado para otimização do método cromatográfico das cascas e tintura e para o preparo de amostra cascas permitiu construir mapas de todo o domínio experimental proposto e selecionar em cada caso os melhores parâmetros para os métodos.

As análises de espectrometria de massas da planta brasileira e peruana indicaram picos de alcalóides oxindólicos pentacíclicos não descritos em literatura para a espécie *Uncaria tomentosa*. São necessários estudos posteriores para confirmar a identidade das substâncias detectadas.

Finalmente o trabalho permitiu o desenvolvimento do método de dispersão de matriz em fase sólida para o preparo de amostra de um produto farmacêutico com concentração dos seus marcadores na ordem de ppm. Esse emprego para a

técnica ainda não é citado em literatura, constituindo grande campo de aplicação para sanar dificuldades encontradas pelos laboratórios de controle de qualidade na análise de analitos em baixa concentração inseridos em uma matriz complexa.

O método desenvolvido permite a análise dos AOP no gel de maneira confiável, demonstrado pelos resultados da validação que comprovam sua seletividade / especificidade, linearidade (r > 0,99), precisão (DPR < 2%), exatidão (recuperação próxima a 100%) e robustez.

REFERÊNCIAS

AGUILAR, J. L. *et al.* Anti-inflammatory activity of two different extracts of *Uncaria tomentosa* (Rubiaceae). **Journal of Ethnopharmacology**, v.81, p.271-276, 2002.

AKESSON, C. *et al.* An extract of *Uncaria tomentosa* inhibiting cell division and NF-kB activity without inducing cell death. **International Immunopharmacology**, v.3, p.1889-1900, 2003.

AQUINO, R. *et al.* Plant metabolites. new compounds and anti-inflammatory activity of *Uncaria tomentosa*. **Journal of Natural Products**, v.54, n.2, p.453-459, 1991.

AQUINO, R.; SIMONE, F.; PIZZA, C. Plant metabolites. structure and in vitro antiviral activity of quinovic acid glycosides from *Uncaria tomentosa* and *Guettarda platypoda*. **Journal of Natural Products**, v.52, n.4, p.679-685, 1989.

BACHER, N. *et al.* Oxindole alkaloids from *Uncaria tomentosa* induce apoptosis in proliferating, G0/G1-arrested and bcl-2-expressing acute lymphoblastic leukaemia cells. **British Journal of Haematology**, v.132, p.615-622, 2005.

BARKER, S. A. Matrix solid-phase dispersion. **Journal of Chromatography A**, v.885, p.115-127, 2000.

______. Matrix solid phase dispersion (MSPD). **Journal of Biochemical and Biophysical Methods**, v.70, p.151-162, 2007.

BECKETT, A. H.; DWUMA-BADU, D.; HADDOCK, R. E. Some new mitragyna-type indoles and oxindoles; the influence of stereochemistry on mass spectra. **Tetrahedron**, v.25, p.5961-5969, 1969.

BOGIALLI, S.; DI CORCIA, A. Matrix solid-phase dispersion as a valuable tool for extracting contaminants from foodstuffs. **Journal of Biochemical and Biophysical Methods**, v.70, p.163-179, 2007.

BOLDI, A. M. Libraries from natural product-like scaffolds. **Current Opinion in Chemical Biology**, v.8, p.281-286, 2004.

BRASIL. SANITÁRIA, A. N. D. V. Resolução-RE n. 899, de 29 de maio de 2003. Dispõe sobre o Guia para validação de métodos analíticos e bioanalíticos. **Diário Oficial da União.** Brasilia, DF, 02 jun. 2003. Disponível em: <http://e-legis.anvisa.gov.br/leisref/public/showAct.php?id=15132&word=>. Acesso em: 17/4/2008.

______. SANITÁRIA, A. N. D. V. Resolução-RDC n. 48, de 16 de março de 2004. Dispõe sobre o registro de medicamentos fitoterápicos. **Diário Oficial da União.** Brasilia, DF, 18 mar. 2004. Disponível em: <http://e-legis.anvisa.gov.br/leisref/public/showAct.php?id=10230&word=>. Acesso em: 17/4/2008.

CALIXTO, J. B. Twenty-five years of research on medicinal plants in Latin America a personal view. **Journal of Ethnopharmacology**, v.100, p.131-134, 2005.

CARBONEZI, C. A. *et al.* Determinação por RMN das configurações relativas e conformações de alcalóides oxindólicos isolados de *Uncaria guianensis*. **Química nova**, v.27, n.6, p.878-881, 2004.

CASTAÑEDA, O. *et al.* Uña de gato en artritis reumatoide: estudio doble ciego, en comparación con placebo. **Revista Peruana de Reumatología**, v.4, n.1, p.15-21, 1998.

CHAN, K. C.; MORSINGH, F.; YEOH, G. B. Alkaloids of *Uncaria pterodopa*. isolation and structures os pteropodine and isopteropodine. **Journal of the Chemical Society C: Organic**, p.2245-2249, 1966.

CHANDRAN, S.; SINGH, R. S. P. Comparison of varions international guidelines for analytical method validation. **Pharmazie**, v.62, p.4-14, 2007.

CIOLA, R. **Fundamentos da cromatografia a líquido de alto desempenho**. 1. ed. São Paulo: Edgard Blücher LTDA, 1998.

CLAS, S. D.; DALTON, C. R.; HANCOCK, B. C. Differential scanning calorimetry: applications in drug development. **Pharmaceutical Science and Technology Today**, v.2, n.8, p.311-320, 1999.

COLLINS, C. H. Princípios básicos de cromatografia. In: COLLINS, C. H.; BRAGA, G. L.; BONATO, P. S. (Org.). **Fundamentos de cromatografia**. 6. ed. Campinas: Editora Unicamp, 2006. p.17-45.

DEWICK, P. M. **Medicinal natural products: a biosynthetic approach**. 1st. ed. New York: John Wiley & Sons, 1997.

ERMER, J. Validation in pharmaceutical analysis. part I: an integrated approach. **Journal of Pharmaceutical and Biomedical Analysis**, v.24, p.755-767, 2001.

FALKIEWICZ, B.; LUKASIAK, J. Vilcacora [*Uncaria tomentosa* (Willd.) D.C. and *Uncaria guianensis* (Aublet) Gmell.] - a review of published scientific literature. **Case Reports and Clinical Practice Review** v.2, n.4, p.305-316, 2001.

FAROONGSARNG, D.; KADEJINDA, W.; SUNTHORNPIT, A. Thermal behavior of a pharmaceutical solid acetaminophen doped with p-aminophenol. **AAPS PharmScitech**, v.1, n.3, 2000. Disponível em: <http://www.aapspharmscitech.org/articles/pt0103/pt010323/pt010323.pdf>. Acesso em: 01/08/2009.

FOOD AND DRUG ADMINISTRATION (FDA). Technical reviwe guide: validation of chromatographic methods. Rockville, 2001. Disponível em: <www.fda.gov/Cder/guidance/cmc3.pdf>. Acesso em: 05/08/2009.

FERREIRA, S. L. C. *et al.* Statistical designs and response surface techniques for the optimization of chromatographic systems. **Journal of Chromatography A**, v.1158, p.2-14, 2007.

GANZERA, M. *et al.* Improved method for the determination of oxindole alkaloids in *Uncaria tomentosa* by high performance liquid chromatography. **Planta Medica**, v.67, p.447-450, 2001.

GARCIA-LOPEZ, M.; CANOSA, P.; RODRIGUEZ, I. Trends and recent applications of matrix solid-phase dispersion. **Analytical and Bioanalytical Chemistry**, v.391, n.3, p.963-974, 2008.

GARRIDO-LOPEZ, A.; TENA, M. T. Experimental design approach for the optimisation of pressurised fluid extraction of additives from polyethylene films. **Journal of Chromatography A**, v.1099, p.75-83, 2005.

GATTUSO, M.; DI SAPIO, O.; LI PEREYRA, E. Morphoanatomical studies of *Uncaria tomentosa* and *Uncaria guianensis* bark and leaves. **Phytomedicine**, v.11, p.213-223, 2004.

GFRERER, M.; LANKMAYR, E. Screening, optimization and validation of microwave-assisted extraction for the determination of persistent organochlorine pesticides. **Analytica Chimica Acta**, v.533, p.203-2011, 2005.

GONÇALVES, C.; DINIS, T.; BATISTA, M. T. Antioxidant properties of proanthocyanidins of *Uncaria tomentosa* bark decoction: a mechanism for anti-inflammatory activity. **Phytochemistry**, v.66, p.89-98, 2005.

HEITZMAN, M. E. *et al.* Ethnobotany, phytochemistry and pharmacology of *Uncaria* (Rubiaceae). **Phytochemistry**, v.66, p.5-29, 2005.

HERATH, W. H. M. W. *et al.* Alkaloidal and other constituents of *Uncaria elliptica* and *Canthium dicoccum*. **Phytochemistry**, v.18, p.1385-1387, 1979.

HUBER, L. Validation of analytical methods: review and strategy. Oberkirch, 2001. Disponível em: <http://www.labcompliance.com/solutions/free_literature.aspx?sm=b_d>. Acesso em: 11/07/2008.

INTERNATIONAL CONFERENCE ON THE HARMONIZATION (ICH). Validation of Analytical Procedures. Text and Methodology - Q2(R1). Geneva, 2005. Disponível em: <www.bioforum.org.il/Uploads/Editor/karen/q2_r1_step4.pdf>. Acesso em: 11/03/2009.

INSTITUTO NACIONAL DE METROLOGIA, NORMALIZAÇÃO E QUALIDADE INDUSTRIAL (INMETRO). Orientações sobre validação de métodos de ensaios químicos: DOQ-CGCRE-008. Rio de Janeiro, 2007. Disponível em: <http://www.inmetro.gov.br/Sidoq/Arquivos/CGCRE/DOQ/DOQ-CGCRE-8_02.pdf>. Acesso em: 12/02/2009.

JARDIM, I. C. S. F.; COLLINS, C. H.; GUIMARÃES, L. F. L. Cromatografia líquida de alta eficiência. In: COLLINS, C. H.; BRAGA, G. L.; BONATO, P. S. (Org.). **Fundamentos de cromatografia**. 6. ed. Campinas: Editora Unicamp, 2006. p.273-398.

JING-SHAN, S. *et al.* Pharmacological actions of *Uncaria* alkaloids, rhynchophylline and isorhynchophylline. **Acta Pharmacologica Sinica**, v.24, n.2, p.97-101, 2003.

JOLLIFFE, G. H.; SHELLARD, E. J. Separation of some oxindole alkaloids using high-efficiency liquid chromatography. **Journal of Chromatography**, v.81, p.150-151, 1973.

JURGENSEN, S. *et al.* Involvement of 5-Ht_2 receptors in the antinociceptive effect of *Uncaria tomentosa*. **Pharmacology, Biochemistry and Behavior**, v.81, p.466-477, 2005.

KEPLINGER, K. *et al. Uncaria tomentosa* (Willd.) D.C. - ethnomedicinal use and new pharmacological, toxicological and botanical results. **Journal of Ethnopharmacology**, v.64, p.23-34, 1999.

KRISTENSON, E. M.; RAMOS, L.; BRINKMAN, U. A. T. Recent advances in matrix solid-phase dispersion. **Trends in Analytical Chemistry**, v.25, n.2, p.96-111, 2006.

LAUS, G.; BROSSNER, D.; KEPLINGER, K. Alkaloids of peruvian *Uncaria tomentosa*. **Phytochemistry**, v.45, p.855-860, 1997.

LAUS, G. *et al.* Analysis of the kinetics of spiro oxindole alkaloids. **Journal of the Chemical Society**, v.2, p.1931-1936, 1996.

LAUS, G.; KEPLINGER, D. Separation of stereoisomeric oxindole alkaloids from *Uncaria tomentosa* by high performance liquid chromatography. **Journal of Chromatography A**, v.662, p.243-249, 1994.

LAUS, G.; KEPLINGER, K. Radix *Uncarie tomentosae* (Will.) DC. - eine monographische beschreibung. **Zeitschrift fur Phytotherapie**, v.18, p.122-126, 1997.

LAUS, G.; WURST, K. X-ray crystal structure of oxindole alkaloids. **Helvetica Chimica Acta**, v.86, p.181-187, 2003.

LIAZID, A. *et al.* Optimization of a new extraction technique for analysis of verbenone and *cis*-verbenol in pine seeds. **Chromatographia**, v.66, p.571-575, 2007.

LOPEZ-AVILA, V.; BENEDICTO, J. Supercritical fluid extraction of oxindole alkaloids from *Uncaria tomentosa*. **Journal of High Resolution Chromatography**, v.20, p.231-236, 1997.

MAZZEI, J. L. **Transposição de escala por modelos na produção de substâncias naturais por cromatografia líquida de alta eficiência**. 271 f. Tese (Doutorado em

Tecnologia de Processos Químicos e Bioquímicos) - Escola de Química, Universidade Federal do Rio de Janeiro, Rio de Janeiro, 2004.

MENDHAM, J. *et al.* **Vogel - análise química quantitativa**. 6. ed. Rio de Janeiro: LTC, 2002.

MUHAMMAD, I. *et al.* Two stereoisomeric pentacyclic oxindole alkaloids from *Uncaria tomentosa*: uncarine c and uncarine e. **Acta Crystallographica**, v.57, p.480-482, 2001.

NETO, B. B.; SCARMINO, I. S.; BRUNS, R. E. **Como fazer experimentos**. 2. ed. Campinas: Editora Unicamp, 2003.

NEWMAN, D. J.; CRAGG, G. M.; SNADER, K. M. Natural products as sources of new drugs over the period 1981-2002. **Journal of Natural Products**, v.66, p.1022-1037, 2003.

PELLATI, F. *et al.* Headspace solid-phase microextraction-gas chromatography-mass spectrometry analysis of the volatile compounds of *Evodia* species fruits. **Journal of Chromatography A**, v.1087, p.265-273, 2005.

PEREIRA, R. C. A. *et al.* In vitro cultivated *Uncaria tomentosa* and *Uncaria guianensis* with determination of the pentacyclic oxindole alkaloid contents and profiles. **Journal of Brazilian Chemical Society**, v.19, n.6, p.1193-1200, 2008.

PERSON, B.; VESSMAN, J. Generating selectivity in analytical chemistry to reach the ultimate specificity. **Trends in analytical chemistry**, v.17, n.3, p.117-119, 1998.

PHILLIPSON, J. D.; HEMINGWAY, S. R. Oxindole alkaloids from *Uncaria macrophylla*. **Phytochemistry**, v.12, p.2795-2798, 1973.

______. Alkaloids of *Uncaria attenuata, U. orientalis* and *U. canescens*. **Phytochemistry**, v.14, p.1855-1863, 1975a.

______. Chromatographic and spectroscopic methods for the identification of alkaloids from herbarium samples of the genus *Uncaria*. **Journal of Chromatography**, v.105, p.163-178, 1975b.

PHILLIPSON, J. D.; HEMINGWAY, S. R.; RIDSDALE, C. E. Alkaloids of *Uncaria*. part V. their occurrence and chemotaxonomy. **Lloydia**, v.41, n.6, p.503-570, 1978.

PHILLIPSON, J. D.; SUPAVITA, N.; ANDERSON, L. A. Separation of heteroyohimbine ond oxindole alkaloids by reversed-phase high-performance liquid chromatography. **Journal of Chromatography**, v.244, p.91-98, 1982.

PILARSKI, R. *et al.* Antiproliferative activity of various *Uncaria tomentosa* preparations on HL-60 promyelocytic leukemia cells. **Pharmacological Reports**, v.59, p.565-572, 2007.

______. Antioxidant activity of ethanolic and aqueous extracts of *Uncaria tomentosa* (Willd.) DC. **Journal of Ethnopharmacology**, v.104, p.18-23, 2006.

PIMENTEL, M. F.; BARROS NETO, B. Calibração: uma revisão para químicos analíticos. **Química Nova**, v.19, n.3, p.268-277, 1996.

POLLITO, A. Z.; TOMAZELLO, M. Anatomia do lenho de *Uncaria guianensis* e *U. tomentosa* (Rubiaceae) do estado do Acre, Brasil. **Acta Amazonica**, v.36, n.2, p.169-176, 2006.

REINHARD, K. H. *Uncaria tomentosa* (Willd.) DC. - cat's claw, uña de gato oder katzenkralle. **Zeitschrift fur Phytotherapie**, v.18, p.112-121, 1997.

REIS, S. R. I. N. *et al.* Immunomodulating and antiviral activities os *Uncaria tomentosa* on human monocytes infected with dengue virus-2. **International Immunopharmacology**, v.8, p.468-476, 2008.

RIBANI, M. *et al.* Validação em métodos cromatográficos e eletroforéticos. **Química nova**, v.27, n.5, p.771-780, 2004.

RIZZI, R. *et al.* Mutagenic and antimutagenic activities of *Uncaria tomentosa* and its extracts. **Journal of Ethnopharmacology**, v.38, p.63-77, 1993.

ROWE, R. C.; SHESKEY, P. J.; WELLER, P. **Handbook of pharmaceutical excipients**. 4th. ed. London: Pharmaceutical Press, 2003.

SANDOVAL, M. *et al.* Anti-inflammatory and antioxidant activities of cat´s claw (*Uncaria tomentosa* and *Uncaria guianensis*) are independent of their alkaloid content. **Phytomedicine**, v.9, p.325-337, 2002.

SEKI, H. *et al.* A nuclear magnetic resonance study on the eleven steroisomers of heteroyohimbine-type oxindole alkaloids. **Chemical and Pharmaceutical Bulletin**, v.41, n.12, p.2077-2086, 1993.

SHABIR, G. A. Validation of high-performance liquid chromatography methods for pharmaceutical analysis. Understanding the differences and similarities between validation requirements of the US food and drug administration, the US pharmacopeia and the international conference on harmonization. **Journal of Chromatography A**, v.987, p.57-66, 2003.

SHAMMA, M. *et al.* The stereochemistry of the pentacyclic oxindole alkaloids. **Journal of the American Chemical Society**, v.89, n.7, p.1739-1740, 1967.

SHELLARD, E. J.; TANTIVATANA, P.; BECKETT, A. H. The *Mitragyna* species of Asia. part. X. the alkaloids of the leaves of *Mitragyna hirsuta* Havil. **Planta Medica**, v.15, p.366-370, 1967.

SHENG, Y. *et al.* An active ingredient of cat's claw water extracts identification and efficacy of quinic acid. **Journal of Ethnopharmacology**, v.96, p.577-584, 2005.

SKOOG, D. A. *et al.* **Fundamentos de química analítica**. 1. ed. São Paulo: Cengage Learning, 2009.

SOLOMONS, T. W. G.; FRYHLE, C. B. **Química orgânica**. 8. ed. Rio de Janeiro: LTC, v.2, 2006.

STUPPNER, H.; STURM, S. Capillary electrophoretic analysis of oxindole alkaloids from *Uncaria tomentosa*. **Journal of Chromatography**, v.609, p.375-380, 1992.

STUPPNER, H. *et al.* A differential sensitivity of oxindole alkaloids to normal and leukemic cell lines. **Planta Medica**, v.59, n.Suplement issue, p.A 583, 1993.

STUPPNER, H.; STURM, S.; KONWALINKA, G. HPLC analysis of the main oxindole alkaloids from *Uncaria tomentosa*. **Chromatographia**, v.34, n.11/12, p.597-600, 1992.

TAVERNIERS, I.; LOOSE, M. D.; BOCKSTELE, E. V. Trends in quality in the analytical laboratory. II. analytical method validation and quality assurance. **Trends in analytical chemistry**, v.23, n.8, p.535-552, 2004.

THOMPSON, M.; ELLISON, S. L. R.; WOOD, R. Harmonized guidelines for single-laboratory validation of methods of analysis. **Pure and Applied Chemistry**, v.74, n.5, p.835-855, 2002.

TIRILLINI, B. Fingerprints of *Uncaria tomentosa* leaf, stem and root bark decoction. **Phytotherapy Research**, v.10, p.S67-S68, 1996.

TOURE, H. *et al.* Complete ^{1}H and ^{13}C NMR chemical shift assignments for some pentacyclic oxindole alkaloids. **Spectroscopy Letters**, v.25, n.2, p.293-300, 1992.

USP - THE UNITED STATES PHARMACOPEIA. 31. ed. Rockville. United States Pharmacopeial Convention, 2008.

VALENTE, L. M. M. *et al.* Desenvolvimento e aplicação de metodologia por cromatografia em camada delgada para determinação do perfil de alcalóides oxindólicos pentacíclicos nas espécies sul-americanas do gênero *Uncaria*. **Revista Brasileira de Farmacognosia**, v.16, n.2, p.216-223, 2006.

VALENTE SOARES, L. M. Como obter resultados confiáveis em cromatografia. **Revista do Instituto Adolfo Lutz**, v.60, n.1, p.79-84, 2001.

VILCHES, L. E. O. **Cat's claw "uña de gato"**. 1. ed. Lima: Instituto de Fitoterapia Americano, 1995.

WAGNER, H.; BLADT, S. Uncarie radix. In: (Org.). **Plant and drug analysis: a thin layer chromatography atlas**. 2. ed. Berlin: Springer, 1996. p.30-31.

WAGNER, H.; KREUTZKAMP, B.; JURCIC, K. Die alkaloide von *Uncaria tomentosa* und ihre phagozytose-steigernde wirkung. **Planta Medica**, v.51, p.419-423, 1985.

WILLIAMS, J. E. Review of antiviral and immunomodulating properties of plants of the peruvian rainforest with a particular emphasis on uña de gato and sangre de grado. **Alternative Medicine Review**, v.6, n.6, p.567-579, 2001.

WURM, M. *et al.* Pentacyclic oxindole alkaloids from *Uncaria tomentosa* induce human endothelial cells to release a lymphocyte-proliferation-regulating factor. **Planta Medica**, v.64, p.701-704, 1998.

ZHANG, W. B.; CHEN, C. X.; SIM, S. M. In vitro vasodilator mechanisms of the indole alkaloids rhynchophylline and isorhynchophylline isolated from the hook of *Uncaria rhynchophylla* (Miquel). **Naunyn-Schmiedeberg's Archives of Pharmacology**, v.369, p.232-238, 2004.

ANEXOS

ANEXO 1 - IDENTIFICAÇÃO BOTÂNICA DA PLANTA BRASILEIRA - *Uncaria tomentosa*, DC.

Instituto de Botânica de São Paulo
Curadoria do Herbário
C. P. 4005
01061-970 São Paulo SP Brasil Fax: (011) 5073-6300 R. 302
Tel: (011) 5073-6300 R. 281

LAUDO DE IDENTIFICAÇÃO

A amostra de planta que nos foi submetida à identificação pela CENTROFLORA, de número 171, cujo nome popular indicado é unha-de-gato, trata-se de:

Uncaria tomentosa (Willd. ex Roem. & Schltz) DC. (Família Rubiaceae)

Dra. Inês Cordeiro
Pesquisador Científico

São Paulo, 23 de setembro de 2004.

Obs. ESTE LAUDO DE IDENTIFICAÇÃO É RESULTADO DA ANÁLISE DE UMA ÚNICA AMOSTRA. Fica proibida a menção ou utilização do nome ou sigla do Instituto de Botânica, em qualquer tipo de publicidade comercial, seja qual for o veículo publicitário, principalmente se o nome do Instituto for utilizado para qualificar produção industrial sobre a qual o mesmo não exerça qualquer meio de controle.

ANEXO 2 - IDENTIFICAÇÃO BOTÂNICA DA PLANTA PERUANA - *Uncaria tomentosa*, DC.

UNIVERSIDAD NACIONAL MAYOR DE SAN MARCOS
Universidad del Perú, DECANA DE AMÉRICA
MUSEO DE HISTORIA NATURAL

89 años estudiando al Perú

CONSTANCIA N°047-USM-2008

LA JEFA DEL HERBARIO SAN MARCOS (USM) DEL MUSEO DE HISTORIA NATURAL, DE LA UNIVERSIDAD NACIONAL MAYOR DE SAN MARCOS, DEJA CONSTANCIA QUE:

La muestra vegetal (planta completa) recibida de la **Empresa PERUVIAN HEITAGE S.A.C.**, ha sido estudiada y clasificada como: ***Uncaria tomentosa (Willd. ex Roem. & Shult.) DC.***, y tiene la siguiente posición taxonómica, según el Sistema de Clasificación de Cronquist (1988):

DIVISION: MAGNOLIOPHYTA

CLASE: MAGNOLIOPSIDA

ORDEN: RUBIALES

FAMILIA: RUBIACEAE

GENERO: ***Uncaria***

ESPECIE: ***Uncaria tomentosa (Willd. ex Roem. & Shult.) DC.***

Nombre vulgar: "Uña de gato"
Determinada por: Mg. Betty Millán S.

Se extiende la presente constancia a solicitud de la parte interesada, para fines de exportación.

Lima, 22 de Mayo de 2008

MUSEO DE HISTORIA NATURAL - JEFE - Herbario San Marcos - USM

Mg. J[illegible]aquina Albán Castillo
[illegible]A DEL [illegible]ERBARIO SAN MARCOS
USM

DDB

Av. Arenales 1256, Jesús María
Apdo. 14-0434, Lima 14, Perú
Telfs.: (511) 471-0117, 470-4471, 470-7918, 619-7000 anexo 5703
Fax: (511) 265-6819
e-mail: museohn@unmsm.edu.pe
http://museohn.unmsm.edu.pe
